# Formation of Heterobimetallic Complexes by Addition of d^10^-Metal Ions to [(Me_3_P)_*x*_M(2-C_6_F_4_PPh_2_)_2_] (*x* = 1, 2; M = Ni and Pt): A Synthetic and Computational Study of Metallophilic Interactions

**DOI:** 10.1021/acs.inorgchem.3c00311

**Published:** 2023-05-31

**Authors:** Robert Gericke, Martin A. Bennett, Steven H. Privér, Suresh K. Bhargava

**Affiliations:** † Centre for Advanced Materials and Industrial Chemistry, School of Applied Sciences (Applied Chemistry), RMIT University, GP.O. Box 2476, Melbourne, Victoria 3001, Australia; ‡ Institute of Resource Ecology, Helmholtz-Zentrum Dresden-Rossendorf e. V., Bautzner Landstraße 400, Dresden 01328, Germany; § Research School of Chemistry, Australian National University, Canberra ACT 2601, Australia

## Abstract

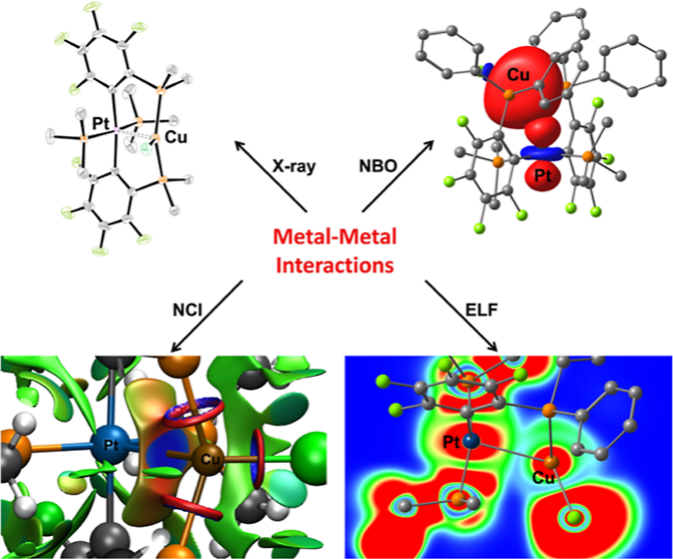

Treatment of the bis(chelate) complexes *trans*-[M(κ^2^-2-C_6_F_4_PPh_2_)_2_] (*trans*-**1M**; M = Ni, Pt) and *cis*-[Pt(κ^2^-2-C_6_F_4_PPh_2_)_2_] (*cis*-**1Pt**) with equimolar amounts or excess of PMe_3_ solution gave complexes of the type [(Me_3_P)_*x*_M(2-C_6_F_4_PPh_2_)_2_] (*x* = 2: **2M**^**a**^, **2M**^**b**^*x* = 1: **3M**^**a**^, **3M**^**b**^; M = Ni, Pt). The reactivity of complexes of the type **2M** and **3M** toward monovalent coinage metal ions (M′ = Cu, Ag, Au) was investigated next to the reaction of **1M** toward [AuCl(PMe_3_)]. Four different complex types [(Me_3_P)_2_M(μ-2-C_6_F_4_PPh_2_)_2_M′Cl] (**5MM′**; M = Ni, Pt; M′ = Cu, Ag, Au), [(Me_3_P)M(κ^2^-2-C_6_F_4_PPh_2_)(μ-2-C_6_F_4_PPh_2_)M′Cl]_*x*_ (*x* = 1: **6MM′**; M = Pt; M′ = Cu, Au; *x* = 2: **6PtAg**), head-to-tail-[(Me_3_P)ClM(μ-2-C_6_F_4_PPh_2_)_2_M′] (**7MM′**; M = Ni, Pt; M′ = Au), and head-to-head-[(Me_3_P)ClM(μ-2-C_6_F_4_PPh_2_)_2_M′] (**8MM′**; M = Ni, Pt; M′ = Cu, Ag, Au) were observed. Single-crystal X-ray analyses of complexes **5**–**8** revealed short metal–metal separations (2.7124(3)–3.3287(7) Å), suggestive of attractive metal–metal interactions. Quantum chemical calculations (atoms in molecules (AIM), electron localization function (ELF), non-covalent interaction (NCI), and natural bond orbital (NBO)) gave theoretical support that the interaction characteristics reach from a pure attractive non-covalent to an electron-shared (covalent) character.

## Abstract 2

A series of related mono and double bridged heterobimetallic d^8^−d^10^ complexes with a [MM′]^3+^ core (M = Ni, Pt; M′ = Cu, Ag, Au) have been synthesized. Dependent on the synthesis strategy, a variety of isomers of the composition [(Me_3_P)_*x*_M(2-C_6_F_4_PPh_2_)_2_M′Cl] (*x* = 1, 2) was isolated. All synthesized compounds feature short metal−metal separations (2.7124(3)−3.3287(7) Å). Metallophilic bonding trends were discussed along the isomeric series using DFT analysis (NCI, ELF, NBO).

## Introduction

**Chart 1:**
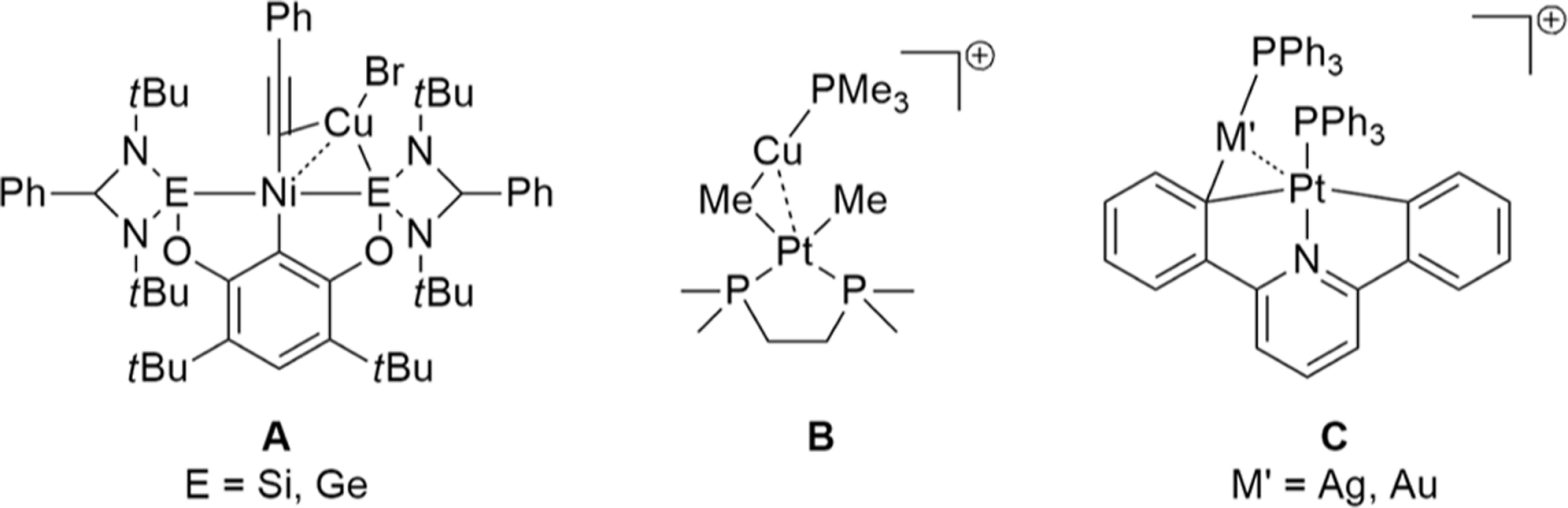
Examples of Complexes Featuring d^8^–d^10^-Metal Pairs

Heterobimetallic compounds are of great interest due to their tuneable metal–metal bonds or interactions.^1^ Hence, the chemistry of heterobinuclear complexes featuring two transition metals give rise to various intriguing redox, spectroscopic, and photophysical properties, which lead to a wide range of applications.^2^ These features extend the scope of their monometallic counterparts. Such binuclear complexes bearing d^8^–d^8^, d^8^–d^10^, or d^10^–d^10^ metal pairs (d^8^: Ir^I^, Ni^II^, Pd^II^, Pt^II^, Au^III^; d^10^: Cu^I^, Ag^I^, Au^I^, Hg^II^) have been generated unsupported or supported by bridging ligands.^3^ Heterobimetallic systems featuring groups 10 and 11 metals are of current interest due to their key role in cooperative catalysis.^4^ Recent success in trapping a reactive intermediate was reported for a Ni-catalyzed cross-coupling reaction, where a Ni–Cu complex could be isolated as a representative snapshot of the catalytic cycle (Chart 1, **A**).^5^ Similarly, transmetalation from methyl groups from a Pt^II^ toward a Au^I^ center was reported to proceed via direct Pt^II^–Au^I^ bond formation (supported by mass spectrometric data).^6^ Structural information of such reactive intermediates is given for the homologue Pt^II^–Cu^I^ complex (Chart 1, **B**) and analogue Pt^II^–Ag^I^ and Pt^II^–Au^I^ complexes (Chart 1, **C**) where in solution a dynamic coinage metal–carbon bond behavior was observed.^7^

Discrete synthetically prepared d^8^–d^10^ heterobimetallic compounds are important for the fundamental understanding of the relation between the nature of the metal–metal interactions and the complex structure.^8^ Various M–M′ interactions with late transition metals (M, M′) have been studied during the past decades, and they were found to span between long range dispersion (London) energy^9^ and ionic contribution^10^ to metal-to-metal charge transfer^11^ with significant covalency.^12^

**Scheme 1:**
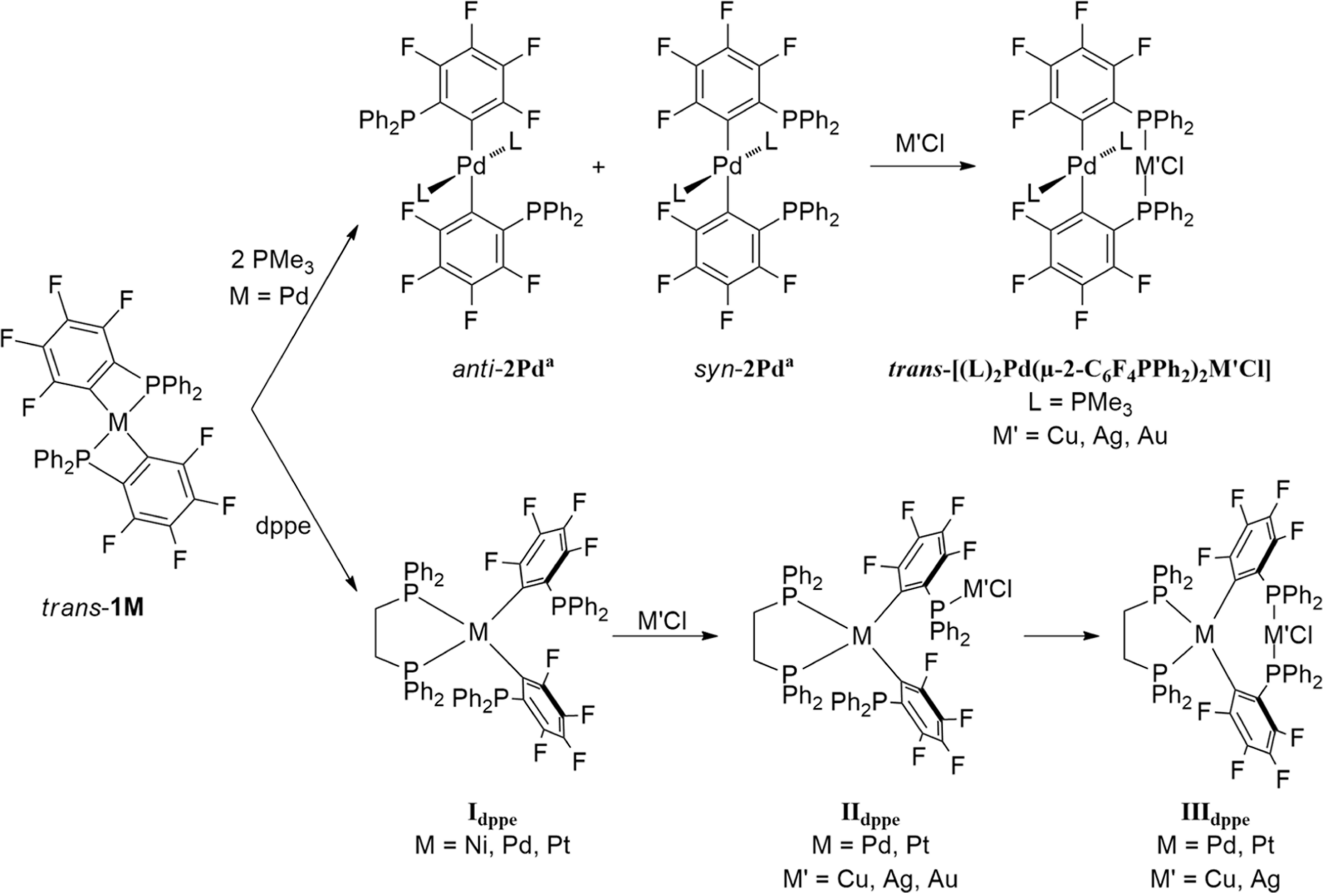
Synthesis of Complexes of Type *trans*-[(Me_3_P)_2_Pd(μ-2-C_6_F_4_PPh_2_)_2_M′Cl], [(dppe)M(κC-2-C_6_F_4_PPh_2_)(μ-2-C_6_F_4_PPh_2_)M′Cl] (**II**_**dppe**_), and [(dppe)M(μ-2-C_6_F_4_PPh_2_)_2_M′Cl] (**III**_**dppe**_)

The complexes *trans*-[M(κ^2^-2-C_6_F_4_PPh_2_)_2_] (M = Ni, Pd, Pt; *trans*-**1M**)^13^ were previously used as a d^8^-metal source to generate such bimetallic complexes.^14,15^ The four-membered rings of the ortho-metalated C,P-ligand can open by treatment of *trans*-**1M** with neutral alkyl phosphines like PMe_3_ or dppe ((diphenylphosphino)ethane), leading to a *cis*- or *trans*-orientation of the dangling κC-2-C_6_F_4_PPh_2_ ligands about the transition-metal center (Scheme 1).

These dangling C,P-ligands show the ability to coordinate coinage metal ions in a mono bridged (**II**_**dppe**_) or double bridge (*trans*-[(Me_3_P)_2_Pd(μ-2-C_6_F_4_PPh_2_)_2_M′Cl], **III**_**dppe**_) manner. Caused by steric crowding, the M···M′ separations are found to be longer for the dppe-containing complexes (2.9104(3)–3.8715(5) Å) in comparison to the PMe_3_-containing compounds (2.7707(11)–2.9423(3) Å). The M···M′ interaction types were reported to be either attractive non-covalent (dispersion) or donor–acceptor interactions. In order to obtain periodic trends for the interaction types among the d^8^-metals of the nickel triads and the d^10^-metal of coinage metal ions, we investigated the hitherto unknown nickel and platinum homologues, using the sterically less demanding PMe_3_ ligand, toward coinage metal ions. Due to platinum’s rich *cis*/*trans*-isomeric chemistry, we were also interested in a synthetic strategy to obtain the heavier homologue *cis*-isomer of *trans*-[(Me_3_P)_2_M(μ-2-C_6_F_4_PPh_2_)_2_M′Cl] and the influence of isomerization toward the M···M′ interaction.

## Results and Discussion

### Complexes of the Type [(Me_3_P)_*x*_M(2-C_6_F_4_PPh_2_)_2_]

**Scheme 2:**
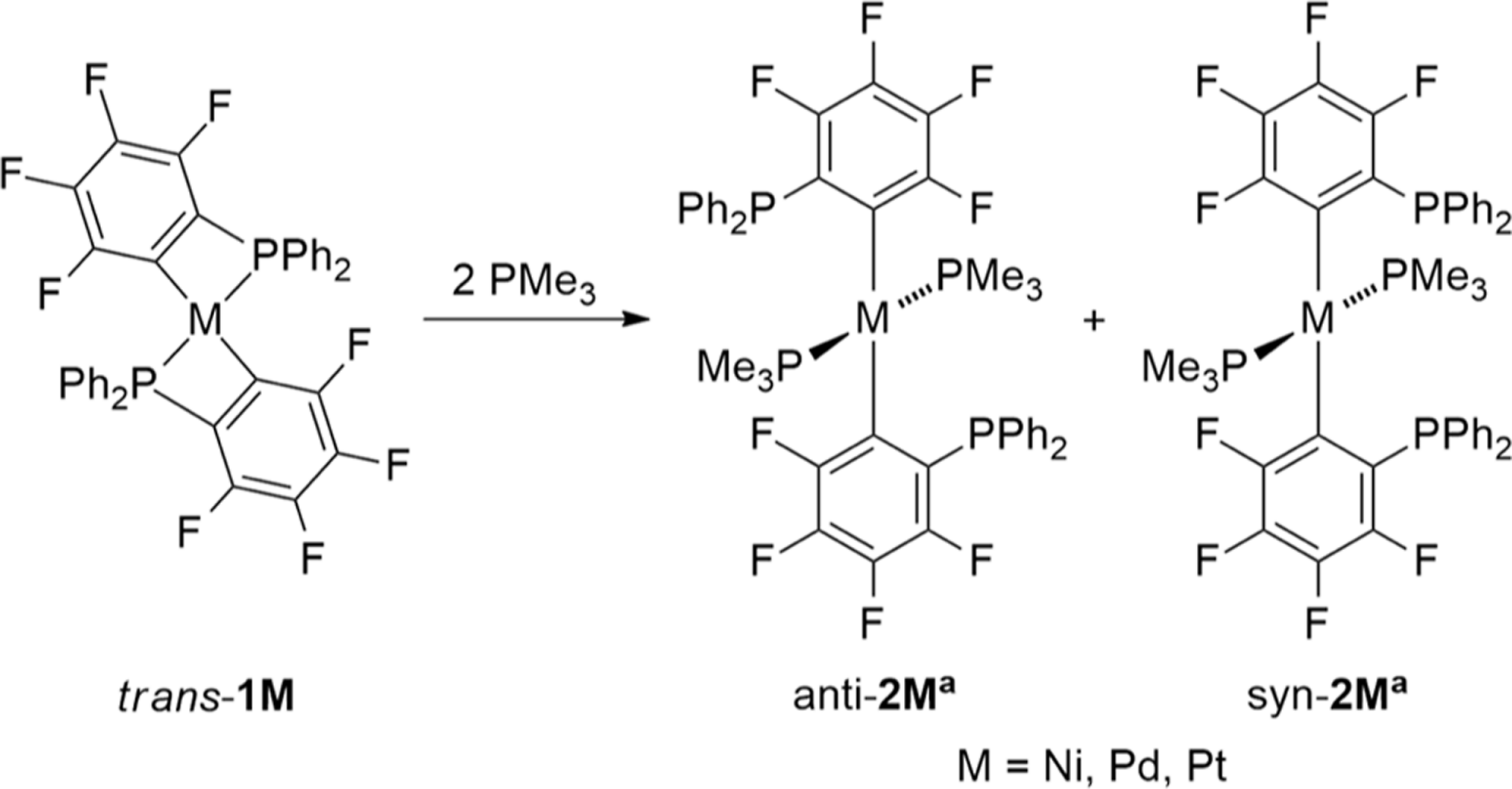
Formation of *trans*-[(Me_3_P)_2_M(κC-2-C_6_F_4_PPh_2_)_2_] (**2M**^**a**^)

The compounds *trans*-[M(κ^2^-2-C_6_F_4_PPh_2_)_2_] (M = Ni, Pd, Pt; *trans*-**1M**) were synthesized following the literature methods.^13^ For M = Pt, the analogous complex *cis*-[Pt(κ^2^-2-C_6_F_4_PPh_2_)_2_] (*cis*-**1Pt**) was formed during the synthesis and could be separated from the *trans*-isomer by fractional crystallization. The reaction of *trans*-**1M** with more than 2 equiv PMe_3_ resulted in the formation of *trans*-[(Me_3_P)_2_M(κC-2-C_6_F_4_PPh_2_)_2_] (**2M**^**a**^, Scheme 2).^14^ The ^31^P NMR spectrum (Table 1) in C_6_D_6_ of **2Ni**^**a**^ is similar to the palladium homologue. **2Ni**^**a**^ potentially appears as a mixture of *anti*-**2Ni**^**a**^ and *syn*-**2Ni**^**a**^-isomer in a 1:8 ratio, similar to that for **2Pd**^**a**^ (1:6 ratio) as previously reported.^14^ Only one isomer was observed for **2Pt**^**a**^ in C_6_D_6_ (Figure S6), whereas in CDCl_3_ a second signal set was visible in the ^31^P NMR spectrum (Figure S7). Our quantum chemical calculations at DFT level of theory have shown that in general the *syn*-isomer is thermodynamically more stable by 3.4–4.3 kcal mol^–1^ (Ni: 4.21, Pd: 3.42, Pt: 4.34 kcal mol^–1^). The magnitude is in agreement with the co-existence of both isomers in solution. Therefore, we assume that the major product detected by NMR spectroscopy could be assigned to the *syn*-isomer, where the isomeric shifts of the dangling PPh_2_ groups are systematically shifted to a higher field by approximately 6 ppm, presumably caused by the proximity of both PPh_2_ groups.

**Table 1:** ^31^P NMR Chemical Shifts (ppm) of Complexes of the Type **2M**^**a**^ (M = Ni, Pd,^14^ Pt) in C_6_D_6_^a^

M	*anti*		*syn*	
	PMe_3_	μ-2-C_6_F_4_PPh_2_	PMe_3_	μ-2-C_6_F_4_PPh_2_
Ni	–15.0	–3.6	–15.0	–9.4
Pd	–19.1	–2.9	–18.7	–8.4
Pt	[−24.4 (2700)^b^]	[−5.4 (160)^b^]	–24.6 (2700)	–11.1 (90)

a *J*_Pt,P_ (Hz) coupling constants are given in brackets.

b ^31^P NMR shifts of the *anti*-isomer were observed in CDCl_3_ solution.

**Scheme 3:**
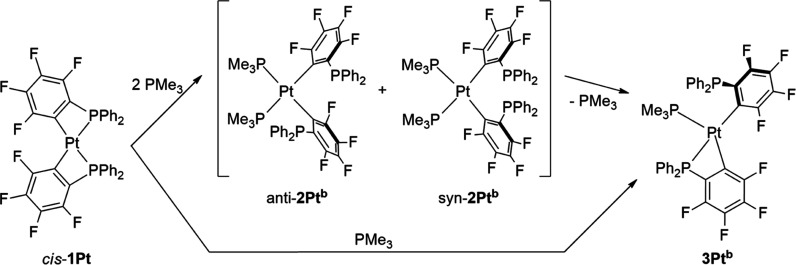
Formation of Intermediate Compounds *cis*-[(Me_3_P)_2_Pt(κC-2-C_6_F_4_PPh_2_)_2_] (**2Pt**^**b**^) and *cis*-[(Me_3_P)Pt(κ^2^-2-C_6_F_4_PPh_2_)(κC-2-C_6_F_4_PPh_2_)] (**3Pt**^**b**^)

The ^31^P NMR spectrum of the crude reaction mixture of *cis*-**1Pt** with excess PMe_3_ in CH_2_Cl_2_ solution (with C_6_D_6_-insert) shows two multiplet resonances at −1.8 ppm (^3^*J*_Pt,P_ ≈ 240 Hz) and −2.8 ppm (^3^*J*_Pt,P_ ≈ 240 Hz) and one resonance at −31.3 ppm (^1^*J*_Pt,P_ = 2170 Hz) in a 1:1:2 ratio. This observation can be assigned to *cis*-[(Me_3_P)_2_Pt(κC-2-C_6_F_4_PPh_2_)_2_] (**2Pt**^**b**^), which occurs also as *anti*- and *syn*-isomer in a 1:1 ratio (ratio is estimated from integrals of ^31^P signals, Scheme 3). Quantum chemical calculations reveal that both *cis* isomers show similar thermodynamic stability (Δ|*E*| ∼2 kcal mol^–1^), thus indicating coexistence of both isomers in equilibrium. However, the *cis*-isomers *anti*/*syn*-**2Pt**^**b**^ are thermodynamically less favored than the corresponding *trans*-isomers *anti*/*syn*-**2Pt**^**a**^ [*syn*-**2Pt**^**b**^ (8.11) > *anti*-**2Pt**^**b**^ (5.54) > *anti*-**2Pt**^**a**^ (4.34) > *syn*-**2Pt**^**a**^ (0.00 kcal mol^–1^)]. Attempts to isolate **2Pt**^**b**^ failed due to loss of PMe_3_ and formation of *cis*-[(Me_3_P)Pt(κ^2^-2-C_6_F_4_PPh_2_)(κ*C*-2-C_6_F_4_PPh_2_)] (**3Pt**^**b**^). **3Pt**^**b**^ can also be synthesized by addition of 1 equiv PMe_3_ to *cis*-**1Pt**. The same behavior has been observed in the reaction of *cis*-[Pt(κ^2^-2-C_6_H_4_PPh_2_)_2_] with excess P(OMe)_3_ or Bu^*t*^NC as reported in the literature.^16^

Interestingly, dissolving a pure sample of *trans*-[(Me_3_P)_2_Ni(κC-2-C_6_F_4_PPh_2_)_2_] (**2Ni**^**a**^) in C_6_D_6_ shows a slight loss of PMe_3_. According to the ^31^P and ^19^F NMR spectra, the characteristic signal pattern of traces (≈5%) of *trans*-[(Me_3_P)Ni(κ^2^-2-C_6_F_4_PPh_2_)_2_] (**3M**^**a**^) could be detected (vide infra).

**Figure 1:**
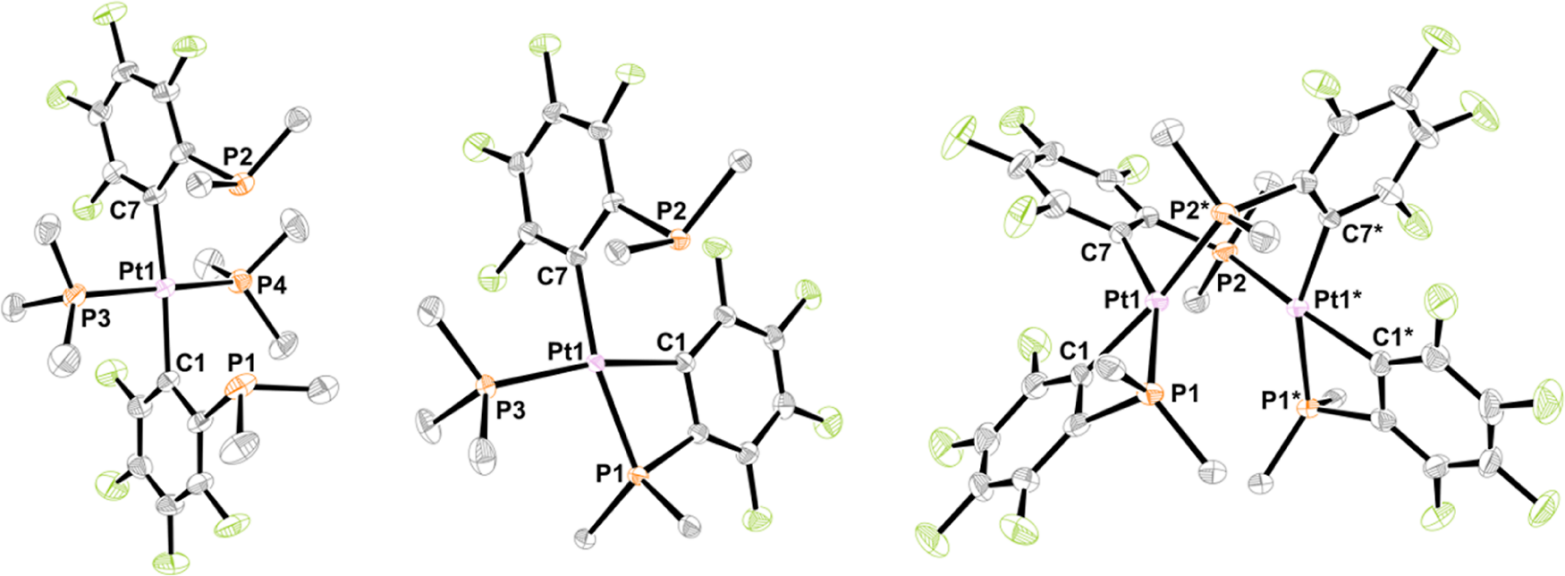
Molecular structures of *syn*-**2Pt**^**a**^, **3Pt**^**b**^, and **4Pt** (left to right). Ellipsoids are shown at 50% probability level. Hydrogen atoms and solvent molecules are omitted, and only the *ipso*-carbons of the PPh_2_ groups are depicted for clarity.

**Scheme 4:**
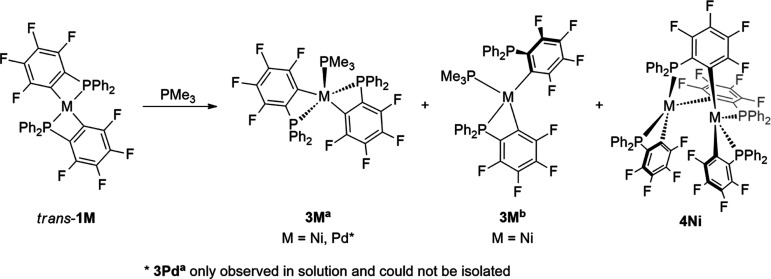
Reaction of *trans*-**1M** with 1 equiv of PMe_3_

In contrast to observations along the nickel triad of the reaction of *trans*-**1M** with excess PMe_3_, the reaction of *trans*-**1M** with 1 equiv PMe_3_ shows a different behavior depending on the transition metal. The ^31^P NMR spectrum of the crude reaction mixture of *trans*-**1Pt** with 1 equiv PMe_3_ shows the characteristic signal pattern of **2Pt**^**a**^ and unreacted *trans*-**1Pt** beside traces of unknown impurities. The ^31^P NMR spectrum of the crude reaction mixture of *trans*-**1Pd** with 1 equiv PMe_3_ shows a 1:1:1 ratio of *trans*-**1Pd** (−55.4 ppm), *syn/anti*-**2Pd**^**a**^ (−18.7, −8.4, −19.2, −2.9 ppm), and **3Pd**^**a**^ (−35.5 ppm, ^2^*J*_P,P_ = 228.7 Hz, doublet, 2P; −13.8 ppm, ^2^*J*_P,P_ = 228.7 Hz, triplet, 1P). The presence of the symmetric isomer **3Pd**^**a**^ could be confirmed by ^19^F NMR spectroscopy (−117.2, −128.6, −152.1, −159.5; Scheme 4). Attempts to isolate **3Pd**^**a**^ from the reaction mixture failed. The crude reaction mixture of *trans*-**1Ni** with 1 equiv PMe_3_ shows the formation of **3Ni**^**a**^ with a small amount of **3Ni**^**b**^ (confirmed by ^19^F and ^31^P NMR spectroscopy). Rocamora et al. reported a similar nickel complex *trans*-[(Et_3_P)Ni(κ^2^-2-C_6_Cl_4_PPh_2_)_2_] to **3Ni**^**a**^ as the final product.^17^ Stirring a dichloromethane (DCM) solution of **3Ni**^**a**^ for 4 days at ambient temperature resulted in ring opening and slow formation of **3Ni**^**b**^. The obtained 1:3 mixture of the **3Ni**^**a**^ and **3Ni**^**b**^ does show eight additional signals in the ^19^F and two signals in the ^31^P NMR spectra. Through fractional crystallization, it was possible to separate and characterize the byproduct as [Ni_2_(κ^2^-2-C_6_F_4_PPh_2_)_2_(μ-2-C_6_F_4_PPh_2_)_2_] (**4Ni**). It was only possible to isolate an isomeric mixture of **3Ni**^**a**^ and **3Ni**^**b**^ as a second fraction. Counter-intuitively, the homologous platinum compound [Pt_2_(κ^2^-2-C_6_F_4_PPh_2_)_2_(μ-2-C_6_F_4_PPh_2_)_2_] (**4Pt**) was accessible by quickly treating *trans*-**1Pt** with an excess of PMe_3_ (ca. 2.8 equiv: 11% **4Pt**, ca. 5.5 equiv: 17% **4Pt**, addition of PMe_3_ within 1 s). Compound **4Pd** was not accessible by either of the described synthesis methods used for **4Ni** or **4Pt**. In spite of the formation of [Pt_2_(κ^2^-2-C_6_H_4_PPh_2_)_2_(μ-2-C_6_H_4_PPh_2_)_2_] by refluxing a toluene solution of *cis*-[Pt(κ^2^-2-C_6_H_4_PPh_2_)_2_], similar treatment of *cis*-**1Pt** or *trans*-**1M** in toluene does not form compound **4M**.^18^ The energy difference between *trans*-**1M** and **4M** was calculated to be approximately −20 to −30 kcal mol^–1^ [M = Ni (−22.6 kcal mol^–1^), Pd (−29.3 kcal mol^–1^), Pt (−31.3 kcal mol^–1^)], which leads to the conclusion that PMe_3_ can act as a dimerization promoter. Starting from *trans*-**1Ni** or *cis*-**1Pt** and reacting it with 1 equiv of PMe_3_ resulted in compounds of the type **3M**^**b**^ with *cis*-C-M-C arrangement. The same *cis*-C-M-C configuration is visible in both compounds of the type **4M**. Our quantum chemical calculations reveal that the relative energies of **3M**^**a**^ and **3M**^**b**^ for M = Ni and Pt are similar (**3M**^**b**^ is slightly more stable by <1.6 kcal mol^–1^, respectively). The molecular structures of *syn*-**2M**^**a**^, **3M**^**b**^, and **4M** (M = Ni, Pt) were confirmed by single-crystal X-ray diffraction (Figure 1 and Table S1). A detailed structural description is given in the Supporting Information.

### Complexes of the Type [(Me_3_P)_2_M(μ-2-C_6_F_4_PPh_2_)_2_M′Cl]

**Scheme 5:**
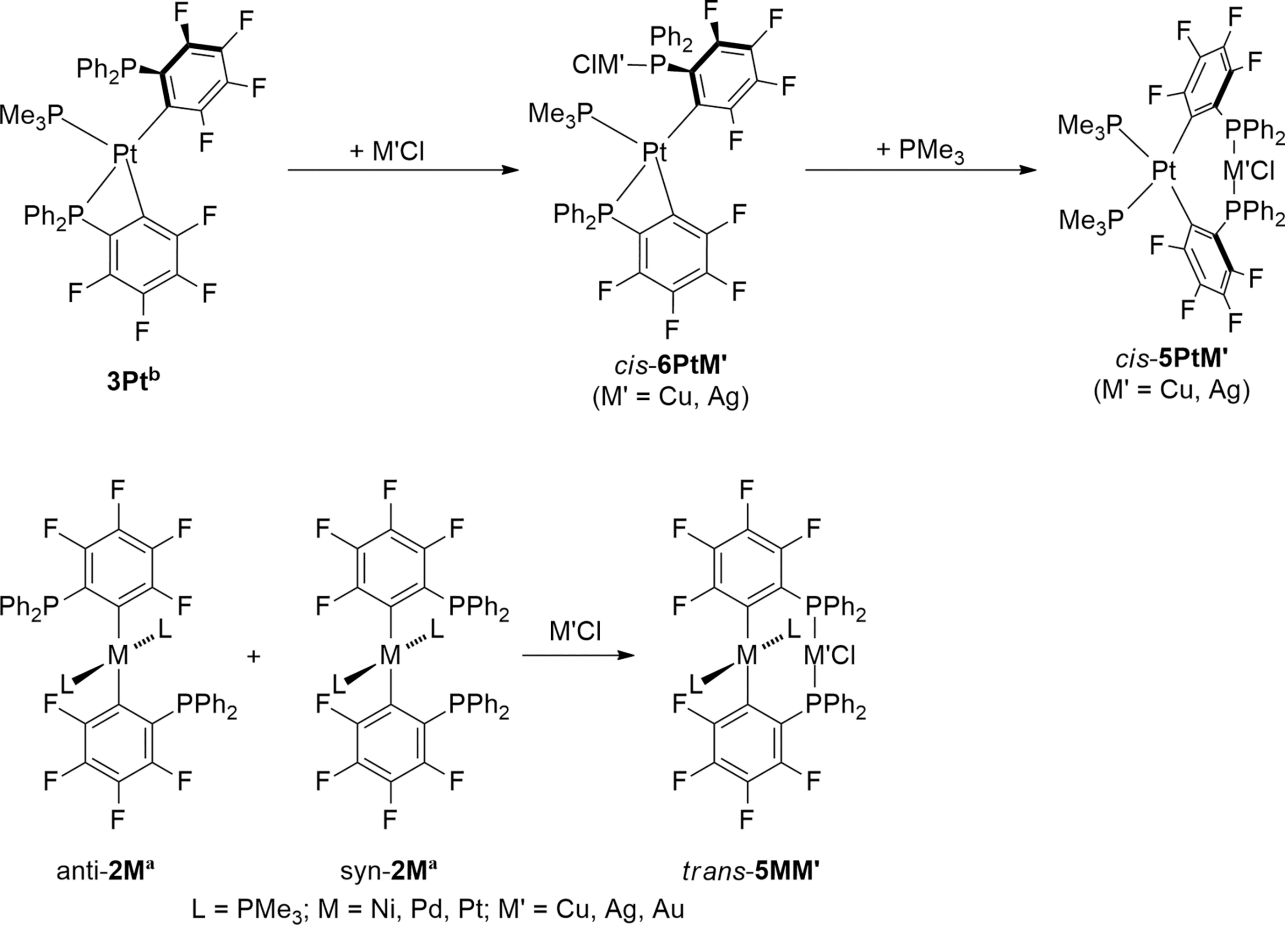
Formation of Heterobimetallic Complexes of the Type [(Me_3_P)_2_M(μ-2-C_6_F_4_PPh_2_)_2_M′Cl]

**Scheme 6:**
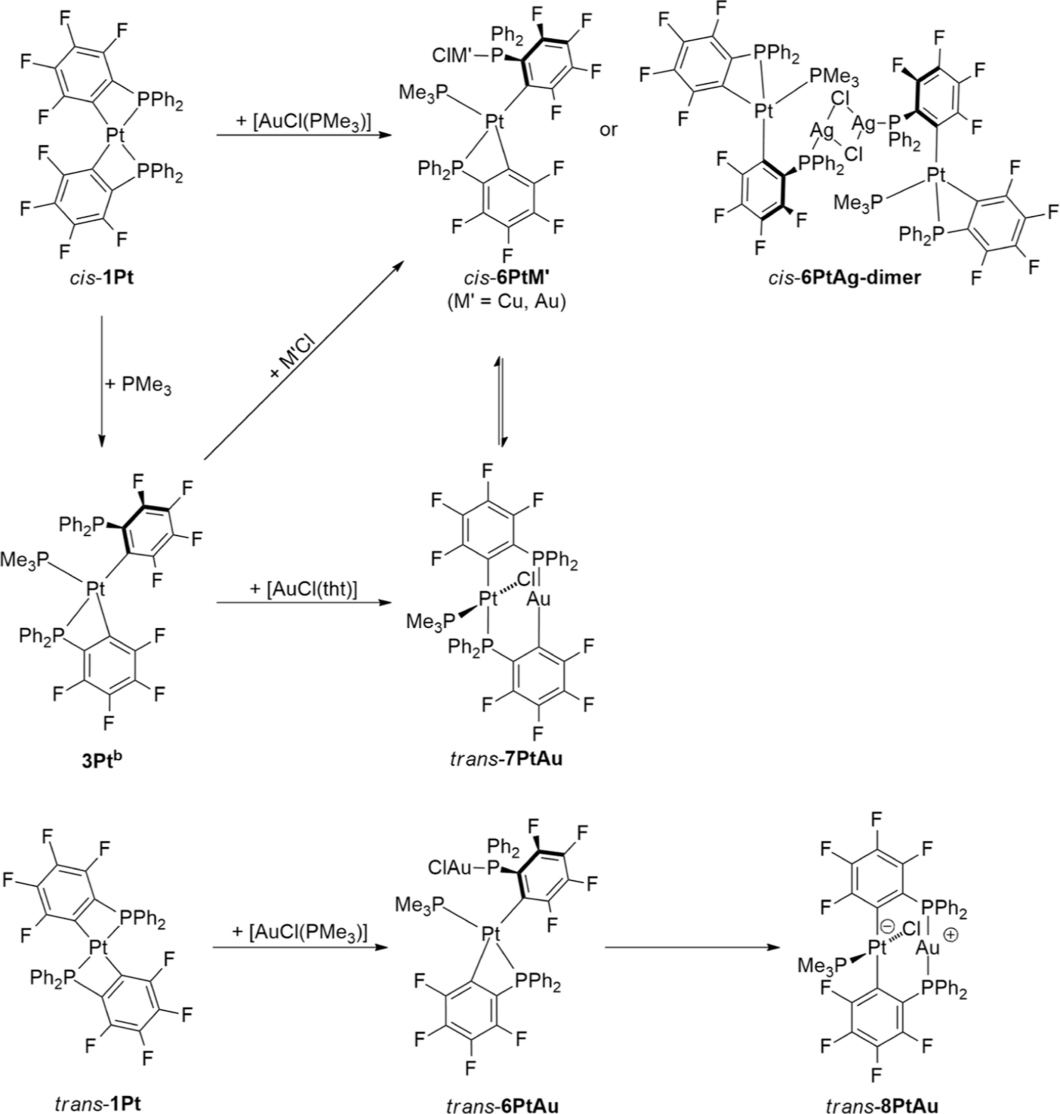
Synthesis of Various Isomers of Compounds of the Composition [(Me_3_P)Pt(2-C_6_F_4_PPh_2_)_2_M′Cl]

Reacting *cis*-**1Pt** with excess PMe_3_ resulted in the formation of *syn/anti*-**2Pt**^**b**^ (Scheme 3), which was impossible to isolate. An in situ reaction of *syn/anti*-**2Pt**^**b**^ with M′Cl (M′ = Cu, Ag, Au(tht); tht = tetrahydrothiophene) was not suitable to form the homologue *cis*-**5PtM′** due to the presence of unreacted PMe_3_. The excess PMe_3_ can easily react with the coinage metal chlorides to form various species of the form [M′Cl(PMe_3_)_*x*_]_*n*_ (*x* = 1–4, *n* = 4–1).^19^ To prevent byproduct formation, the synthesis was started from **3Pt**^**b**^, which was reacted with 1 equiv M′Cl (M′ = Cu, Ag) to form complexes of type *cis*-**6PtM′** (vide infra). The isolated compounds *cis*-**6PtM′** were treated with 1 equiv PMe_3_ in order to obtain complexes of type *cis*-**5PtM′** (Scheme 5). Compound *cis*-**6PtAu** was accessible by reacting *cis*-**1Pt** with [AuCl(PMe_3_)] (Scheme 6).

**Figure 2:**
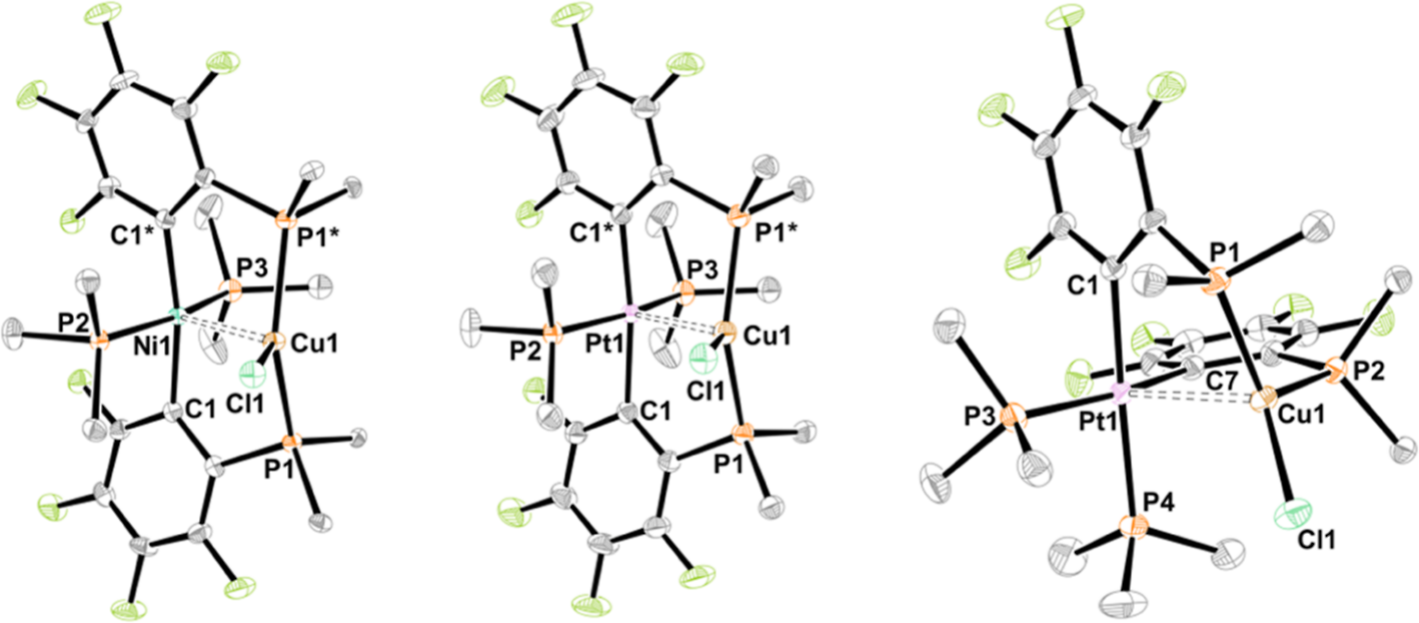
Molecular structures of *trans*-**5NiCu**, *trans*-**5PtCu**, and *cis*-**5PtCu** (left to right). Ellipsoids are shown at 50% probability level. Hydrogen atoms are omitted, and only the *ipso*-carbons of the PPh_2_ groups are depicted for clarity.

Compound *cis*-**5PtCu** was accessible in a pure state by the mentioned synthesis route. Because of difficulties in handling the PMe_3_-toluene solution (air sensitivity and slow oxidation during storage), the addition of exactly 1 equiv PMe_3_ to form complex *cis*-**5PtAg** in a pure state was not possible. Isolated samples of *cis*-**5PtAg** showed impurities of either *cis*-**6PtAg-dimer** by adding small amounts of PMe_3_ or **3Pt**^**b**^ by adding excess PMe_3_ (presumably removing AgCl as [AgCl(PMe_3_)_*x*_]_*n*_ (*x* = 1–4, *n* = 4–1)).^19^ The impurities could not be separated by crystallization. The presence of *cis*-**5PtAg** could be confirmed by ^19^F and ^31^P NMR spectroscopy in CDCl_3_ solution [^19^F: −106.8, −116.4, −150.8, −159.3; ^31^P: 14.3 (^1^*J*_(107/109)Ag,P_ = 380 Hz, 1P, AgPPh_2_), −31.4 (^1^*J*_Pt,P_ = 2140 Hz, 1P, PMe_3_)]. The reaction of *cis*-**6PtAu** with 1 equiv PMe_3_ only led to product mixtures. It was possible to obtain crystals suitable for single-crystal X-ray diffraction of *cis*-**5PtCu** and *cis*-**5PtAg** from dichloromethane/*n*-hexane solution (Figure 2, Table S2).

C*is*-**5PtCu** and *cis*-**5PtAg** show the expected *cis*-configuration of the bridging ligands μ-2-C_6_F_4_PPh_2_. The same mutual *cis*-configuration was found in the corresponding complexes [(dppe)M(μ-2-C_6_F_4_PPh_2_)_2_M′Cl] (M = Pd, M′ = Cu, Ag; M = Pt, M′ = Cu; dppe = 1,2-bis(diphenylphosphino)ethane).^15^ We attribute the significant longer Pt···Cu separation in [(dppe)Pt(μ-2-C_6_F_4_PPh_2_)_2_CuCl] (d(Pt···Cu): 2.9466(3) Å) in comparison to *cis*-**5PtCu** to the steric crowding between the Cu–Cl unit and the phenyl groups of the dppe ligand. The longer Pt···Ag separation is also observed in [(dppe)Pt(μ-2-C_6_F_4_PPh_2_)_2_AgCl] (d(Pt···Ag): 3.0964(4) Å) in comparison to *cis*-**5PtAg**.

Following the reported synthesis strategy of the formation of *trans*-**5PdM′** (M′ = Cu, Ag, Au),^14^ a respective mixture of *anti*/*syn*-**2M**^**a**^ (M = Ni, Pt) was reacted with CuCl, AgCl, or [AuCl(tht)] (tht = tetrahydrothiophen) in dichloromethane to form complexes of the type *trans*-**5MM′** (M = Ni, Pt; M′ = Cu, Ag, Au (Scheme 5). The ^31^P NMR chemical shifts of the 9 complexes of the type *trans*-**5MM′** are given in Table 2.

**Table 2:** ^31^P NMR Chemical Shifts of Complexes of the Type *trans*-**5MM′** (M = Ni, Pd,^14^ Pt; M′ = Cu, Ag, Au) in CDCl_3_

M/M′	Cu		Ag		Au	
	PMe_3_	μ-2-C_6_F_4_PPh_2_	PMe_3_	μ-2-C_6_F_4_PPh_2_	PMe_3_	μ-2-C_6_F_4_PPh_2_
Ni	–14.4	7.2	–15.1	15.9	–15.9	41.8
Pd^a^	–16.6	3.1	–17.7	11.8	–17.7	37.9
Pt	–21.7	–0.9	–24.1	6.4	–26.0	35.2

a Measured in CD_2_Cl_2_.

By changing the coinage metal and keeping the d^8^-metal equal, the chemical shift of the ^31^P NMR signal of the PMe_3_ group is changing in a narrow range (Δδ_P_ = 1.1–4.3 ppm) as expected. In contrast, by changing the d^8^-metal of the nickel triad and keeping the coinage metal equal, the chemical shift of the ^31^P NMR signal of the bridging μ-2-C_6_F_4_PPh_2_ shows a high field shift along the series Ni > Pd > Pt in a range of Δδ_P_ = 8.1 (Cu), 9.5 (Ag), and 6.6 ppm (Au). For the series *trans*-**5MCu** (M = Ni, Pd,^14^ Pt), it was possible to determine all three molecular structures, which are isomorphous (orthorhombic, space group *Pnma*). The molecular structures of *trans*-**5NiCu** (left) and *trans*-**5PtCu** (middle) are shown in Figure 2, and selected interatomic distances and angles are given in Table S2.

The structure overlay (Figure S65) of complexes of the type *trans*-**5MCu** (M = Ni, Pd,^14^ Pt) shows that the three complexes are almost identical (root-mean-square deviation of atomic positions of M1, M′1, Cl1, P1, and P1*: rmsd < 0.04 Å). The M–C1 bond length is increasing in the order Ni < Pd < Pt by approximately 0.13 Å, and the M–P2/3 bond length are increasing in the order Ni < Pt < Pd by approximately 0.17 Å (see Supporting Information for details). The Cu1–P1 bond lengths show a minor elongation by approximately 0.03 Å and the Cu1–Cl1 bond lengths are slightly elongated by less than 0.02 Å, which is in contrast to the observations of the formal large Δδ_P_ in the ^31^P NMR spectra. The M···Cu distances in the complexes of the type *trans*-**5MCu** [M = Ni: 2.82(1); Pd: 2.84(1); Pt: 2.82(1) Å] are similar. The sum of the covalent radii [Ni–Cu: 2.56(6); Pd–Cu: 2.71(8); Pt–Cu: 2.68(8) Å]^20^ in the series are shorter than the observed M–Cu distances by approximately 0.13–0.26 Å. The M···PPh_2_ distances in the series follow the order 3.38(1) (Ni) < 3.43(1) (Pd) < 3.45(1) Å (Pt), which would suggest an increasing diamagnetic shielding contribution of heavier metal atoms in the ^31^P NMR shifts of the PPh_2_ group as observed for PMe_3_. However, in the monometallic complexes of type *syn*-**2M**^**a**^, the M···PPh_2_ distances display a higher deviation [3.33(1) (Ni) < 3.40(1) (Pd) < 3.44(1) Å (Pt)] and are closer to the d^8^-metal with a smaller magnitude in Δδ_P_ in the ^31^P NMR spectra [1.7 ppm (*syn*-**2M**^**a**^) vs 8.1 ppm (*trans*-**5MCu**)]. This would suggest that the diamagnetic shielding contribution of heavier metal atoms only plays a minor role in the ^31^P NMR shifts of the bridging μ-2-C_6_F_4_PPh_2_. Calculation of the ^31^P NMR shift difference of *syn*-**2M**^**a**^ (2.6 ppm) vs *trans*-**5MCu** (6.6 ppm) supports this claim (see Supporting Information for details).

**Figure 3:**
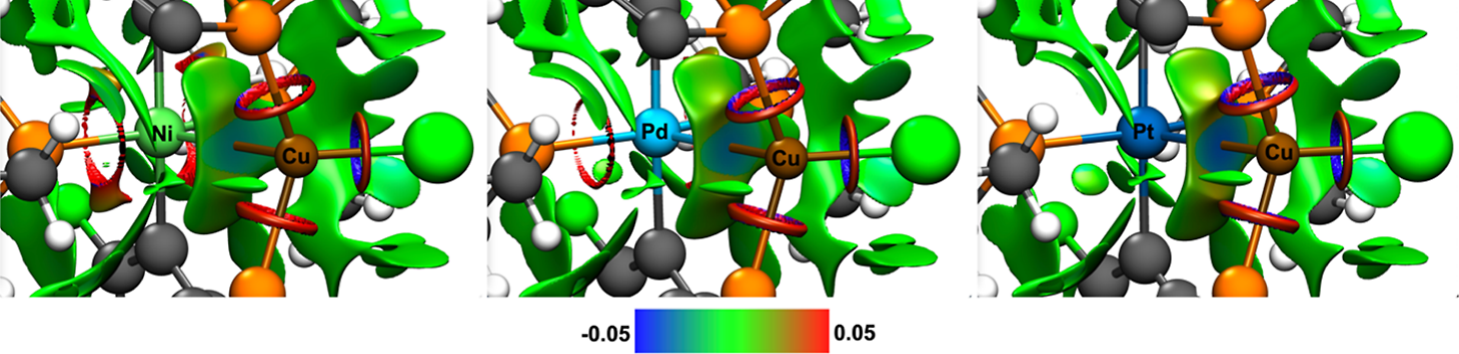
NCI descriptor of *trans*-**5NiCu** (left), *trans*-**5PdCu** (middle), and *trans*-**5PtCu** (right). Isovalue is set to 0.45, and color range is from −0.05 to 0.05 au; color code: blue—attractive interactions; green—van der Waals interactions; red—non-attractive interactions.

Due to the absence of major structural differences among the series of complexes of type *trans*-**5MCu**, we were interested if the electronic structure has an impact on the formal large Δδ_P_ in the ^31^P NMR spectra. The non-covalent interaction (NCI) descriptor^21^ shows clearly an increasing non-covalent attractive interaction along the series Ni < Pd < Pt (Figure 3).

**Figure 4:**
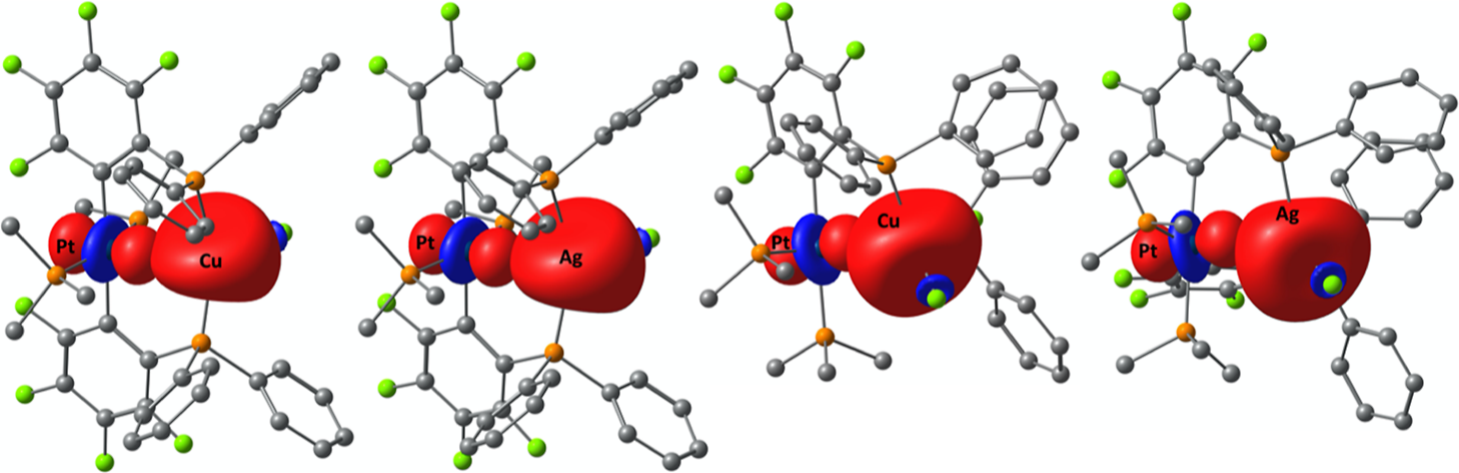
LP(Pt)→LV(M′) main donor–acceptor interaction derived by NBO analyses of *trans*-**5PtCu** (left, M′ = Cu), *trans*-**5PtAg** (middle left, M′ = Ag), *cis*-**5PtCu** (middle right, M′ = Cu), and *cis*-**5PtAg** (right, M′ = Ag). NBOs are displayed with an isosurface value of 0.05.

**Figure 5:**
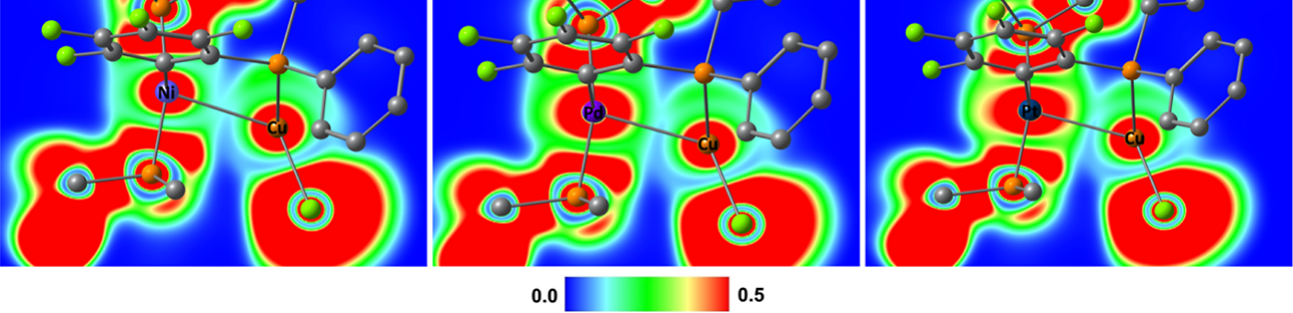
ELF of *trans*-**5NiCu** (left), *trans*-**5PdCu** (middle), and *trans*-**5PtCu** (right). Color range is from ELF = 0.0 to 0.5 au; color code: blue—strong delocalization, red—electron-gas-like pair probability. Hydrogen atoms are omitted for clarity.

Natural localized molecular orbital (NLMO) calculations^22^ of the series *trans*-**5MCu** exhibit a decrease Cu contribution in both Cu–P bonds in the order Ni < Pd < Pt by about 6% in total. In the same order, Cu contributions to the d_z_^2^-orbital of Ni (0.06%), Pd (0.14%), and Pt (0.24%) increase similarly the (main) M→Cu interaction energy (second-order perturbation theory: Ni: 1.12; Pd: 7.67; Pt: 38.73 kcal mol^–1^; Table 3 and Figure 4). In the electron localization function (ELF),^23^ an increase localized electron density is visible in the order Pd (0.121 au) < Ni (0.128 au) < Pt (0.140 au.) (Figure 5), which is especially interesting because of similar covalent radii of Pt (1.36(5) Å) and Pd (1.39(6) Å).^20^ The natural charge (NC) of Cu was found to be 0.67 in all three complexes, whereas for the nickel triad the value decreases in the order Ni (0.42) > Pd (0.26) > Pt (0.23). The possible intermetallic coulombic repulsions between M and M′ therefore drop in intensity along the series. The Wiberg bond order (WBO) supports the increasing M···Cu interaction in the same order [Ni (0.27) < Pd (0.32) < Pt (0.37)].

**Table 3:** NC and NBO Characteristics of the Main Donor–Acceptor Interaction [LP—Valence Lone Pair, LV—Lone Vacant Orbital, NBO Energy Level (*E*_NBO_, au), Donor–Acceptor Energy (kcal mol^–1^)] and WBO of *trans*- and *cis*-**5MM′** (M = Ni, Pd, Pt; M′ = Cu, Ag, Au)

	*trans*-**5NiCu**	*trans*-**5PdCu**	*trans*-**5PtCu**	*trans*-**5PdAg**	*trans*-**5PtAg**	*trans*-**5PdAu**	*trans*-**5PtAu**	*cis*-**5PtCu**	*cis*-**5PtAg**
NC(M)	0.42	0.26	0.23	0.26	0.23	0.27	0.24	0.23	0.23
NC(M′)	0.67	0.67	0.67	0.64	0.65	0.47	0.47	0.71	0.69
*E*_NBO_(LP(M))	–0.26	–0.28	–0.22	–0.27	–0.22	–0.27	–0.21	–0.22	–0.22
LP(M)	100% d	99% d	97% d	99% d	97% d	99% d	97% d	97% d	97% d
*E*_NBO_(LV(M′))	0.09	0.10	0.17	0.35	0.39	0.17	0.20	0.18	0.34
LV(M′)	99% s	99% s	99% s	99% s	99% s	95% s	95% s	99% s	99% s
*E*_M→M′_	1.12	7.67	38.73	7.34	26.38	9.62	30.41	39.86	27.28
WBO	0.27	0.32	0.37	0.32	0.35	0.39	0.39	0.36	0.33

Topological analyses (atoms in molecules, AIM) have been performed to get a better understanding of the covalency of the M···M′ interactions.^24^ The results of the topological parameters of compounds of the type **5MM′** are listed in Table 4. For all complexes of type **5MM′**, critical points with (3, −1) characteristic (bond critical point, bcp) were found along the bond path between M and M′. The definition of a covalent bond “is based on two conditions, namely (i) the existence of a critical point ***r***_**b**_ and its associated maximum electron density path linking the nuclei in question (necessary condition) and (ii) *H*(***r***_**b**_) < 0 which indicates that the accumulation of electron charge in the internuclear region is stabilizing (sufficient condition)”^25^ (*H*(***r***_**b**_) = electron energy density), which is the case for all complexes of type **5MM′**. Dinda and Samuelson gave further classification of the bond characteristic depending on the |*V*(***r***_**b**_)|/*G*(***r***_**b**_) ratio (with *V*(***r***_**b**_) = potential energy density, *G*(***r***_**b**_) = Lagrangian kinetic energy).^12a^ Typical covalent interactions feature |*V*(***r***_**b**_)|/*G*(***r***_**b**_) > 2, whereas ionic character is characterized with |*V*(***r***_**b**_)|/*G*(***r***_**b**_) < 1. For complexes of type **5MM′**, the bcps are in the range 1 < |*V*(***r***_**b**_)|/*G*(***r***_**b**_) < 2, which is characteristic for bonds with intermediate character and does show that the M···M′ interactions are not purely ionic in nature but have some electron-shared (covalent) character. Also, the relatively low electron density ρ(***r***_**b**_) at the bcps together with a positive Laplacian of electron density (∇^2^ρ(***r***_**b**_) > 0) and similar modulus of Lagrangian kinetic energy density and potential energy density (*G*(***r***_**b**_) ≙ |*V*(***r***_**b**_)|) are in support of the presence of a closed shell bonding with donor–acceptor characteristics.^26,27^ The electron density at the bcp ρ(***r***_**b**_) in the series *trans*-**5MCu** does increase in the expected order (M = Ni < Pd < Pt), which is in good agreement with the observations of ELF (Figure 5).

**Table 4:** Results of the Theoretical Topological Analysis of the Bond Critical Points (3, −1) between M and M′ in Complexes *trans*- and *cis*-**5MM′** (M = Ni, Pd, Pt; M′ = Cu, Ag, Au)^a^

	*trans*-**5NiCu**	*trans*-**5PdCu**	*trans*-**5PtCu**	*trans*-**5PdAg**	*trans*-**5PtAg**	*trans*-**5PdAu**	*trans*-**5PtAu**	*cis*-**5PtCu**	*cis*-**5PtAg**
ρ(***r***_**b**)_	0.02208	0.02620	0.03132	0.02879	0.03348	0.03607	0.04117	0.03090	0.03178
∇^2^ρ(***r***_**b**_)	0.03699	0.05862	0.07437	0.07135	0.08161	0.08916	0.09973	0.07583	0.07717
*G*(***r***_**b**_)	0.01299	0.01785	0.02215	0.02193	0.02516	0.02773	0.03093	0.02235	0.02358
*V*(***r***_**b**_)	–0.01672	–0.02105	–0.02572	–0.02601	–0.02994	–0.03319	–0.03698	–0.02575	–0.02788
|*V*(***r***_**b**_**)**|/*G*(***r***_**b**_)	1.288	1.179	1.161	1.186	1.190	1.197	1.196	1.152	1.182
*G*(***r***_**b**_)/ρ(***r***_**b**_)	0.588	0.681	0.707	0.762	0.752	0.769	0.751	0.723	0.742
*H*(***r***_**b**_)	–0.00374	–0.00320	–0.00357	–0.00409	–0.00478	–0.00546	–0.00605	–0.00340	–0.00430
sign(λ_2_(***r***_**b**_))ρ(***r***_**b**_)	–0.022	–0.026	–0.031	–0.029	–0.033	–0.036	–0.041	–0.031	–0.032

a Electron density (ρ(***r***_**b**_) in au), Laplacian of electron density (∇^2^ρ(***r***_**b**_) in au), Lagrangian kinetic energy density (*G*(***r***_**b**_) in au), potential energy density (*V*(***r***_**b**_) in au), ratio |*V*(***r***_**b**_)|/*G*(***r***_**b**_), ratio *G*(***r***_**b**_)/ρ(***r***_**b**_) in au, electron energy density (*H*(***r***_**b**_) in au), the product of sign of second largest eigenvalue of Hessian matrix of electron density (λ_2_(***r***_**b**_)), and ρ(***r***_**b**_) in au.

Closed shell interactions were previously categorized by Macchi et al. as metallic (shared) bonding and donor–acceptor interactions.^27^ We will use these terms as described in the literature. To differentiate between metallic (shared) bonding behavior and donor–acceptor interactions, Macchi et al. suggested for heavy metal interactions that the *G*(***r***_**b**_)/ρ(***r***_**b**_) ratio can be used as a classification criterion (*G*(***r***_**b**_)/ρ(***r***_**b**_) < 1: metallic (shared) bonding vs *G*(***r***_**b**_)/ρ(***r***_**b**_) ∼ 1: closed shell donor–acceptor interaction).^27^ In the series *trans*-**5MCu**, the magnitude of *G*(***r***_**b**_)/ρ(***r***_**b**_) is in the expected range and does increase (closer to 1) in the order *trans*-**5NiCu** < *trans*-**5PdCu** < *trans*-**5PtCu**. The increase closed shell donor–acceptor interaction down the nickel triad is supported by NBO analysis. Besides the main donor–acceptor interactions listed in Table 3, a detailed list of the second-order perturbation theory between M and M′ is given in the Supporting Information. The Σ[LP(M)→LV(Cu)] interactions are M = Ni: 1.23, Pd: 8.19, and Pt: 39.76 kcal mol^–1^ (see Supporting Information for details), whereas the Σ[BD*(M–C/P)←LP(Cu)] interactions are M = Ni: 1.06, Pd: 4.13, and Pt: 10.27 kcal mol^–1^. This proves the better donor ability of Pt toward Cu over Ni and Pd, as well as that Cu is a weaker (or similar) donor toward the d^8^-metal. Within the respective series *trans*-**5PdM′** or *trans*-**5PtM′**, the intensity of the metal–metal interactions is increasing in the order Cu < Ag < Au (WBO, NC, AIM). However, the sum of M→M′ donor–acceptor interaction intensity appears to follow the trend Ag ≈ Cu < Au (NBO) for M = Pd and Ag < Au < Cu (NBO) for M = Pt (see the Supporting Information for details). This change in trend is originated in the differences of how the metal–metal interactions are analyzed. The AIM part is electron density based and describes the situation in whole, whereas with NBO only the orbital part of the interaction can be extracted.

Due to a lack in structural difference, we assume that the ^31^P NMR shift of the phosphorous atom of the bridging ligand μ-2-C_6_F_4_PPh_2_ toward higher field (Table 2) is originated in electronic differences within the complex series caused by metal–metal interactions. For the series *trans*-**5MAg** and *trans*-**5MAu** (M = Ni, Pd, Pt), the same high field shift of the ^31^P NMR resonance of the bridging ligand μ-2-C_6_F_4_PPh_2_ down the nickel triad was detected. The trends found by AIM and NBO calculations (M = Pd, Pt; M′ = Ag, Au) are in accord with the results found for the series *trans*-**5MCu** (M = Ni, Pd, Pt). Within the respective series *trans*-**5MCu**, *trans*-**5MAg**, and *trans*-**5MAu**, increasing metal–metal interaction energies correlate with a high field shift at the ^31^P nuclei of the bridging ligands μ-2-C_6_F_4_PPh_2_ in the order Ni < Pd < Pt.

Compound *trans*-**5PdAu** shows similar BD*(Pd–C/P)←LP(Au) (10.17 kcal mol^–1^) and LP(Pd)→LV(Au) interactions (10.62 kcal mol^–1^). Therefore, the main character of the interaction should be described as predominantly metallic (shared) bonding. The complex *cis*-**5PtCu** and *cis*-**5PtAg** show similar metal–metal interactions as observed in the corresponding compounds *trans*-**5PtCu** and *trans*-**5PtAg**, respectively. The magnitude of |*V*(***r***_**b**_)|/*G*(***r***_**b**_) is slightly lower for *cis*-**5PtM′** as found in the respective *trans*-**5PtM′** complex, which is indicative of a more pronounced ionic contribution in the *cis* configuration over *trans*. This is reflected in a higher NC at Cu over Ag in complexes with *cis* arrangement over *trans* leading to a higher coulomb repulsion. In conclusion, the metallophilic interactions can be described as predominantly metallic (shared) bonding (Ni–Cu, Pd–Au) with additional donor–acceptor bonding characteristic (Pd→Cu, Pd→Ag, Pt→Cu, Pt→Ag, Pt→Au).

### Complexes of the Type [(Me_3_P)M(2-C_6_F_4_PPh_2_)_2_M′Cl]

As described above, *trans*-**1Ni** and *cis*-**1Pt** show the ability to form complexes of the form **3M**^**b**^ (M = Ni, Pt), where one 2-C_6_F_4_PPh_2_ ligand remains chelating and the other is dangling. Treatment of **3Pt**^**b**^ with 1 equiv CuCl or AgCl gave the expected complex *cis*-**6PtCu** and *cis*-**6PtAg** (Scheme 6), that proves the ability of the dangling PPh_2_ unit to coordinate a coinage metal chloride.

The ^31^P NMR spectra of both complexes show the expected three equally intense resonances, corresponding to the three inequivalent phosphorus nuclei. In both complexes, a respective signal appears at ca. −30 ppm, flanked by ^195^Pt satellites of ca. 2200 Hz, and can be assigned to the PMe_3_ ligand. The signals at −60 ppm, flanked by ^195^Pt satellites of ca. 1600 Hz, can be assigned to the chelating κ^2^-2-C_6_F_4_PPh_2_ ligand. The third resonance appears at 16.5 ppm for *cis*-**6PtCu** (flanked by ^195^Pt satellites of ca. 340 Hz) and at 21.6 ppm for *cis*-**6PtAg** (split into a doublet of doublets; 600 Hz due to coupling with ^107^Ag and 690 Hz due to coupling with ^109^Ag, flanked by ^195^Pt satellites of ca. 395 Hz) can be assigned to the bridging μ-2-C_6_F_4_PPh_2_ ligand. In both cases, the ^19^F NMR spectra show eight equally intense signals, confirming two different 2-C_6_F_4_PPh_2_ ligand environments. The spectroscopic results show the same pattern as observed for the starting material **3Pt**^**b**^ (−6.8, ^3^*J*_Pt,P_ = 207 Hz, κC-PPh_2_; −29.3, ^1^*J*_Pt,P_ = 2350 Hz, PMe_3_; −59.9, ^1^*J*_Pt,P_ = 1540 Hz, κ^2^-PPh_2_), supporting that the reaction only takes place at the dangling κC-2-C_6_F_4_PPh_2_ ligand and no isomerization takes place.

**Figure 6:**
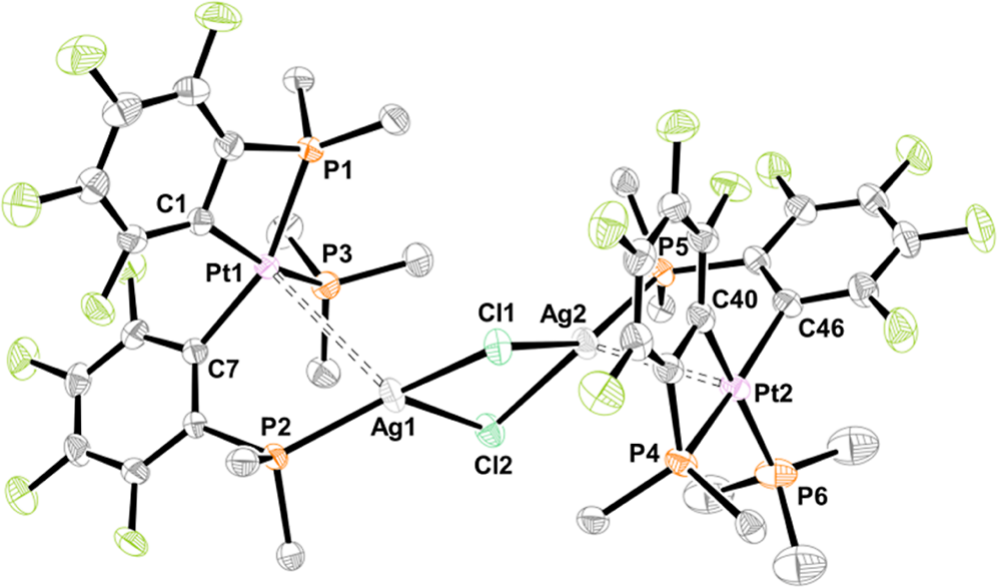
Molecular structures of *cis*-**6PtAg-dimer**. Ellipsoids are shown at a 50% probability level. Hydrogen atoms are omitted, and only the *ipso*-carbons of the PPh_2_ groups are depicted for clarity.

The molecular structure was confirmed by single-crystal X-ray diffraction analysis for *cis*-**6PtCu** and *cis*-**6PtAg** (Figure 6 and Table S3; detailed structure description is given in the Supporting Information). The homologue gold compound *cis*-**6PtAu** was accessible from the reaction of *cis*-**1Pt** with 1 equiv [AuCl(PMe_3_)], in a similar manner to its PPh_3_ analogue *cis*-[(Ph_3_P)Pt(κ^2^-2-C_6_F_4_PPh_2_)(μ-2-C_6_F_4_PPh_2_)AuCl].^18b^ The ^31^P NMR spectrum in C_6_D_6_ of *cis*-**6PtAu** reveals the expected three equally intense resonances at 37.6, −31.9 (^195^Pt satellites of 2285 Hz), and −62.6 ppm (^195^Pt satellites of 1700 Hz), corresponding to three inequivalent phosphorus nuclei. After leaving *cis*-**6PtAu** in solution for several hours, a new set of signals appear at 33.9 (^195^Pt satellites of 205 Hz), 12.5 (^195^Pt satellites of 2285 Hz), and −35.5 ppm (^2^*J*_P,P_ = 16 Hz, ^195^Pt satellites of 3655 Hz). The signal at 33.9 ppm can be assigned to the bridging ligand μ-2-C_6_F_4_PPh_2_ with a P–Au functionality, and the signal at −35.5 ppm to the PMe_3_ ligand bonded at the Pt center. The third signal is low field shifted by about 74 ppm, indicating a bridging functionality of the μ-2-C_6_F_4_PPh_2_ ligand, with the Pt–P bond remaining intact. The same signal pattern can be observed after the reaction of **3Pt**^**b**^ with 1 equiv [AuCl(tht)] and can be assigned to compound *trans*-**7PtAu** (Scheme 6). The bridging μ-2-C_6_F_4_PPh_2_ ligands do show a head-to-tail arrangement at both metal centers with a *trans* orientation about the Pt atom. Complex *trans*-**7PtAu** also undergoes isomerization to *cis*-**6PtAu** in solution, indicating the presence of an equilibrium between both isomers. This observation stands in contrast to the behavior of *cis*-[(Ph_3_P)Pt(κ^2^-2-C_6_F_4_PPh_2_)(μ-2-C_6_F_4_PPh_2_)AuCl], which did not show any isomerization product even after stirring the solution for 5 days.^18b^ This can be explained by PMe_3_ being less sterically demanding as PPh_3_, which appears to favor isomerization.

**Figure 7:**
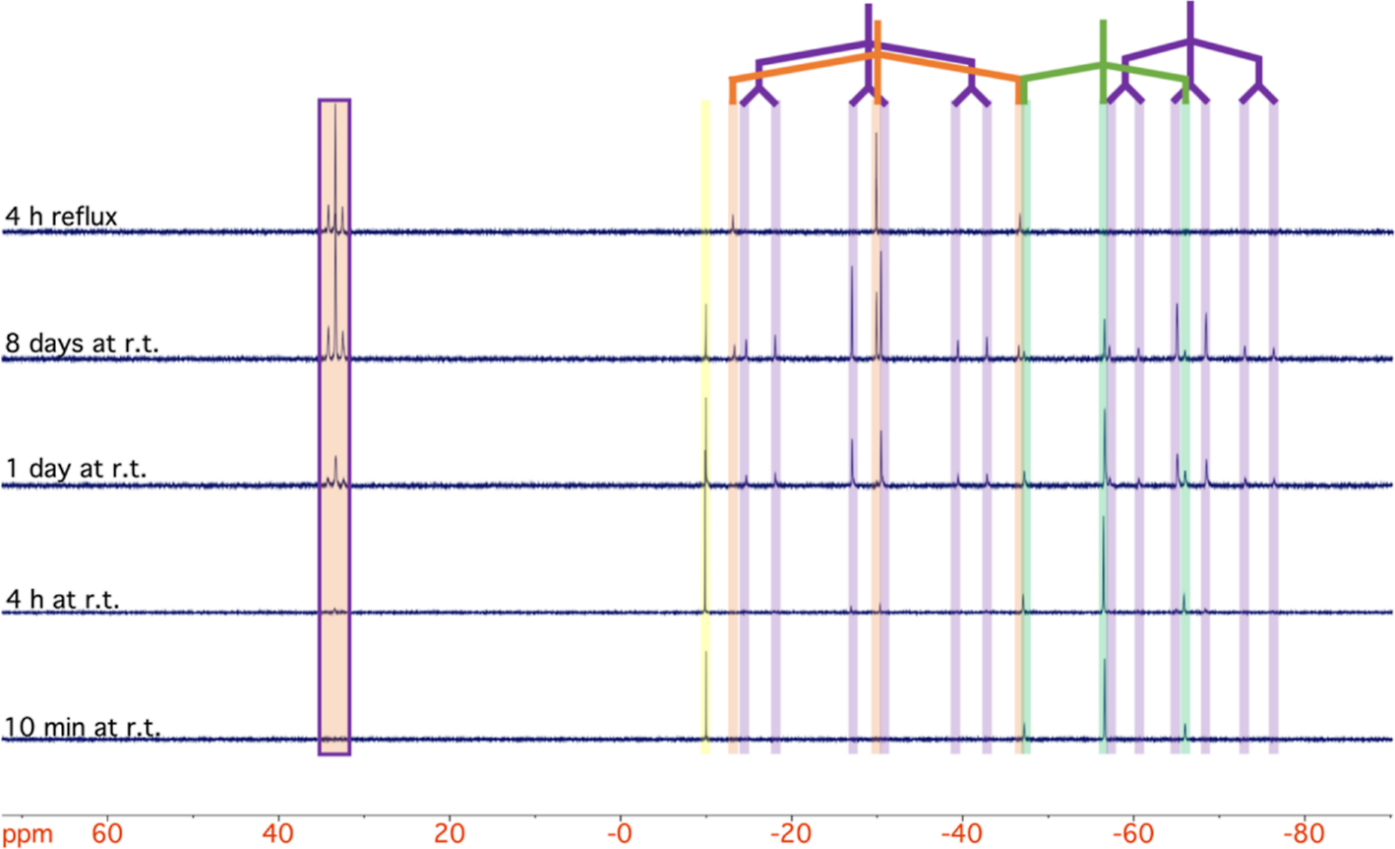
^31^P NMR spectra of the reaction mixture of *trans*-**1Pt** with [AuCl(PMe_3_)] in DCM (from bottom to top: 10 min, 4 h, 1 day, and 8 days at room temperature and fresh prepared reaction mixture refluxed for 4 h). Signal assignments have been highlighted: green (*trans*-**1Pt**: −56.58 ppm (^1^*J*_Pt,P_ = 2280 Hz)), yellow ([AuCl(PMe_3_)]: 9.97 ppm), purple (*trans*-**6PtAu**: 33.35 ppm (^3^*J*_Pt,P_ = 230 Hz), −28.75 ppm (^2^*J*_P,P_ = 410 Hz, ^1^*J*_Pt,P_ = 3010 Hz), −66.76 ppm (^2^*J*_P,P_ = 410 Hz, ^1^*J*_Pt,P_ = 1920 Hz)), and orange (*trans*-**8PtAu**: 33.35 ppm (^1^*J*_Pt,P_ = 200 Hz), −29.93 (^1^*J*_Pt,P_ = 4080 Hz)).

**Figure 8:**
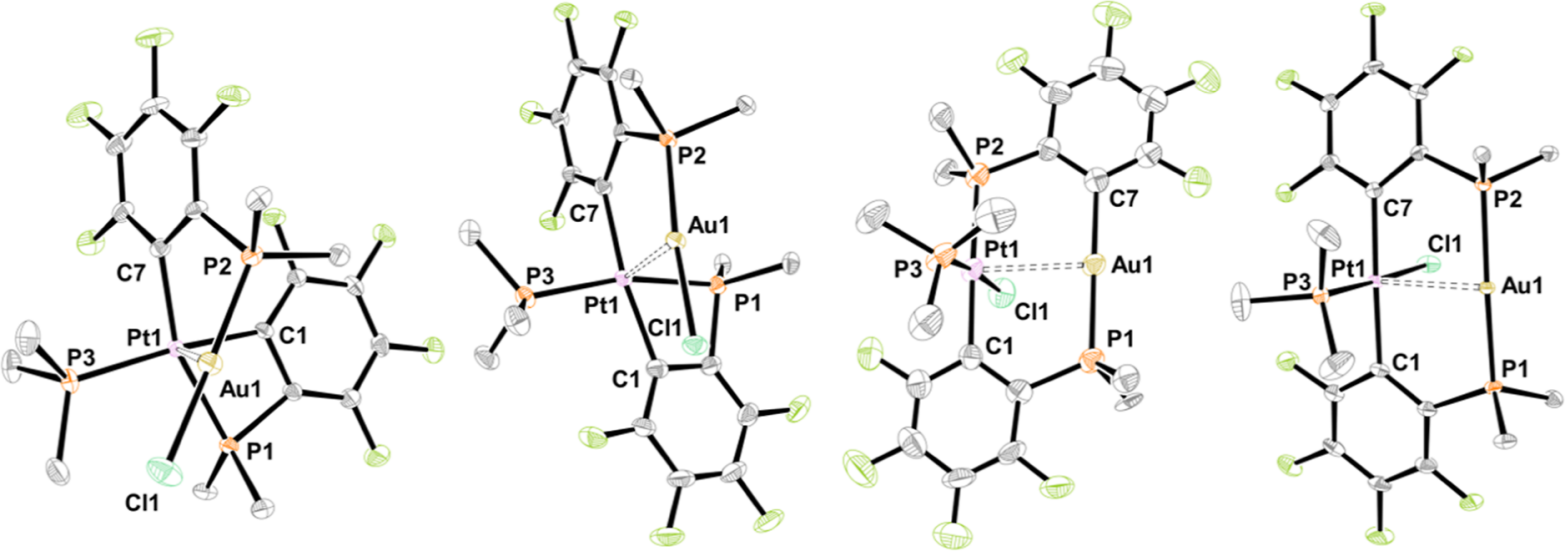
Molecular structures of *cis*-**6PtAu**, *trans*-**6PtAu**, *trans*-**7PtAu**, and *trans*-**8PtAu** (from left to right). Ellipsoids are shown at 50% probability level. Hydrogen atoms and solvent molecules are omitted, and only the *ipso*-carbons of the PPh_2_ groups are depicted for clarity. Selected interatomic separations (Å) and angles (deg) of *cis*-**6PtAu** and *trans*-**6PtAu** are given in Table S3: *trans*-**7PtAu**: Pt1–Au1 2.848(1), Pt1–P2 2.333(4), Pt1–C1 2.05(1), Pt1–Cl1 2.369(3), Pt1–P3 2.236(3), Au1–P1 2.279(4), Au1–C7 2.07(1), P2–Pt1–C1 172.6(4), P3–Pt1–Cl1 173.7(1), P1–Au1–C7 178.0(4); *trans*-**8PtAu**: Pt1–Au1 2.8008(2), Pt1–C1 2.074(3), Pt1–C7 2.065(3), Pt1–Cl1 2.3890(7), Pt1–P3 2.2060(7), Au1–P1 2.2996(7), Au1–P2 2.3044(7), C1–Pt1–C7 173.42(10), P3–Pt1–Cl1 177.94(3), P1–Au1–P2 174.16(2).

Also, upon mixing *trans*-**1Pt** with [AuCl(PPh_3_)], no reaction was observed.^18b^ In contrast, *trans*-**1Pt** with 1 equiv [AuCl(PMe_3_)] in CH_2_Cl_2_ does show slow reaction to *trans*-**6PtAu**, which immediately reacts further to *trans*-**8PtAu**. The reaction was followed by ^31^P NMR spectroscopy. The ^31^P NMR spectrum of the crude reaction mixture (Figure 7) shows the characteristic signals of the starting materials ([AuCl(PMe_3_)], *trans*-**1Pt**) besides the signal sets for *trans*-**6PtAu** and *trans*-**8PtAu**. By addition of *n*-hexane to the crude reaction mixture, it was possible to grow crystals of *trans*-**6PtAu** and *trans*-**8PtAu** suitable for single-crystal X-ray diffraction (vide infra). A pure sample of *trans*-**8PtAu** was accessible by refluxing a mixture of *trans*-**1Pt** with 1 equiv [AuCl(PMe_3_)] in CH_2_Cl_2_ for 4 days. The formation of *trans*-**8PtAu** from the reaction of *trans*-**1Pt** with 1 equiv [AuCl(PMe_3_)] is in agreement with the reaction behavior found for the palladium homologue; however, an intermediate species *trans*-**6PdAu** was not observed.^14^ It was possible to determine molecular structures using single-crystal X-ray diffraction analysis for all four isomers *cis*-**6PtAu**, *trans*-**6PtAu**, *trans*-**7PtAu**, and *trans*-**8PtAu** (Figure 8 and Table S3; detailed structure description is given in the Supporting Information).

**Figure 9:**
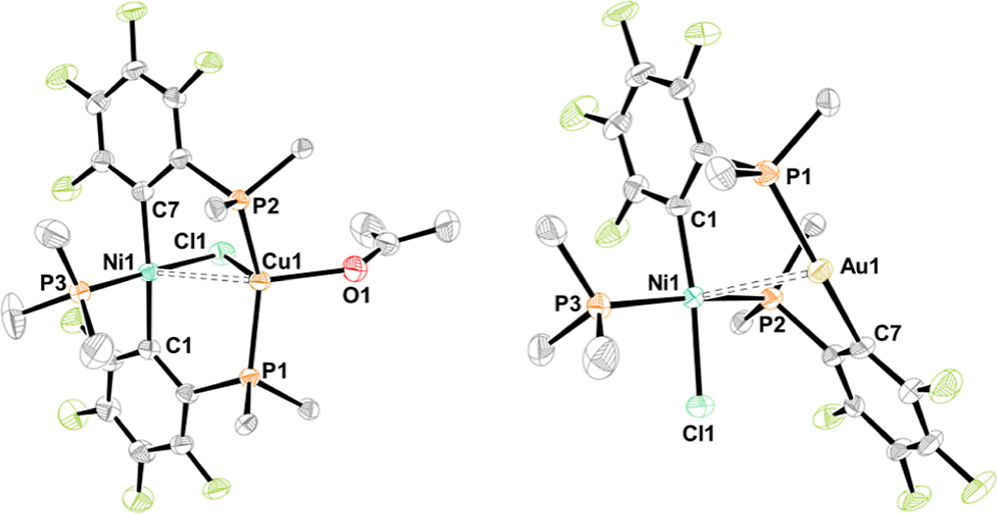
Molecular structures of *trans*-**8NiCu** (left) and *cis*–*trans*-**7NiAu** (right). Ellipsoids are shown at 50% probability level. Hydrogen atoms and solvent molecules are omitted, and only the *ipso*-carbons of the PPh_2_ groups are depicted for clarity. Selected interatomic separations (Å) and angles (deg): *trans*-**8NiCu**: Ni1–Cu1 2.7124(3), Ni1–C1 1.939(2), Ni1–C7 1.925(2), Ni1–Cl1 2.2609(6), Ni1–P3 2.1420(6), Cu1–P1 2.2582(5), Cu1–P2 2.2593(5), Cu1–Cl1 2.4589(5), Cu1–O1 2.200(2), C1–Ni1–C7 172.12(8), Cl1–Ni1–P3 176.27(2), P1–Cu1–P2 143.26(2), O1–Ni1–Cl1 107.53(5), Ni1–Cl1–Cu1 70.01(2); *cis*–*trans*-**7NiAu**: Ni1–Au1 2.853(1), Ni1–C1 1.895(8), Ni1–Cl1 2.204(2), Ni1–P2 2.258(2), Ni1–P3 2.211(2), Au1–P1 2.277(2), Au1–C7 2.070(8), C1–Ni1–Cl1 173.9(3), P2–Ni1–P3 167.7(1), P1–Au1–C7 168.0(3).

**Scheme 7:**
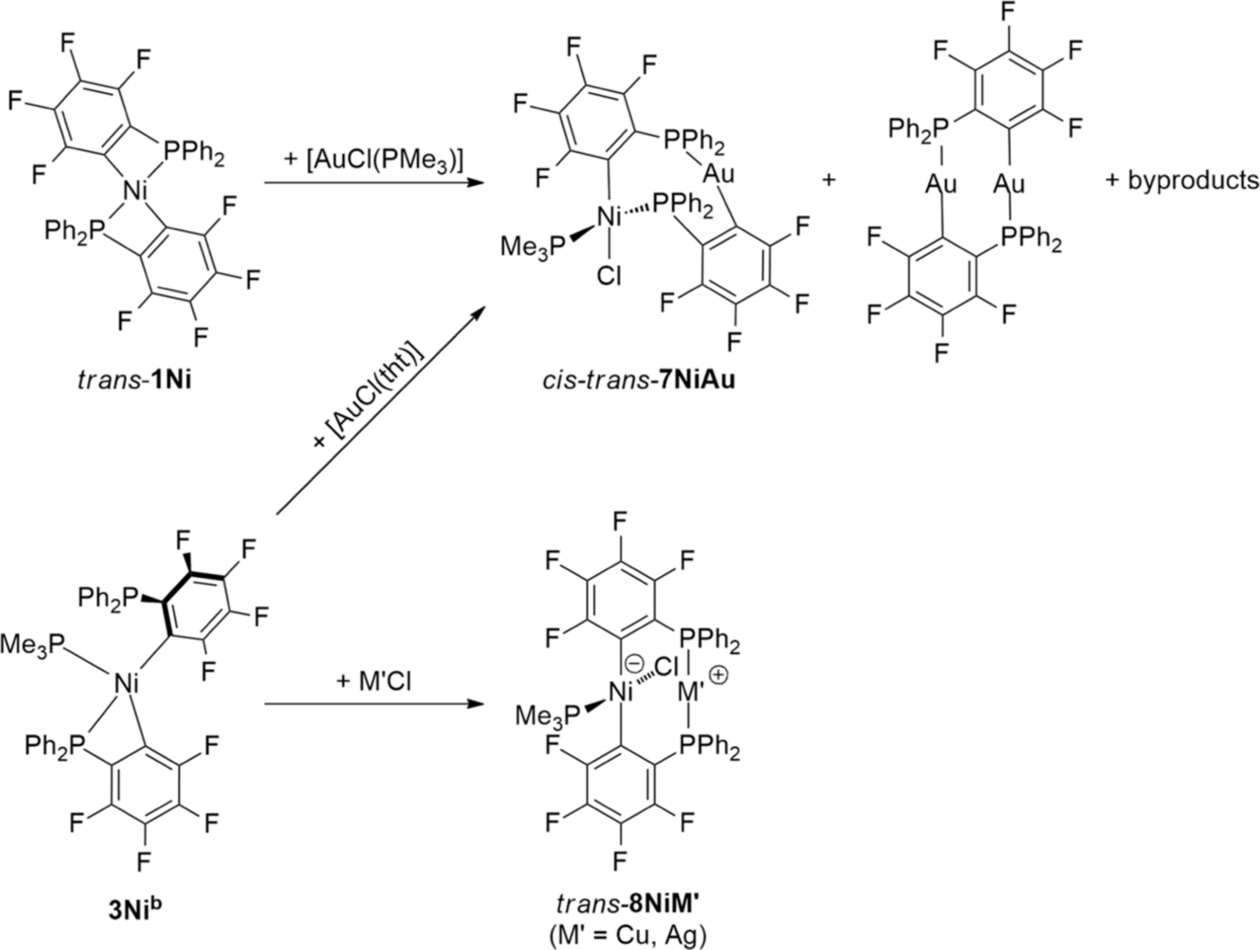
Synthesis of Compounds of the Composition [(Me_3_P)NiCl(2-C_6_F_4_PPh_2_)_2_M′]

The ^31^P NMR spectra of the corresponding reactions of **3Ni**^**b**^ with either CuCl or AgCl show two signals in a 2:1 ratio (Cu: 4.2, −4.7 ppm; Ag: 14.6 (^1^*J*_(109)AgP_ = 566 Hz, ^1^*J*_(107)AgP_ = 490 Hz), −6.1 ppm). For both complexes, the ^19^F NMR spectra show four equally intense signals, which indicates a symmetric μ-2-C_6_F_4_PPh_2_ environment. Therefore, the reaction led to the formation of the nickel homologous *trans*-**8NiM′** (M′ = Cu, Ag, Scheme 7), which stands in contrast to the reaction behavior of **3Pt**^**b**^ with either CuCl or AgCl. The ^31^P NMR spectra of the reaction of **3Ni**^**b**^ with [AuCl(tht)] or *trans*-**1Ni** with [AuCl(PMe_3_)] show three signals at 45.3 (AuPPh_2_), 21.2 (^2^*J*_P,P_ = 330 Hz, NiPPh_2_), and −14.8 ppm (^2^*J*_P,P_ = 330 Hz, NiPMe_3_) together with the characteristic signal of gold dimer (42 ppm)^8c^ and some additional unknown impurities. The ratio of the main product to the gold dimer does increase from 2:1 in the reaction of *trans*-**1Ni** with [AuCl(PMe_3_)] to 10:1 in the reaction of **3Ni**^**b**^ with [AuCl(tht)] in favor of the desired Ni–Au complex. The ^19^F NMR spectrum of the isolated complex clearly reveals two unsymmetrical 2-C_6_F_4_PPh_2_ ligands (−106.3, −116.4, −121.0, −129.9, −152.8, −153.0, −159.4, −160.1 ppm). The ^2^*J*_P,P_ coupling constant of 330 Hz stays in good agreement with the *trans* Me_3_P–Ni-PPh_2_ arrangement, indicating the main complex to be *cis*–*trans*-**7NiAu**, which was separated from the byproducts by fractional crystallization. It was possible to analyze the molecular structure of *trans*-**8NiCu**·acetone and *cis*–*trans*-**7NiAu** with single-crystal X-ray diffraction (Figure 9; detailed structure description is given in the Supporting Information).

**Chart 2:**
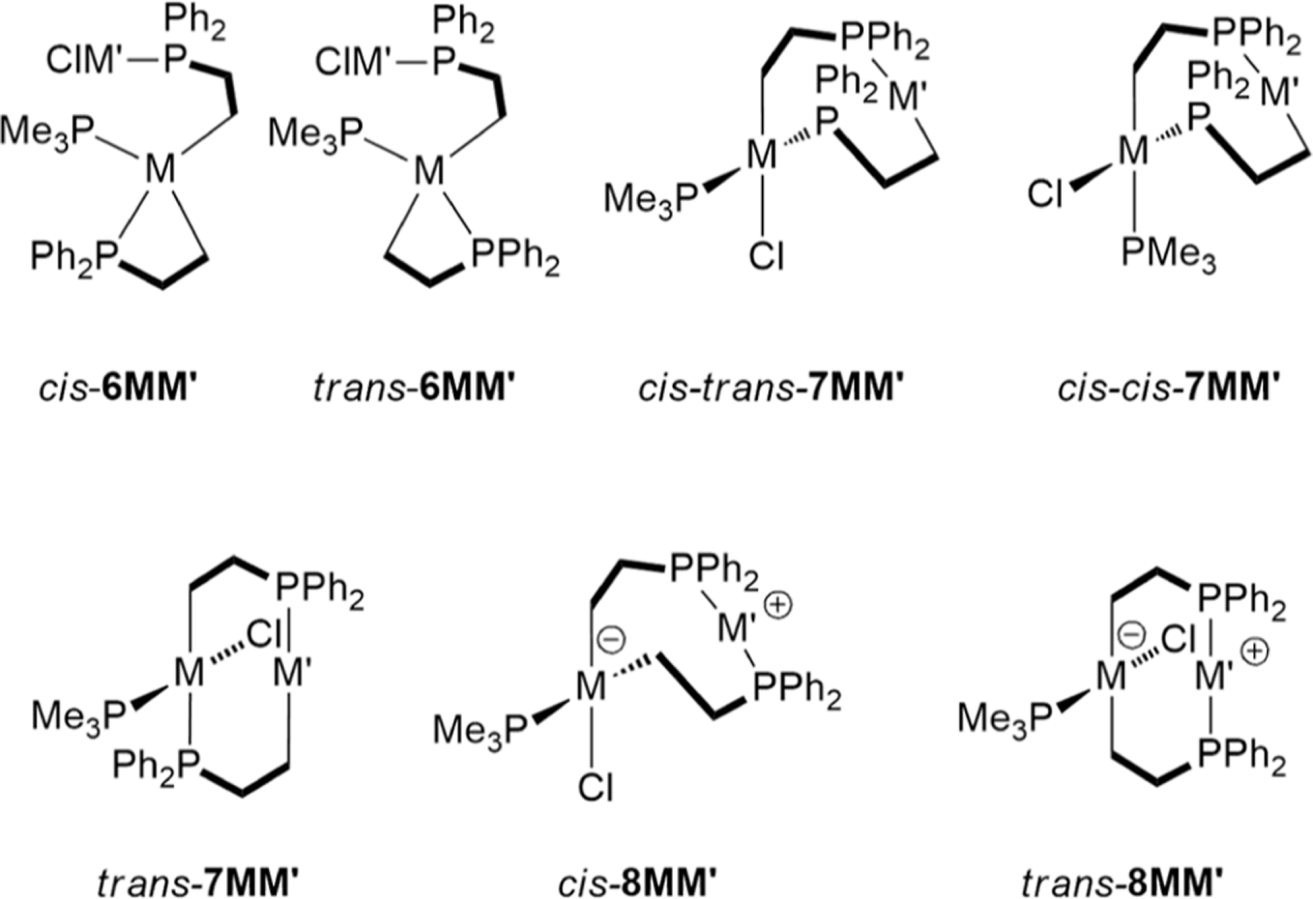
Isomers of Compounds of the Composition [(Me_3_P)M(2-C_6_F_4_PPh_2_)_2_M′Cl]

Quantum chemical calculations were carried out to shed some light into the relative energy levels of the seven possible monomeric isomers of complexes of the type **6**, **7**, and **8** (Chart 2 and Table 5). For M = Ni, the isomers *trans*-**8NiCu**, *trans*-**8NiAg**, and *cis*–*trans*-**7NiAu** were experimentally observed and calculated to be thermodynamically stable isomers. For M′ = Cu and Ag, isomers **6** and *cis*–*trans*-**7NiM′** were predicted to have similar energy levels in comparison to the experimentally observed isomer *trans*-**8NiM′**. For M = Pt and M′ = Cu or Ag, the isomers *cis*-**6PtM′** and *trans*-**6PtM′** revealed similar energy levels, with the former being lower. The monomeric or dimeric form of *cis*-**6PtM′** were experimentally accessible. The respective experimentally observed interconversion of *cis*-**6PtAu** and *trans*-**7PtAu** stayed slightly in contrast with the higher energy level of the latter (Δ*E* = 4.81 kcal mol^–1^). The energy level of *trans*-**8PtAu** against *trans*-**6PtAu** was by about 3.97 kcal mol^–1^ and stayed in contrast to the experimentally observed isomerization of *trans*-**6PtAu** into *trans*-**8PtAu**. Nonetheless, the magnitude of energy difference is in the range that both isomers can coexist in solution. Alternatively, more complex scenarios (e.g., by coordination-dissociation effects of discrete solvent molecules or dimerization with the μ-Cl bridging mode) might also play reasonable roles and could potentially further lower the energy levels of the experimentally observed isomers. The isomer *cis*–*trans*-**7MAu** was predicted to be the thermodynamically most stable isomer for M = Ni, Pd,^14^ and Pt. However, attempts to isolate the complex *cis*–*trans*-**7PtAu** by refluxing a toluene solution of *cis*-**6PtAu** or *trans*-**7PtAu** failed because of decomposition (Figure S66).

**Table 5:** Energy Difference (Δ*E*, kcal mol^–1^) between the Coordination Isomers of Complexes of the Type **6MM′**, **7MM′**, and **8MM′** (M = Ni, Pt; M′ = Cu, Ag, Au)^a^

M	M′	*cis*-**6MM′**	*trans*-**6MM′**	*cis*–*trans*-**7MM′**	*cis-cis*-**7MM′**	*trans*-**7MM′**	*cis*-**8MM′**	*trans*-**8MM′**
Ni	Cu	2.17	2.04	2.11	5.97	6.89	12.03	***0*.*00***
Pt		***0*.*00***	2.32	6.64	7.79	11.79	14.83	11.09
Ni	Ag	0.37	0.00	1.89	6.22	7.43	11.75	1.53
Pt		0.00	2.00	8.08	9.92	13.23	16.89	10.76
Ni	Au	8.05	8.51	***0*.*00***	4.60	6.57	15.06	2.58
Pt		***1*.*17***	***3*.*37***	0.00	2.28	***5*.*99***	13.44	***7*.*34***

a Energy values of the crystallographically characterized isomers are written in italics and bold style.

**Figure 10:**
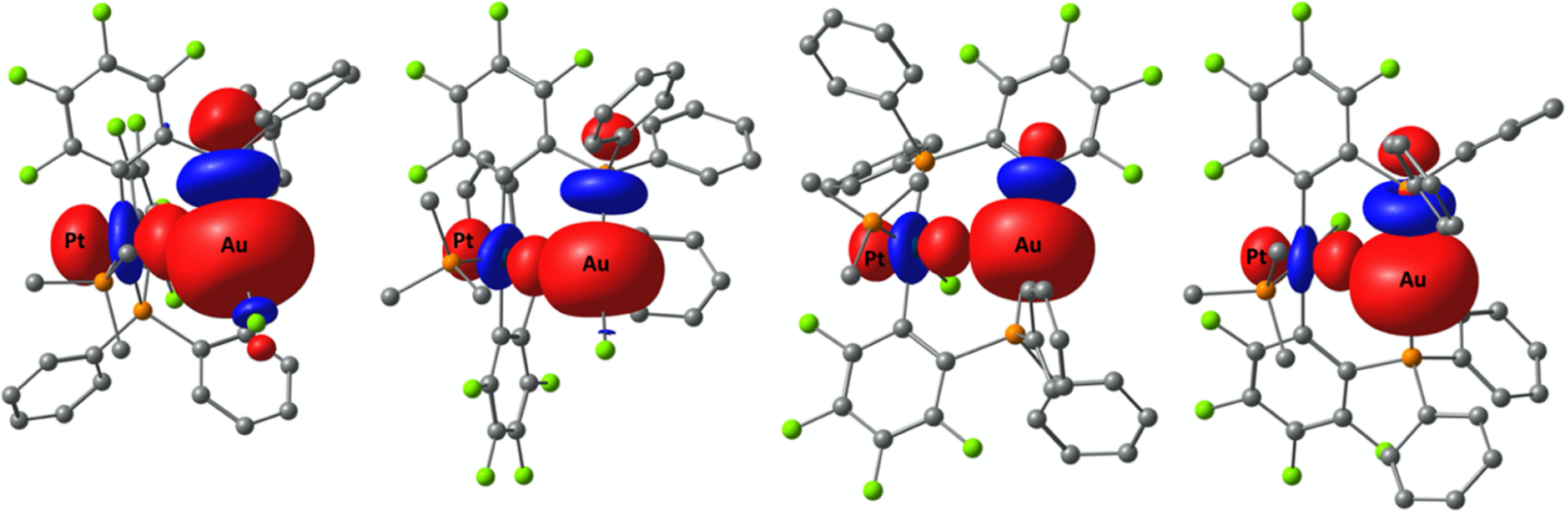
LP(Pt)→BD*(Au′–C/P) main donor–acceptor interaction derived by NBO analyses of *cis*-**6PtAu** (left), *trans*-**6PtAu** (middle left), *trans*-**7PtAu** (middle right), and *trans*-**8PtAu** (right). NBOs are displayed with an isosurface value of 0.05.

The metal–metal interactions in complexes of type **6**, **7**, and **8** (M = Ni, Pt; M′ = Cu, Au) were investigated in an analogous manner to the complexes of type **5** (vide supra). According to the NBO/NLMO calculations, the energy levels of the main donor-orbital of Pt with one PMe_3_ ligand in their coordination sphere are ranging between −0.22 and −0.24 au (Table 6, Figure 10) and are very similar to the energy levels of the complexes of type **5** (−0.21 to −0.22 au, Table 3).

**Table 6:** NC and NBO Characteristics of the Main Donor–Acceptor Interaction [LP—Valence Lone Pair, LV—Lone Vacant Orbital, BD*—Valence Antibond, NBO Energy Level (*E*_NBO_, au), Donor–Acceptor Energy (kcal mol^–1^)] and WBO of Complexes of the Type **6MM′**, **7MM′**, and **8MM′** (M = Ni, Pt; M′ = Cu, Au)

	*cis*-**6PtCu**	*cis*-**6PtAu**	*trans*-**6PtAu**	*cis*–*trans*-**7NiAu**	*trans*-**7PtAu**	*trans*-**8NiCu**·acetone	*trans*-**8PtAu**
NC(M)	0.24	0.26	0.25	0.47	0.24	0.54	0.34
NC(M′)	0.61	0.37	0.38	0.36	0.35	0.72	0.30
*E*_NBO_(LP(M))	–0.23	–0.22	–0.22	–0.31_Au_	–0.24	–0.25	–0.23
LP(M)	96% d	96% d	97% d	89% d_Au_	97% d	99% d	97% d
*E*_NBO_(LV(M′))	0.05					0.14	
LV(M′)	95% s					99% s	
*E*_NBO_(BD*(M′–P/C))		0.39	0.38	0.09_Ni–P_	0.22		0.20
BD*(M′–P/C)		74% Au (88% s, 12% d)	74% Au (88% s, 12% d)	75% Ni (57% s, 43% d)	74% Au (86% s, 13% d)		74% Au (90% s, 10% d)
*E*_D→A_	26.44	6.62	13.94	4.82	14.10	1.71	19.20
WBO	0.36	0.30	0.37	0.32	0.41	0.30	0.44

The Pt···Cu separation in *cis*-**6PtCu** is significant longer, by approximately 0.06 Å, in comparison to *trans*-**5PtCu** and *cis*-**5PtCu**, caused by steric repulsion of the chloride atom toward the ligand backbone. The topologic analysis (Table 7) of *cis*-**6PtCu** reveals a bcp between Pt and Cu with the same characteristics found in *trans*-**5PtCu** and *cis*-**5PtCu** (1 < |*V*(***r***_**b**_)|/*G*(***r***_**b**_) < 2, ∇^2^ρ(***r***_**b**_) > 0, *G*(***r***_**b**_) ≙ |*V*(***r***_**b**_)|, *H*(***r***_**b**_) < 0, WBO = 0.36–0.37), categorizing the Pt···Cu interaction in *cis*-**6PtCu** as predominantly metallic (shared) with an additional donor–acceptor bonding characteristic. However, the copper contribution in the main donor lone pair located at Pt in *cis*-**6PtCu** is about 0.29% and is similar to *trans*-**5PtCu** (0.24%) and *cis*-**5PtCu** (0.34%). Also, the lower acceptor orbital energy at copper (linear Cl–Cu–P arrangement) in *cis*-**6PtCu** (by about 0.1 au) in comparison to *trans*-**5PtCu** and *cis*-**5PtCu** (trigonal planar Cl–Cu–P_2_ arrangement) leads to a less intense interaction energy (Δ*E*_2_ ≈ 13 kcal mol^–1^). Therefore, a formal loss of Pt···Cu interaction intensity is present going from *cis*-**6PtCu** to either *trans*-**5PtCu** or *cis*-**5PtCu**, which is also reflected in lower ρ(***r***_**b**_) in *cis*-**6PtCu**. This behavior of Pt···Cu interaction intensity is visible by a shortening of the Pt···Cu separation in complexes of type **5** over **6** due to the absence of steric repulsion. In contrast, loss of interaction intensity between Pt···Cu caused by an increase in the coordination number of copper was observed between [(dppe)Pt(κC-2-C_6_F_4_PPh_2_)(μ-2-C_6_F_4_PPh_2_)CuCl] (linear Cl–Cu–P arrangement with significant donor–acceptor interactions) and [(dppe)Pt(μ-2-C_6_F_4_PPh_2_)_2_CuCl] (trigonal planar Cl–Cu–P_2_ arrangement absence of significant metallophilic interaction), where steric repulsion is present in both configurations.^15^ Interestingly, in complexes with a trigonal planar ClM′(PR_3_)_2_ unit (type **5**) and complexes with a linear ClM′PR_3_ unit (type **6**) a trend of stronger Pt→M′ donor–acceptor interaction with M′ = Au < Cu is observed.

**Table 7:** Results of the Theoretical Topological Analysis of the Bond Critical Points (3, −1) between M and M′ in Complexes **6MM′**, **7MM′**, and **8MM′** (M = Ni, Pt; M′ = Cu, Au)^a^

	*cis*-**6PtCu**	*cis*-**6PtAu**	*trans*-**6PtAu**	*cis*–*trans*-**7NiAu**	*trans*-**7PtAu**	*trans*-**8NiCu**·acetone	*trans*-**8PtAu**
ρ(***r***_**b**_)	0.02865	0.02747	0.03683	0.02971	0.04399		0.04910
∇^2^ρ(***r***_**b**_)	0.06417	0.06334	0.08831	0.06891	0.10923		0.11946
*G*(***r***_**b**_)	0.01908	0.01815	0.02673	0.02119	0.03430		0.03867
*V*(***r***_**b**_)	–0.02213	–0.02048	–0.03143	–0.02516	–0.04137		–0.04757
|*V*(***r***_**b**_)|/*G*(***r***_**b**_)	1.160	1.129	1.176	1.187	1.206		1.230
*G*(***r***_**b**_)/ρ(***r***_**b**_)	0.666	0.661	0.726	0.713	0.780		0.788
*H*(***r***_**b**_)	–0.00305	–0.00233	–0.00470	–0.00397	–0.00707		–0.00890
sign(λ_2_(***r***_**b**_))ρ(***r***_**b**_)	–0.029	–0.027	–0.037	–0.030	–0.044		–0.049

a Electron density (ρ(***r***_**b**_) in au), Laplacian of electron density (∇^2^ρ(***r***_**b**_) in au), Lagrangian kinetic energy density (*G*(***r***_**b**_) in au), potential energy density (*V*(***r***_**b**_) in au), ratio |*V*(***r***_**b**_)|/*G*(***r***_**b**_), ratio *G*(***r***_**b**_)/ρ(***r***_**b**_) in au, electron energy density (*H*(***r***_**b**_) in au), ratio *H*(***r***_**b**_)/ρ(***r***_**b**_) in au, the product of sign of the second largest eigenvalue of Hessian matrix of electron density (λ_2_(***r***_**b**_)), and ρ(***r***_**b**_) in au.

In the series of Pt–Au complexes, the Pt···Au distances are decreasing in the order *cis*-**6PtAu** > *trans*-**6PtAu** > *trans*-**5PtAu** > *trans*-**7PtAu** > *trans*-**8PtAu**. Topologic analyses of the 5 complexes reveal the expected characterization at the bcps (1 < |*V*(***r***_**b**_)|/*G*(***r***_**b**_) < 2, ∇^2^ρ(***r***_**b**_) > 0, *G*(***r***_**b**_) ≙ |*V*(***r***_**b**_)|, *H*(***r***_**b**_) < 0, WBO = 0.30–0.44), categorizing the Pt···Au interactions in **PtAu** complexes as predominantly metallic (shared) with an additional donor–acceptor bonding characteristic. The NCI descriptor and ELF increases in the expected trend from *cis*-**6PtAu** < *trans*-**6PtAu** < *trans*-**5PtAu** < *trans*-**7PtAu** < *trans*-**8PtAu** (Figures S67–S70) and is in agreement with the trend of the Pt···Au distances. The magnitudes of ρ(***r***_**b**_) and ∇^2^ρ(***r***_**b**_) and WBO are increasing in the same order, which is indicative for an increase of strength of the metallophilic interactions. The *G*(***r***_**b**_)/ρ(***r***_**b**_) ratio is getting closer to 1, which suggests more intense donor–acceptor type interaction going from *cis*-**6PtAu** to *trans*-**8PtAu**. The magnitude of *E*_NBO_ main LP→LV/BD* interaction does follow the expected trend as observed by AIM analyses for complexes with a linear coordination sphere at Au (complexes type **6PtAu**, **7PtAu**, and **8PtAu**, Figure 10).

Complex *cis*–*trans*-**7NiAu** shows similar characteristics at the bcp as observed in the series of Pt–Au-complexes (1 < |*V*(***r***_**b**_)|/*G*(***r***_**b**_) < 2, ∇^2^ρ(***r***_**b**_) > 0, *G*(***r***_**b**_) ≙ |*V*(***r***_**b**_)|, *H*(***r***_**b**_) < 0, WBO = 0.32). NBO analysis reveals a stronger Ni←Au donor–acceptor interaction over Ni→Au by 6.27 kcal mol^–1^, which is also the case for *trans*-**7PtAu** (Σ*E*_NBO_(Pt→Au) < Σ*E*_NBO_(Pt←Au) by 1.18 kcal mol^–1^). In complexes of type **7,** the gold atom is a stronger donor over the d^8^-metal (see the Supporting Information for details).

**Figure 11:**
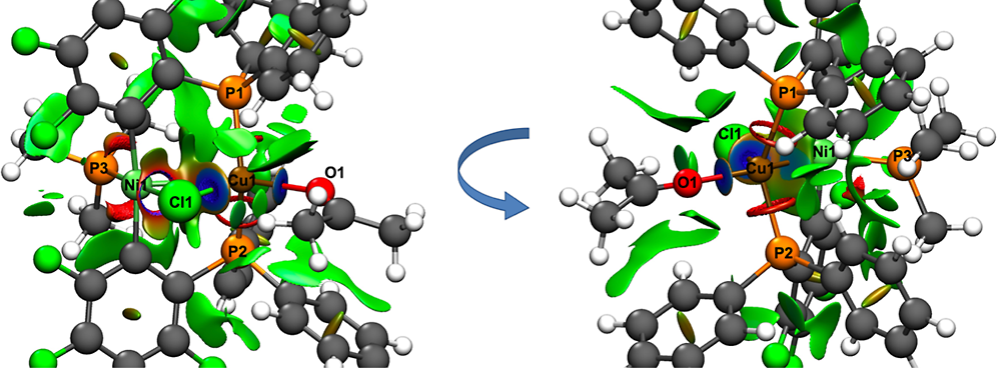
NCI descriptor of *trans*-**8NiCu**·acetone. Isovalue is set to 0.45, and color range is from −0.05 to 0.05 au; color code: blue—attractive interactions; green—van der Waals interactions; red—non-attractive interactions.

NBO calculations at *trans*-**8NiCu** reveal that the Cl atom acts as a lone pair donor toward the copper center (∑*E*_NBO/LP(Cl)→LV(Cu)_ = 30.78 kcal mol^–1^). The NC at Cl (−0.61) and Cu (0.72) supports the presence of attractive coulomb interaction. We find the Cl atom to be covalently bound to nickel and showing attractive dative bonding modes toward copper (WBO_Cu–Cl_ = 0.77 vs WBO_Ni–Cl_ = 1.01), which is in agreement with the interatomic distances. The Cu1···O1 distance is approximately 2.20(1) Å and significantly longer than the sum of the covalent radii (1.98(4) Å).^20^ A similar donor–acceptor behavior between Cu and O (∑*E*_NBO/LP(O)→LV(Cu)_ = 19.98 kcal mol^–1^, NC_O_: −0.61, WBO_Cu–O_ = 0.44) is observed for Cu and Cl but with less intensity. The presence of formal weak Cu1···O1 interactions was also observed in complexes with O=PPh(C_6_H_4_PPh_2_)_2_ as the donor in the copper coordination sphere.^28^ The calculation of the NCI descriptor for *trans*-**8NiCu**·acetone underlines the presence of attractive NCI between Cu1···Cl1 and Cu1···O1 (Figure 11).

The Ni···Cu separation in *trans*-**8NiCu** is significantly shorter than in compound *trans*-**5NiCu** (by 0.1 Å), which can be explained by an additional chloride bridge in *trans*-**8NiCu**. However, AIM calculations did not support a bcp between Ni and Cu in *trans*-**8NiCu**. We address this absence of a bcp with three parameters: (i) Cu is distorted tetrahedral coordinated by P, Cl, and O donor atoms and is therefore sterically saturated for weak covalent interactions, (ii) the NCs for Ni and Cu are significantly higher in magnitude and therefore the Ni and Cu are more positively charged in comparison to other complexes presented in this study, and (iii) the donor–acceptor possibility is reduced by a comparative large gap between donor and acceptor orbital energy levels. According to the NCI descriptor, the Ni···Cu interaction should be described as very weak attractive non-covalent intermetallic (dispersion) interaction.

## Conclusions

It has been shown that the homologous compounds *trans*-[(Me_3_P)_2_M(κ*C*-2-C_6_F_4_PPh_2_)_2_] (M = Ni (**2Ni**^**a**^), Pt (**2Pt**^**a**^)) are accessible by the addition of excess PMe_3_ to *trans*-[M(κ^2^-2-C_6_F_4_PPh_2_)_2_] (M = Ni (**1Ni**), Pt (**1Pt**)). Complexes of the type [(Me_3_P)M(κ^2^-2-C_6_F_4_PPh_2_)(κC-2-C_6_F_4_PPh_2_)] (M = Ni (**3Ni**^**b**^), Pt (**3Pt**^**b**^)) were accessible by the stoichiometric addition of PMe_3_ to either *trans*-**1Ni** or *cis*-**1Pt**. As byproducts, complexes of the type [M_2_(κ^2^-2-C_6_F_4_PPh_2_)_2_(μ-2-C_6_F_4_PPh_2_)_2_] (M = Ni (**4Ni**), Pt (**4Pt**)) could be isolated in pure state. Treatment of **2Ni**^**a**^, **2Pt**^**a**^, **3Ni**^**b**^, or **3Pt**^**b**^ with coinage metal chlorides gave bimetallic complexes of type *trans*-[(Me_3_P)_2_M(μ-2-C_6_F_4_PPh_2_)_2_M′Cl] (*trans*-**5MM′**; M = Ni, Pt; M′ = Cu, Ag, Au), [(Me_3_P)M(κ^2^-2-C_6_F_4_PPh_2_)(μ-2-C_6_F_4_PPh_2_)M′Cl]_*x*_ (*x* = 1: *cis*-**6PtCu**; *x* = 2: *cis*-**6PtAg-dimer**), or [(Me_3_P)ClM(μ-2-C_6_F_4_PPh_2_)M′] (*cis*–*trans*-**7NiAu**, *trans*-**7PtAu**, *trans*-**8NiCu**, *trans*-**8NiAg**). Treatment of *trans*-**1Pt** with [AuCl(PMe_3_)] gave *trans*-**6PtAu** as an intermediate, which isomerized to *trans***-8PtAu**, whereas treatment of *cis*-**1Pt** with [AuCl(PMe_3_)] resulted in the formation of *cis*-**6PtAu**, which stayed in equilibrium with *trans*-**7PtAu**. From the reaction of *cis*-**6PtCu** or *cis*-**6PtAg-dimer** with 1 equiv PMe_3_, the homologous complexes *cis*-[(Me_3_P)_2_Pt(μ-2-C_6_F_4_PPh_2_)_2_M′Cl] (*cis*-**5PtM′**; M′ = Cu, Ag) could be observed. Quantum chemical calculations (AIM, ELF, NCI, and NBO) gave insight into the M···M′ interaction characteristics. The interaction type reaches from pure attractive non-covalent (*trans*-**8NiCu**·acetone) to bonds with intermediate character (not purely ionic in nature but have some electron-shared (covalent) character). This intermediate character could be further divided into interaction types with predominantly metallic (shared) bonding (Ni–Cu, Pd–Cu, Pd–Ag, Pt–Au) and additional with donor–acceptor bonding characteristic (Ni←Au, Pd←Au, Pt←Au, Pt→Cu, Pt→Ag, Pt→Au). The interaction strength is increasing in the order Ni < Pd < Pt (at the same coinage metal and in the same complex type). Depending on the coinage metal coordination sphere, the interaction strength along group 11 is either increasing (e.g., *trans*-**5MM′**, trigonal-planar coordination at M′) or decreasing (e.g., *trans*-**5MM′**, linear coordination at M′). Along a series of Pt–Au complexes, it could be demonstrated that with the following changes, (1) decreasing steric hindrance (from *cis*-**6PtAu** to *trans*-**6PtAu**), (2) double over single bridged Pt–Au (from *trans*-**6PtAu** to *trans*-**7PtAu**), and (3) accumulated negative charged ligands at Pt and neutral ligands at Au (from *trans*-**7PtAu** to *trans*-**8PtAu**), the Pt···Au interaction can be intensified.

## Experimental Section

### General Comments

Dichloromethane, diethyl ether, and *n*-hexane were dried by the use of a standard column drying system, and tetrahydrofuran was distilled from Na/benzophenone. [M(κ^2^-2-C_6_F_4_PPh_2_)_2_] (M = Ni, Pd, Pt),^13^ [AuCl(tht)]^29^ (tht = tetrahydrothiophene), and [AuCl(PMe_3_)]^30^ (from [AuCl(tht)] with PMe_3_ solution in CH_2_Cl_2_) were prepared by the literature methods. CuCl and AgCl were prepared by the literature methods.^15^ Other chemicals were commercially available and used as received. ^1^H (300, 500 MHz), ^19^F (282, 471 MHz), and ^31^P (121, 202 MHz) NMR spectra were measured as CDCl_3_ solutions, unless otherwise stated, on a Bruker Avance 300 or Bruker Avance 500 spectrometer at room temperature. Chemical shifts were initially referenced to residual solvent signals (^1^H), CFCl_3_ (^19^F), or external 85% H_3_PO_4_ (^31^P). Electrospray mass spectra were measured for *cis*-**7NiAu**, *trans*-**8NiCu**, and *trans*-**8NiAg** on an “expression CMS L” spectrometer (Advion, Ithaca, USA) or for all other compounds on an “HP 5970 MSD” spectrometer. Elemental analyses were carried out by the Microanalytical Unit at the Research School of Chemistry, ANU.

### Single-Crystal X-ray Crystallography

Crystals suitable for single-crystal X-ray diffraction were obtained from dichloromethane (*trans*-**5PtAg**), benzene/MeOH (*syn*-**2Ni**^**a**^), dichloromethane/methanol (*syn*-**2Pt**^**a**^·0.88(CH_2_Cl_2_), **3Pt**^**b**^, **4Ni**·0.63(CH_2_Cl_2_), **4Pt**·0.52(CH_2_Cl_2_), *trans*-**5NiCu**), diethyl ether/MeOH (**3Ni**^**b**^), dichloromethane/*n*-hexane (**3Pt**^**b**^, *trans*-**5PtCu**, *cis*-**5PtCu**, *cis*-**5PtAg**, *cis*-**6PtCu**, *cis*-**6PtAg-**dimer, *trans*-**6PtAu**·0.72(CH_2_Cl_2_), *cis*–*trans*-**7NiAu**, *trans*-**7PtAu**, *trans*-**8PtAu**), dichloromethane/MeOH/*n*-hexane (*trans*-**5PtAu**), toluene/*n*-hexane (*cis*-**6PtAu**), or acetone/*n*-hexane (*trans*-**8NiCu**·2(C_3_H_6_O)). Using a drop of inert oil (Nujol), crystals were mounted on a nylon loop or glass capillary and transferred into a stream of cold nitrogen. For *cis*–*trans*-**7NiAu** and *trans*-**8NiCu**, the single-crystal diffraction data sets were collected with ω-scans at an “IPDS-2(T)” diffractometer (STOE, Darmstadt, Germany) using Mo Kα radiation (λ = 0.71073 Å). The absorption correction was performed with XShape using the integration correction type. For all other compounds, the reflections were collected on a D8 Bruker diffractometer equipped with an APEX-II area detector using graphite-monochromated Mo Kα radiation (λ = 0.71073 Å) from a 1 μS micro source. The computer programs SMART^31^ and SAINT^32^ were used for data collection in φ- and ω-scan modes and data processing, respectively, and absorption corrections were done using SADABS.^33^ All structures were solved using direct methods and refined with full-matrix least-squares methods on *F*^2^ using the SHELX-TL package.^34,35^ X-ABS2 was used for *syn*-**2Pt**^**a**^·0.88(CH_2_Cl_2_), *trans*-**7PtAu**, *trans*-**5PtAg**, and *cis*-**5PtAg** to perform the absorption correction.^36^ Parameters of data collection and structure refinement of the crystal structures discussed in this paper are reported in the Supporting Information. CIF files have been deposited with the Cambridge Crystallographic Data Center (CCDC) and can be obtained free of charge (for inquiry contact: CCDC, 12 Union Road, Cambridge, CB2 1EZ, UK, fax: +44-1223-336033, e-mail: deposit@ccdc.cam.ac.uk) quoting the following reference numbers: CCDC-2027717 (*syn*-**2Ni**^**a**^), 2027716 (*syn*-**2Pt**^**a**^·0.88(CH_2_Cl_2_)), 2027705 (**3Ni**^**b**^), 2027707 (**3Pt**^**b**^ (*P*2_1_/*c*)), 2027712 (**3Pt**^**b**^ (*Pn*)), 2027710 (**4Ni**·0.63(CH_2_Cl_2_)), 2027711 (**4Pt**·0.52(CH_2_Cl_2_)), 2027719 (*trans*-**5NiCu**), 2027720 (*trans*-**5PtCu**), 2027722 (*trans*-**5PtAu**), 2027709 (*cis*-**5PtCu**), 2027706 (*cis*-**5PtAg**), 2027715 (*cis*-**6PtCu**), 2027721 (*cis*-**6PtAg-dimer**), 2027708 (*cis*-**6PtAu**), 2027727 (*trans*-**6PtAu**·0.722(CH_2_Cl_2_)), 2027713 (*cis*–*trans*-**7NiAu**), 2027725 (*trans*-**7PtAu**), 2027724 (*trans*-**8NiCu**·2(C_3_H_6_O)), 2027726 (*trans*-**8PtAu**), 2027714 (**A**), 2027718 (**B1**), 2027723 (**B2**·CH_2_Cl_2_).

### Quantum Chemical Calculations

The geometry optimizations were carried out with ORCA 5.0.2 or 5.0.3^37^ using the restricted PBE0 functional with relativistically recontracted Karlsruhe basis sets ZORA-def2- TZVPP^38^ (for H, C, F, P, Cl, Ni, Cu) and SARC-ZORA-TZVPP (for Pd, Ag, Pt, Au),^39^ the scalar relativistic ZORA Hamiltonian,^40^ atom-pairwise dispersion correction with the Becke–Johnson damping scheme (D3BJ),^41^ and COSMO solvation (CH_2_Cl_2_, ε = 8.9, rsolv = 3.55, xfeps = 0.8333).^42^ Very-TightSCF and slowconv options were applied, and the DEFGRID3 was used with a radial integration accuracy of 10 for all transition-metal atoms for all calculations. Calculations were started from the molecular structures obtained by single-crystal X-ray diffraction analysis, and isomers were created by modifying these structures. Numerical frequency calculations were performed to prove convergence at the local minimum after geometry optimization and to obtain the Gibbs free energy (293.15 K). After optimization of the H-atom positions of the molecular structures obtained by single-crystal X-ray diffraction analyses, NBO and NLMO calculations were performed using Gaussian09^43^ with the NBO6 package^22^ using the restricted PBE0 functional with Karlsruhe basis sets def2-TZVPP (for all atoms).^38^ NBO and ELF graphics were generated using ChemCraft.^44^ NCI,^21^ ELF,^23^ AIM^24^ and WBO^45^ calculations were carried out by using MultiWFN^46^ at the same level of theory as used for NBO/NLMO calculations. The NCI results were depicted with VMD.^47^

## Supporting Information Available

Synthesis; XRD; ^1^H, ^19^F, ^31^P NMR spectra; parameters of data collection and structure refinement; discussion of molecular structures; NMR calculations; structural overlay of *trans*-5NiCu, *trans*-5PdCu, and *trans*-5PtCu; additional ELF and NCI; second-order perturbation theory analysis of Fock matrix in NBO basis; and graphical representations of optimized molecular structures, total energies, and atomic coordinates (PDF)
