# Supplementary material for: Formation of Heterobimetallic Complexes by Addition of d10-Metal Ions to [(Me3P)xM(2-C6F4PPh2)2] (x = 1, 2; M = Ni and Pt): A Synthetic and Computational Study of Metallophilic Interactions
Source: Inorg Chem. 2023 May 31;62(23):8846–62. doi: 10.1021/acs.inorgchem.3c00311 (PMC10265710; doi:10.1021/acs.inorgchem.3c00311)
Supplement: Supplementary file 1 — ic3c00311_si_001.pdf [file ic3c00311_si_001.pdf]

Formation of Heterobimetallic Complexes by Addition of d^{10}-Metal Ions to $[(Me_3P)_xM(2\text{-}C_6F_4PPh_2)_2]$ (x = 1, 2; M = Ni and Pt): A Synthetic and Computational Study of Metallophilic Interactions

Robert Gericke,[§,†] Martin A. Bennett,[‡] Steven H. Privér,[§] and Suresh K. Bhargava,[§]

[§] School of Applied Sciences (Applied Chemistry), RMIT University,

GPO Box 2476, Melbourne, Victoria 3001, Australia

[†] Institute of Resource Ecology, Helmholtz-Zentrum Dresden-Rossendorf e.V.,

Bautzner Landstraße 400, 01328 Dresden, Germany

[‡] Research School of Chemistry, Australian National University,

Canberra, ACT 2601, Australia

Contents:

1. Synthesis & ^{1}H, ^{19}F, ^{31}P NMR spectra — page 2
2. Parameters of data collection and structure refinement — page 45
3. Discussion of molecular structures of syn-**2M**[a], **3M**[b] and **4M** (M = Ni, Pt) — page 53
4. Discussion of molecular structures of *trans*-**5MCu** (M = Ni, Pd, Pt) — page 54
5. NMR calculations — page 55
6. Discussion of molecular structures of *cis*-**6PtCu** and *cis*-**6PtAg-dimer** — page 56
7. Discussion of molecular structures of *cis*-**6PtAu**, *trans*-**6PtAu**, *trans*-**7PtAu** and *trans*-**8PtAu** — page 58
8. Discussion of molecular structures of *trans*-**8NiCu**·acetone and *cis-trans*-**7NiAu** — page 58
9. Structural overlay of *trans*-**5NiCu**, *trans*-**5PdCu** and *trans*-**5PtCu** and molecular structures of **A** and **B** — page 59
10. Additional ELF — page 60
11. Additional NCI — page 60
12. Second Order Perturbation Theory Analysis of Fock Matrix in NBO Basis — page 61
13. Graphical representations of optimized molecular structures, total energies and atomic coordinates — page 65
14. Literature — page 217

1. Syntheses:

***trans*-[(Me$_3$P)$_2$Ni(*κC*-2-C$_6$F$_4$PPh$_2$)$_2$] (2Ni^a).** To a stirred yellow solution of *trans*-[Ni(κ^2-2-C$_6$F$_4$PPh$_2$)$_2$] (150 mg, 207 µmol) in 15 mL dichloromethane was added 1 M PMe$_3$-toluene-solution (600 µL). During this time the colour of the solution changed to deep red and back to yellow. After stirring the solution for 15 min at ambient temperature, methanol (10 mL) was added and the volume of the solution was reduced. The pale yellow solid was filtered off, washed with methanol and dried at 40 °C *in vacuo*. Yield: 156 mg (178 µmol, 86%).

Proportions are according to the ^{19}F NMR spectrum, which shows the expected product in two isomeric forms (1:8 = isomer 1: isomer 2) and 5% impurity of [(Me$_3$P)Ni(κ^2-2-C$_6$F$_4$PPh$_2$)$_2$].

^{1}H NMR (C$_6$D$_6$): 0.39 (m, 18H, PMe$_3$), 6.95-7.11 (br. m, 12H, aryl), 7.57-7.66 (br. m, 1H, aryl), 7.71-7.86 (br. m, 7H, aryl). ^{19}F NMR (C$_6$D$_6$): -111.1 (m, 2F, isomer 1), -111.7 (m, 16F, isomer 2), -117.4 (m, 2F, isomer 1), -119.6 (m, 16F, isomer 2), -154.9 (m, 2F, isomer 1), -155.3 (m, 16F, isomer 2), -159.8 to -160.2 (m, 18F, isomer 1 + 2). ^{31}P NMR (C$_6$D$_6$): -3.6 (br. m, 2P, PPh$_2$, isomer 1), -9.4 (m, 16P, PPh$_2$, isomer 2), -15.0 (m, 18P, PMe$_3$, isomer 1+2). Anal. Calcd. for C$_{42}$H$_{38}$F$_8$P$_4$Ni (MW: 877.3): C 57.50, H 4.37, F 17.32; found: C 57.62, H 4.24, F 17.49. ESI-MS (m/z): 877.08 [M]$^+$

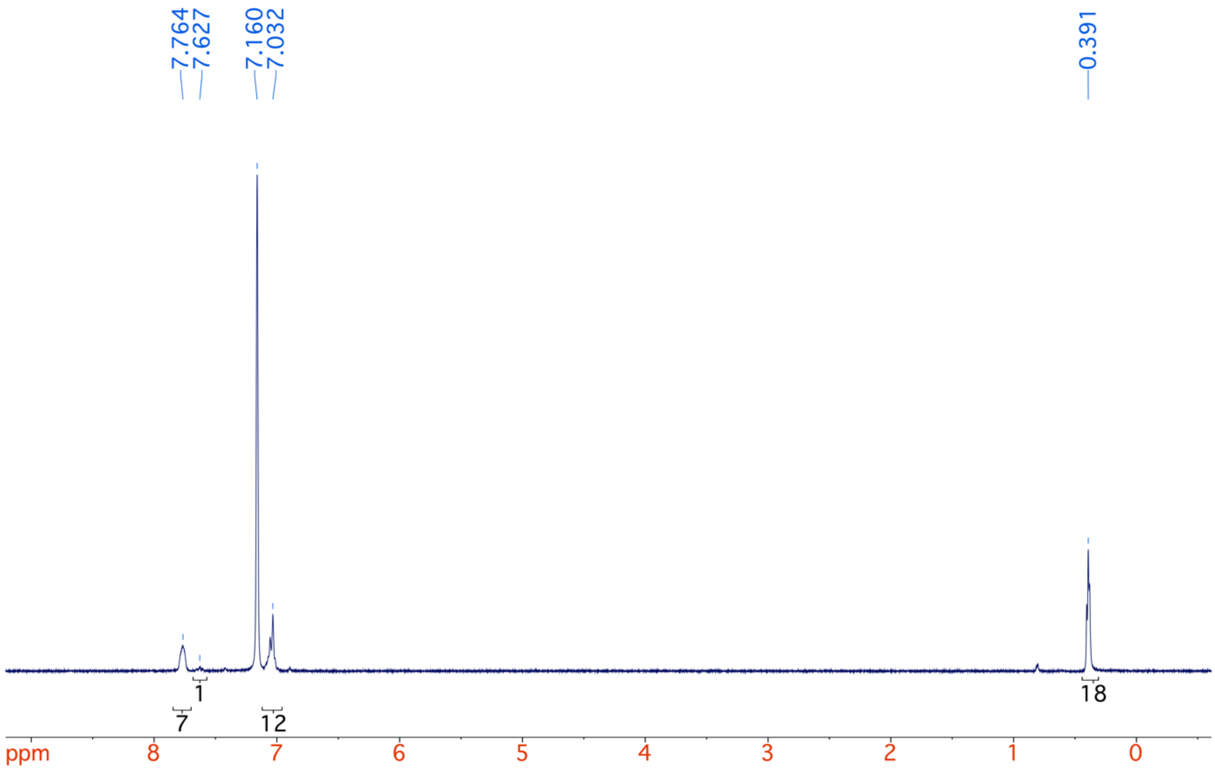

Figure S1. ^{1}H NMR spectrum in C$_6$D$_6$ of *trans*-[(Me$_3$P)$_2$Ni(*κC*-2-C$_6$F$_4$PPh$_2$)$_2$] (**2Ni^a**).

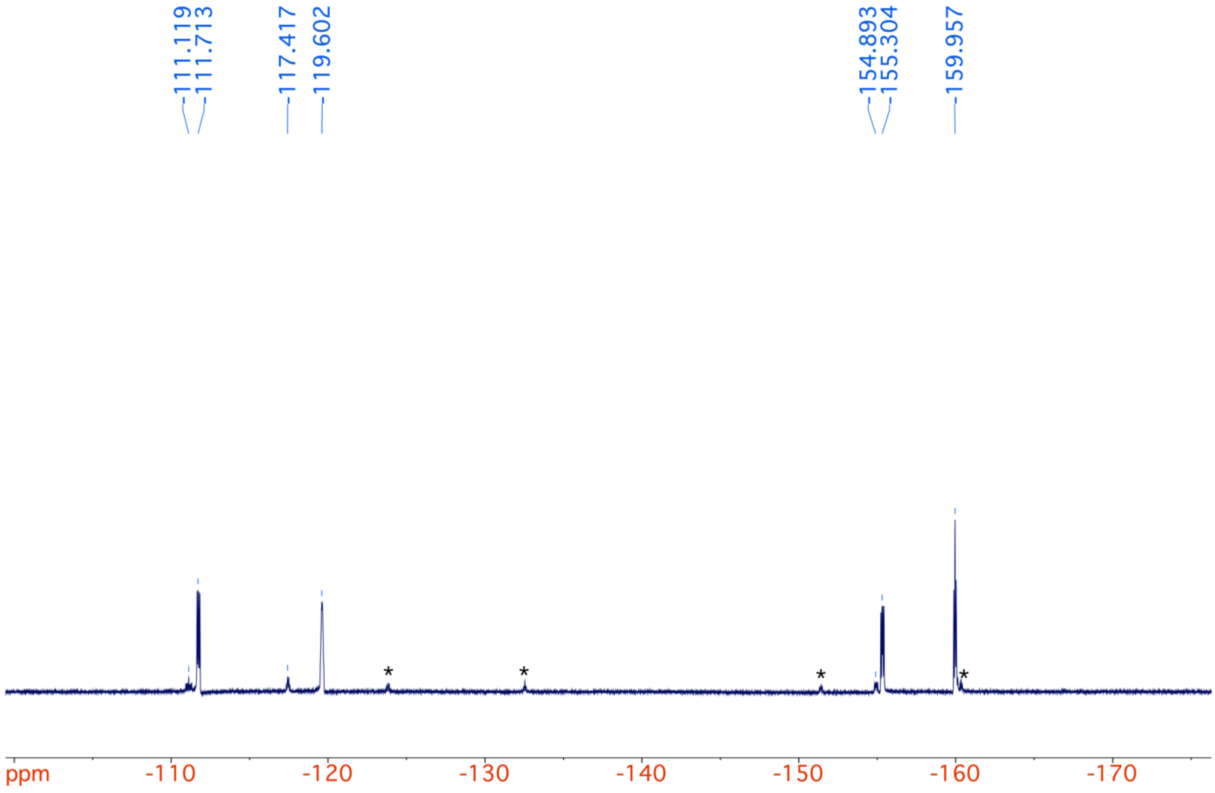

Figure S2. ^{19}F NMR spectrum in C_6D_6 of *trans*-[($Me_3P)_2Ni(\kappa C$-2-$C_6F_4PPh_2)_2$] (**2Ni**[a]). Impurities of [($Me_3P)Ni(\kappa^2$-2-$C_6F_4PPh_2)_2$] are marked with asterisks.

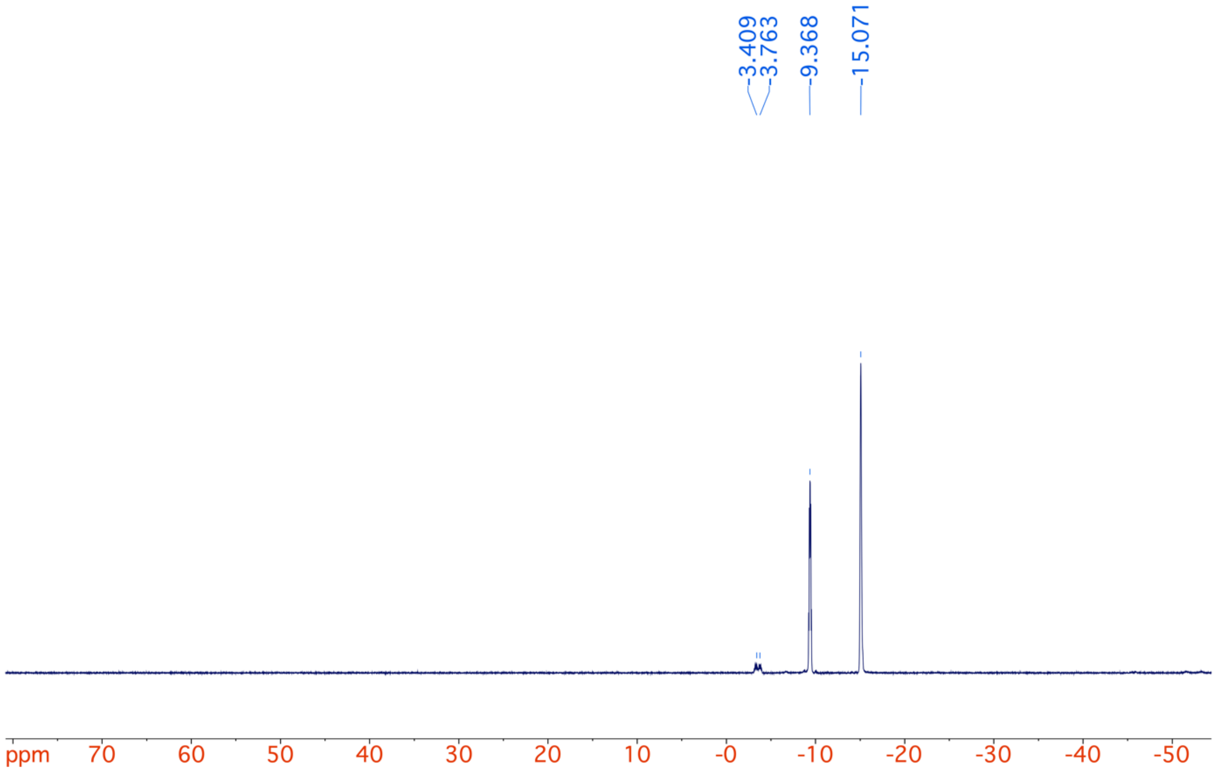

Figure S3. ^{31}P NMR spectrum in C_6D_6 of *trans*-[($Me_3P)_2Ni(\kappa C$-2-$C_6F_4PPh_2)_2$] (**2Ni**[a]).

***trans*-[(Me$_3$P)$_2$Pt(κC-2-C$_6$F$_4$PPh$_2$)$_2$] (2Pt^a).** To a stirred solution of *trans*-[Pt(κ^2-2-C$_6$F$_4$PPh$_2$)$_2$] (100 mg, 116 µmol) in dichloromethane (15 mL) was added dropwise 1 M PMe$_3$-toluene-solution (236 µL). The clear solution was stirred at ambient temperature for 5 min. After adding methanol (10 mL) the volume of the solution was reduced. The white solid was filtered off, washed with methanol and air dried. Yield: 81 mg (80 µmol, 69%).

^{1}H NMR (C$_6$D$_6$): 0.55 (m, 18H, PMe$_3$), 6.98-7.11 (br. m, 12H, aryl), 7.66-7.77 (m, 8H, aryl). ^{19}F NMR (C$_6$D$_6$): -112.9 (br. m), -120.5 (m), -154.9 (m), -160.4 (m). ^{31}P NMR (C$_6$D$_6$): -11.1 (m, $^3J_{Pt,P}$ = 90 Hz, PPh$_2$), -24.6 (m, $^1J_{Pt,P}$ = 2700 Hz, PMe$_3$). Anal. Calcd. for C$_{42}$H$_{38}$F$_8$P$_4$Pt (MW: 1013.7): C 49.76, H 3.78, F: 14.99; found: C 49.84, H 3.65, F 14.81. ESI-MS (m/z): 1014.15 [M+H]$^+$

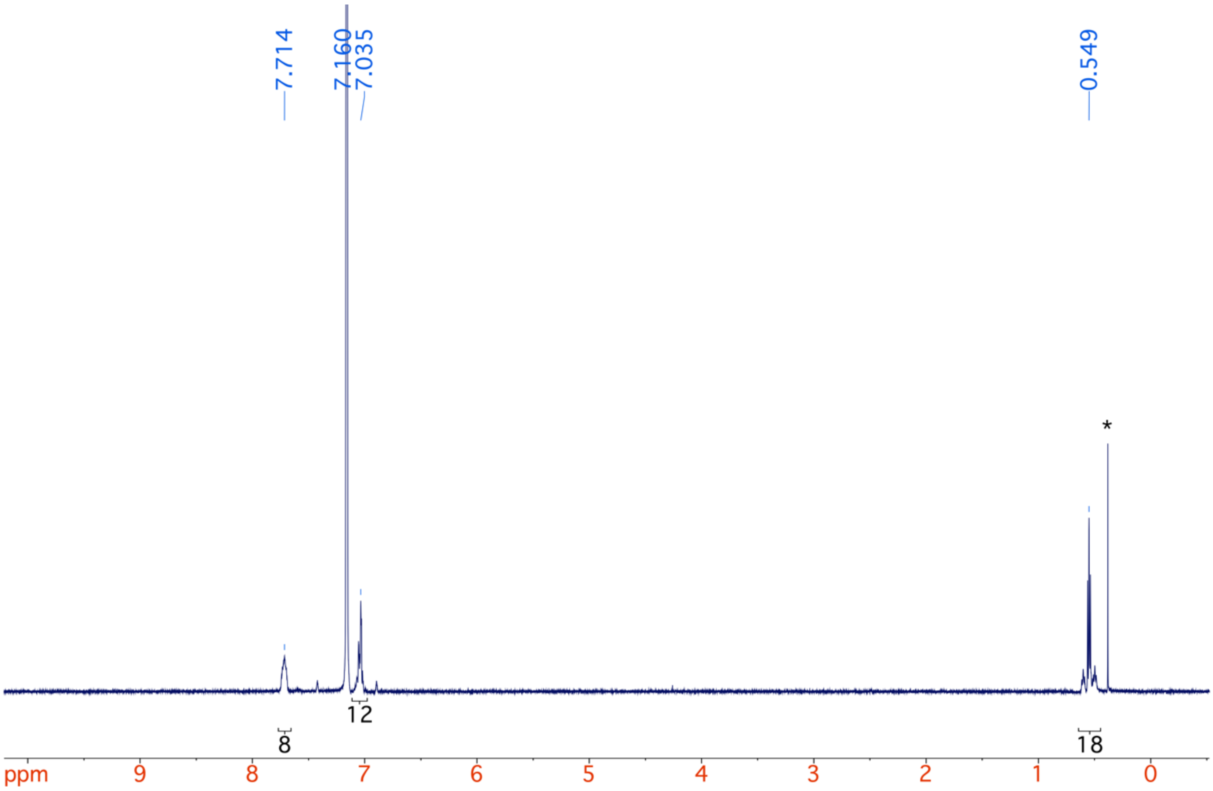

Figure S4. ^{1}H NMR spectrum in C$_6$D$_6$ of *trans*-[(Me$_3$P)$_2$Pt(κC-2-C$_6$F$_4$PPh$_2$)$_2$] (**2Pt^a**). ^{1}H NMR signal of H$_2$O is marked with asterisk.

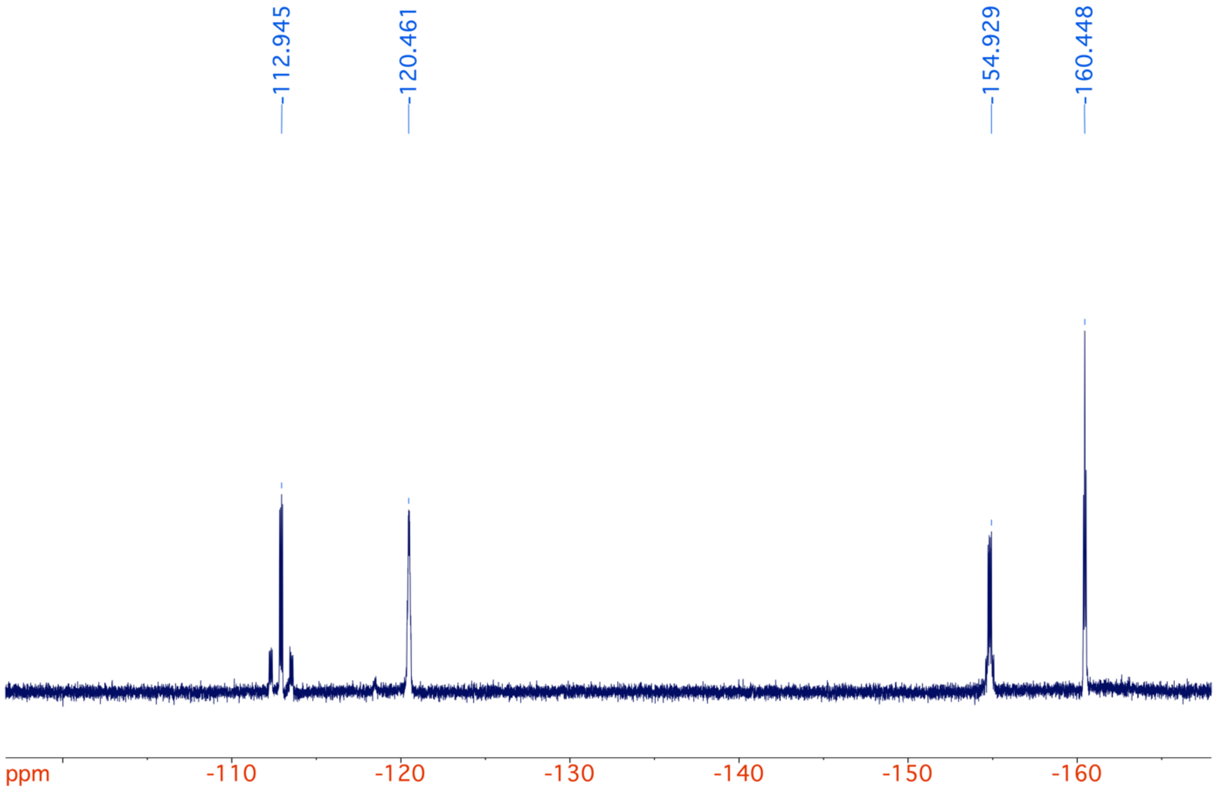

Figure S5. ^{19}F NMR spectrum in C_6D_6 of *trans*-[$(Me_3P)_2Pt(\kappa C$-2-$C_6F_4PPh_2)_2$] (**2Pt^a**).

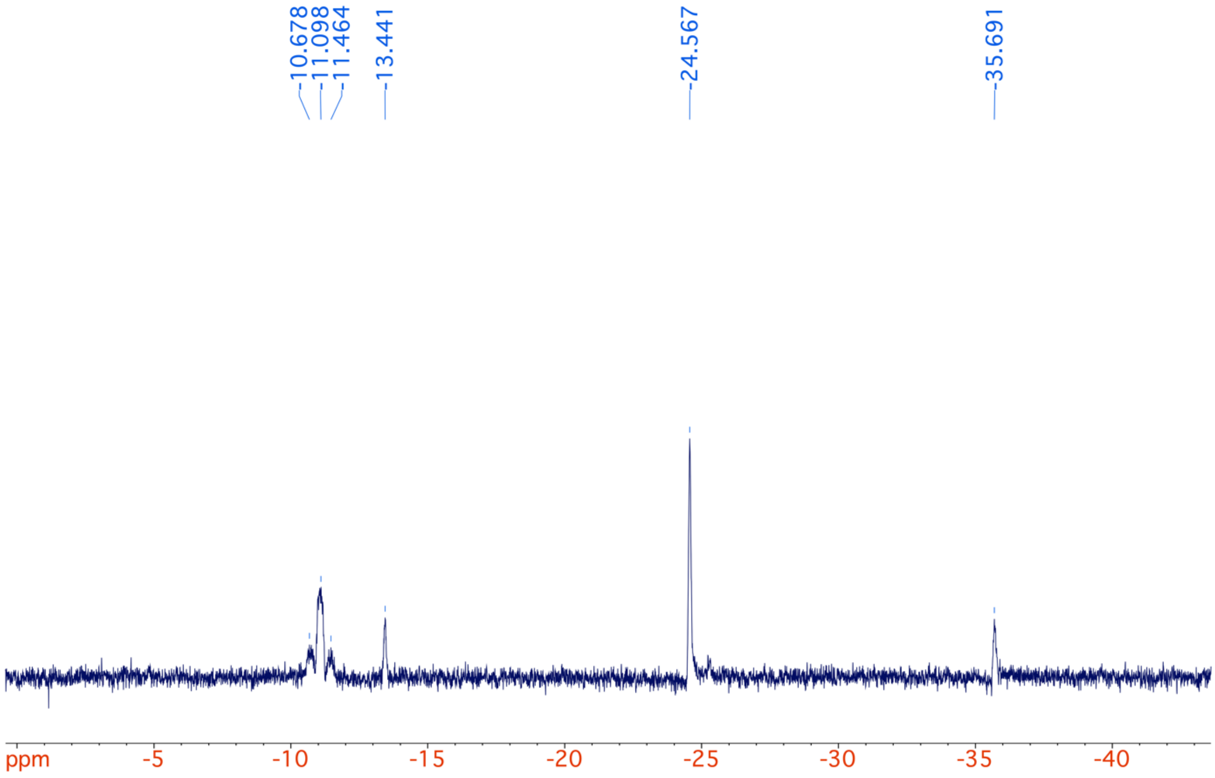

Figure S6. ^{31}P NMR spectrum in C_6D_6 of *trans*-[$(Me_3P)_2Pt(\kappa C$-2-$C_6F_4PPh_2)_2$] (**2Pt^a**).

Proportions are according to the ^{31}P NMR spectrum which shows the expected product in two isomeric forms (1:8 = isomer 1: isomer 2) in $CDCl_3$ solution of the crude reaction mixture.

^{31}P NMR ($CDCl_3$): -5.4 (br. m, $^3J_{Pt,P}$ = 160 Hz, 2P, PPh_2, isomer 2), -11.5 (m, $^3J_{Pt,P}$ = 90 Hz, 16P, PPh_2, isomer 1), -23.7 (m, $^1J_{Pt,P}$ = 2700 Hz, 18P, PMe_3, isomer 1), -24.4 (m, $^1J_{Pt,P}$ = 2700 Hz, 2P, PMe_3, isomer 2).

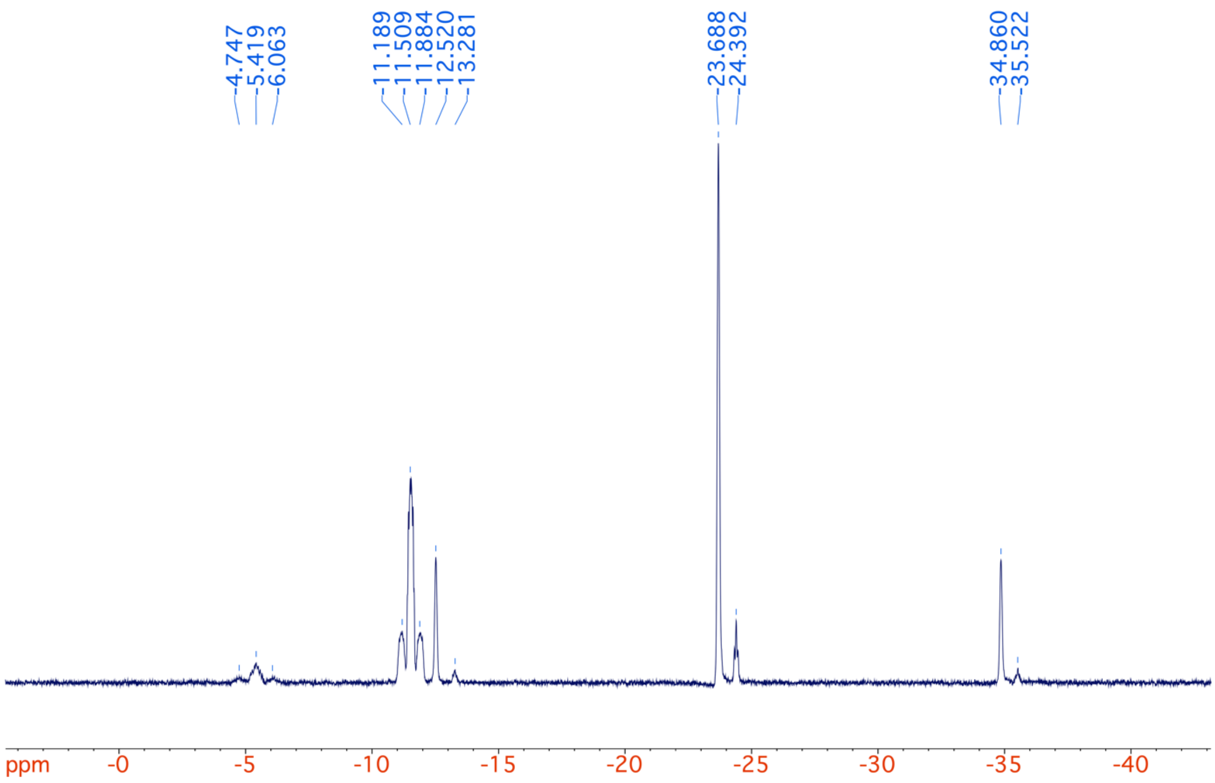

Figure S7. ^{31}P NMR spectrum in $CDCl_3$ of *trans*-[$(Me_3P)_2Pt(\kappa C$-2-$C_6F_4PPh_2)_2$] (**2Pt^a**).

[$(Me_3P)Ni(\kappa^2$-2-$C_6F_4PPh_2)_2$] (3Ni^a), [$(Me_3P)Ni(\kappa^2$-2-$C_6F_4PPh_2)(\kappa C$-2-$C_6F_4PPh_2)$] (3Ni^b), [$Ni_2(\kappa^2$-2-$C_6F_4PPh_2)_2(\kappa C$-2-$C_6F_4PPh_2)_2$] (4Ni). To a stirred solution of *trans*-[$Ni(\kappa^2$-2-$C_6F_4PPh_2)_2$] (230 mg, 317 µmol) in dichloromethane (15 mL) was added 1 M PMe_3-toluene-solution (317 µL). After stirring the deep red solution for 4 days at ambient temperature, methanol (10 mL) was added and the volume of the solution was reduced. The first fraction, a yellow precipitate of [$Ni_2(\kappa^2$-2-$C_6F_4PPh_2)_2(\mu$-2-$C_6F_4PPh_2)_2$], was filtered off, washed with methanol and dried at 40 °C *in vacuo* (Yield: 12 mg, 8 µmol, 5%). The volume of the solution was reduced further and a red solid precipitated out, which was filtered off, washed with methanol and dried at 40 °C *in vacuo* (Yield: 125 mg, 156 µmol, 49%).

First fraction: **[$Ni_2(\kappa^2$-2-$C_6F_4PPh_2)_2(\kappa C$-2-$C_6F_4PPh_2)_2$] (4Ni)**: ^{1}H NMR: 6.65 (br. m, 4H, aryl), 6.88 (br. m, 8H, aryl), 6.99-7.16 (br. m, 12H, aryl), 7.17-7.37 (br. m, 10H, aryl), 7.50 (br. m, 6H, aryl). ^{19}F NMR: -100.1 (m, 2F), -117.9 (m, 2F), -123.6 (m, 2F), -134.5 (m, 2F), -148.3 (m, 2F), -153.2 (m, 2F), -156.8 (m, 2F), -161.5 (m, 2F). ^{31}P NMR: 25.2 (m, 2P, µ-PPh_2), -55.3 (m, 2P, κ^2-PPh_2). Anal. Calcd. For $C_{72}H_{40}F_{16}P_4Ni_2\cdot1.7CH_2Cl_2$ (MW: 1594.7): C 55.51, H 2.74, F 19.06; found: C 55.99, H 2.99, F 18.55. ESI-MS (m/z): 1471.04 $[M+Na]^+$

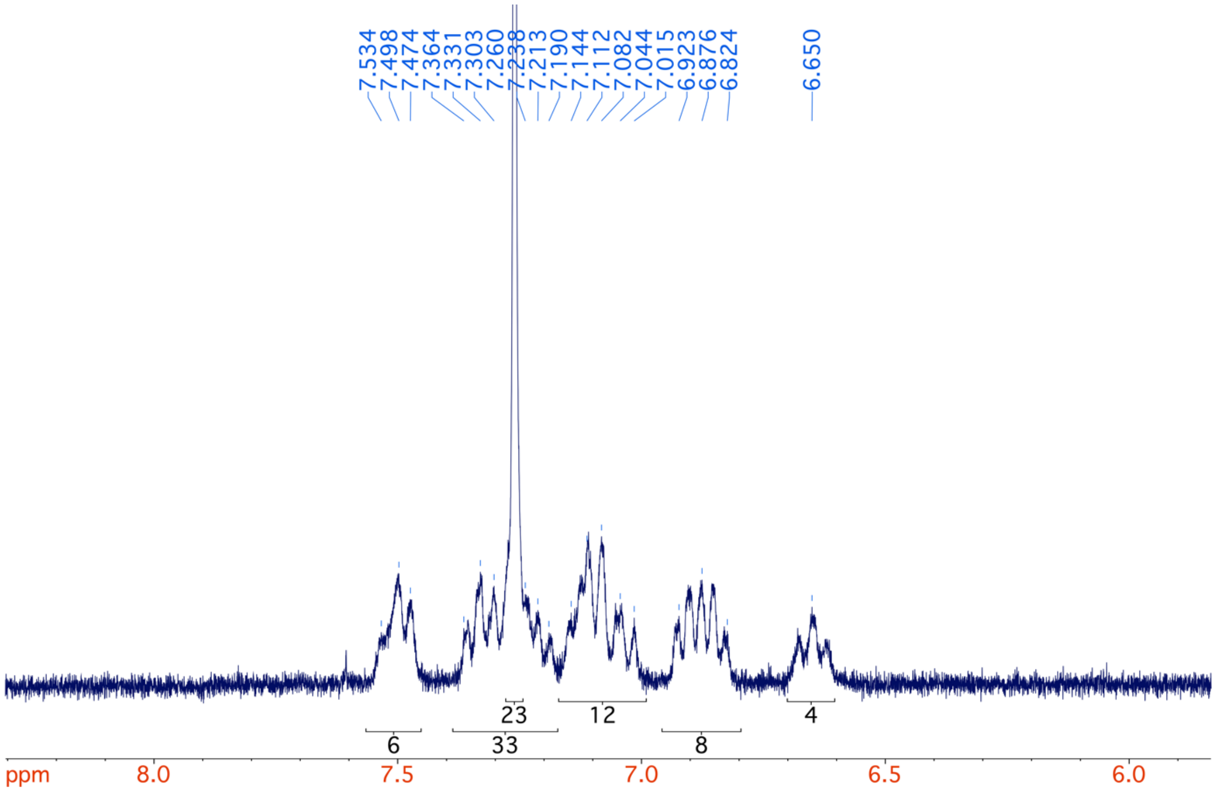

Figure S8. ¹H NMR spectrum in $CDCl_3$ of [Ni_2(κ^2-2-$C_6F_4PPh_2$)$_2$(κC-2-$C_6F_4PPh_2$)$_2$] (**4Ni**).

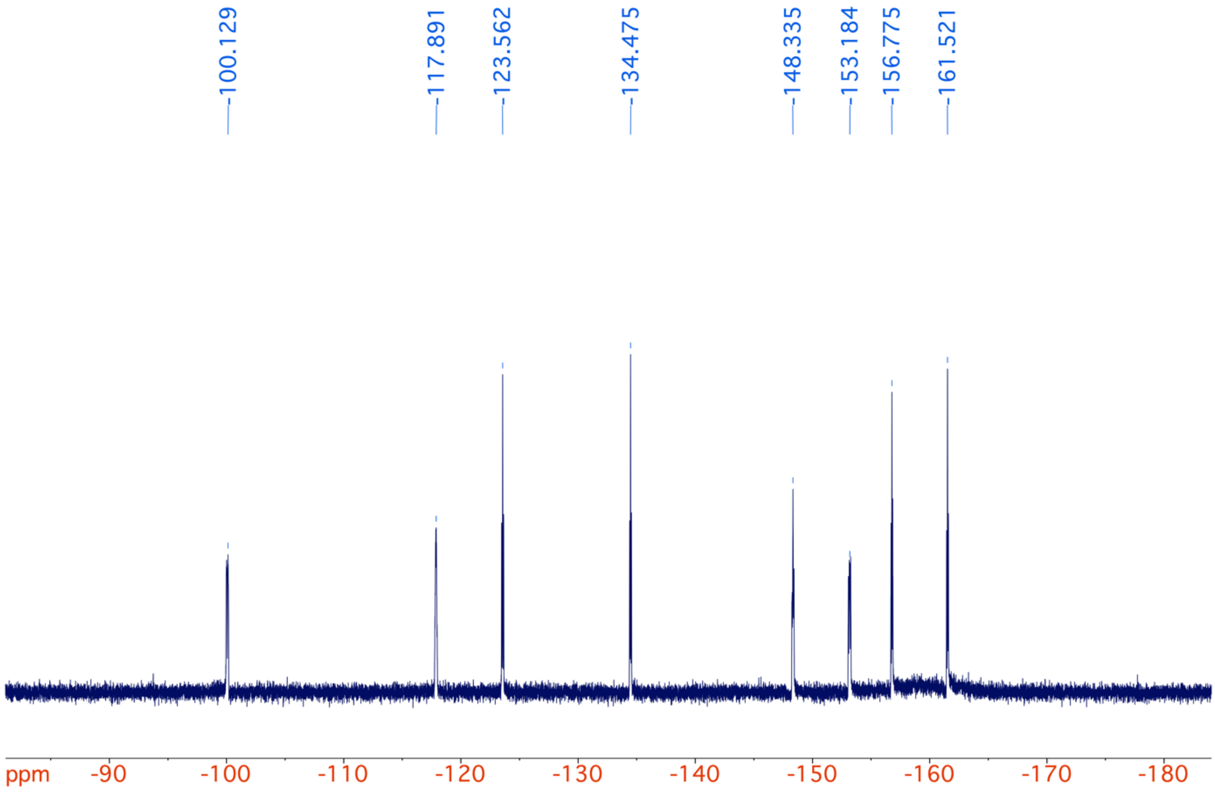

Figure S9. ¹⁹F NMR spectrum in $CDCl_3$ of [Ni_2(κ^2-2-$C_6F_4PPh_2$)$_2$(κC-2-$C_6F_4PPh_2$)$_2$] (**4Ni**).

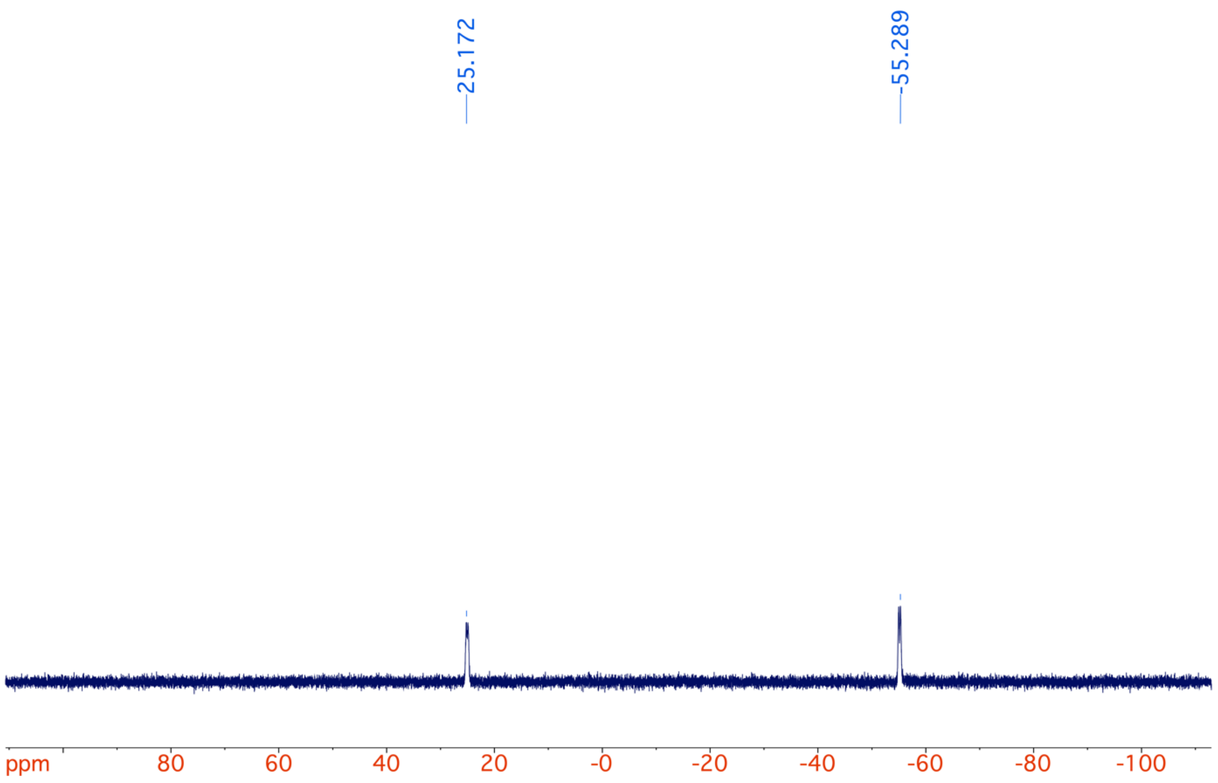

Figure S10. ^{31}P NMR spectrum in $CDCl_3$ of [$Ni_2(\kappa^2$-2-$C_6F_4PPh_2)_2(\kappa C$-2-$C_6F_4PPh_2)_2$] (**4Ni**).

Second fraction: **[(Me_3P)Ni(κ^2-2-$C_6F_4PPh_2)_2$]** (**3Ni^a**) and **[(Me_3P)Ni(κ^2-2-$C_6F_4PPh_2$)(2-$C_6F_4PPh_2$)]** (**3Ni^b**): mixture of two isomers (1:3 = **3Ni^a** : **3Ni^b**, from PMe_3 integrals in ^{1}H NMR spectrum), ^{1}H NMR (C_6D_6): 0.64 (d, $^1J_{P,H}$ = 8.53 Hz, 27H, PMe_3), 0.82 (d, $^1J_{P,H}$ = 8.74 Hz, 9H, PMe_3), 6.79-7.12 (br. m, 45H, aryl), 7.38-7.59 (br. m, 25H, aryl), 7.78 (br. m, 5H, aryl), 8.13 (br. m, 5H, aryl). ^{19}F NMR (C_6D_6): -119.4 (m, 3F, **3Ni^b**), -123.8 (br. m, 2F, **3Ni^a**), -126.1 (m, 3F, **3Ni^b**), -128.4 (m, 3F, **3Ni^b**), -132.6 (br. m, 1F, **3Ni^a**), -137.6 (m, 3F, **3Ni^b**), -149.6 (m, 3F, **3Ni^b**), -151.4 (br. m, 2F, **3Ni^a**), -155.4 (m, 3F, **3Ni^b**), -159.3 (m, 3F, **3Ni^b**), -160.3 (br. m, 2F, **3Ni^a**), -161.1 (m, 3F, **3Ni^b**). ^{31}P NMR (C_6D_6): -6.7 (m, -PPh_2, **3Ni^b**), -14.2 (dm, $^2J_{P,P}$ = 52 Hz, PMe_3, **3Ni^b**), -23.0 (br. t, $^2J_{P,P}$ ≈ 220 Hz, PMe_3, **3Ni^a**), -45.7 (dm, $^2J_{P,P}$ = 52 Hz, chelate P-Ni, **3Ni^b**), -52.7 (br. d, $^2J_{P,P}$ ≈ 220 Hz, chelate P-Ni, **3Ni^a**). Anal. Calcd. for $C_{39}H_{29}F_8P_3Ni$ (MW: 801.2): C 58.46, H 3.65, F 18.97; found: C 58.65, H 3.37, F 19.35. ESI-MS (m/z): 801.08 $[M+H]^+$

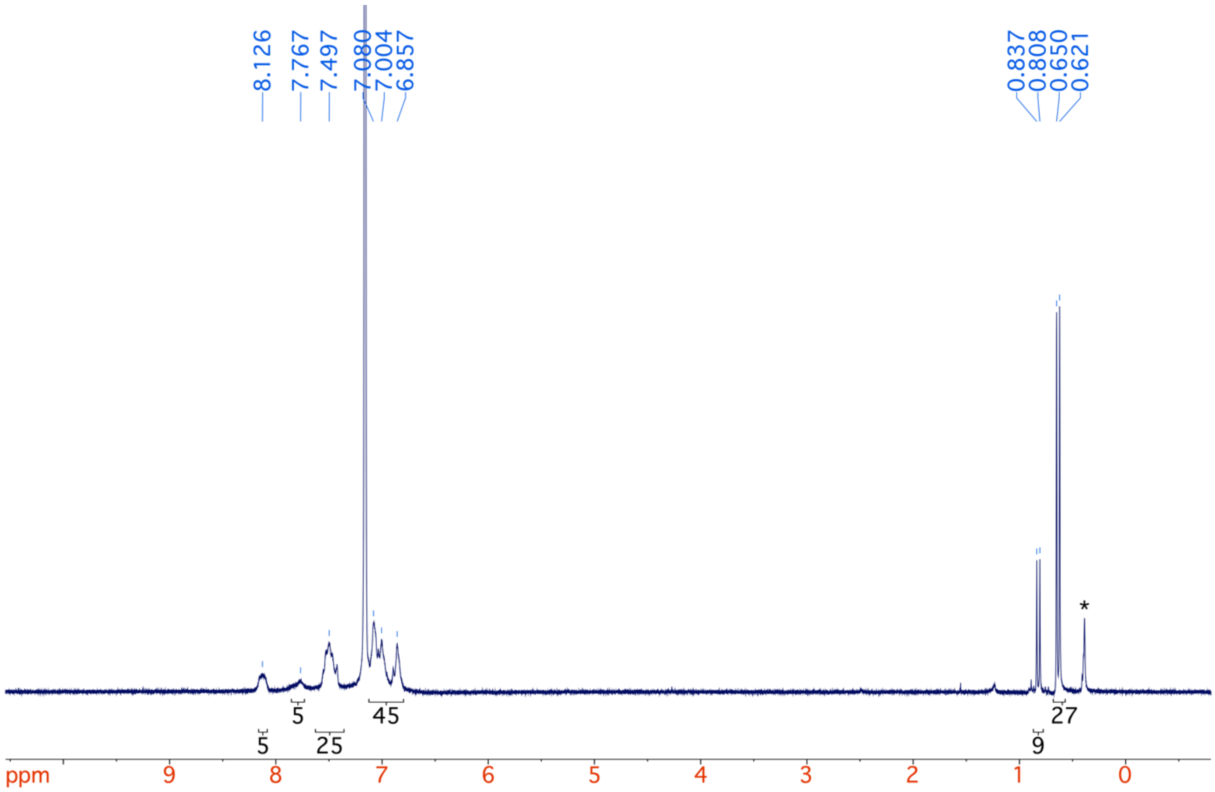

Figure S11. ^{1}H NMR spectrum in C_6D_6 of [(Me$_3$P)Ni(κ^2-2-$C_6F_4PPh_2$)$_2$] (**3Ni^a**) and [(Me$_3$P)Ni(κ^2-2-$C_6F_4PPh_2$)(2-$C_6F_4PPh_2$)] (**3Ni^b**). ^{1}H NMR signal of H_2O is marked with asterisk.

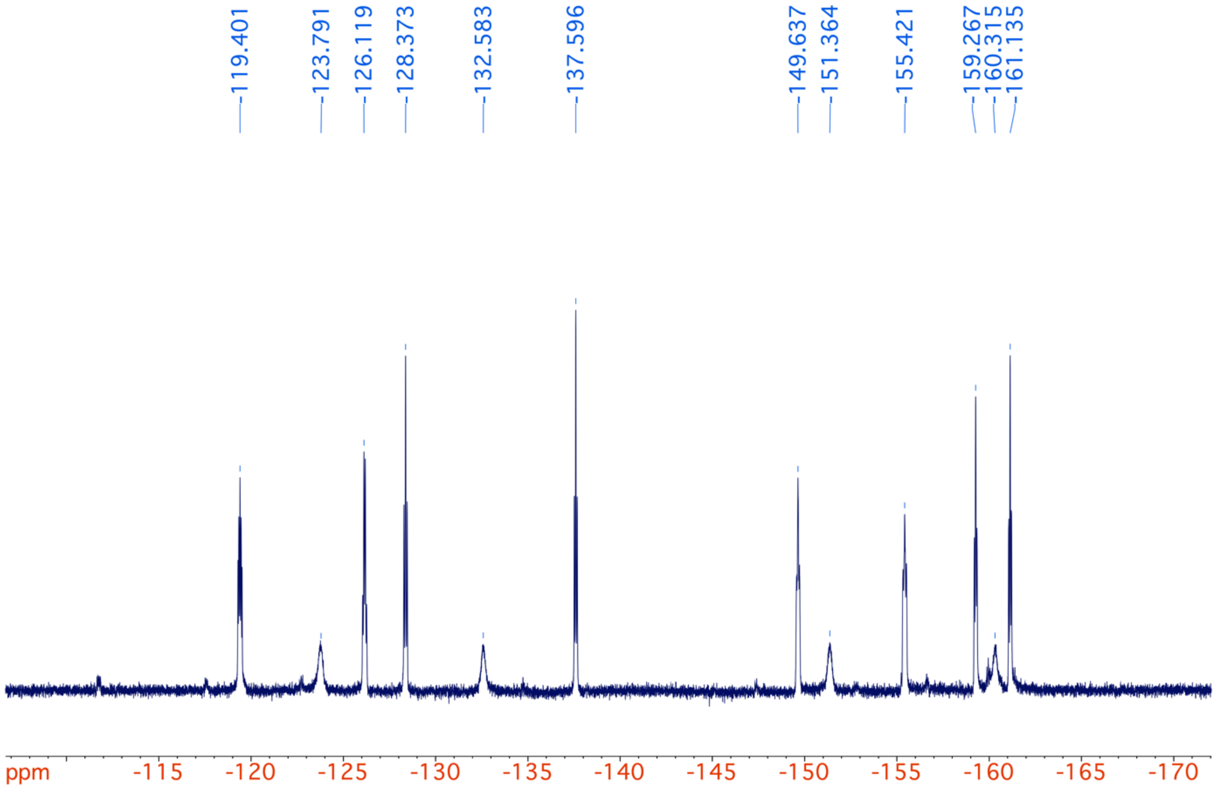

Figure S12. ^{19}F NMR spectrum in C_6D_6 of [(Me$_3$P)Ni(κ^2-2-$C_6F_4PPh_2$)$_2$] (**3Ni^a**) and [(Me$_3$P)Ni(κ^2-2-$C_6F_4PPh_2$)(2-$C_6F_4PPh_2$)] (**3Ni^b**).

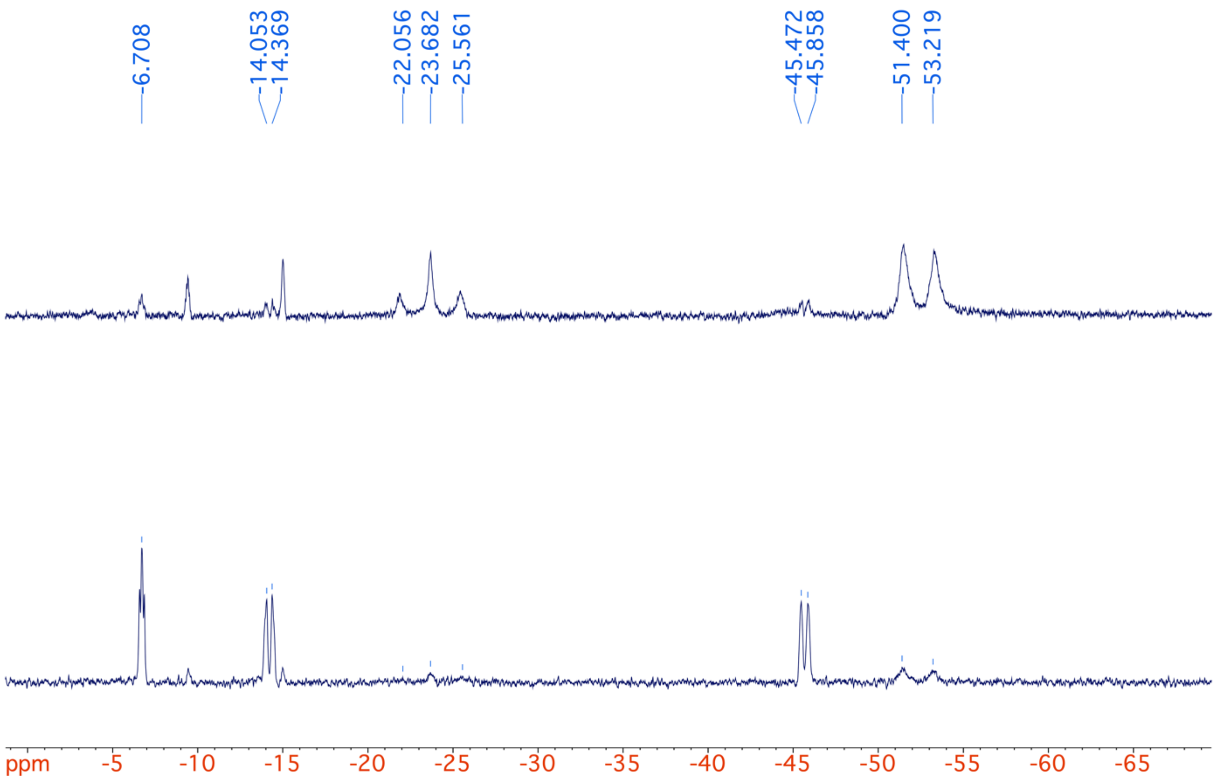

Figure S13. ^{31}P NMR spectra in C_6D_6 of [(Me$_3$P)Ni(κ^2-2-$C_6F_4PPh_2$)$_2$] (**3Ni^a**) and [(Me$_3$P)Ni(κ^2-2-$C_6F_4PPh_2$)(2-$C_6F_4PPh_2$)] (**3Ni^b**). Top: crude reaction mixture. Bottom: isolated second fraction.

[(Me$_3$P)Pt(κ^2-2-C$_6$F$_4$PPh$_2$)(μ-2-C$_6$F$_4$PPh$_2$)] (3Pt^b). To a stirred solution of *cis*-[Pt(*κ*^{2}-2-C$_6$F$_4$PPh$_2$)$_2$] (204 mg, 237μmol) in dichloromethane (15 mL) was added dropwise 1 M PMe3-toluene-solution (260 μL). The clear solution was stirred at ambient temperature for 15 min. After adding methanol (10 mL) the volume of the solution was reduced. The white solid was filtered off, washed with methanol and dried at 40 °C *in vacuo*. Yield: 168 mg (179 μmol, 76%).

^{1}H NMR: 1.28 (d, $^2J_{P,H}$ = 9.63 Hz, satellite: $^3J_{Pt,H}$ = 26.58 Hz, 9H, PMe$_3$), 7.03-7.19 (m, 3H, aryl), 7.19-7.33 (m, 7H, aryl), 7.43-7.60 (m, 6H, aryl), 7.66 (br. m, 2H, aryl), 7.98 (br. m, 2H, aryl). ^{19}F NMR: -117.5 (br. m, 1F), -125.7 (m, 1F), -127.0 (br. m, 1F), -137.7 (m, 1F), -149.8 (m, 1F), -155.6 (m, 1F), -160.4 (m, 1F), -161.4 (m, 1F). ^{31}P NMR: -6.8 (dm, $^3J_{F,P}$ = 22 Hz, $^3J_{Pt,P}$ = 207 Hz, 1P, κC-PPh$_2$), -29.3 (m, $^1J_{Pt,P}$ = 2350 Hz, 1P, PMe$_3$), -59.9 (m, $^1J_{Pt,P}$ = 1540 Hz, 1P, κ^2-PPh$_2$). Anal. Calcd. for C$_{39}$H$_{29}$F$_8$P$_3$Pt (MW: 937.6): C 49.96, H 3.12, F 16.21; found: C 50.20, H 3.06, F 16.19. ESI-MS (m/z): 938.11 [M+H]$^+$

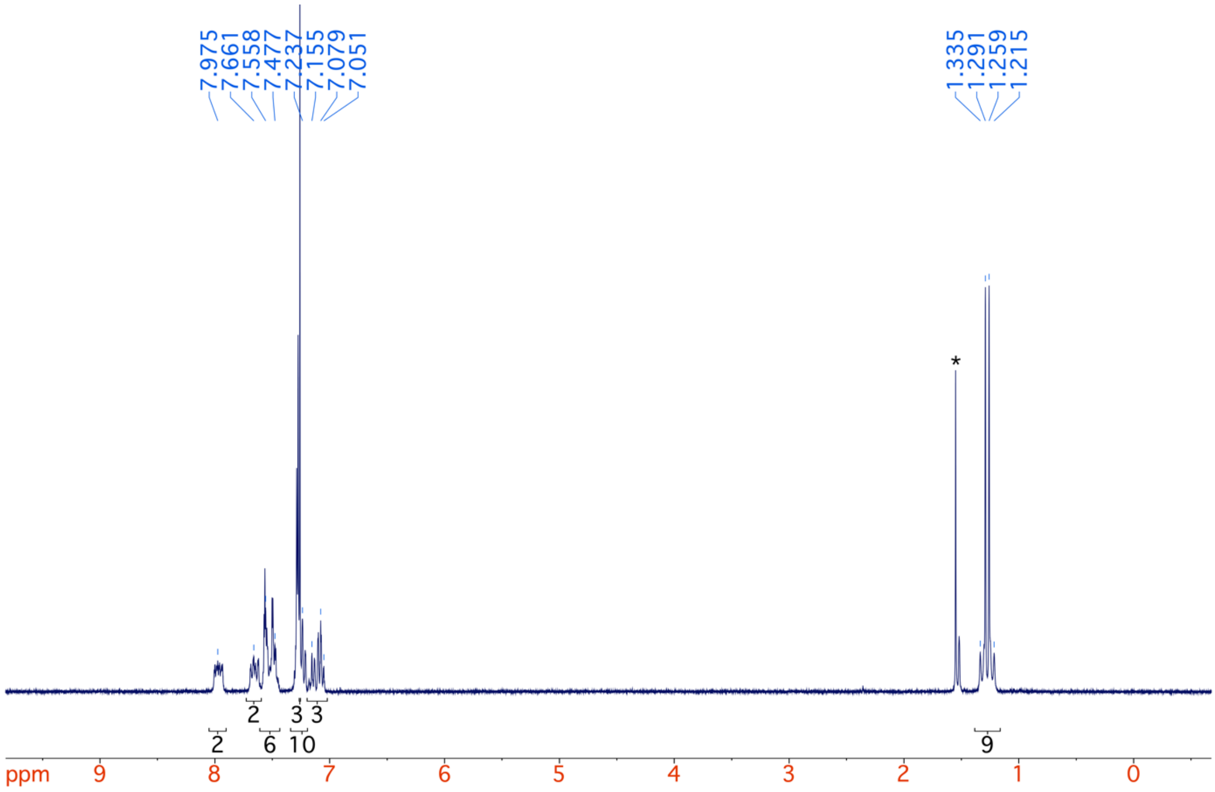

Figure S14. ^{1}H NMR spectrum in CDCl$_3$ of [(Me$_3$P)Pt(κ^2-2-C$_6$F$_4$PPh$_2$)(μ-2-C$_6$F$_4$PPh$_2$)] (**3Pt^b**). ^{1}H NMR signal of H$_2$O is marked with asterisk.

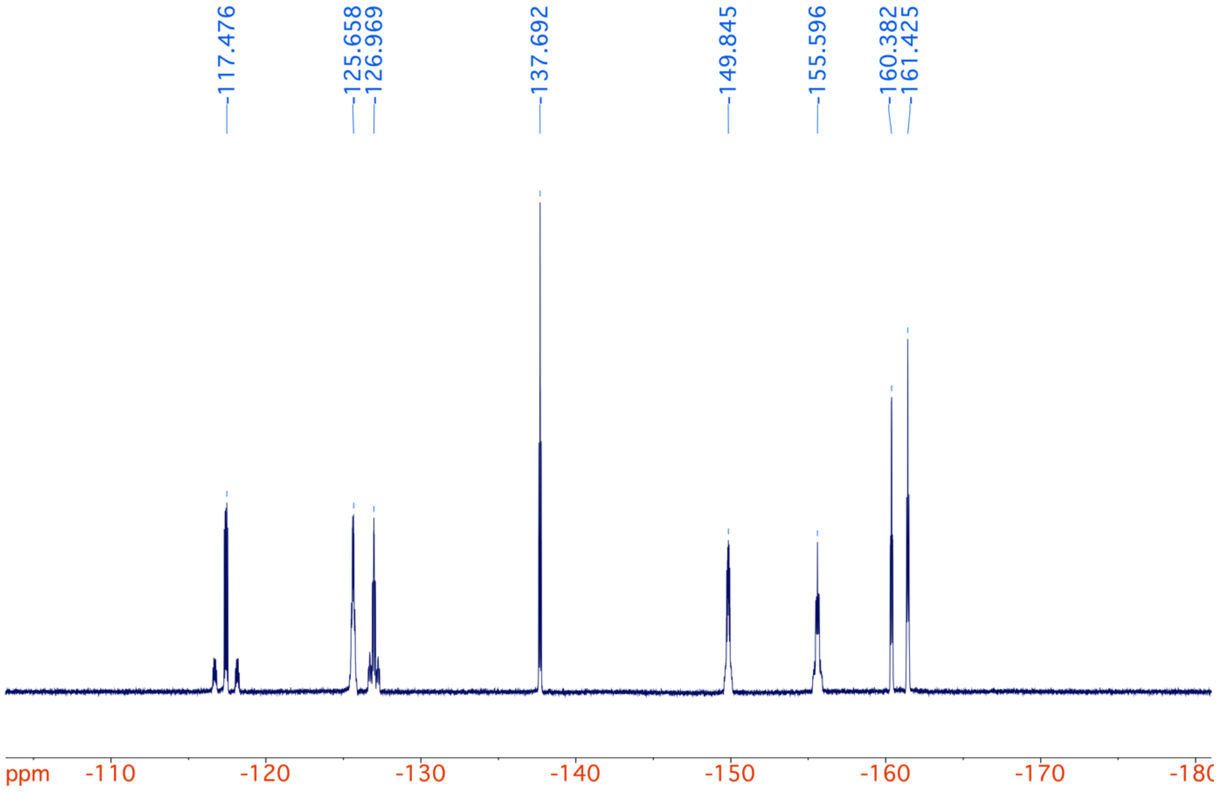

Figure S15. ^{19}F NMR spectrum in $CDCl_3$ of $[(Me_3P)Pt(\kappa^2\text{-}2\text{-}C_6F_4PPh_2)(\mu\text{-}2\text{-}C_6F_4PPh_2)]$ (**3Pt^b**).

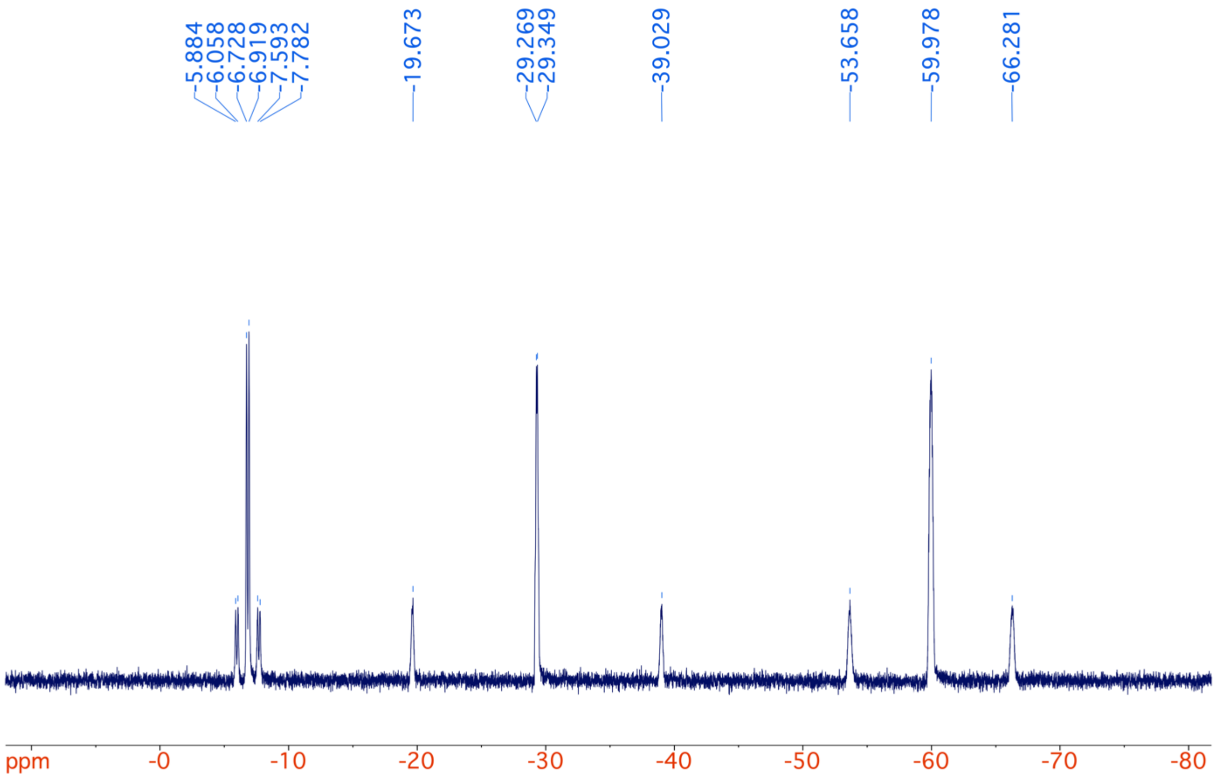

Figure S16. ^{31}P NMR spectrum in $CDCl_3$ of $[(Me_3P)Pt(\kappa^2\text{-}2\text{-}C_6F_4PPh_2)(\mu\text{-}2\text{-}C_6F_4PPh_2)]$ (**3Pt^b**).

[Pt_2(κ^2-2-$C_6F_4PPh_2$)$_2$(μ-2-$C_6F_4PPh_2$)$_2$] (4Pt). To a stirred solution of *trans*-[Pt(*κ*^{2}-2-$C_6F_4PPh_2$)$_2$] (92mg, 107μmol) in dichloromethane (15mL) was quickly added 1 M PMe_3-toluene-solution (300 μL). The clear solution was stirred at ambient temperature for 10 min. After adding methanol (10 mL) the volume of the solution was reduced. The white solid was filtered off, washed with methanol and dried at 40 °C *in vacuo* (m = 74 mg). NMR-data showed the product to be a mixture of [Pt_2(κ^2-2-$C_6F_4PPh_2$)$_2$(μ-2-$C_6F_4PPh_2$)$_2$] and *trans*-[(Me_3P)$_2$Pt(κC-2-$C_6F_4PPh_2$)$_2$], which could not be separated by recrystallization. The mixture (47 mg) was suspended with CuCl (16 mg, 162 μmol) in dichloromethane (10 mL) and stirred for 1 d. The suspension was filtered through celite, washed with dichloromethane and to the filtrate methanol (10 mL) was added. Fractional crystallisation under reduced pressure gave traces of [Pt_2(κ^2-2-$C_6F_4PPh_2$)$_2$(μ-2-$C_6F_4PPh_2$)$_2$], which could be isolated as the first fraction. The white solid was filtered off, washed with methanol and dried at 40 °C *in vacuo*. Yield: 9 mg (5 μmol, 5%).

^{1}H NMR: 6.83-7.15 (br. m, 18H, aryl), 7.17-7.41 (br. m, 17H, aryl), 7.47 (br, m, 5H, aryl). ^{19}F NMR: -103.4 (br. m, 2F), -118.3 (m, 2F), -125.6 (br. m, 2F), -135.2 (m, 2F), -148.5 (m, 2F), -151.9 (m, 2F), -158.5 (m, 2F), -161.5 (m, 2F). ^{31}P NMR: 15.7 (m, $^3J_{F,P}$ = 155 Hz, $^1J_{Pt,P}$ = 2375 Hz, 2P, μ-PPh_2), -66.0 (m, $^1J_{Pt,P}$ = 1690 Hz, 2P, κ^2-PPh_2). Anal. Calcd. for $C_{72}H_{40}F_{16}P_4Pt_2$ (MW: 1723.1): C 50.19, H 2.34, F 17.64; found: C 49.85, H 2.67, F 17.83. ESI-MS (m/z): 1745.10 $[M+Na]^+$

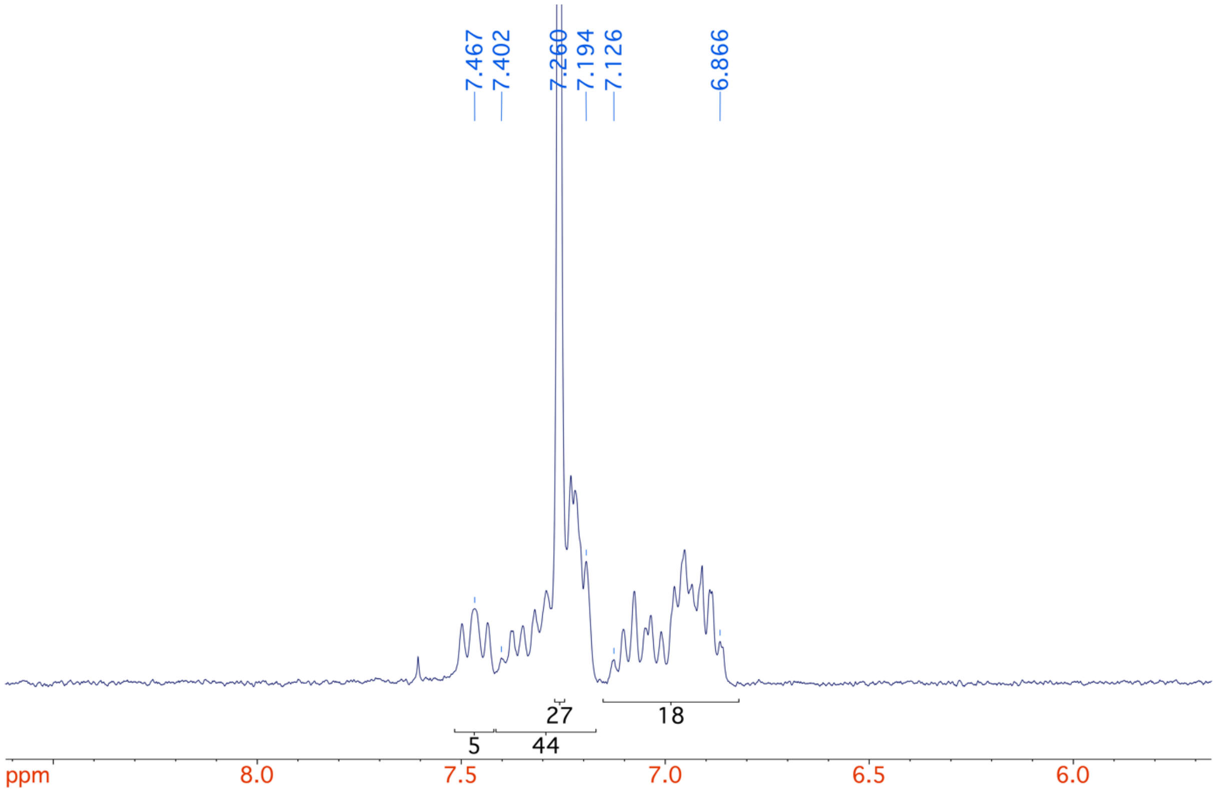

Figure S17. ^{1}H NMR spectrum in $CDCl_3$ of [Pt_2(κ^2-2-$C_6F_4PPh_2$)$_2$(μ-2-$C_6F_4PPh_2$)$_2$] (**4Pt**).

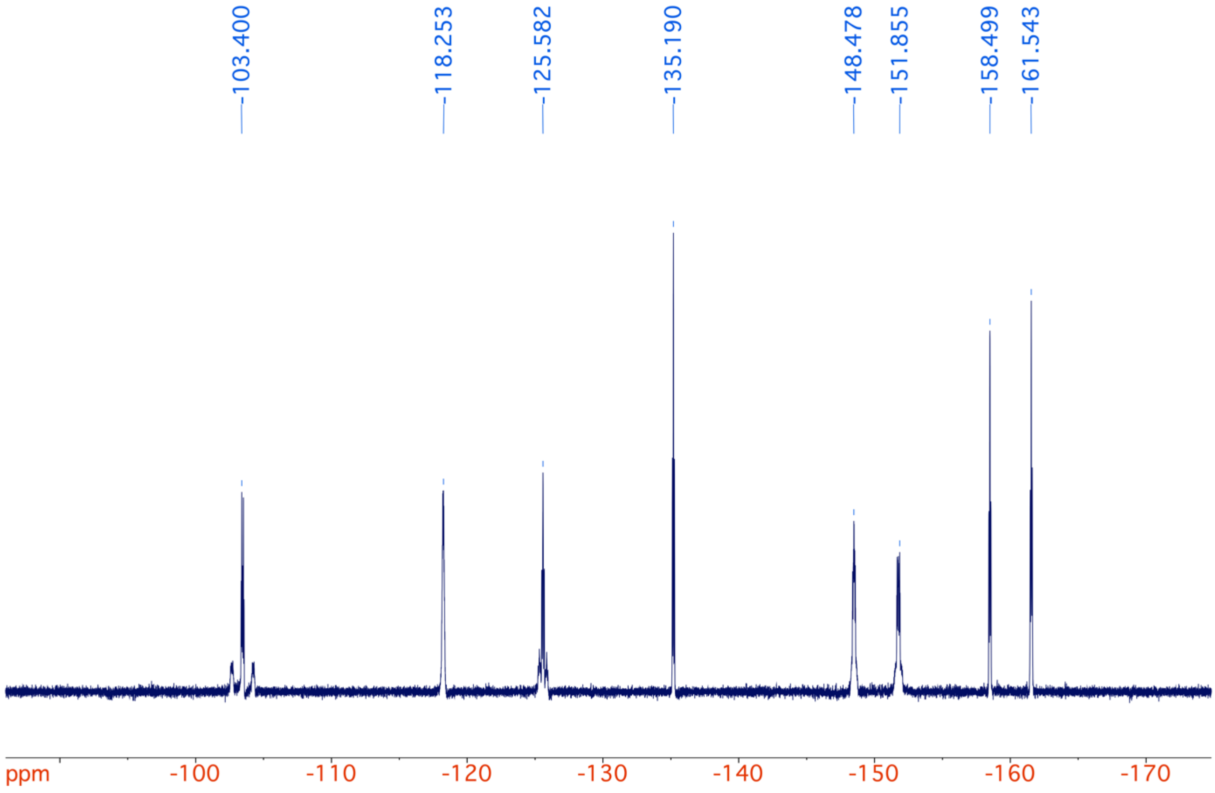

Figure S18. ^{19}F NMR spectrum in $CDCl_3$ of $[Pt_2(\kappa^2\text{-}2\text{-}C_6F_4PPh_2)_2(\mu\text{-}2\text{-}C_6F_4PPh_2)_2]$ (**4Pt**).

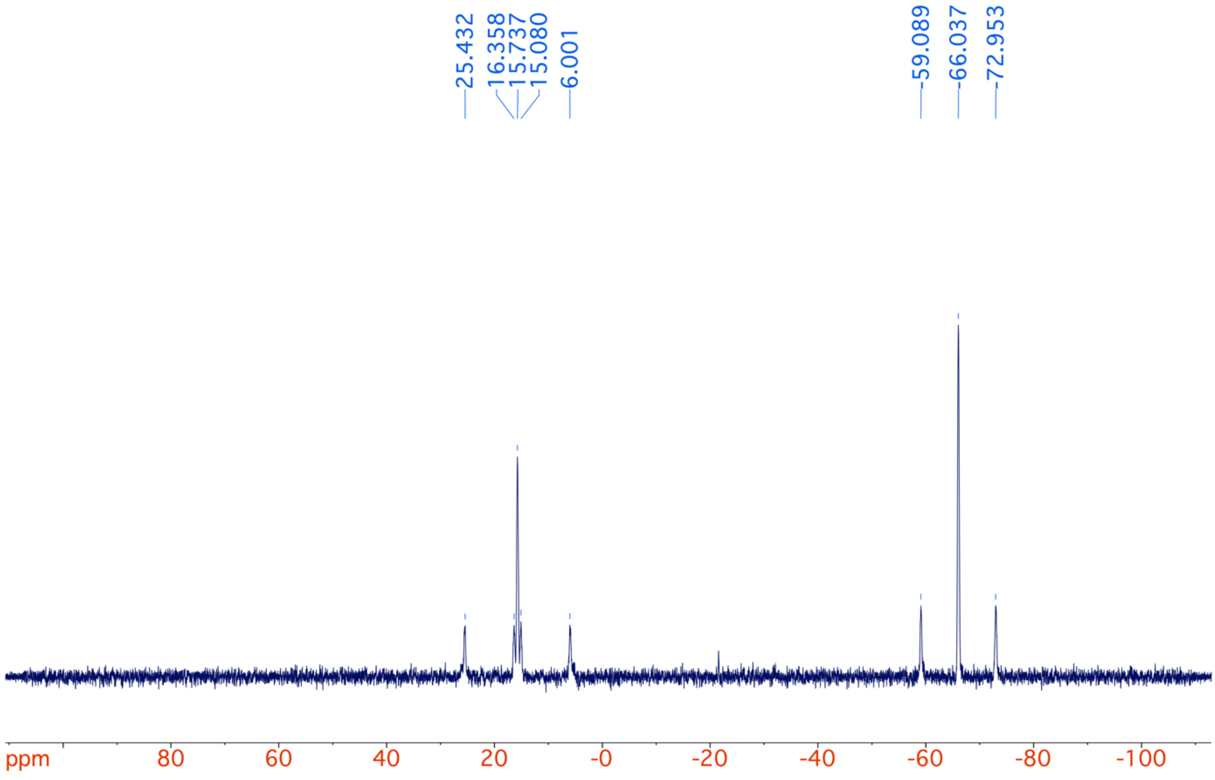

Figure S19. ^{31}P NMR spectrum in $CDCl_3$ of $[Pt_2(\kappa^2\text{-}2\text{-}C_6F_4PPh_2)_2(\mu\text{-}2\text{-}C_6F_4PPh_2)_2]$ (**4Pt**).

***trans*-[(Me$_3$P)$_2$Ni(μ-2-C$_6$F$_4$PPh$_2$)$_2$CuCl] (*trans*-5NiCu).** An orange suspension of *trans*-[(Me$_3$P)$_2$Ni(μ-2-C$_6$F$_4$PPh$_2$)$_2$] (90 mg, 103 µmol) and CuCl (10 mg, 101 µmol) in dichloromethane (15mL) was stirred at ambient temperature for 6 h. To the clear yellow solution methanol (10 mL) was added. The volume of the solution was reduced and the pale yellow solid was filtered off and washed with methanol. The solid was recrystallized from dichloromethane/hexane and dried at 40 °C *in vacuo*. Yield: 68 mg (70 µmol, 69%).

^{1}H NMR: 0.86 (t, $^2J_{P,H}$ = 3.88 Hz, 18H, PMe$_3$), 7.26-7.33 (m, 8H, aryl), 7.34-7.42 (m, 4H, aryl), 7.56 (br. m, 8H, aryl). ^{19}F NMR: -108.8 (m, 2F), -117.2 (m, 2F), -151.9 (m, 2F), -158.5 (m, 2F). ^{31}P NMR: 7.1 (m, 2P, P-Cu), -14.5 (s, 2P, P-Ni). Anal. Calcd. for C$_{42}$H$_{38}$CuClF$_8$P$_4$Ni (MW: 976.3): C 51.67, H 3.92, Cl 3.63, F 15.57; found: C 51.93, H 3.76, Cl 3.72, F 15.36. ESI-MS (m/z): 939.04 [M-Cl]$^+$

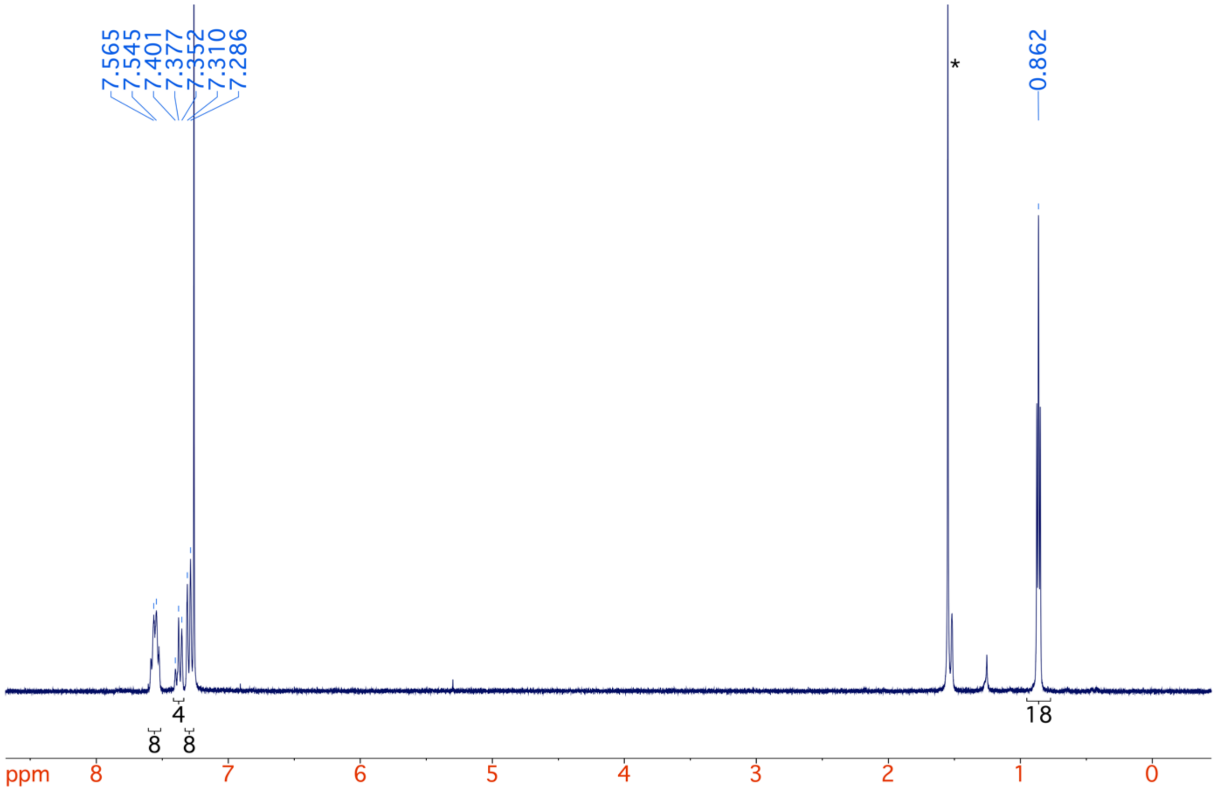

Figure S20. ^{1}H NMR spectrum in CDCl$_3$ of *trans*-[(Me$_3$P)$_2$Ni(μ-2-C$_6$F$_4$PPh$_2$)$_2$CuCl] (*trans*-**5NiCu**). ^{1}H NMR signal of H$_2$O is marked with asterisk.

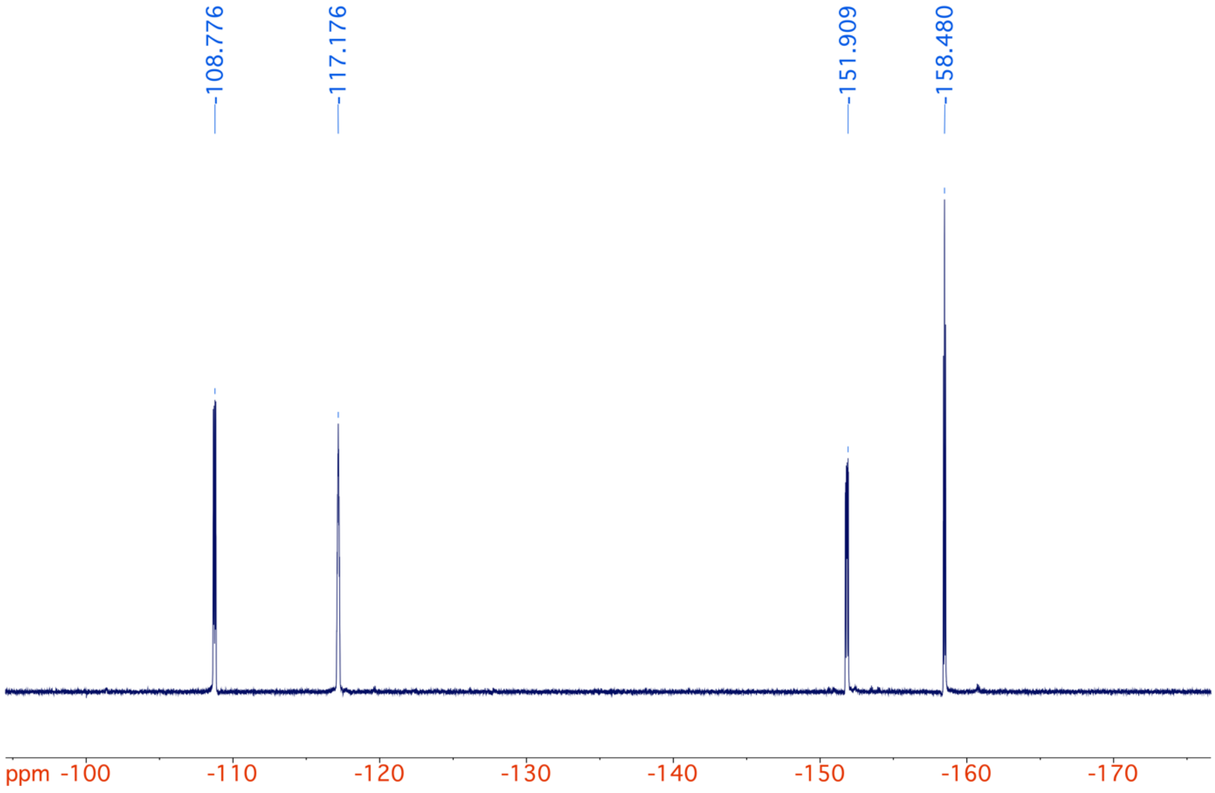

Figure S21. ^{19}F NMR spectrum in $CDCl_3$ of *trans*-[$(Me_3P)_2Ni(\mu$-2-$C_6F_4PPh_2)_2CuCl$] (*trans*-**5NiCu**).

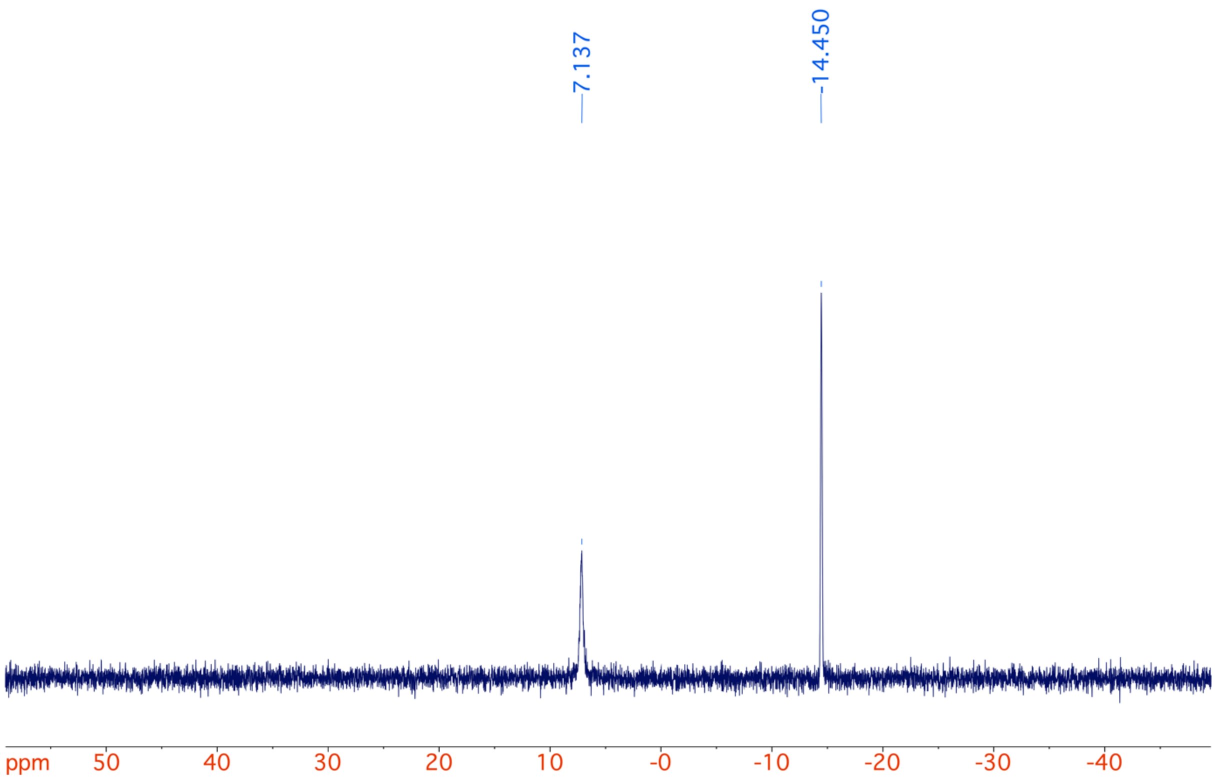

Figure S22. ^{31}P NMR spectrum in $CDCl_3$ of *trans*-[$(Me_3P)_2Ni(\mu$-2-$C_6F_4PPh_2)_2CuCl$] (*trans*-**5NiCu**).

***trans*-[($Me_3P)_2Ni(\mu$-2-$C_6F_4PPh_2)_2$AgCl] (*trans*-5NiAg).** A suspension of *trans*-[($Me_3P)_2Ni(\mu$-2-$C_6F_4PPh_2)_2$] (65 mg, 74 µmol) and AgCl (10 mg, 70 µmol) in dichloromethane (20 mL) was stirred at ambient temperature with light protection overnight. The yellow solid was filtered off, washed with dichloromethane and dried at 40 °C *in vacuo*. Yield: 41mg (40µmol, 57%).

^{1}H NMR: 0.79 (t, $^2J_{P,H}$ = 3.92 Hz, 18H, PMe_3), 7.31-7.45 (br. m, 12H, aryl), 7.62-7.75 (br. m, 8H, aryl). ^{19}F NMR: -107.2 (m, 2F), -117.7 (m, 2F), -151.5 (m, 2F), -157.9 (m, 2F). ^{31}P NMR: 15.9 (dm, $^1J_{AgP}$ = 460 Hz, 2P, P-Ag), -15.1 (m, 2P, P-Ni). Anal. Calcd. for $C_{42}H_{38}AgClF_8P_4Ni$ (MW: 1020.6): C 49.42, H 3.75, Cl 3.47, F 14.89; found: C 49.46, H 3.62, Cl 3.23, F 14.48. ESI-MS (m/z): 1042.98 $[M+Na]^+$

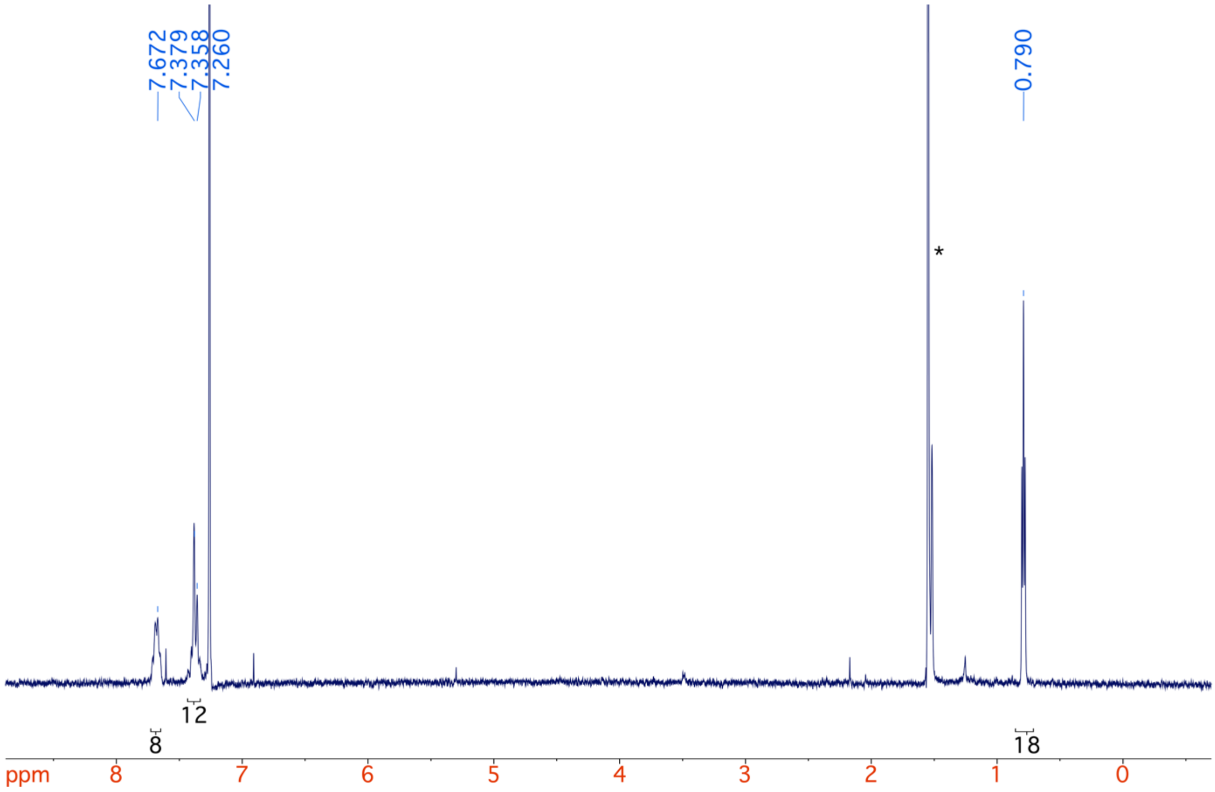

Figure S23. ^{1}H NMR spectrum in $CDCl_3$ of *trans*-[($Me_3P)_2Ni(\mu$-2-$C_6F_4PPh_2)_2$AgCl] (*trans*-**5NiAg**). ^{1}H NMR signal of H_2O is marked with asterisk.

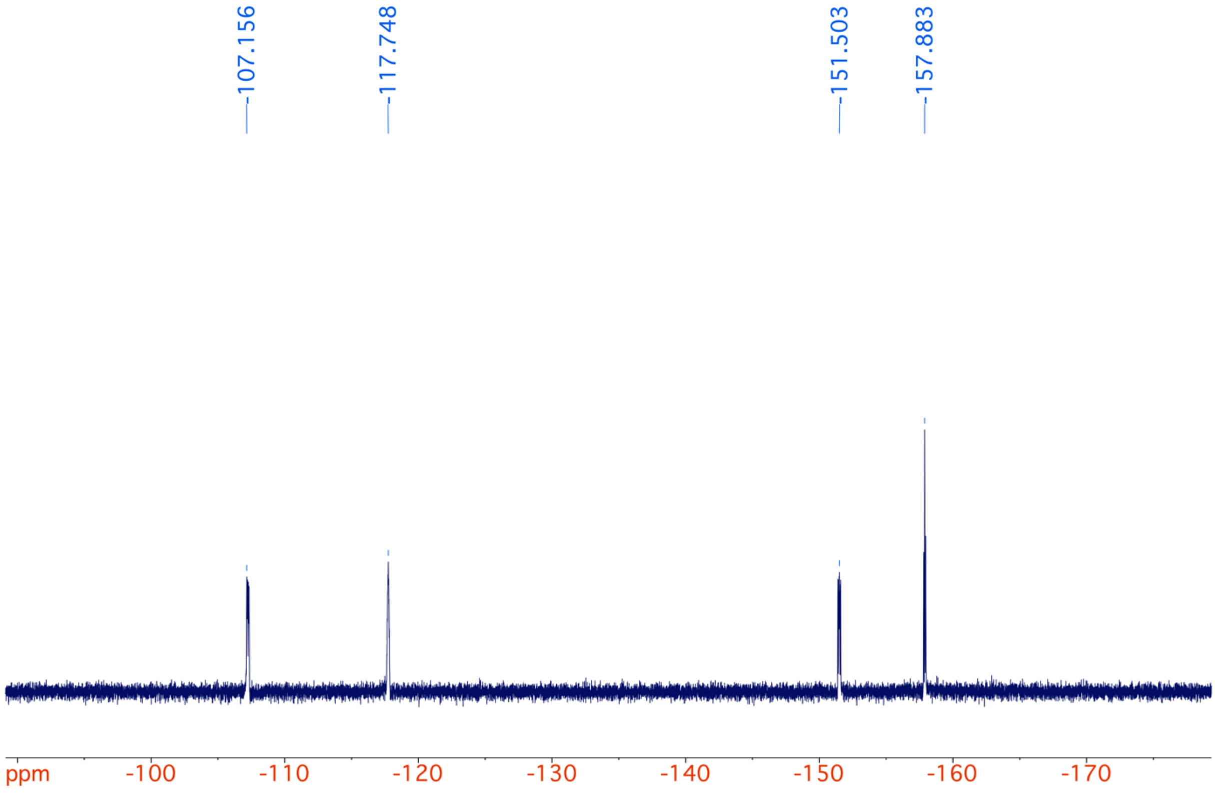

Figure S24. ^{19}F NMR spectrum in $CDCl_3$ of *trans*-[$(Me_3P)_2Ni(\mu$-2-$C_6F_4PPh_2)_2AgCl$] (*trans*-**5NiAg**).

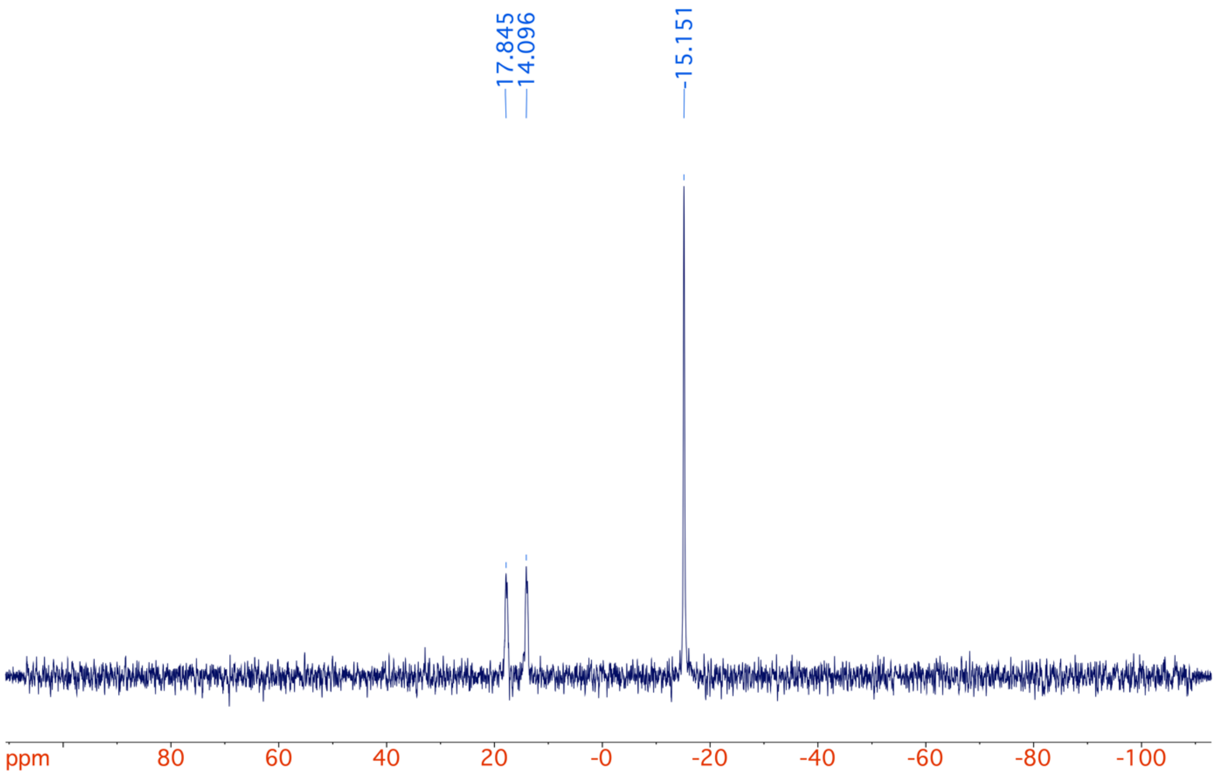

Figure S25. ^{31}P NMR spectrum in $CDCl_3$ of *trans*-[$(Me_3P)_2Ni(\mu$-2-$C_6F_4PPh_2)_2AgCl$] (*trans*-**5NiAg**).

***trans*-[(Me$_3$P)$_2$Ni(μ-2-C$_6$F$_4$PPh$_2$)$_2$AuCl] (*trans*-5NiAu).** To a stirred solution of *trans*-[(Me$_3$P)$_2$Ni(μ-2-C$_6$F$_4$PPh$_2$)$_2$] (100 mg, 114 µmol) in dichloromethane (15 mL) cooled in a NaCl-ice bath was added solid [AuCl(tht)] (36.5 mg, 114 µmol). As soon as a clear solution was obtained, *n*-hexane (10 mL) was added and the volume of the deep red solution was reduced. The yellow solid was filtered off, washed with *n*-hexane and dried at 40 °C *in vacuo*. Yield: 52 mg (45 µmol, 39%).

^{1}H NMR (CD$_2$Cl$_2$): 0.74 (t, $^2J_{P,H}$ = 3.73 Hz, 18H, PMe$_3$), 7.38-7.54 (br. m, 12H, aryl), 7.77-7.90 (br. m, 8H, aryl). ^{19}F NMR (CD$_2$Cl$_2$): -105.2 (m, 2F), -118.3 (m, 2F), -151.1 (m, 2F), -158.9 (m, 2F). ^{31}P NMR (CD$_2$Cl$_2$): 41.9 (m, 2P, P-Au), -15.9 (m, 2P, P-Ni). Anal. Calcd. for C$_{42}$H$_{38}$AuClF$_8$P$_4$Ni·0.2*n*-hexane (MW: 1149.6): C 45.14, H 3.54, Cl 3.08, F 13.22; found: C 45.18, H 3.49, Cl 3.06, F 13.68. ESI-MS (m/z): 1073.08 [M-Cl]$^+$

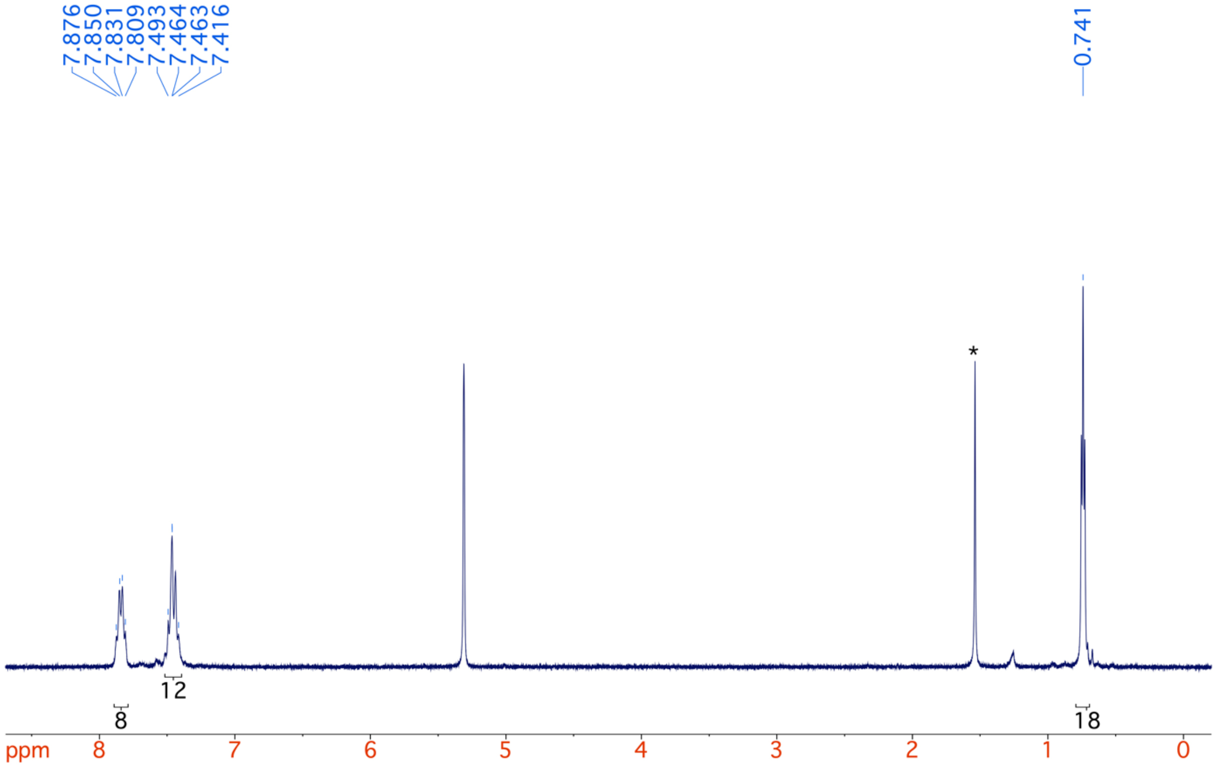

Figure S26. ^{1}H NMR spectrum in CD$_2$Cl$_2$ of *trans*-[(Me$_3$P)$_2$Ni(μ-2-C$_6$F$_4$PPh$_2$)$_2$AuCl] (*trans*-**5NiAu**). ^{1}H NMR signal of H$_2$O is marked with asterisk.

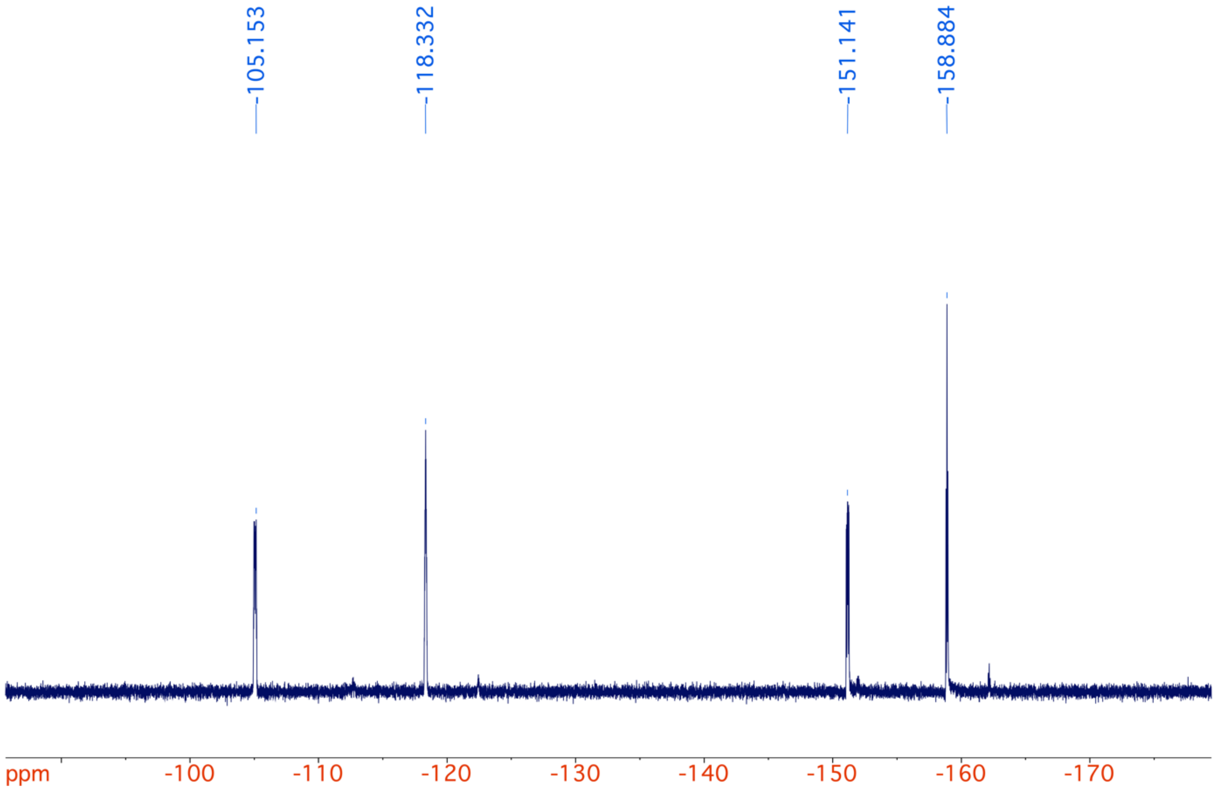

Figure S27. ^{19}F NMR spectrum in CD_2Cl_2 of *trans*-[$(Me_3P)_2Ni(\mu$-2-$C_6F_4PPh_2)_2AuCl$] (*trans*-**5NiAu**).

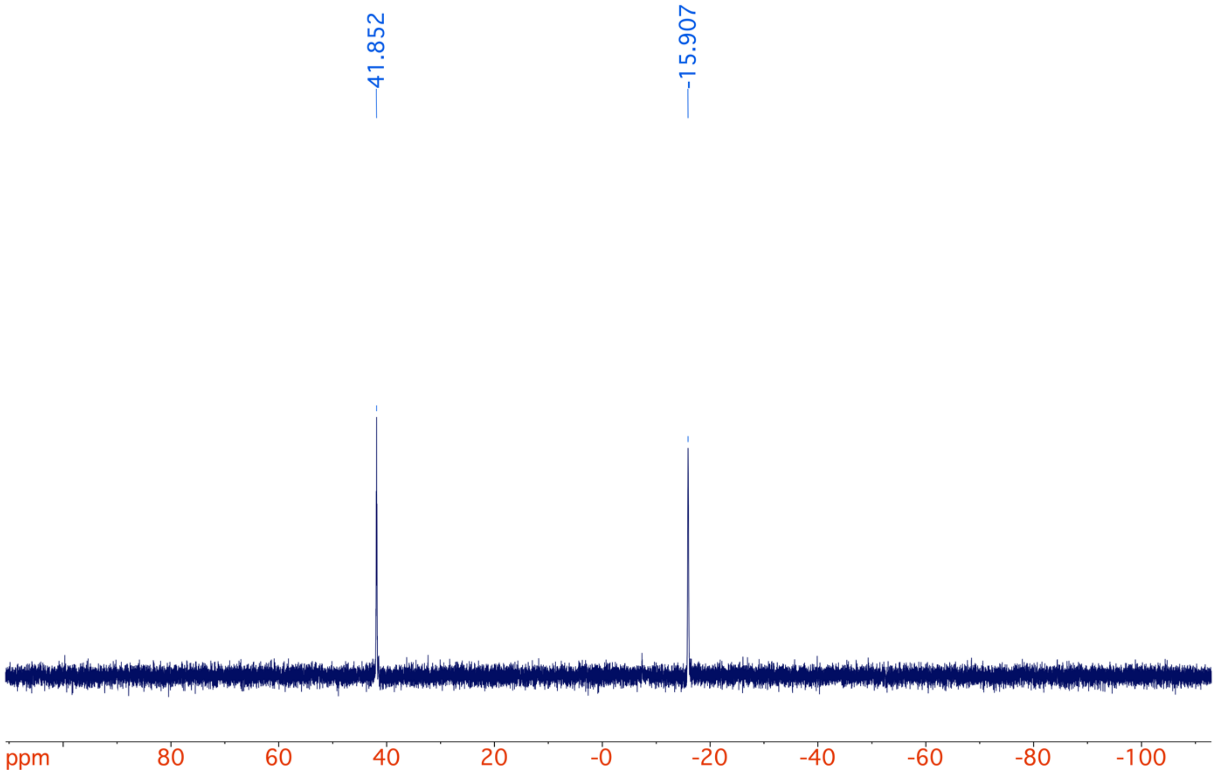

Figure S28. ^{31}P NMR spectrum in CD_2Cl_2 of *trans*-[$(Me_3P)_2Ni(\mu$-2-$C_6F_4PPh_2)_2AuCl$] (*trans*-**5NiAu**).

***trans*-[(Me$_3$P)$_2$Pt(µ-2-C$_6$F$_4$PPh$_2$)$_2$CuCl] (*trans*-5PtCu).** To a stirred solution of *trans*-[(Me$_3$P)$_2$Pt(μ-2-C$_6$F$_4$PPh$_2$)$_2$] (100 mg, 99 µmol) in dichloromethane (15 mL) was added solid CuCl (12 mg, 121 µmol). The suspension was stirred at ambient temperature overnight. The mixture was filtered through Celite, and methanol (10 mL) was added to the filtrate. The volume of the solution was reduced and the white/greenish solid was filtered off, washed with methanol and dried at 40 °C *in vacuo*. Yield: 69 mg (62 µmol, 63%).

^{1}H NMR: 1.10 (t, $^2J_{P,H}$ = 3.84 Hz, $^3J_{Pt,H}$ = 30.86 Hz, 18H, PMe$_3$), 7.21-7.31 (br. m, 8H, aryl), 7.31-7.40 (br. m, 4H, aryl), 7.44-7.54 (br. m, 8H, aryl). ^{19}F NMR: -111.0 (br. m, 2F), -118.3 (m, 2F), -151.2 (m, 2F), -159.2 (m, 2F). ^{31}P NMR: -0.9 (m, $^3J_{Pt,P}$ = 160 Hz, 2P, P-Cu), -21.7 (m, $^3J_{Pt,P}$ = 2565 Hz, 2P, P-Pt). Anal. Calcd. for $C_{42}H_{38}CuClF_8P_4Pt$ (MW: 1112.7): C 45.34, H 3.44, Cl 3.19, F 13.66; found: C 45.64, H 3.48, Cl 3.25, F 13.67. ESI-MS (m/z): 1077.08 [M-Cl+H]$^+$

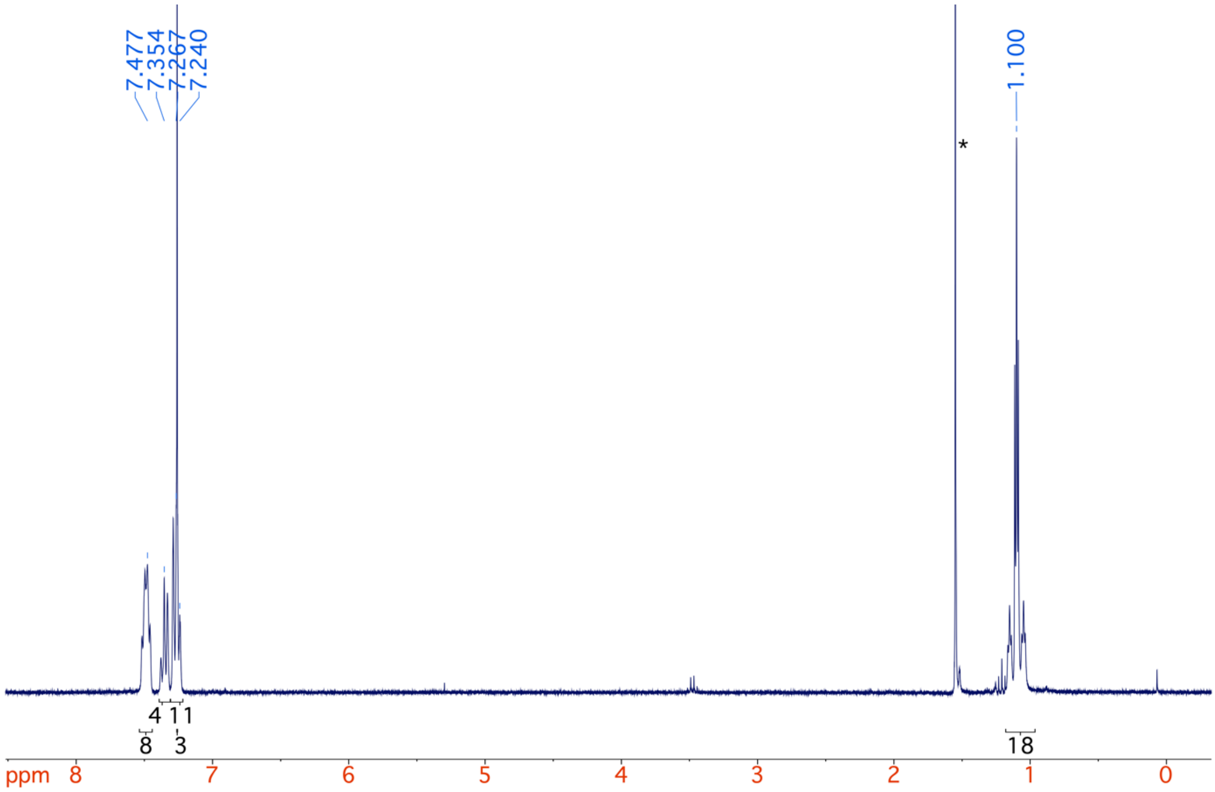

Figure S29. ^{1}H NMR spectrum in $CDCl_3$ of *trans*-[(Me$_3$P)$_2$Pt(μ-2-C$_6$F$_4$PPh$_2$)$_2$CuCl] (*trans*-**5PtCu**). ^{1}H NMR signal of H_2O is marked with asterisk.

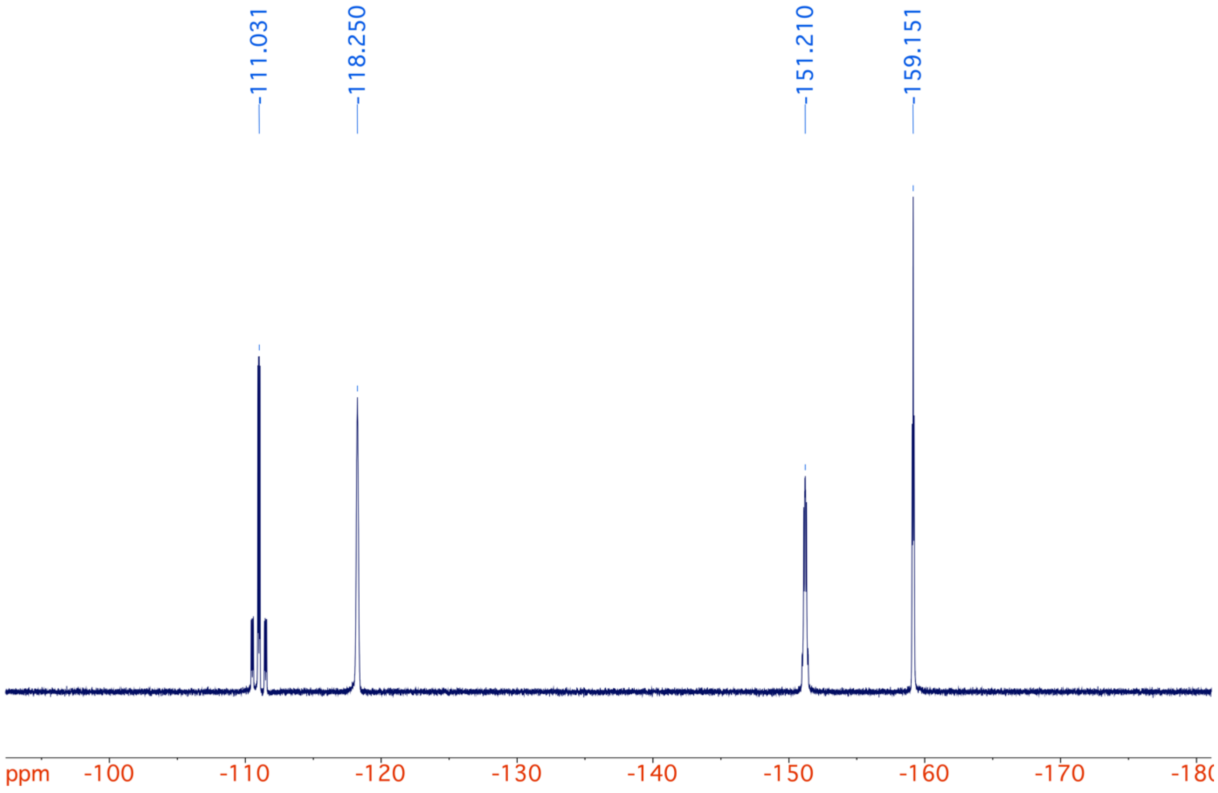

Figure S30. ^{19}F NMR spectrum in $CDCl_3$ of *trans*-[$(Me_3P)_2Pt(\mu$-2-$C_6F_4PPh_2)_2CuCl$] (*trans*-**5PtCu**).

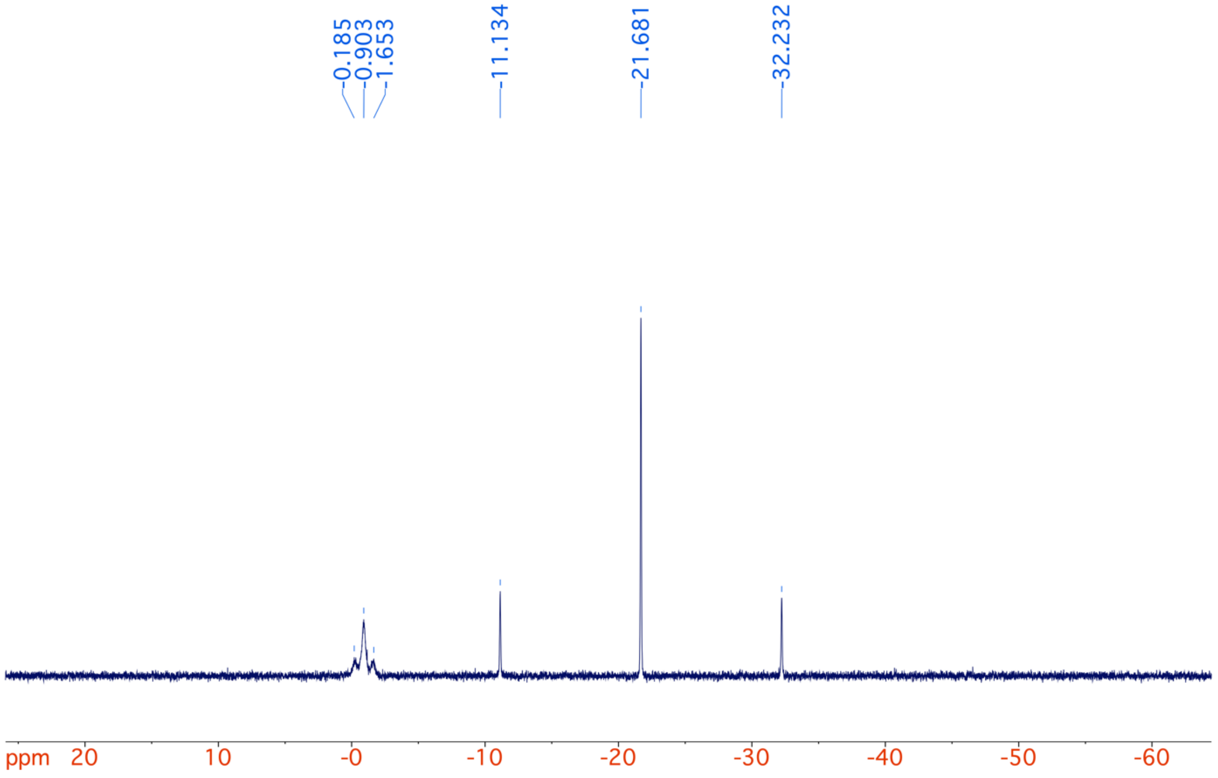

Figure S31. ^{31}P NMR spectrum in $CDCl_3$ of *trans*-[$(Me_3P)_2Pt(\mu$-2-$C_6F_4PPh_2)_2CuCl$] (*trans*-**5PtCu**).

***trans*-[(Me$_3$P)$_2$Pt(μ-2-C$_6$F$_4$PPh$_2$)$_2$AgCl] (*trans*-5PtAg).** To a stirred solution of *trans*-[Pt(κ^2-2-C$_6$F$_4$PPh$_2$)$_2$] (237 mg, 275 μmol) in dichloromethane (40 mL) was added dropwise 1 M PMe$_3$-toluene-solution (1.5 mL). The clear solution was stirred at ambient temperature for 5 min. After adding methanol (10 mL) the volume of the solution was reduced. The white solid was filtered off, washed with methanol and air dried. 120 mg of this 1:5 mixture of [Pt$_2$(κ^2-2-C$_6$F$_4$PPh$_2$)$_2$(μ-2-C$_6$F$_4$PPh$_2$)$_2$] and *trans*-[(Me$_3$P)$_2$Pt(μ-2-C$_6$F$_4$PPh$_2$)$_2$] (99 μmol) was dissolved in dichloromethane (15 mL) and solid AgCl (12 mg, 84 μmol) was added. The mixture was stirred in the dark at room temperature overnight. The white solid product was filtered off, washed with dichloromethane and dried at 40 °C *in vacuo*. Yield: 65 mg (56 μmol, 67%).

^{1}H NMR (CD$_2$Cl$_2$): 1.01 (t, $^2J_{P,H}$ = 3.81 Hz, $^3J_{Pt,H}$ = 30.19 Hz, 18H, PMe$_3$), 7.29-7.47 (br. m, 12H, aryl), 7.50-7.61 (m, 8H, aryl). ^{19}F NMR (CD$_2$Cl$_2$): -109.0 (br. m, 2F), -119.5 (m, 2F), -151.9 (m, 2F), -160.0 (m, 2F). ^{31}P NMR (CD$_2$Cl$_2$): 6.4 (ddm, $^1J_{(109)Ag,P}$ = 470 Hz, $^1J_{(107)Ag,P}$ = 400 Hz, $^3J_{Pt,P}$ = 220 Hz, 2P, PAg), -24.1 (m, $^1J_{Pt,P}$ = 2535 Hz, 2P, P-Pt). Anal. Calcd. for C$_{42}$H$_{38}$AgClF$_8$P$_4$Pt (MW: 1157.0): C 43.60, H 3.31, F 13.14; found: C 43.93, H 3.16, F 13.50. ESI-MS (m/z): 1121.06 [M-Cl+H]$^+$

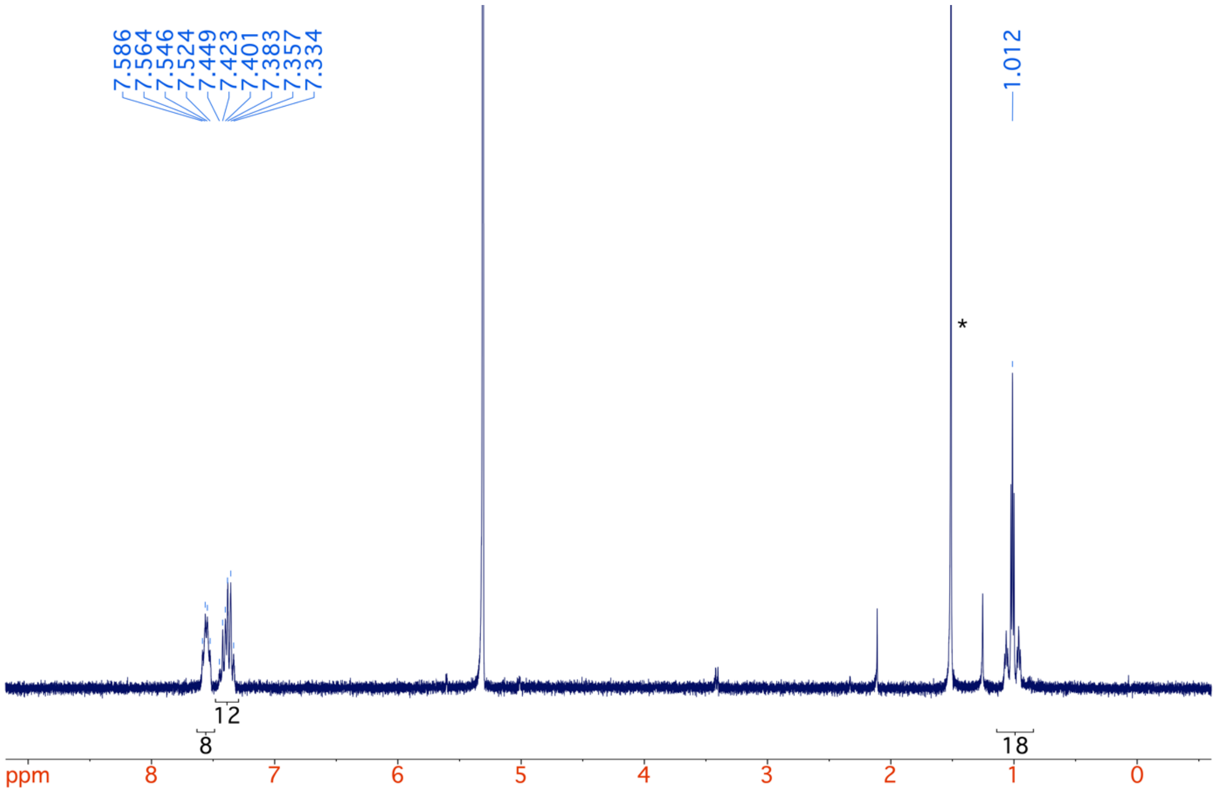

Figure S32. ^{1}H NMR spectrum in CD$_2$Cl$_2$ of *trans*-[(Me$_3$P)$_2$Pt(μ-2-C$_6$F$_4$PPh$_2$)$_2$AgCl] (*trans*-**5PtAg**). ^{1}H NMR signal of H$_2$O is marked with asterisk.

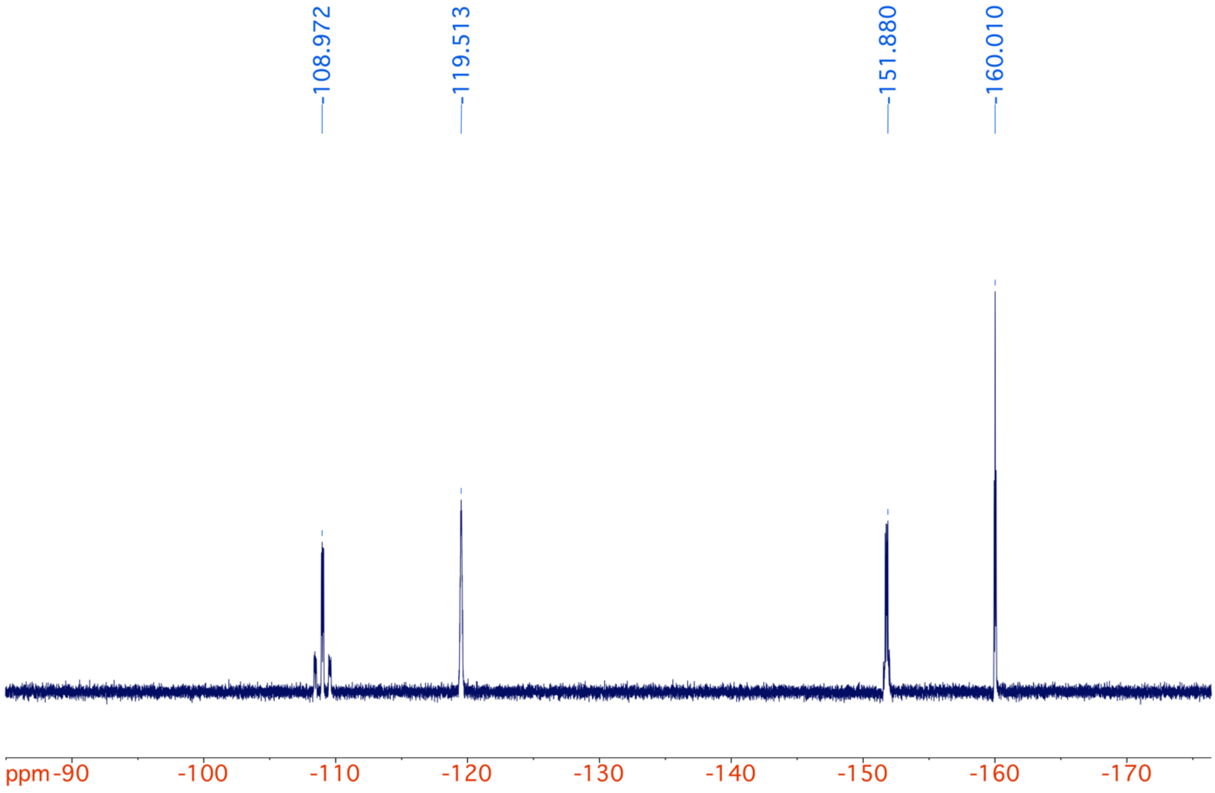

Figure S33. ^{19}F NMR spectrum in CD_2Cl_2 of *trans*-[$(Me_3P)_2Pt(\mu$-2-$C_6F_4PPh_2)_2AgCl$] (*trans*-**5PtAg**).

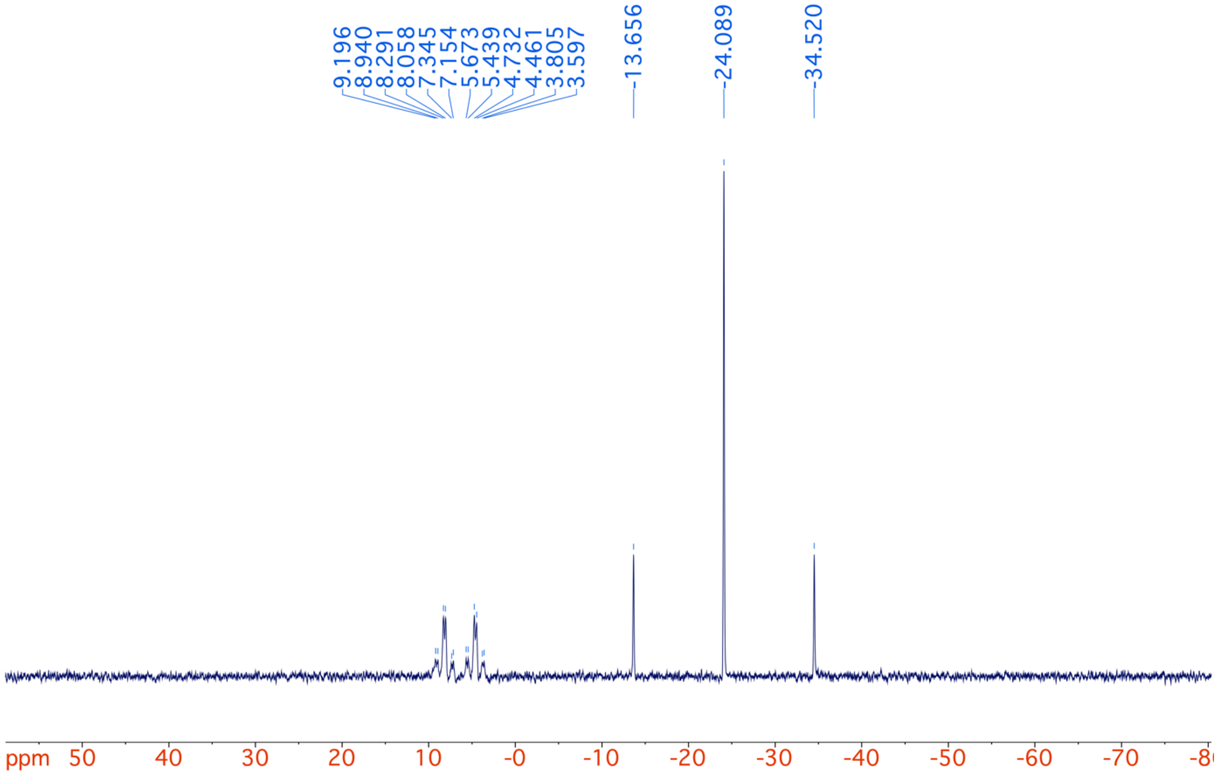

Figure S34. ^{31}P NMR spectrum in CD_2Cl_2 of *trans*-[$(Me_3P)_2Pt(\mu$-2-$C_6F_4PPh_2)_2AgCl$] (*trans*-**5PtAg**).

***trans*-[(Me$_3$P)$_2$Pt(μ-2-C$_6$F$_4$PPh$_2$)$_2$AuCl] (*trans*-5PtAu).** To a stirred solution of *trans*-[(Me$_3$P)$_2$Pt(μ-2-C$_6$F$_4$PPh$_2$)$_2$] (85 mg, 84 μmol) in dichloromethane (15 mL) cooled in a NaCl-ice bath was added solid [AuCl(tht)] (27 mg, 84 μmol). The mixture was stirred for 5 min, *n*-hexane (10 mL) was added and the volume of the pale yellow solution was reduced. The white/greenish solid was filtered off, washed with *n*-hexane and dried at 40 °C *in vacuo.* Yield: 50 mg (40 μmol, 48%).

^{1}H NMR (CD$_2$Cl$_2$): 1.06 (t, $^2J_{P,H}$ = 3.74 Hz, $^3J_{Pt,H}$ = 30.23 Hz, 18H, PMe$_3$), 7.42-7.68 (br. m, 20H, aryl). ^{19}F NMR (CD$_2$Cl$_2$): -106.6 (br. m, 2F), -117.9 (m, 2F), -148.3 (m, 2F), -158.7 (m, 2F). ^{31}P NMR (CD$_2$Cl$_2$): 35.2 (m, $^3J_{Pt,P}$ = 215 Hz, 2P, P-Au), -26.0 (m, $^1J_{Pt,P}$ = 2555 Hz, 2P, P-Pt). Anal. Calcd. for C$_{42}$H$_{38}$AuClF$_8$P$_4$Pt (MW: 1246.1): C 40.48, H 3.07, Cl 2.84, F 12.20; found: C 40.48, H 3.04, Cl 3.12, F 12.17. ESI-MS (m/z): 1210.11 [M-Cl]$^+$

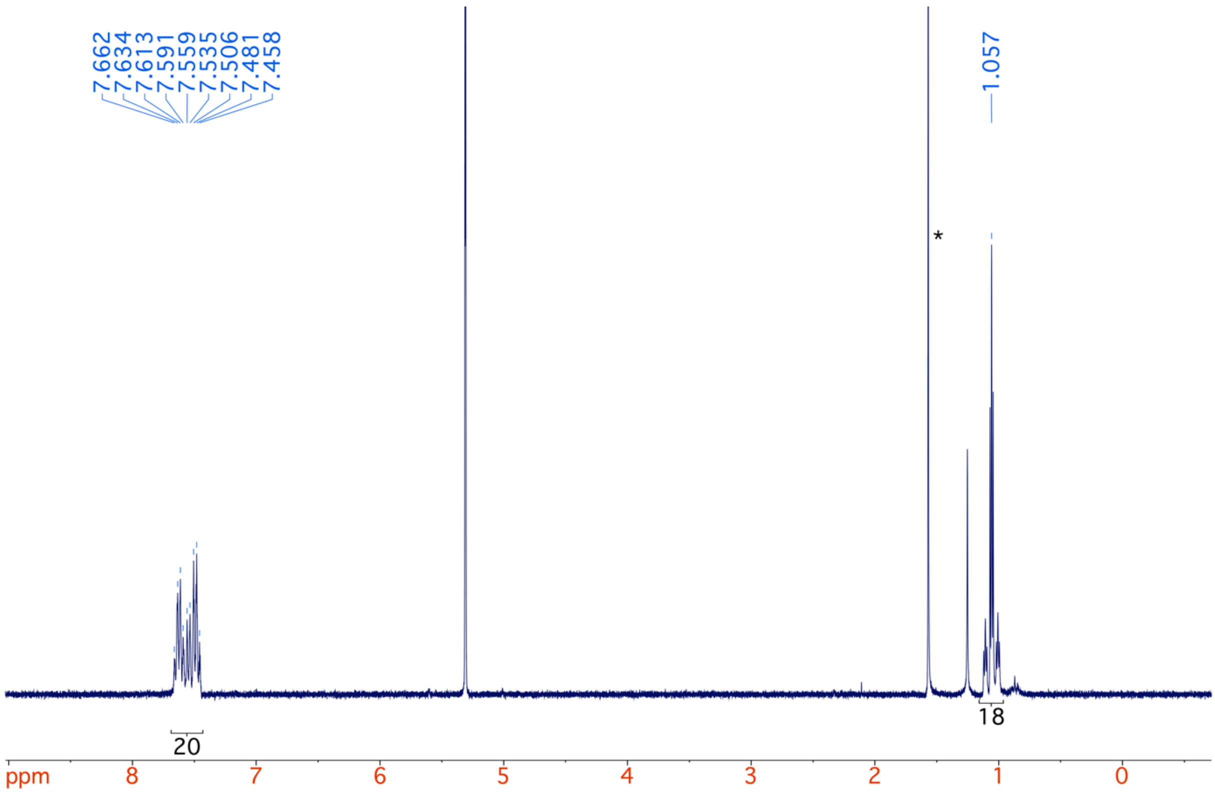

Figure S35. ^{1}H NMR spectrum in CD$_2$Cl$_2$ of *trans*-[(Me$_3$P)$_2$Pt(μ-2-C$_6$F$_4$PPh$_2$)$_2$AuCl] (*trans*-**5PtAu**). ^{1}H NMR signal of H$_2$O is marked with asterisk.

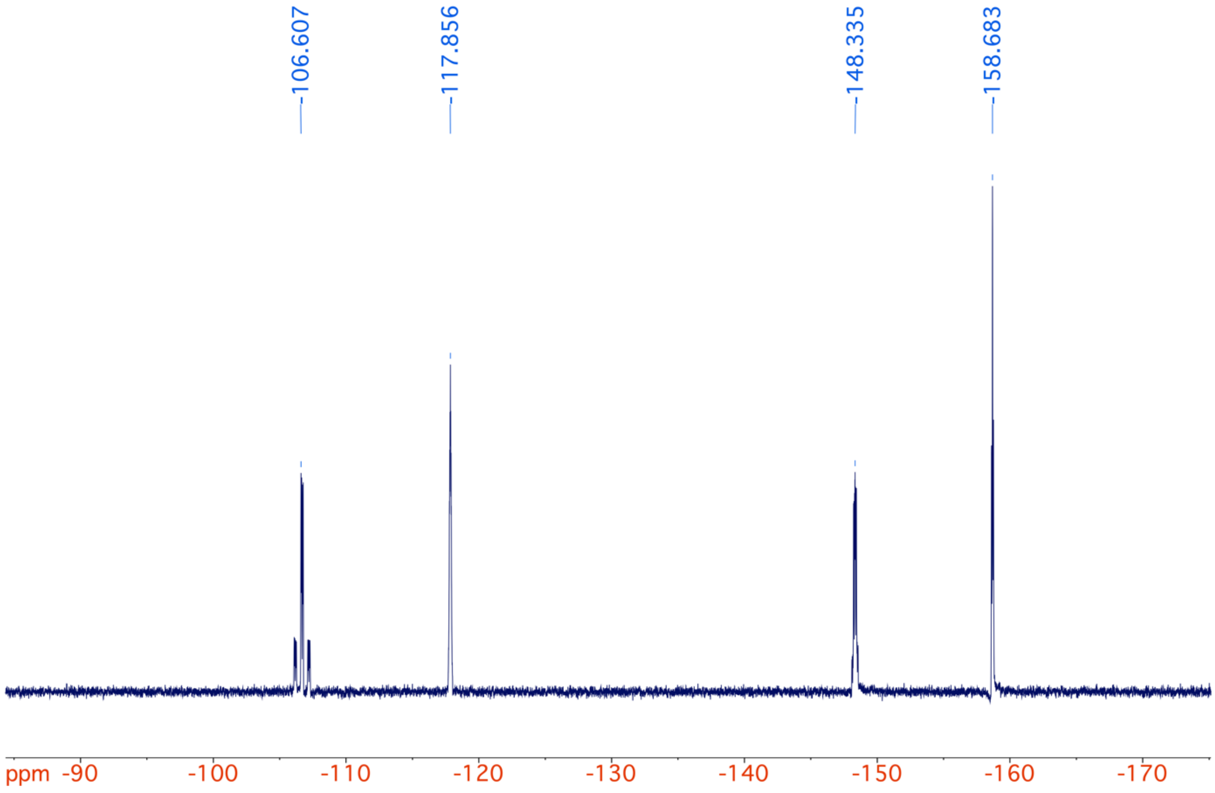

Figure S36. ^{19}F NMR spectrum in CD_2Cl_2 of *trans*-[$(Me_3P)_2Pt(\mu$-2-$C_6F_4PPh_2)_2AuCl$] (*trans*-**5PtAu**).

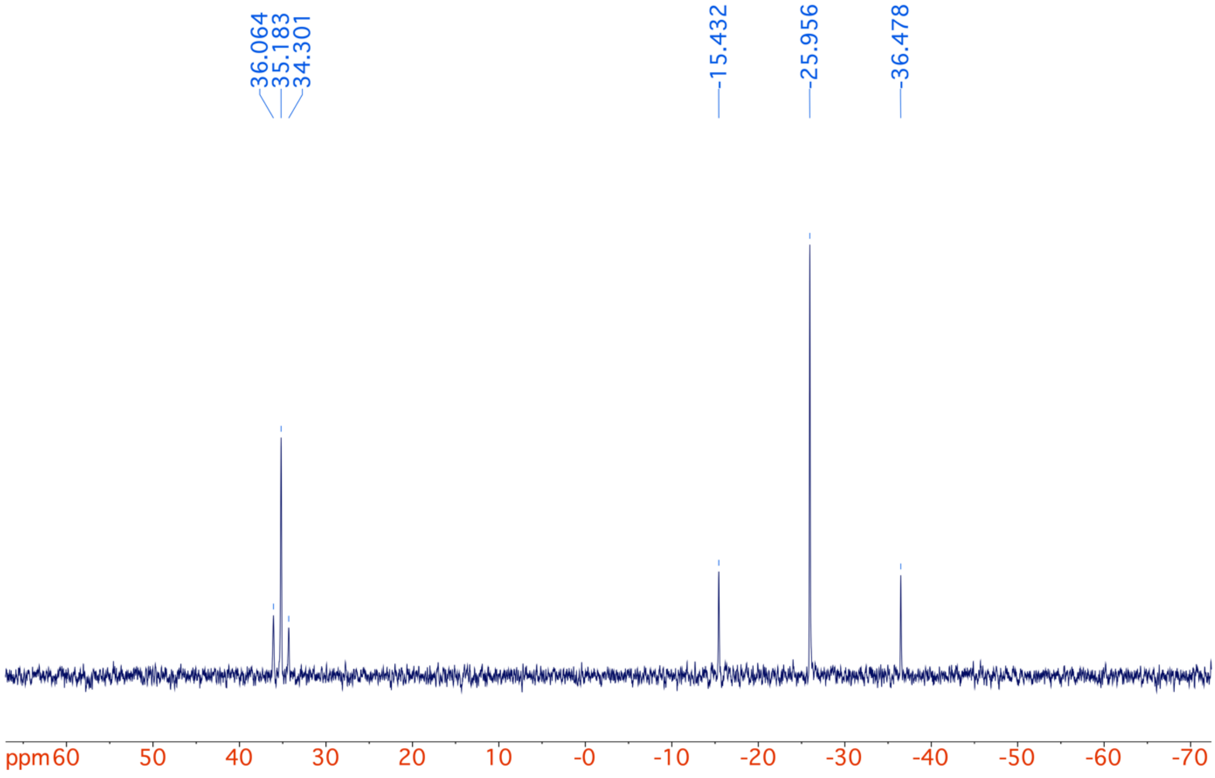

Figure S37. ^{31}P NMR spectrum in CD_2Cl_2 of *trans*-[$(Me_3P)_2Pt(\mu$-2-$C_6F_4PPh_2)_2AuCl$] (*trans*-**5PtAu**).

***cis*-[(Me$_3$P)$_2$Pt(μ-2-C$_6$F$_4$PPh$_2$)$_2$CuCl] (*cis*-5PtCu).** [(Me$_3$P)Pt(κ^2-2-C$_6$F$_4$PPh$_2$)(κC-2-C$_6$F$_4$PPh$_2$)] (100 mg, 107 µmol) and CuCl (13 mg, 131 µmol) were suspended in dichloromethane (15 mL). After stirring the colourless suspension at ambient temperature for 5 h, the mixture was filtered thru Celite. To the filtrate 1 M PMe$_3$-toluene-solution (170 µL) was added dropwise. After stirring the mixture for additional 10 min, methanol (10 mL) was added to the filtrate and the volume of the solution was reduced. The white solid was filtered off, washed with methanol and dried at 40 °C *in vacuo*. Yield: 47 mg (42 µmol, 39%).

^{1}H NMR: 1.17 (d, $^2J_{P,H}$ = 9.19 Hz, satellite: $^3J_{Pt,H}$ = 23.37 Hz, 18H, PMe$_3$), 6.62 (br. m, 4H, aryl), 6.87 (m, 4H, aryl), 7.06 (m, 2H, aryl), 7.43 (m, 6H, aryl), 8.04 (br. m, 4H, aryl). ^{19}F NMR: -109.0 (br. m, 2F), -117.4 (m, 2F), -151.2 (m, 2F), -159.9 (m, 2F). ^{31}P NMR: 1.7 (m, 2P, PPh$_2$), -30.6 (s, $^1J_{Pt,P}$ = 2130 Hz, 1P, PMe$_3$). Anal. Calcd. for $C_{42}H_{38}CuClF_8P_4Pt$ (MW: 1112.7): C 45.34, H 3.44, Cl 3.19, F 13.66; found: C 45.34, H 3.29, Cl 3.41, F 13.38. ESI-MS (m/z): 1077.08 [M-Cl+H]$^+$

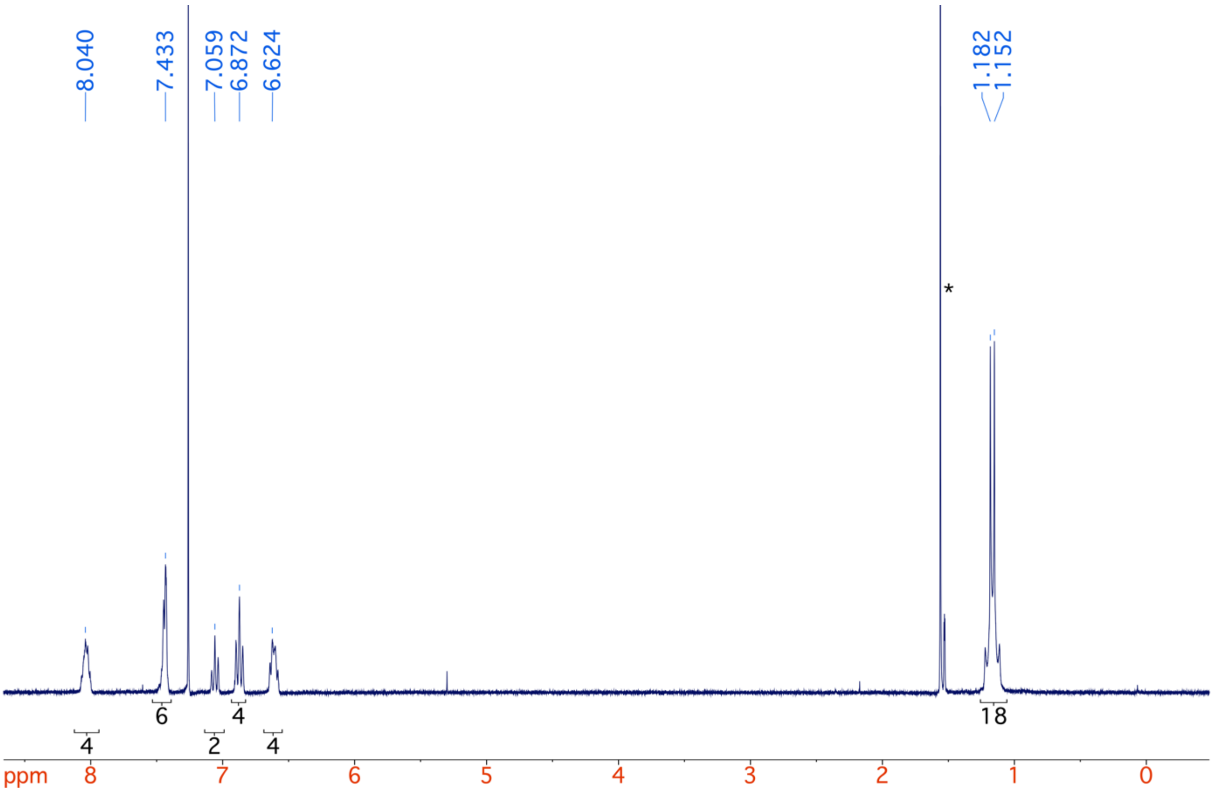

Figure S38. ^{1}H NMR spectrum in CDCl$_3$ of *cis*-[(Me$_3$P)$_2$Pt(*µ*-2-C$_6$F$_4$PPh$_2$)$_2$CuCl] (*cis*-**5PtCu**). ^{1}H NMR signal of H$_2$O is marked with asterisk.

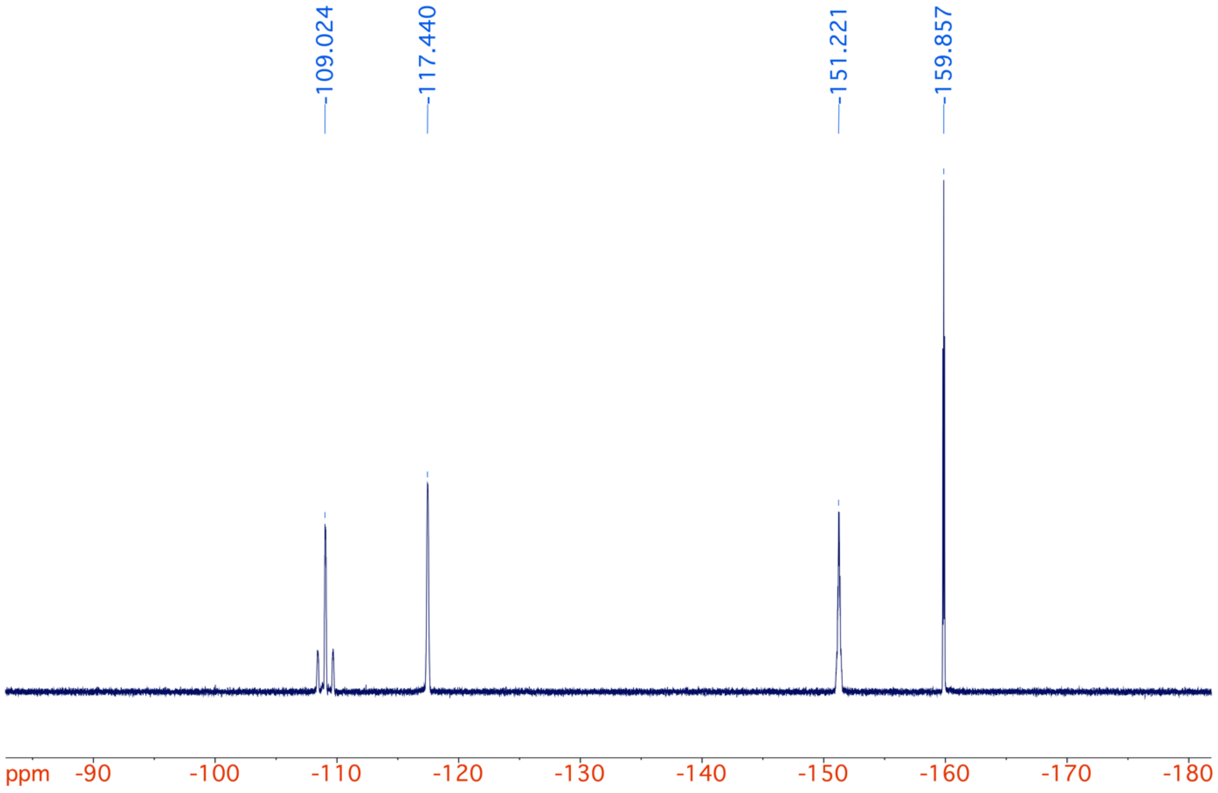

Figure S39. ^{19}F NMR spectrum in $CDCl_3$ of *cis*-[$(Me_3P)_2Pt(\mu\text{-}2\text{-}C_6F_4PPh_2)_2CuCl$] (*cis*-**5PtCu**).

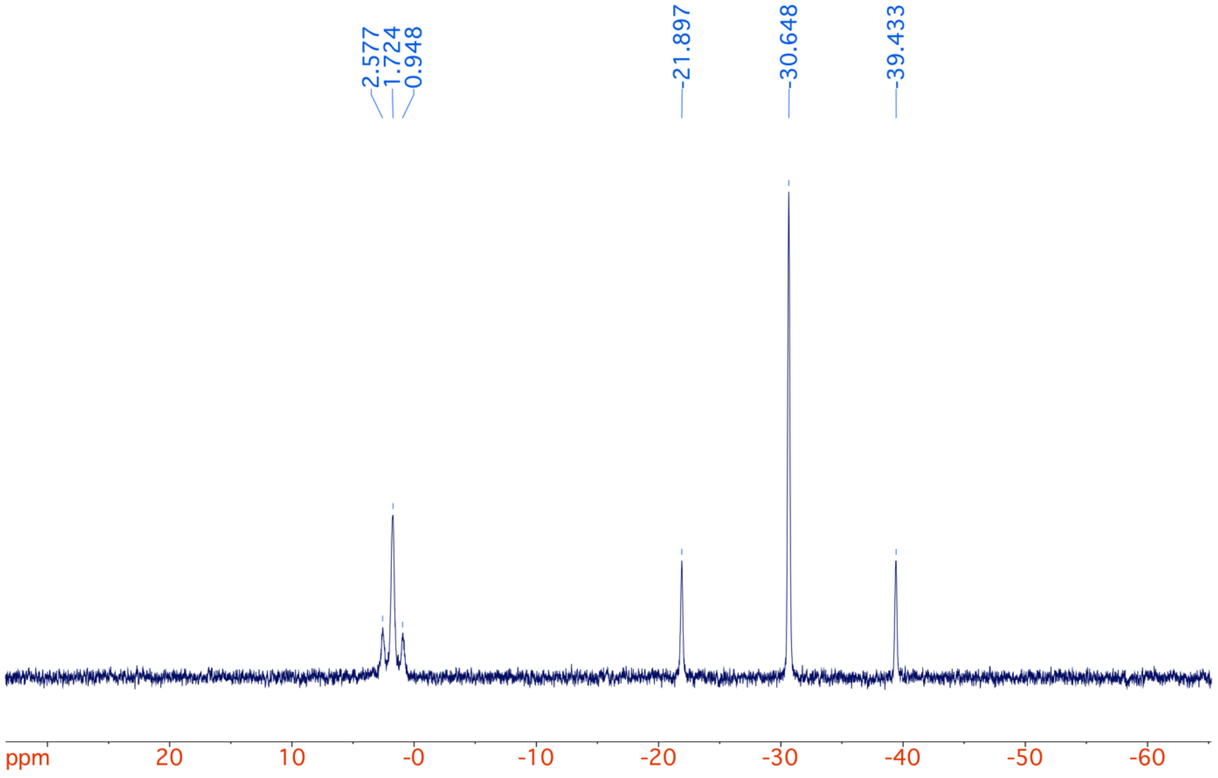

Figure S40. ^{31}P NMR spectrum in $CDCl_3$ of *cis*-[$(Me_3P)_2Pt(\mu\text{-}2\text{-}C_6F_4PPh_2)_2CuCl$] (*cis*-**5PtCu**).

***cis*-[(Me$_3$P)Pt(κ^2-2-C$_6$F$_4$PPh$_2$)(μ-2-C$_6$F$_4$PPh$_2$)CuCl] (*cis*-6PtCu).** [(Me$_3$P)Pt(κ^2-2-C$_6$F$_4$PPh$_2$)(κC-2-C$_6$F$_4$PPh$_2$)] (100 mg, 107 μmol) and CuCl (13 mg, 131 μmol) were suspended in dichloromethane (15 mL). After stirring the colourless suspension at ambient temperature for 5 h, the mixture was filtered thru Celite, and methanol (10mL) was added to the filtrate. The volume of the solution was reduced. The white solid was filtered off, washed with methanol and dried at 40 °C *in vacuo*. Yield: 68 mg (66 μmol, 62%).

^{1}H NMR: 1.58 (d, $^2J_{P,H}$ = 9.89 Hz, satellite: $^3J_{Pt,H}$ = 26.89 Hz, 9H, PMe$_3$), 7.26-7.73 (br. m, 18H, aryl), 7.82-7.94 (br. m, 2H, aryl). ^{19}F NMR: -113.2 (br. m, 1F), -123.6 (m, 1F), -126.0 (br. m, 1F), -135.2 (m, 1F), -149.1 (m, 1F), -151.5 (m, 1F), -158.0 (m, 1F), -159.3 (m, 1F). ^{31}P NMR: 16.5 (m, $^3J_{Pt,P}$ = 340 Hz, 1P, CuPPh$_2$), -29.5 (m, $^1J_{Pt,P}$ = 2180 Hz, 1P, PMe$_3$), -60.0 (m, $^1J_{Pt,P}$ = 1575 Hz, 1P, PtPPh$_2$). Anal. Calcd. for C$_{39}$H$_{29}$CuClF$_8$P$_3$Pt (MW: 1036.6): C 45.19, H 2.82, Cl 3.42, F 14.66; found: C 45.27, H 2.78, Cl 3.43, F 14.49. ESI-MS (m/z): 1001.04 [M-Cl+H]$^+$

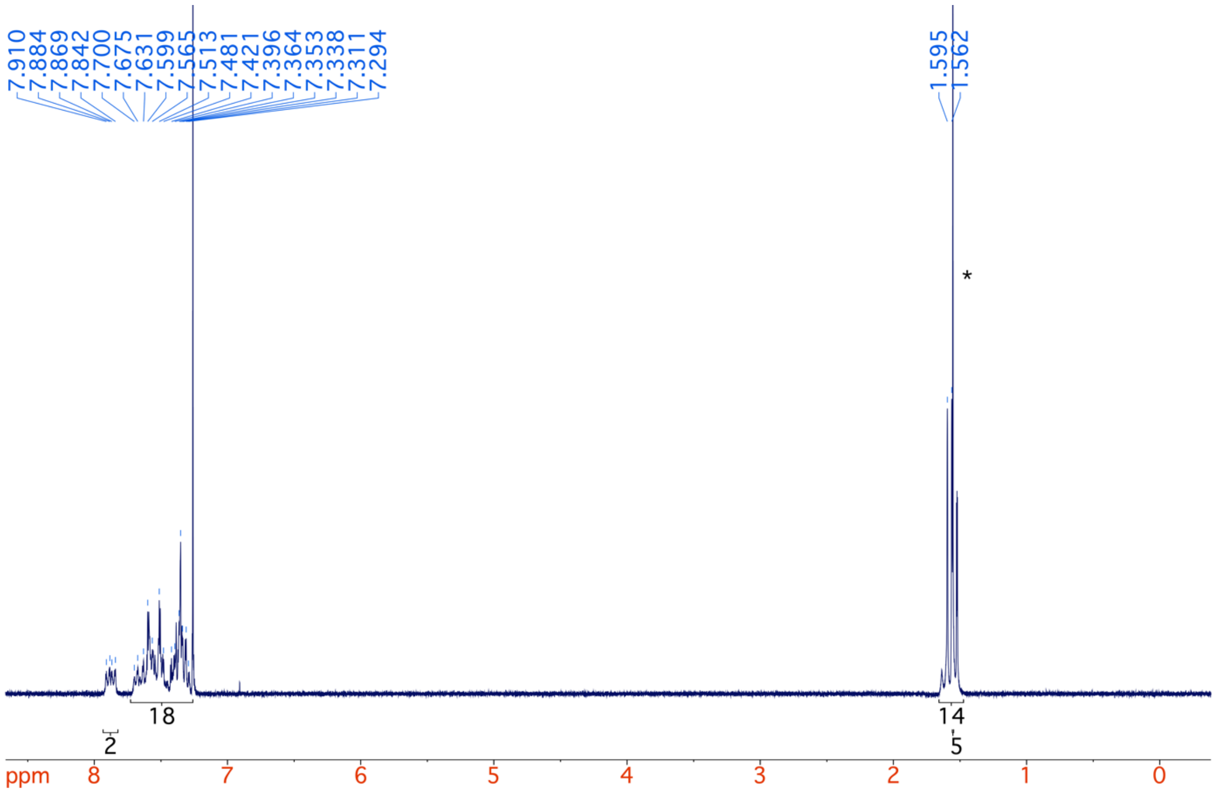

Figure S41. ^{1}H NMR spectrum in CDCl$_3$ of *cis*-[(Me$_3$P)Pt(κ^2-2-C$_6$F$_4$PPh$_2$)(μ-2-C$_6$F$_4$PPh$_2$) CuCl] (*cis*-**6PtCu**). ^{1}H NMR signal of H$_2$O is marked with asterisk.

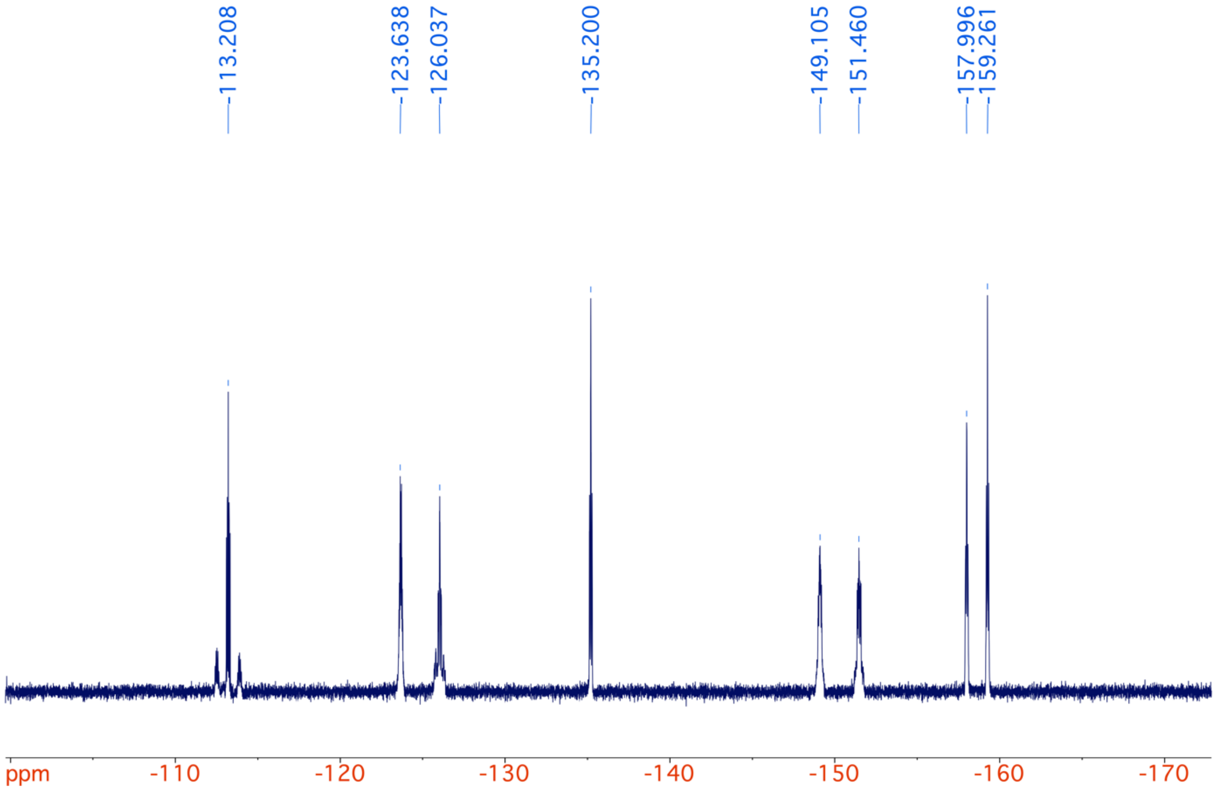

Figure S42. ^{19}F NMR spectrum in $CDCl_3$ of *cis*-[$(Me_3P)Pt(\kappa^2\text{-}2\text{-}C_6F_4PPh_2)(\mu\text{-}2\text{-}C_6F_4PPh_2)$ CuCl] (*cis*-**6PtCu**).

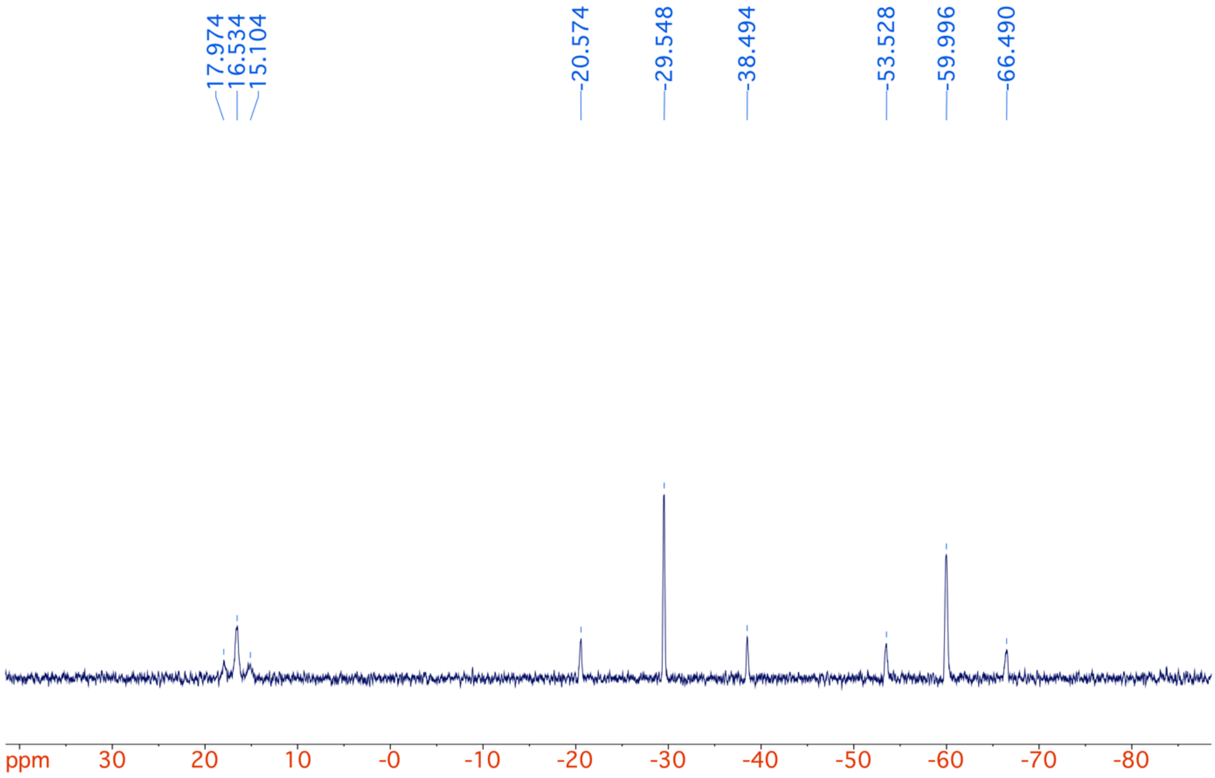

Figure S43. ^{31}P NMR spectrum in $CDCl_3$ of *cis*-[$(Me_3P)Pt(\kappa^2\text{-}2\text{-}C_6F_4PPh_2)(\mu\text{-}2\text{-}C_6F_4PPh_2)$ CuCl] (*cis*-**6PtCu**).

***cis*-[(Me_3P)Pt(κ^2-2-$C_6F_4PPh_2$)(μ-2-$C_6F_4PPh_2$)AgCl]$_2$ (*cis*-6PtAg-dimer).** [(Me_3P)Pt(κ^2-2-$C_6F_4PPh_2$)(κC-2-$C_6F_4PPh_2$)] (100 mg, 107 µmol) and AgCl (18 mg, 126 µmol) were suspended in dichloromethane (15 mL). After stirring the colourless suspension at ambient temperature in the dark overnight, the mixture was filtered thru Celite, and methanol (10 mL) was added to the filtrate. The volume of the solution was reduced. The white solid was filtered off, washed with methanol and dried at 40 °C *in vacuo*. Yield: 63 mg (29 µmol, 54%).

^{1}H NMR: 1.54 (d, $^2J_{P,H}$ = 9.24 Hz, satellite: $^3J_{Pt,H}$ = 25.57 Hz, 18H, PMe_3), 7.27-7.57 (br. m, 26H, aryl), 7.58-7.66 (br. m, 6H, aryl), 7.68-7.87 (br. m, 8H, aryl). ^{19}F NMR: -111.3 (br. m, 2F), -122.4 (m, 2F), -126.1 (m, 2F), -135.2 (m, 2F), -148.7 (m, 2F), -151.1 (m, 2F), -157.7 (m, 2F), -158.9 (m, 2F). ^{31}P NMR: 21.6 (ddd, $^1J_{(109)Ag,P}$ = 690 Hz, $^1J_{(107)Ag,P}$ = 600 Hz, $^3J_{F,P}$ = 17 Hz, $^3J_{Pt,P}$ = 395 Hz, 2P, $AgPPh_2$), -29.8 (m, $^1J_{Pt,P}$ = 2180 Hz, 2P, PMe_3), -60.7 (m, $^1J_{Pt,P}$ = 1575 Hz, 2P, $PtPPh_2$). Anal. Calcd. for $C_{78}H_{58}Ag_2Cl_2F_{16}P_6Pt_2$ (MW: 2161.9): C 43.33, H 2.70, Cl 3.28, F 14.06; found: C 43.64, H 2.64, Cl 2.96, F 13.86. ESI-MS (m/z): 2125.99 $[M-Cl+H]^+$

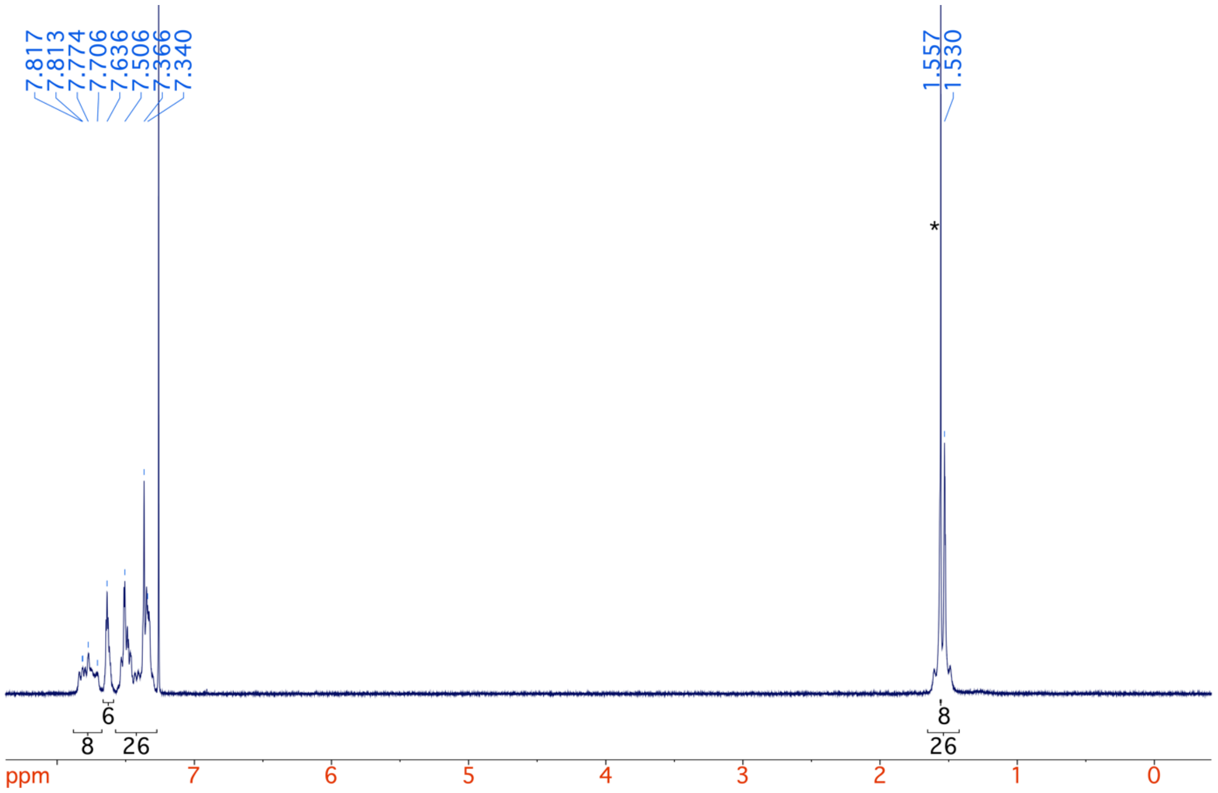

Figure S44. ^{1}H NMR spectrum in $CDCl_3$ of *cis*-[(Me_3P)Pt(κ^2-2-$C_6F_4PPh_2$)(μ-2-$C_6F_4PPh_2$) AgCl]$_2$ (*cis*-**6PtAg-dimer**). ^{1}H NMR signal of H_2O is marked with asterisk.

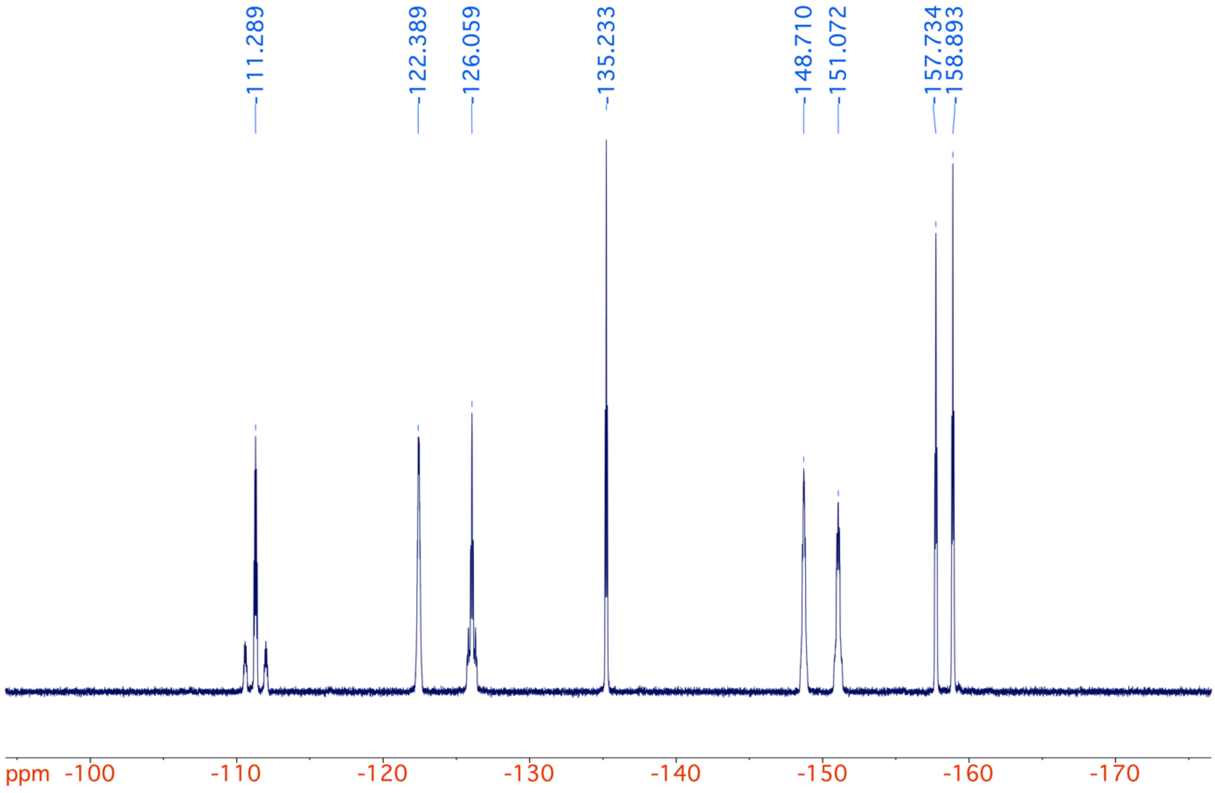

Figure S45. ^{19}F NMR spectrum in $CDCl_3$ of *cis*-[$(Me_3P)Pt(\kappa^2$-2-$C_6F_4PPh_2)(\mu$-2-$C_6F_4PPh_2)$ $AgCl]_2$ (*cis*-**6PtAg-dimer**).

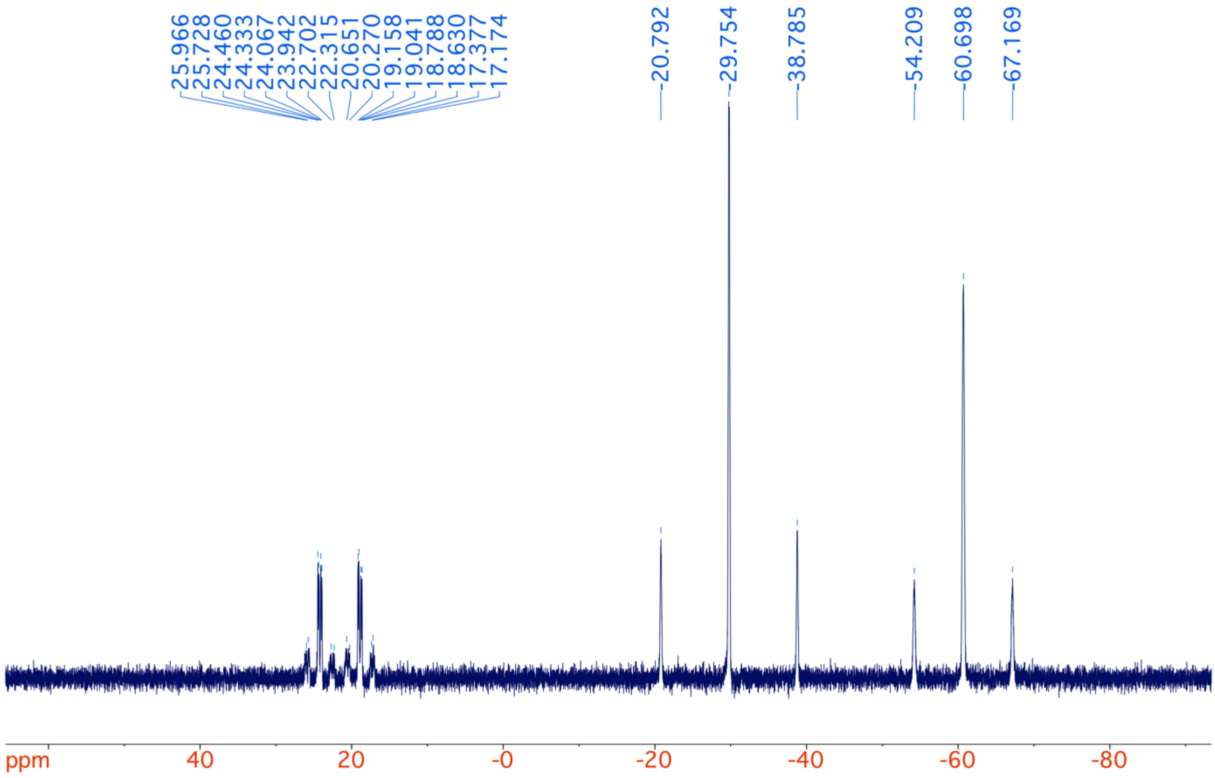

Figure S46. ^{31}P NMR spectrum in $CDCl_3$ of *cis*-[$(Me_3P)Pt(\kappa^2$-2-$C_6F_4PPh_2)(\mu$-2-$C_6F_4PPh_2)$ $AgCl]_2$ (*cis*-**6PtAg-dimer**).

***cis*-[(Me$_3$P)Pt(κ^2-2-C$_6$F$_4$PPh$_2$)(μ-2-C$_6$F$_4$PPh$_2$)AuCl] (*cis*-6PtAu).** *Cis*-[Pt(κ^2-2-C$_6$F$_4$PPh$_2$)$_2$] (100 mg, 116 μmol) and [AuCl(PMe$_3$)] (35.8 mg, 116 μmol) were dissolved in dichloromethane (10 mL). As soon as a clear solution was obtained, *n*-hexane (10 mL) was added and the volume of the solution was reduced. The white solid was filtered off, washed with *n*-hexane and air dried. Yield: 96 mg (82 μmol, 71%).

^{1}H NMR (C$_6$D$_6$): 1.36 (d, $^2J_{P,H}$ = 9.79 Hz, satellite: $^3J_{Pt,H}$ = 25.23 Hz, 9H, PMe$_3$), 6.76-6.86 (m, 2H, aryl), 6.86-7.09 (br. m, 6H, aryl), 7.20-7.44 (br. m, 6H, aryl), 7.64-7.77 (m, 2H, aryl), 7.82-7.97 (m, 4H, aryl). ^{19}F NMR (C$_6$D$_6$): -108.3 (br. m, 1F), -122.4 (m, 1F), -125.5 (br. m, 1F), -136.7 (m, 1F), -149.3 (m, 1F), -151.4 (m, 1F), -159.7 (m, 2F). ^{31}P NMR (C$_6$D$_6$): 37.6 (ddm, $^3J_{F,P}$ = 11.5 Hz, , $^4J_{F,P}$ = 11.5 Hz, $^3J_{Pt,P}$ = 300 Hz, 1P, AuPPh$_2$), -32.0 (m, $^1J_{Pt,P}$ = 2285 Hz, 1P, PMe$_3$), -62.7 (m, $^1J_{Pt,P}$ = 1700 Hz, 1P, PtPPh$_2$). Anal. Calcd. For C$_{39}$H$_{29}$AuClF$_8$P$_3$Pt (MW: 1170.1): C 40.03, H 2.50, Cl 3.03, F 12.99; found: C 40.40, H 2.89, Cl 3.22, F 12.69. ESI-MS (m/z): 1134.07 [M-Cl]$^+$

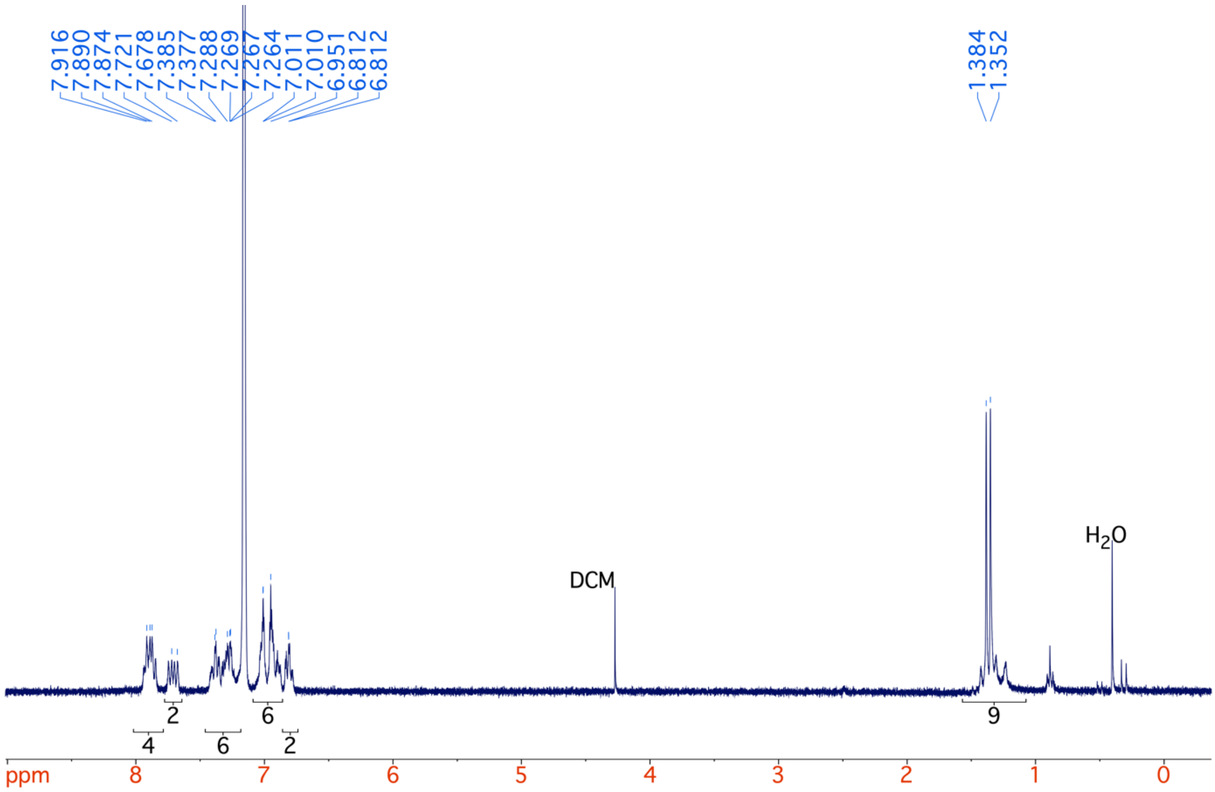

Figure S47. ^{1}H NMR spectrum in C$_6$D$_6$ of *cis*-[(Me$_3$P)Pt(κ^2-2-C$_6$F$_4$PPh$_2$)(μ-2-C$_6$F$_4$PPh$_2$) AuCl] (*cis*-**6PtAu**).

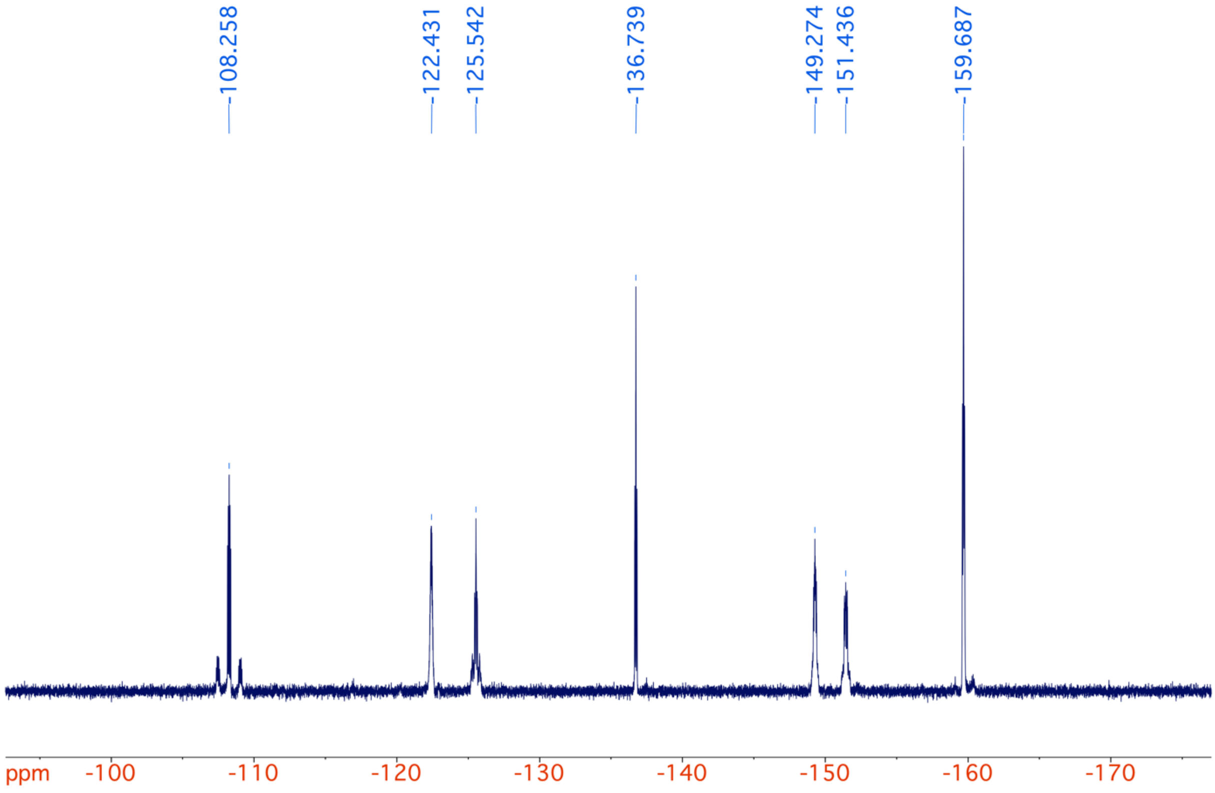

Figure S48. ^{19}F NMR spectrum in C_6D_6 of *cis*-[(Me_3P)Pt(κ^2-2-$C_6F_4PPh_2$)(μ-2-$C_6F_4PPh_2$)AuCl] (*cis*-**6PtAu**).

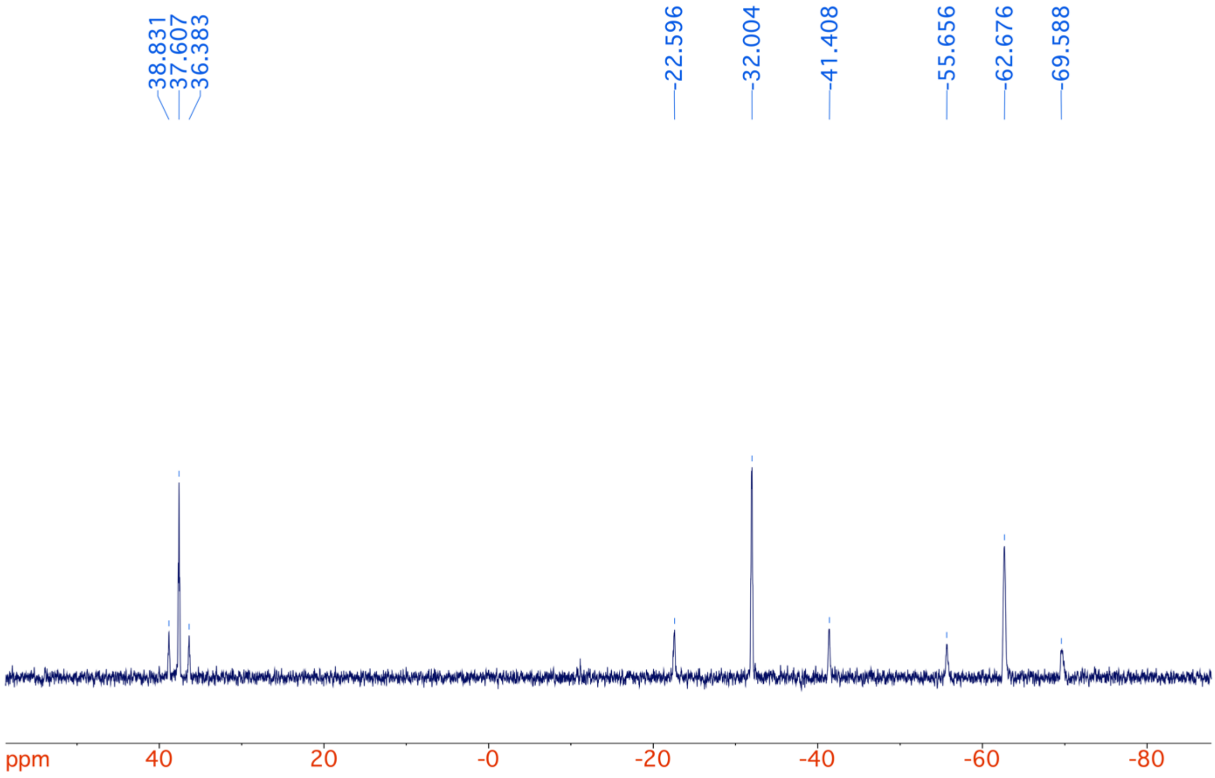

Figure S49. ^{31}P NMR spectrum in C_6D_6 of *cis*-[(Me_3P)Pt(κ^2-2-$C_6F_4PPh_2$)(μ-2-$C_6F_4PPh_2$)AuCl] (*cis*-**6PtAu**).

***Cis-trans*-[(Me$_3$P)ClNi(μ-2-C$_6$F$_4$PPh$_2$)$_2$Au] (*cis-trans*-7NiAu).** To a stirred solution of [(Me$_3$P)Ni(*μ*-2-C$_6$F$_4$PPh$_2$)$_2$] (100 mg, 125 μmol) in dichloromethane (20 mL) cooled in a dry-ice/isopropanol bath was added a solution of [AuCl(tht)] (40 mg, 125 μmol) in dichloromethane (20 mL) over a period of 1 h. After stirring the mixture for an additional 1 h at -78 °C, the solution was allowed to warm up to ambient temperature. Methanol (20 mL) was added and the volume of the orange solution was reduced. The yellow solid was filtered off and the filtrate was reduced further. The orange title compound could be isolated by filtration as second fraction. The solid was washed with methanol and dried *in vacuo*. Yield: 54 mg (52 μmol, 42%).

^{1}H NMR (CD$_2$Cl$_2$): 0.77 (dd, $^2J_{P,H}$ = 10.65 Hz, $^4J_{P,H}$ = 1.45 Hz, 9H, PMe$_3$), 6.22 (m, 2H, aryl), 6.86 (m, 2H, aryl), 7.18 (br. m, 4H, aryl), 7.31 (m, 1H, aryl), 7.43 (m, 3H, aryl), 7.52 (m, 1H, aryl), 7.62 (m, 2H, aryl), 7.67 (m, 1H, aryl), 8.07 (m, 2H, aryl), 8.30 (m, 2H, aryl). ^{19}F NMR (CD$_2$Cl$_2$): -106.7 (m, 1F), -116.4 (m, 1F), -121.0 (m, 1F), -129.9 (m, 1F), -152.9 (m, 1F), -153.0 (m, 1F), -159.5 (m, 1F), -160.1 (m, 1F). ^{31}P NMR (CD$_2$Cl$_2$): 45.3 (m, 1P, AuPPh$_2$), 21.2 (dm, $^2J_{P,P}$ = 330 Hz, 1P, NiPPh$_2$), -14.8 (d, $^2J_{P,P}$ = 330 Hz, 1P, NiPMe$_3$). Anal. Calcd. for $C_{39}H_{29}AuClF_8P_3Ni$ (MW: 1033.7): C 45.32, H 2.83; found: C 45.07, H 2.67. ESI-MS (m/z): 1037.95 [M-F+Na]$^+$

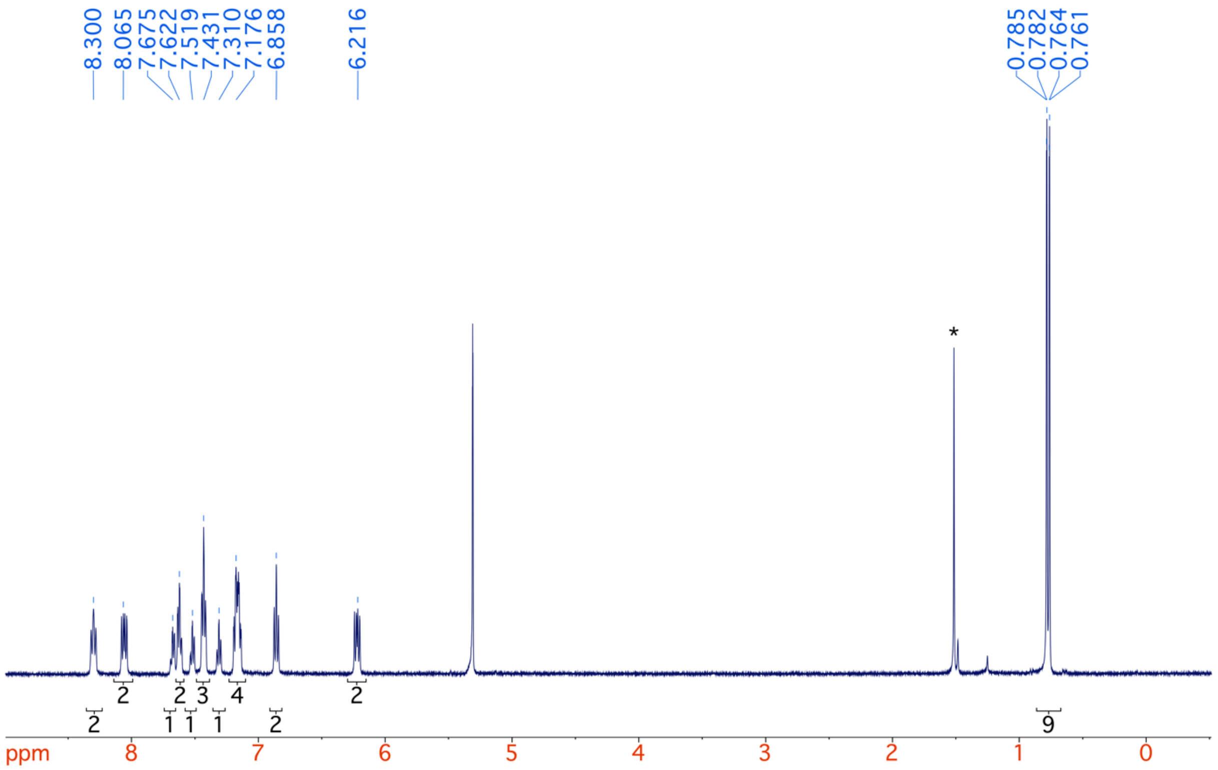

Figure S50. ^{1}H NMR spectrum in CD$_2$Cl$_2$ of *cis-trans*-[(Me$_3$P)ClNi(μ-2-C$_6$F$_4$PPh$_2$)$_2$Au] (*cis-trans*-**7NiAu**). ^{1}H NMR signal of H$_2$O is marked with asterisk.

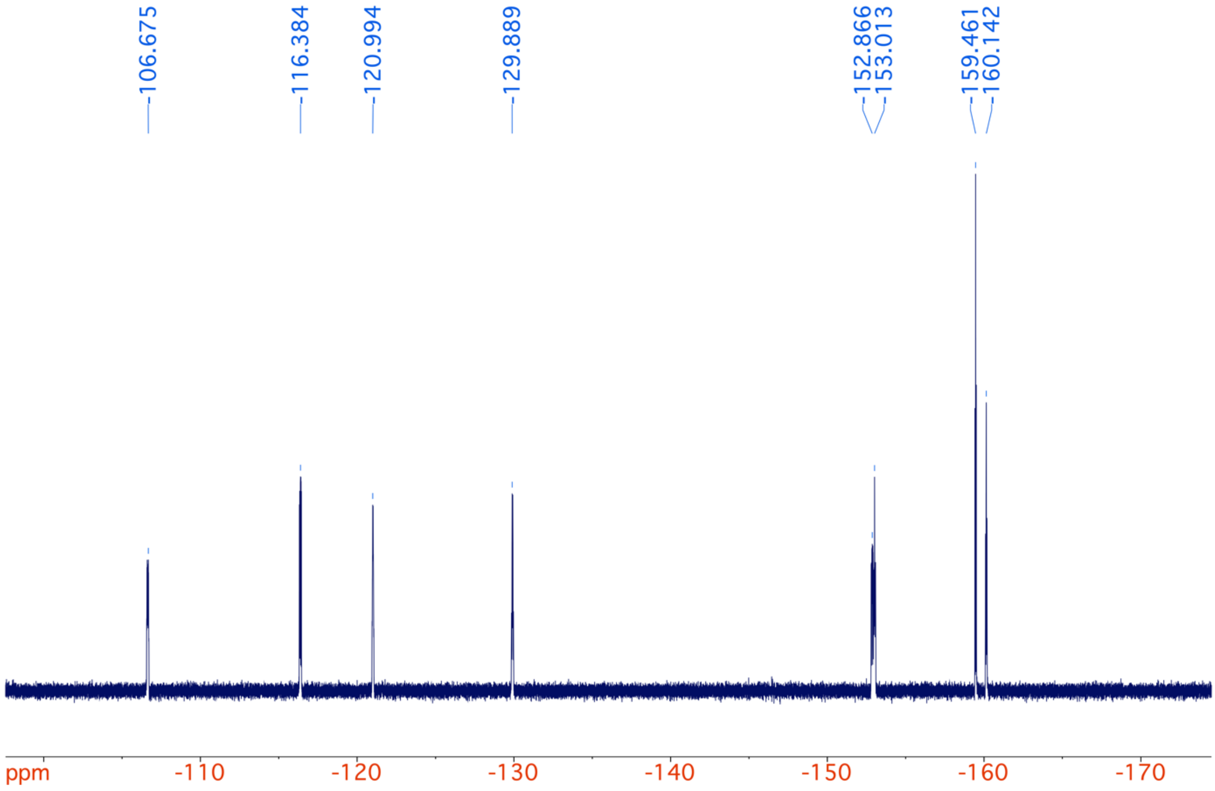

Figure S51. ^{19}F NMR spectrum in CD_2Cl_2 of *cis*-*trans*-[$(Me_3P)ClNi(\mu$-2-$C_6F_4PPh_2)_2Au$] (*cis*-*trans*-**7NiAu**).

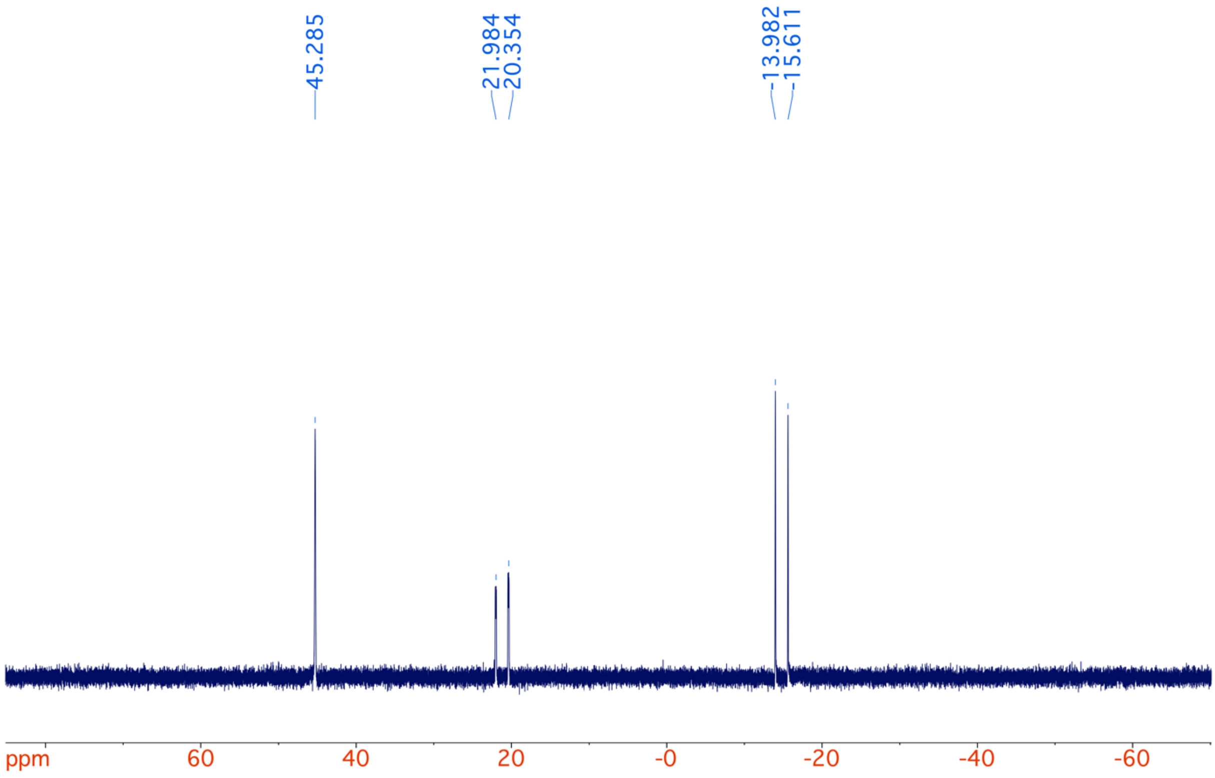

Figure S52. ^{31}P NMR spectrum in CD_2Cl_2 of *cis*-*trans*-[$(Me_3P)ClNi(\mu$-2-$C_6F_4PPh_2)_2Au$] (*cis*-*trans*-**7NiAu**).

***trans*-[(Me$_3$P)ClPt(μ-2-C$_6$F$_4$PPh$_2$)$_2$Au] (*trans*-7PtAu).** To a stirred solution of [(Me$_3$P)Pt(κ^2-2-C$_6$F$_4$PPh$_2$)(κC-2-C$_6$F$_4$PPh$_2$)] (100 mg, 107 μmol) in dichloromethane (15 mL) cooled in a NaCl-ice bath was slowly added solid [AuCl(tht)] (34 mg, 106 μmol). After stirring the mixture for 5 min, *n*-hexane (10 mL) was added and the volume of the solution was reduced. The white oily product was triturated with diethyl ether (2 mL) and the solid was filtered off, washed with ether and air dried. Yield: 53 mg (45 μmol, 42%).

^{1}H NMR (C$_6$D$_6$): 0.49 (d, $^2J_{P,H}$ = 10.85 Hz, satellite: $^3J_{Pt,H}$ = 39.25 Hz, 9H, PMe$_3$), 6.86-7.12 (br. m, 12H, aryl), 7.60-7.71 (m, 2H, aryl), 7.71-7.90 (m, 4H, aryl), 8.06 (br. m, 2H, aryl). ^{19}F NMR (C$_6$D$_6$): -105.6 (m, 1F), -112.2 (br. m, 1F), -117.0 (m, 1F), -120.2 (m, 1F), -149.2 (m, 2F), -156.7 (m, 1F), -159.1 (m, 1F). ^{31}P NMR (C$_6$D$_6$): 33.9 (m, $^3J_{Pt,P}$ = 205 Hz, 1P, AuPPh$_2$), 12.5 (m, $^1J_{Pt,P}$ = 2255 Hz, 1P, PtPPh$_2$), -35.5 (d, $^2J_{P,P}$ = 16 Hz, $^1J_{Pt,P}$ = 3655 Hz, 1P, PMe$_3$). Anal. Calcd. for C$_{39}$H$_{29}$AuClF$_8$P$_3$Pt (MW: 1170.1): C 40.03, H 2.50, Cl 3.03, F 12.99; found: C 40.05, H 2.40, Cl 3.15, F 12.65. ESI-MS (m/z): 1134.07 [M-Cl]$^+$

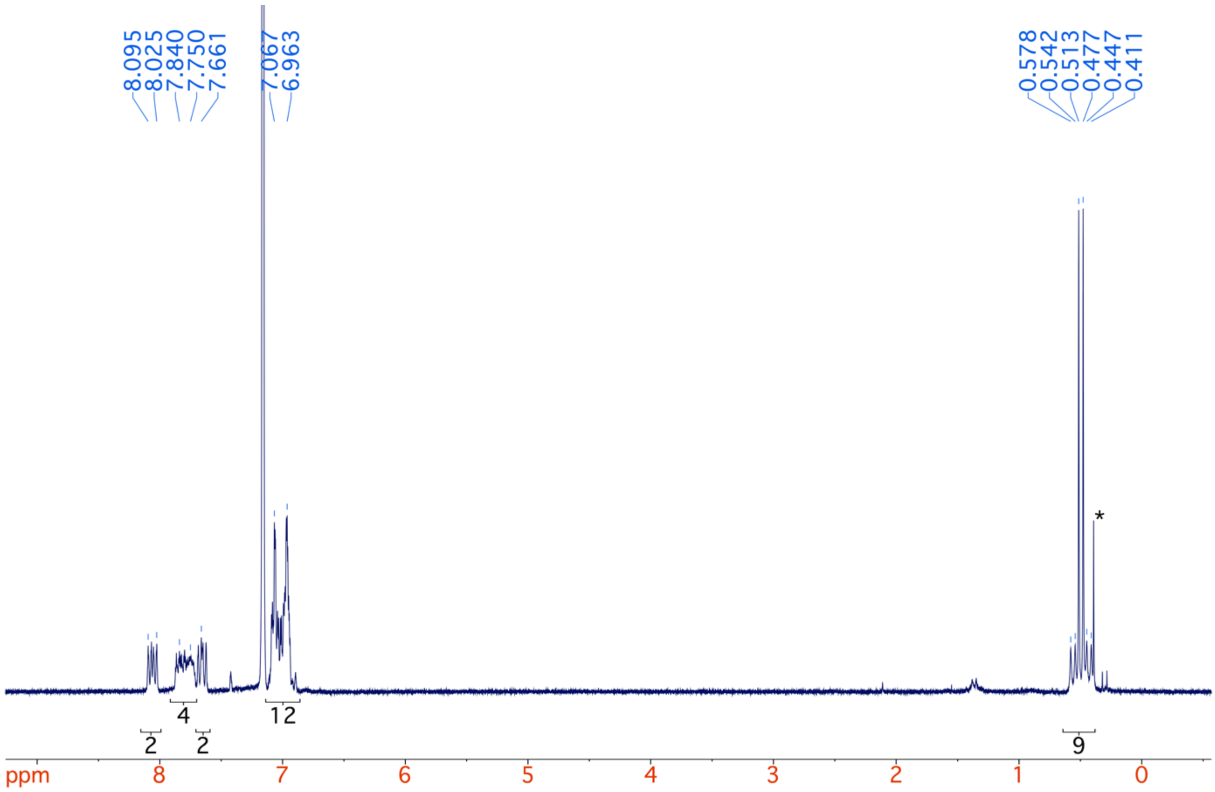

Figure S53. ^{1}H NMR spectrum in C$_6$D$_6$ of *trans*-[(Me$_3$P)ClPt(μ-2-C$_6$F$_4$PPh$_2$)$_2$Au] (*trans*-**7PtAu**). ^{1}H NMR signal of H$_2$O is marked with asterisk.

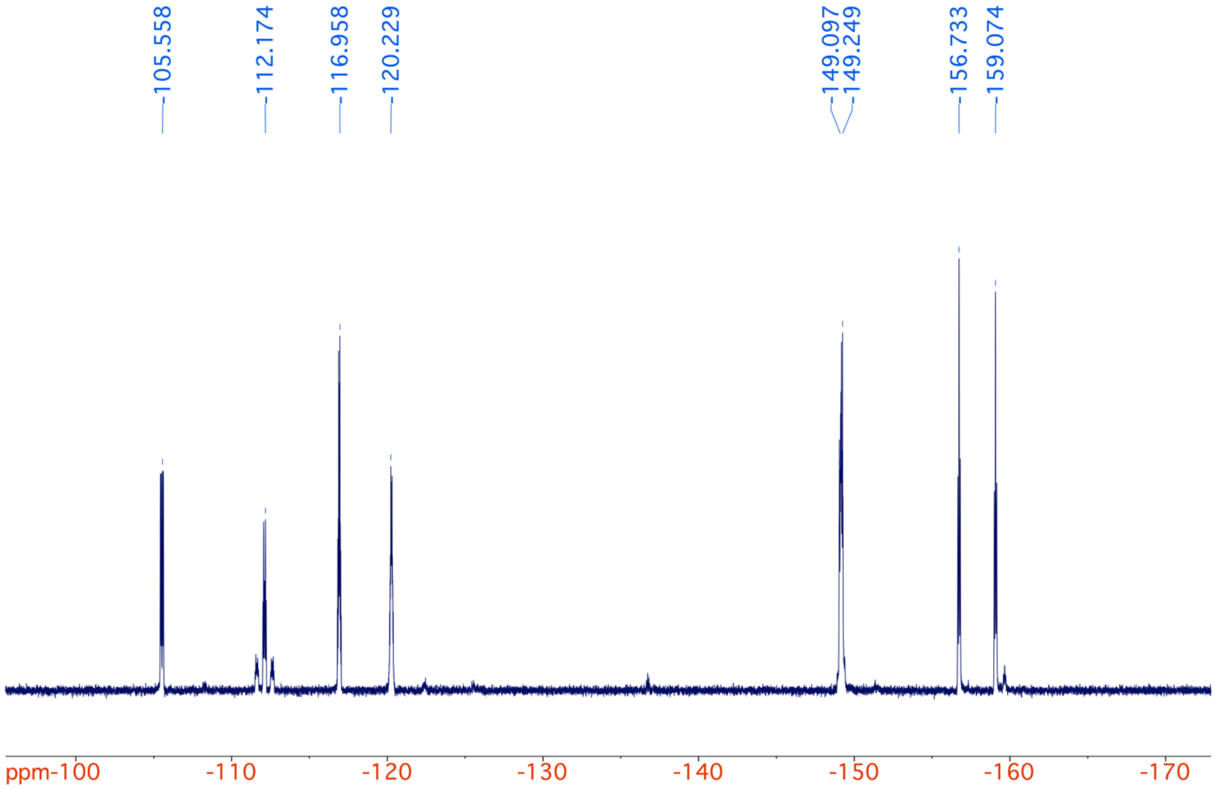

Figure S54. ^{19}F NMR spectrum in C_6D_6 of *trans*-[(Me$_3$P)ClPt(μ-2-$C_6F_4PPh_2$)$_2$Au] (*trans*-**7PtAu**).

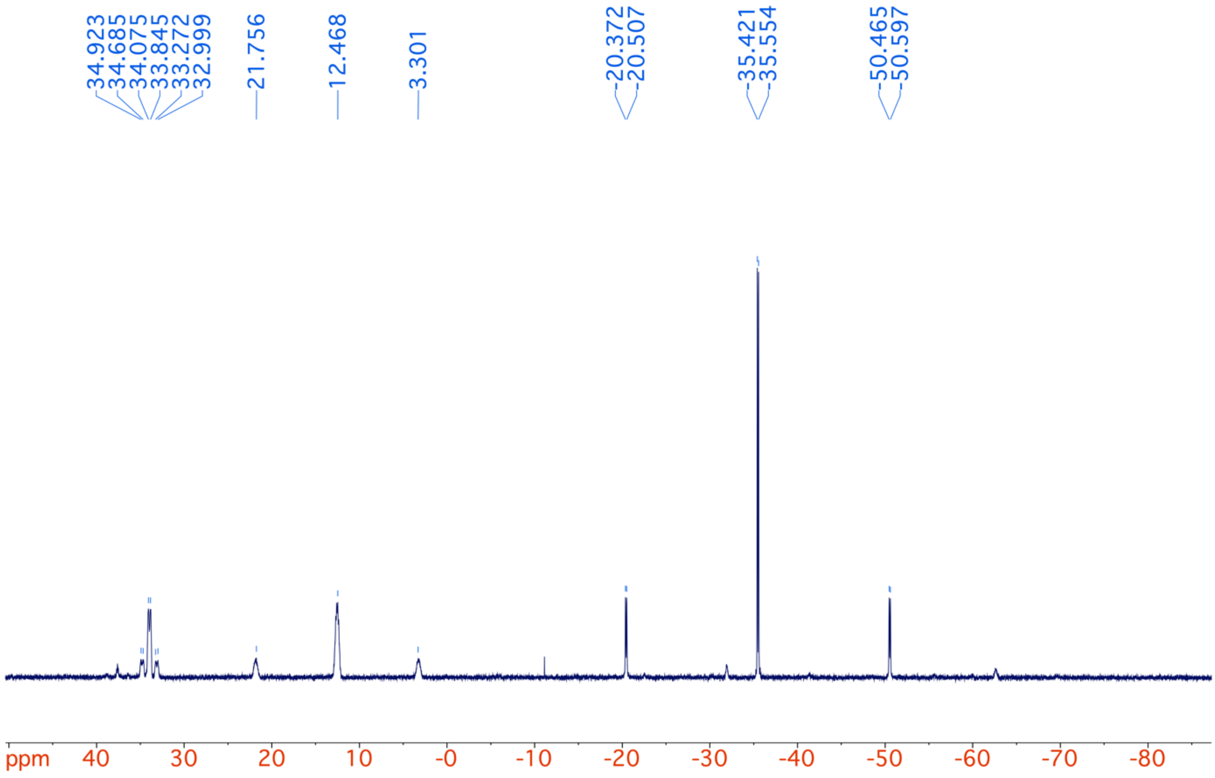

Figure S55. ^{31}P NMR spectrum in C_6D_6 of *trans*-[(Me$_3$P)ClPt(μ-2-$C_6F_4PPh_2$)$_2$Au] (*trans*-**7PtAu**).

***trans*-[(Me$_3$P)ClNi(μ-2-C$_6$F$_4$PPh$_2$)$_2$Cu] (*trans*-8NiCu).** An orange suspension of [(Me$_3$P)Ni(*μ*-2-C$_6$F$_4$PPh$_2$)$_2$] (100 mg, 125 μmol) and CuCl (30 mg, 303 μmol) in dichloromethane (10 mL) was stirred at ambient temperature for 30 min. The yellow suspension was stored at ambient temperature for 30 min to sediment excess material of CuCl. The clear solution was decanted off and to the clear yellow solution was added *n*-hexane (10 mL). The volume of the solution was reduced and the yellow solid was filtered off, washed with *n*-hexane and dried *in vacuo.* Yield: 88 mg (98 μmol, 78%).

^{1}H NMR (CD$_2$Cl$_2$): 0.44 (d, $^2J_{P,H}$ = 10.60 Hz, 9H, PMe$_3$), 7.26-7.37 (br. m, 6H, aryl), 7.42 (m, 4H, aryl), 7.50 (m, 4H, aryl), 7.56 (m, 2H, aryl), 7.84 (m, 4H, aryl). ^{19}F NMR (CD$_2$Cl$_2$): -116.4 (m, 2F), -122.8 (m, 2F), -153.6 (m, 2F), -161.8 (m, 2F). ^{31}P NMR (CD$_2$Cl$_2$): 4.2 (br. m, 2P, CuPPh$_2$), -4.7 (m, 2P, PMe$_3$). Anal. Calcd. for C$_{39}$H$_{29}$CuClF$_8$P$_3$Ni (MW: 900.2): C 52.03, H 3.25; found: C 51.94, H 3.40. ESI-MS (m/z): 904.13 [M-F+Na]$^+$

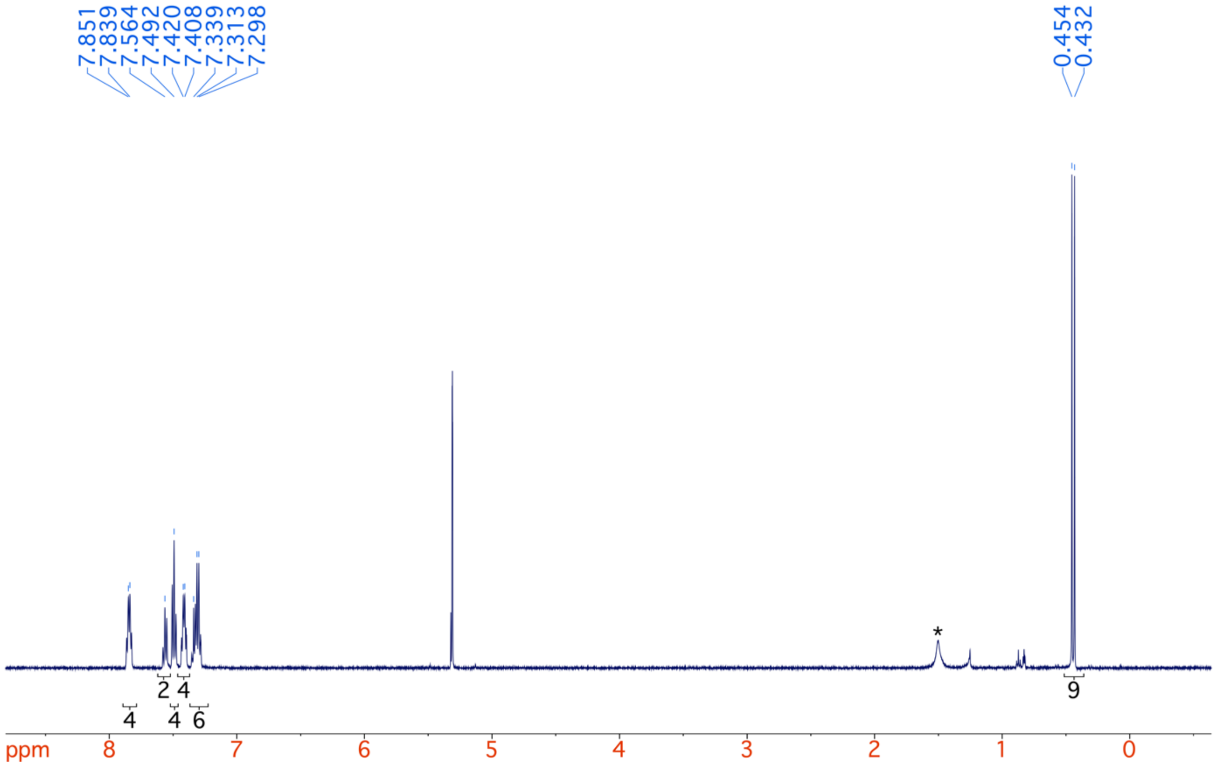

Figure S56. ^{1}H NMR spectrum in CD$_2$Cl$_2$ of *trans*-[(Me$_3$P)ClNi(μ-2-C$_6$F$_4$PPh$_2$)$_2$Cu] (*trans*-**8NiCu**). ^{1}H NMR signal of H$_2$O is marked with asterisk.

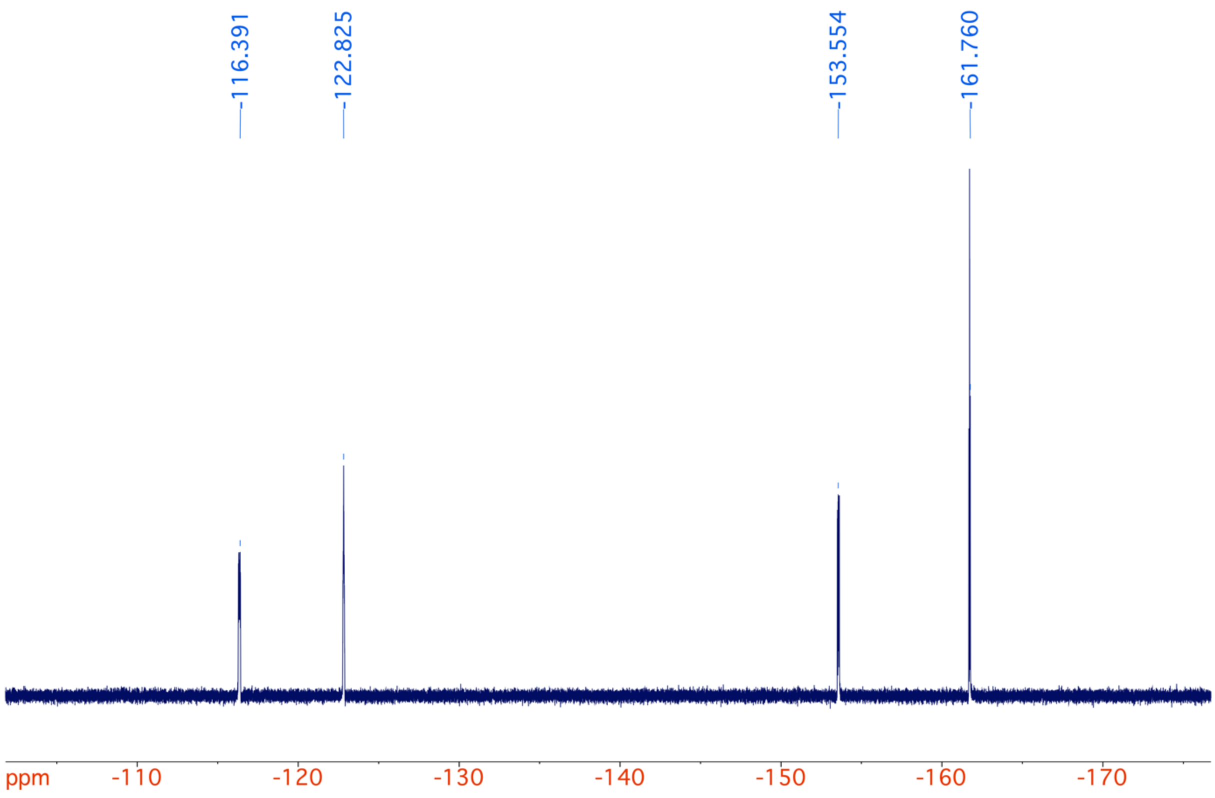

Figure S57. ^{19}F NMR spectrum in CD_2Cl_2 of *trans*-[$(Me_3P)ClNi(\mu\text{-}2\text{-}C_6F_4PPh_2)_2Cu$] (*trans*-**8NiCu**).

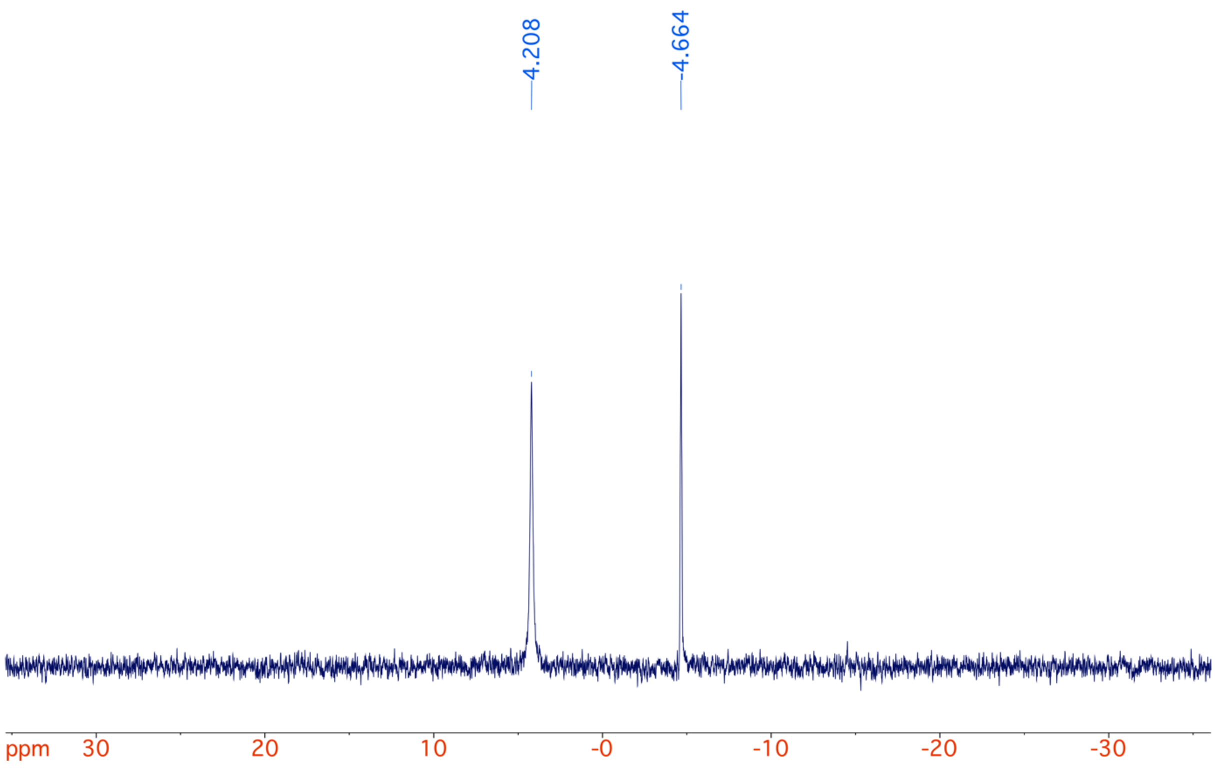

Figure S58. ^{31}P NMR spectrum in CD_2Cl_2 of *trans*-[$(Me_3P)ClNi(\mu\text{-}2\text{-}C_6F_4PPh_2)_2Cu$] (*trans*-**8NiCu**).

***trans*-[(Me$_3$P)ClNi(μ-2-C$_6$F$_4$PPh$_2$)$_2$Ag] (*trans*-8NiAg).** An orange suspension of [(Me$_3$P)Ni(*μ*-2-C$_6$F$_4$PPh$_2$)$_2$] (100 mg, 125 μmol) and AgCl (45 mg, 314 μmol) in dichloromethane (10 mL) was stirred at ambient temperature with light protection for 1 h. The yellow suspension was stored at ambient temperature for 1 h to sediment excess material of AgCl. The clear solution was decanted off and to the clear yellow solution was added *n*-hexane (10 mL). The volume of the solution was reduced and the yellow solid was filtered off, washed with *n*-hexane and dried *in vacuo*. Yield: 59 mg (62 μmol, 50%).

^{1}H NMR (CD$_2$Cl$_2$): 0.49 (d, $^2J_{P,H}$ = 10.27 Hz, 9H, PMe$_3$), 7.29-7.37 (br. m, 6H, aryl), 7.43 (m, 4H, aryl), 7.53-7.61 (br. m, 6H, aryl), 7.86 (m, 4H, aryl). ^{19}F NMR (CD$_2$Cl$_2$): -114.1 (m, 2F), -121.8 (m, 2F), -153.1 (m, 2F), -161.8 (m, 2F). ^{31}P NMR (CD$_2$Cl$_2$): 14.6 (dd, $^1J_{(109)AgP}$ = 566 Hz, $^1J_{(107)AgP}$ = 490 Hz, 2P, AgPPh$_2$), -6.1 (m, 1P, PMe$_3$). Anal. Calcd. for C$_{39}$H$_{29}$AgClF$_8$P$_3$Ni (MW: 944.6): C 49.59, H 3.09; found: C 49.28, H 3.16. ESI-MS (m/z): 948.37 [M-F+Na]$^+$

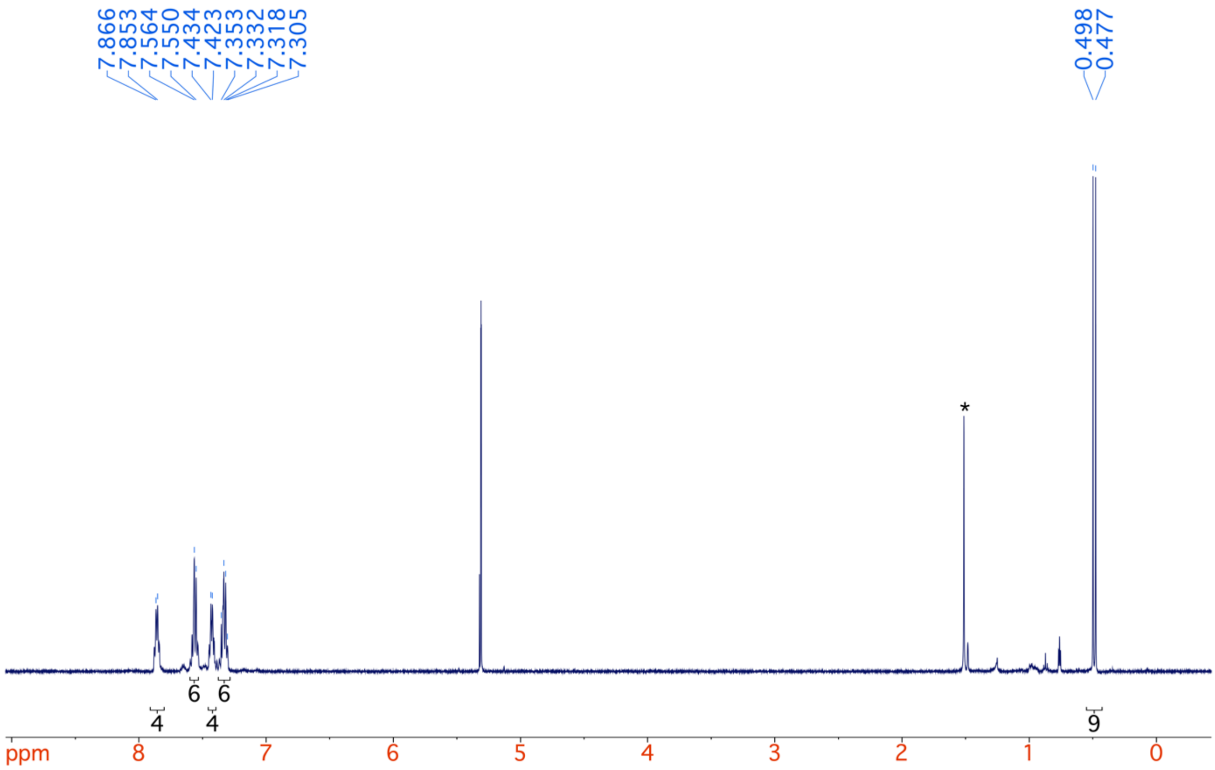

Figure S59. ^{1}H NMR spectrum in CD$_2$Cl$_2$ of *trans*-[(Me$_3$P)ClNi(μ-2-C$_6$F$_4$PPh$_2$)$_2$Ag] (*trans*-**8NiAg**). ^{1}H NMR signal of H$_2$O is marked with asterisk.

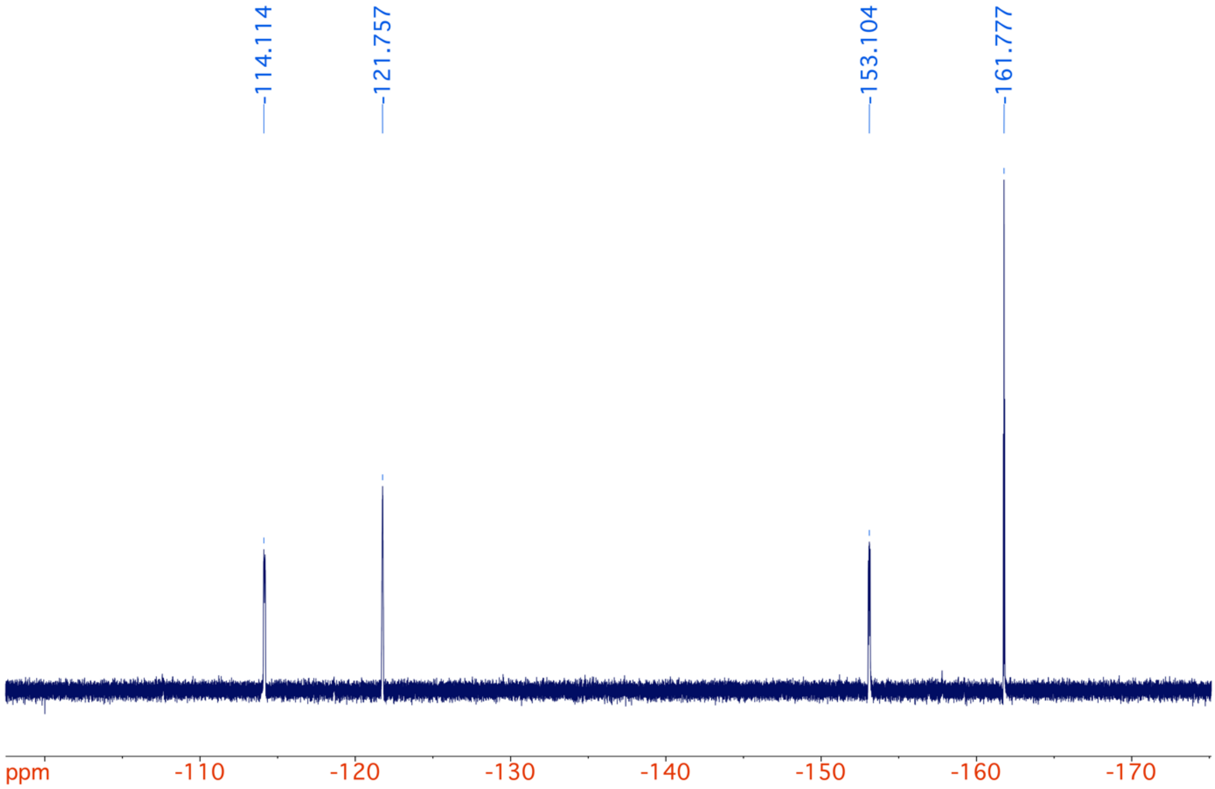

Figure S60. ^{19}F NMR spectrum in CD_2Cl_2 of *trans*-[$(Me_3P)ClNi(\mu$-2-$C_6F_4PPh_2)_2Ag$] (*trans*-**8NiAg**).

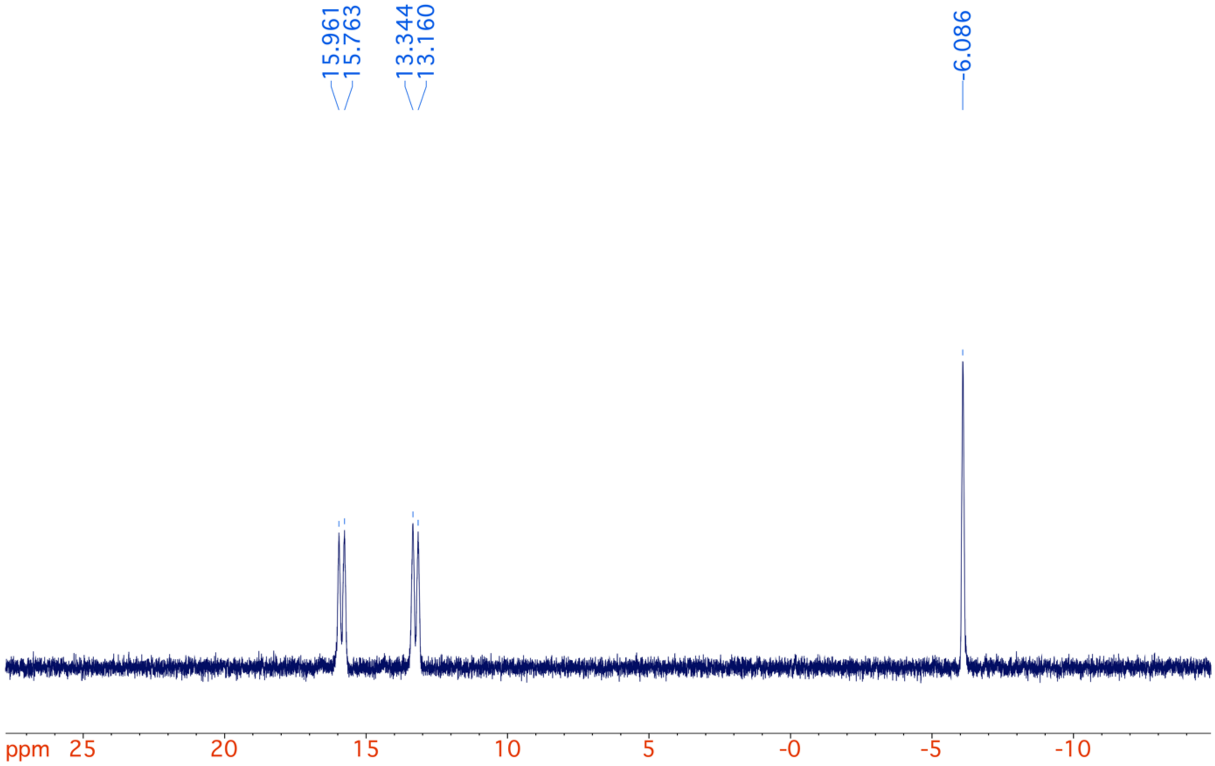

Figure S61. ^{31}P NMR spectrum in CD_2Cl_2 of *trans*-[$(Me_3P)ClNi(\mu$-2-$C_6F_4PPh_2)_2Ag$] (*trans*-**8NiAg**).

***trans*-[(Me$_3$P)ClPt(μ-2-C$_6$F$_4$PPh$_2$)$_2$Au] (*trans*-8PtAu).** *Trans*-[Pt(κ^2-2-C$_6$F$_4$PPh$_2$)$_2$] (84 mg, 97 μmol) and [AuCl(PMe$_3$)] (30 mg, 97 μmol) were dissolved in dichloromethane (30 mL) and refluxed for 4 days. To the colourless solution was filtered thru Celite and to the filtrate methanol (~10 mL) was added and the volume of the solution was reduced. The white solid was filtered off, washed with methanol and dried *in vacuo*. Yield: 98 mg (84 μmol, 87%).

^{1}H NMR: 1.02 (d, $^2J_{P,H}$ = 11.05 Hz, satellite: $^3J_{Pt,H}$ = 46.76 Hz, 9H, PMe$_3$), 7.31-7.42 (br. m, 6H, aryl), 7.47-7.61 (br. m, 6H, aryl), 7.67-7.81 (br. m, 8H, aryl). ^{19}F NMR: -110.1 (br. m, 2F), -121.3 (m, 2F), -149.4 (m, 2F), -161.2 (m, 2F). ^{31}P NMR: 33.5 (m, $^3J_{Pt,P}$ = 200 Hz, 2P, AuPPh$_2$), -29.8 (m, $^1J_{Pt,P}$ = 4080 Hz, 1P, PtPMe$_3$). Anal. Calcd. for C$_{39}$H$_{29}$AuClF$_8$P$_3$Pt (MW: 1170.1): C 40.03, H 2.50; found: C 39.90, H 2.45. ESI-MS (m/z): 1134.06 [M-Cl]$^+$

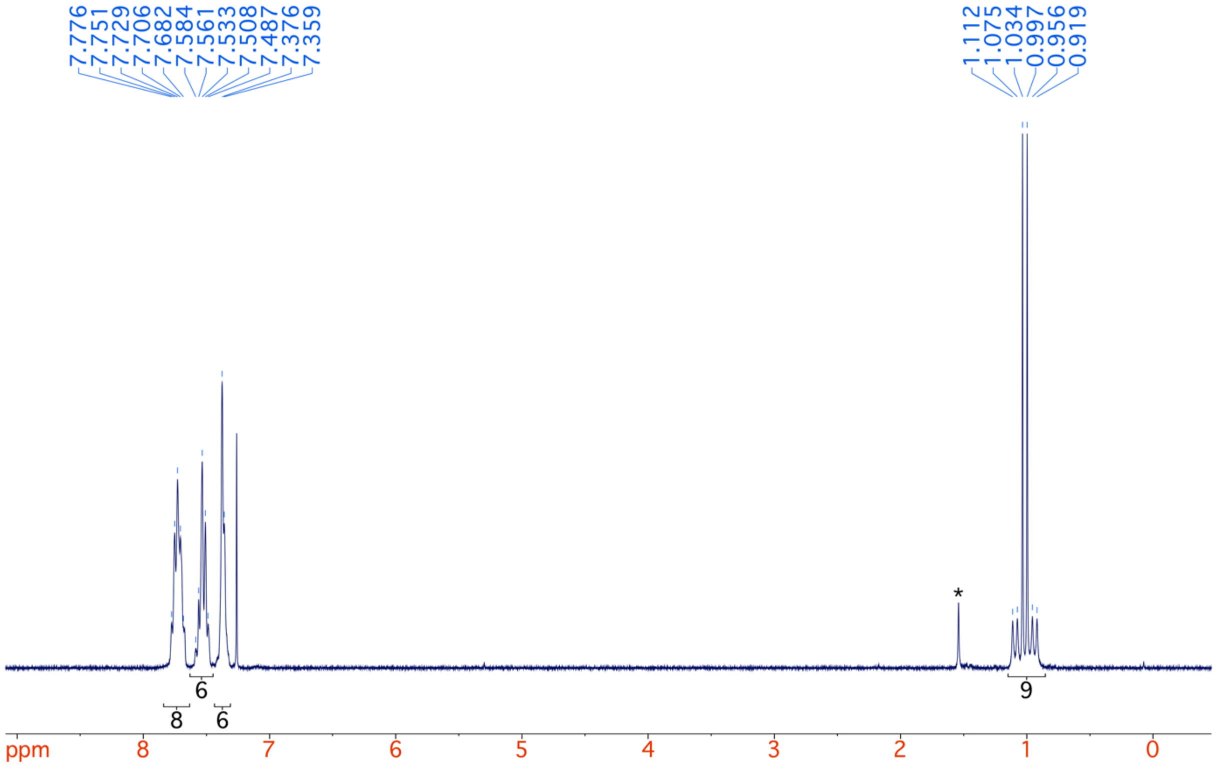

Figure S62. ^{1}H NMR spectrum in CDCl$_3$ of *trans*-[(Me$_3$P)ClPt(μ-2-C$_6$F$_4$PPh$_2$)$_2$Au] (*trans*-**8PtAu**). ^{1}H NMR signal of H$_2$O is marked with asterisk.

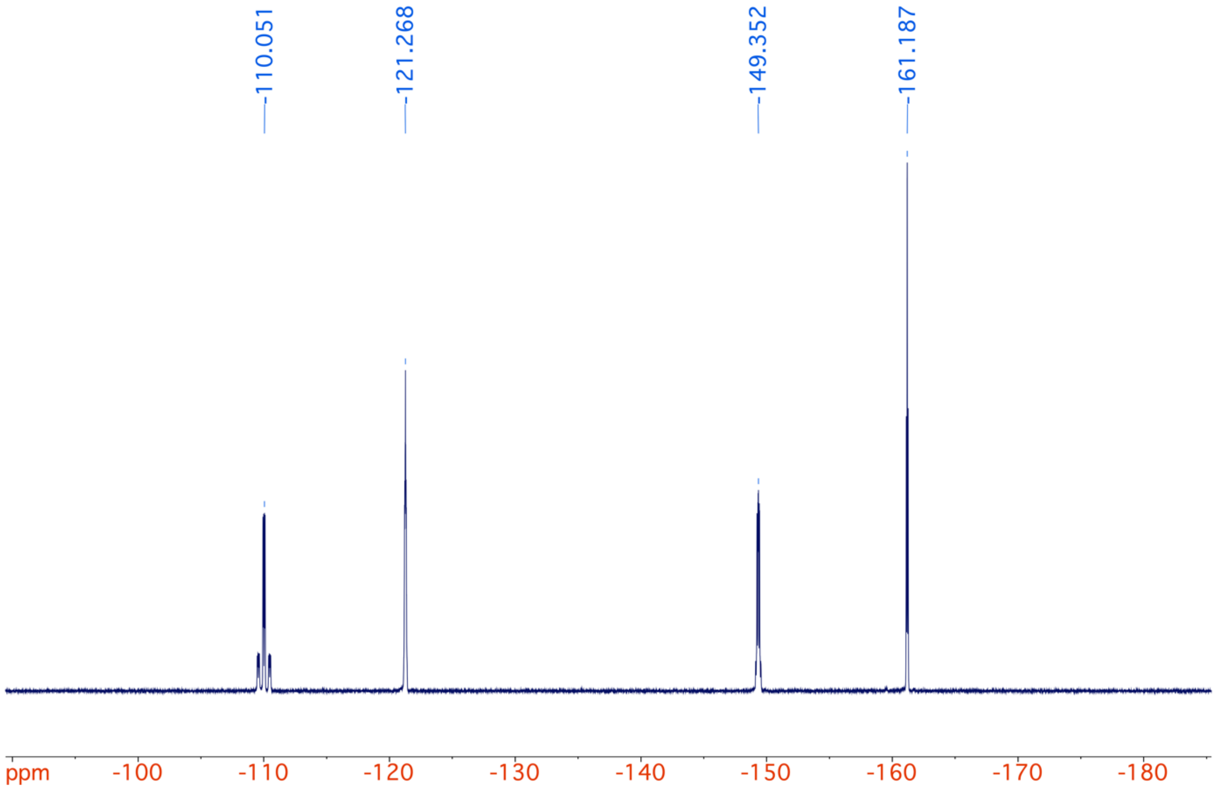

Figure S63. ^{19}F NMR spectrum in $CDCl_3$ of *trans*-[$(Me_3P)ClPt(\mu\text{-}2\text{-}C_6F_4PPh_2)_2Au$] (*trans*-**8PtAu**).

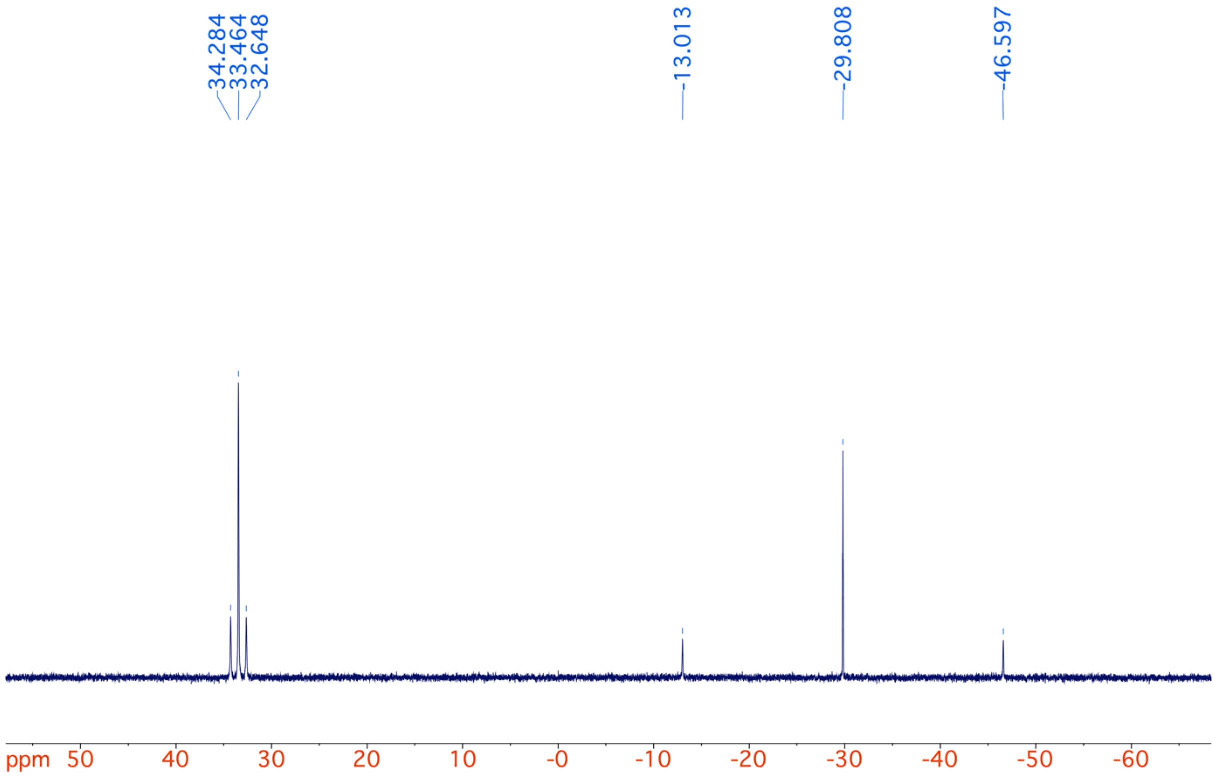

Figure S64. ^{31}P NMR spectrum in $CDCl_3$ of *trans*-[$(Me_3P)ClPt(\mu\text{-}2\text{-}C_6F_4PPh_2)_2Au$] (*trans*-**8PtAu**).

2. Parameters of data collection and structure refinement:

Experiments were carried out with Mo $K\alpha$ radiation using a Bruker APEX-II CCD. H-atom parameters were constrained.

	syn-**2Ni**[a]	*syn*-**2Pt**[a]·0.88(CH_2Cl_2)	**3Ni**[b]
Crystal data			
Chemical formula	$C_{42}H_{38}F_8NiP_4$	$C_{42}H_{38}F_8P_4Pt\cdot0.88(CH_2Cl_2)$	$C_{39}H_{29}F_8NiP_3$
M_r	877.31	1088.43	801.24
Crystal system, space group	Triclinic, $P\overline{1}$	Triclinic, $P\overline{1}$	Monoclinic, $P2_1/c$
Temperature (K)	142	200	199
a, b, c (Å)	11.2871 (18), 11.4750 (18), 15.760 (3)	11.225 (2), 11.790 (2), 17.452 (3)	15.8419 (13), 11.3677 (9), 20.8336 (17)
α, β, γ (°)	82.236 (4), 79.545 (4), 85.323 (3)	104.392 (4), 95.635 (5), 92.553 (4)	90, 107.418 (2), 90
V (Å^3)	1985.5 (5)	2220.5 (7)	3579.8 (5)
Z	2	2	4
μ (mm^{-1})	0.72	3.49	0.75
Crystal size (mm)	0.21 × 0.11 × 0.07	0.22 × 0.12 × 0.04	0.34 × 0.29 × 0.10
Data collection			
Absorption correction	Multi-scan *SADABS*	Part of the refinement model (ΔF) Parkin S,Moezzi B & Hope H, (1995) J. Appl. Cryst. 28, 53-56 Cubic fit to sin(theta)/lambda - 24 parameters	Multi-scan *SADABS*
T_{min}, T_{max}	0.707, 0.746	0.061, 0.446	0.626, 0.746
No. of measured, independent and observed [$I > 2\sigma(I)$] reflections	43895, 11564, 9682	11895, 11895, 10358	51334, 13076, 10562
R_{int}	0.028	0	0.061
$(\sin\theta/\lambda)_{max}$ (Å^{-1})	0.703	0.684	0.761
Refinement			
$R[F^2 > 2\sigma(F^2)]$, $wR(F^2)$, S	0.030, 0.077, 1.02	0.035, 0.084, 1.08	0.040, 0.119, 1.08
No. of reflections	11564	11895	13076
No. of parameters	533	531	463
No. of restraints	0	82	0
$\Delta\rho_{max}$, $\Delta\rho_{min}$ (e Å^{-3})	0.41, -0.25	1.90, -1.49	0.59, -0.56

Experiments were carried out with Mo $K\alpha$ radiation using a Bruker APEX-II CCD. Absorption was corrected for by multi-scan methods, *SADABS*. H-atom parameters were constrained.

	**3Pt**[b]	**3Pt**[b]	**4Ni**·0.63(CH_2Cl_2)
Crystal data			
Chemical formula	$C_{39}H_{29}F_8P_3Pt$	$C_{39}H_{29}F_8P_3Pt$	$C_{72}H_{40}F_{16}Ni_2P_4$·0.63($CH_2Cl_2$)
M_r	937.62	937.62	1504.25
Crystal system, space group	Monoclinic, $P2_1/c$	Monoclinic, *Pn*	Orthorhombic, *Ibca*
Temperature (K)	196	142	200
a, *b*, *c* (Å)	10.507 (4), 19.790 (7), 17.621 (6)	9.8140 (14), 8.0302 (11), 22.806 (3)	20.7594 (14), 23.9267 (16), 24.9979 (17)
α, β, γ (°)	90, 95.857 (8), 90	90, 98.824 (4), 90	90, 90, 90
V (Å^3)	3645 (2)	1776.0 (4)	12416.6 (15)
Z	4	2	8
μ (mm^{-1})	4.05	4.16	0.86
Crystal size (mm)	0.29 × 0.11 × 0.10	0.60 × 0.14 × 0.12	0.38 × 0.13 × 0.11
Data collection			
T_{min}, T_{max}	0.644, 0.747	0.653, 0.748	0.635, 0.748
No. of measured, independent and observed [$I > 2\sigma(I)$] reflections	61448, 16014, 12110	69294, 16529, 15506	50202, 9019, 6440
R_{int}	0.057	0.031	0.088
$(\sin \theta/\lambda)_{max}$ (Å^{-1})	0.807	0.847	0.703
Refinement			
$R[F^2 > 2\sigma(F^2)]$, $wR(F^2)$, S	0.028, 0.063, 0.99	0.018, 0.036, 0.80	0.047, 0.114, 1.04
No. of reflections	16014	16529	9019
No. of parameters	463	463	440
No. of restraints	0	2	1
$\Delta\rho_{max}$, $\Delta\rho_{min}$ (e Å^{-3})	1.10, -1.32	0.92, -0.72	0.60, -0.50
Absolute structure	–	Flack x determined using 6743 quotients [(I+)-(I-)]/[(I+)+(I-)] (Parsons, Flack and Wagner, Acta Cryst. B69 (2013) 249-259).	–
Absolute structure parameter	–	0.0090 (18)	–

Experiments were carried out with Mo $K\alpha$ radiation using a Bruker APEX-II CCD. Absorption was corrected for by multi-scan methods, *SADABS*.

	**4Pt**·0.52(CH_2Cl_2)	*trans*-**5NiCu**	*trans*-**5PtCu**
Crystal data			
Chemical formula	$C_{72}H_{40}F_{16}P_4Pt_2 \cdot 0.52(CH_2Cl_2)$	$C_{42}H_{38}ClCuF_8NiP_4$	$C_{42}H_{38}ClCuF_8P_4Pt$
M_r	1767.62	976.30	1112.68
Crystal system, space group	Orthorhombic, *Ibca*	Orthorhombic, *Pnma*	Orthorhombic, *Pnma*
Temperature (K)	73	150	200
a, b, c (Å)	20.946 (2), 23.879 (3), 25.191 (3)	12.1319 (13), 23.748 (2), 14.1989 (15)	12.2430 (6), 24.1559 (12), 14.1793 (7)
V (Å^3)	12600 (2)	4090.9 (7)	4193.4 (4)
Z	8	4	4
μ (mm^{-1})	4.68	1.27	4.12
Crystal size (mm)	0.58 × 0.12 × 0.04	0.25 × 0.07 × 0.04	0.20 × 0.07 × 0.04
Data collection			
T_{min}, T_{max}	0.536, 0.747	0.676, 0.747	0.670, 0.748
No. of measured, independent and observed [$I > 2\sigma(I)$] reflections	54840, 5879, 4456	23892, 5035, 3328	52712, 6231, 5244
R_{int}	0.061	0.108	0.075
$(\sin \theta/\lambda)_{max}$ (Å^{-1})	0.606	0.660	0.703
Refinement			
$R[F^2 > 2\sigma(F^2)]$, $wR(F^2)$, S	0.027, 0.072, 1.03	0.038, 0.070, 0.87	0.028, 0.059, 1.05
No. of reflections	5879	5035	6231
No. of parameters	412	280	281
No. of restraints	133	15	24
H-atom treatment	H-atom parameters constrained	H atoms treated by a mixture of independent and constrained refinement	H atoms treated by a mixture of independent and constrained refinement
	$w = 1/[\sigma^2(F_o^2) + (0.0335P)^2 + 42.8016P]$ where $P = (F_o^2 + 2F_c^2)/3$	$w = 1/[\sigma^2(F_o^2) + (0.0121P)^2]$ where $P = (F_o^2 + 2F_c^2)/3$	$w = 1/[\sigma^2(F_o^2) + (0.012P)^2 + 6.913P]$ where $P = (F_o^2 + 2F_c^2)/3$
$\Delta\rho_{max}$, $\Delta\rho_{min}$ (e Å^{-3})	1.17, -1.23	0.82, -0.73	1.31, -1.32

Experiments were carried out with Mo *K*α radiation using a Bruker APEX-II CCD.

	cis-**5PtCu**	*trans*-**5PtAu**	*cis*-**5PtAg**
Crystal data			
Chemical formula	$C_{42}H_{38}ClCuF_8P_4Pt$	$C_{42}H_{38}AuClF_8P_4Pt$	$C_{42}H_{38}AgClF_8P_4Pt$
M_r	1112.68	1246.11	1157.01
Crystal system, space group	Triclinic, $P\bar{1}$	Orthorhombic, *Pnma*	Triclinic, $P\bar{1}$
Temperature (K)	200	174	199
a, *b*, *c* (Å)	12.0018 (16), 12.4231 (16), 14.5372 (19)	12.2048 (11), 24.214 (2), 14.2180 (13)	12.125 (4), 12.572 (4), 14.362 (4)
α, β, γ (°)	91.254 (3), 97.042 (3), 108.219 (3)	90, 90, 90	90.949 (6), 97.102 (6), 108.914 (6)
V (Å^3)	2039.1 (5)	4201.8 (7)	2051.5 (11)
Z	2	4	2
μ (mm^{-1})	4.24	7.10	4.17
Crystal size (mm)	0.14 × 0.08 × 0.07	0.14 × 0.06 × 0.04	0.13 × 0.10 × 0.05
Data collection			
Absorption correction	Multi-scan *SADABS*	Multi-scan *SADABS*	Part of the refinement model (Δ*F*) Parkin S,Moezzi B & Hope H, (1995) J. Appl. Cryst. 28, 53-56 Cubic fit to sin(theta)/lambda - 24 parameters
T_{min}, T_{max}	0.662, 0.747	0.616, 0.746	0.747, 0.900
No. of measured, independent and observed [$I > 2\sigma(I)$] reflections	77846, 21278, 17952	105427, 7776, 6336	63676, 17878, 14913
R_{int}	0.066	0.068	0.062
$(\sin \theta/\lambda)_{max}$ (Å^{-1})	0.856	0.756	0.807
Refinement			
$R[F^2 > 2\sigma(F^2)]$, $wR(F^2)$, S	0.031, 0.072, 1.00	0.023, 0.044, 1.05	0.032, 0.075, 1.00
No. of reflections	21278	7776	17878
No. of parameters	531	281	522
No. of restraints	0	36	0
H-atom treatment	H-atom parameters constrained	H atoms treated by a mixture of independent and constrained refinement	H-atom parameters constrained
$\Delta\rho_{max}$, $\Delta\rho_{min}$ (e Å^{-3})	1.30, -1.23	0.85, -0.69	1.61, -1.78

Experiments were carried out with Mo *K*α radiation using a Bruker APEX-II CCD. Absorption was corrected for by multi-scan methods, *SADABS*. H-atom parameters were constrained.

	cis-**6PtCu**	*cis*-**6PtAg-dimer**	*cis*-**6PtAu**
Crystal data			
Chemical formula	$C_{39}H_{29}ClCuF_8P_3Pt$	$C_{78}H_{58}Ag_2Cl_2F_{16}P_6Pt_2$	$C_{39}H_{29}AuClF_8P_3Pt$
M_r	1036.61	2161.88	1170.04
Crystal system, space group	Monoclinic, $P2_1$	Monoclinic, $P2_1/c$	Monoclinic, $P2_1$
Temperature (K)	200	200	150
a, b, c (Å)	11.4906 (13), 11.6960 (13), 13.9653 (15)	11.4489 (10), 23.4326 (19), 28.748 (3)	11.4886 (12), 11.6926 (12), 13.9750 (15)
β (°)	94.724 (3)	98.200 (2)	94.642 (2)
V (Å^3)	1870.5 (4)	7633.5 (11)	1871.1 (3)
Z	2	4	2
μ (mm^{-1})	4.57	4.44	7.92
Crystal size (mm)	0.19 × 0.10 × 0.05	0.11 × 0.10 × 0.08	0.25 × 0.25 × 0.11
Data collection			
T_{min}, T_{max}	0.576, 0.746	0.656, 0.747	0.584, 0.747
No. of measured, independent and observed [$I > 2\sigma(I)$] reflections	49838, 13607, 12950	217222, 29940, 21428	34928, 15902, 14905
R_{int}	0.040	0.094	0.025
$(\sin \theta/\lambda)_{max}$ (Å^{-1})	0.759	0.777	0.807
Refinement			
$R[F^2 > 2\sigma(F^2)]$, $wR(F^2)$, S	0.020, 0.044, 0.95	0.036, 0.071, 1.01	0.022, 0.043, 1.02
No. of reflections	13607	29940	15902
No. of parameters	481	963	482
No. of restraints	1	0	1
$\Delta\rho_{max}$, $\Delta\rho_{min}$ (e Å^{-3})	1.15, -0.81	1.01, -0.88	0.91, -0.63
Absolute structure	Flack x determined using 5917 quotients [(I+)-(I-)]/[(I+)+(I-)] (Parsons and Flack (2004), Acta Cryst. A60, s61).	–	Refined as an inversion twin.
Absolute structure parameter	0.007 (2)	–	0.209 (3)

Experiments were carried out with Mo $K\alpha$ radiation. H-atom parameters were constrained.

	trans-**6PtAu**·0.722(CH_2Cl_2)	*cis-trans*-**7NiAu**	*trans*-**7PtAu**
Crystal data			
Chemical formula	$C_{39}H_{29}AuClF_8P_3PT\cdot0.722(CH_2Cl_2)$	$C_{39}H_{29}AuClF_8NiP_3$	$C_{39}H_{29}AuClF_8P_3Pt$
M_r	1231.40	1033.66	1170.04
Crystal system, space group	Tetragonal, $I4_1/a$	Monoclinic, $P2_1$	Monoclinic, $C2/c$
Temperature (K)	100	180	199
a, b, c (Å)	38.469 (4), 38.469 (4), 10.9570 (13)	10.6153 (4), 12.6351 (4), 14.4750 (6)	43.749 (11), 8.3652 (18), 25.706 (7)
α, β, γ (°)	90, 90, 90	90, 92.251 (3), 90	90, 125.560 (11), 90
V (Å^3)	16215 (4)	1939.97 (12)	7653 (3)
Z	16	2	8
μ (mm^{-1})	7.41	4.52	7.74
Crystal size (mm)	0.18 × 0.14 × 0.13	0.20 × 0.10 × 0.02	0.16 × 0.05 × 0.03
Data collection			
Diffractometer	Bruker *APEX*-II CCD	Stoe *IPDS*2T	Bruker *APEX*-II CCD
Absorption correction	Multi-scan *SADABS*	Integration *X-SHAPE*	Part of the refinement model (ΔF) Parkin S,Moezzi B & Hope H, (1995) J. Appl. Cryst. 28, 53-56 Cubic fit to sin(theta)/lambda - 24 parameters
T_{min}, T_{max}	0.578, 0.746	0.596, 0.881	0.125, 0.174
No. of measured, independent and observed [$I > 2\sigma(I)$] reflections	259181, 11815, 11132	18534, 8549, 7541	6875, 6875, 5983
R_{int}	0.040	0.048	0.066
$(\sin \theta/\lambda)_{max}$ (Å^{-1})	0.703	0.643	0.606
Refinement			
$R[F^2 > 2\sigma(F^2)]$, $wR(F^2)$, S	0.023, 0.046, 1.29	0.032, 0.069, 1.06	0.067, 0.139, 1.24
No. of reflections	11815	8549	6875
No. of parameters	519	482	489
No. of restraints	7	1	0
	$w = 1/[\sigma^2(F_o^2) + (0.0049P)^2 + 84.8293P]$ where $P = (F_o^2 + 2F_c^2)/3$	$w = 1/[\sigma^2(F_o^2) + (0.025P)^2 + 3.6473P]$ where $P = (F_o^2 + 2F_c^2)/3$	$w = 1/[\sigma^2(F_o^2) + 412.8095P]$ where $P = (F_o^2 + 2F_c^2)/3$
$\Delta\rho_{max}$, $\Delta\rho_{min}$ (e Å^{-3})	0.95, -0.88	1.30, -0.83	1.84, -3.25
Absolute structure	-	Refined as an inversion twin.	–
Absolute structure parameter	-	0.444 (7)	–

For all structures: $Z = 4$. Experiments were carried out with Mo $K\alpha$ radiation. H-atom parameters were constrained. Crystals of **A** obtained from dichloromethane/methanol.

	trans-**8NiCu**·2(C_3H_6O)	*trans*-**8PtAu**	**A**
Crystal data			
Chemical formula	$C_{42}H_{35}ClCuF_8NiOP_3{\cdot}C_3H_6O$	$C_{39}H_{29}AuClF_8P_3Pt$	$C_{27}H_{37}AuClF_4P_4Pt{\cdot}Cl$
M_r	1016.39	1170.04	1024.40
Crystal system, space group	Monoclinic, $P2_1/n$	Monoclinic, $P2_1/n$	Monoclinic, $P2_1/c$
Temperature (K)	200	100	200
a, b, c (Å)	11.6546 (3), 12.8205 (5), 30.3555 (8)	13.9841 (8), 18.9077 (11), 14.8806 (8)	11.5120 (7), 15.9462 (9), 18.463 (1)
β (°)	98.376 (2)	90.521 (1)	99.449 (3)
V (Å^3)	4487.3 (2)	3934.4 (4)	3343.3 (3)
μ (mm^{-1})	1.13	7.53	8.95
Crystal size (mm)	0.30 × 0.15 × 0.10	0.19 × 0.07 × 0.05	0.38 × 0.08 × 0.04
Data collection			
Diffractometer	Stoe *IPDS*2T	Bruker *APEX*-II CCD	Bruker *APEX*-II CCD
Absorption correction	Integration *X-SHAPE*	Multi-scan *SADABS*	Multi-scan *SADABS*
T_{min}, T_{max}	0.778, 0.934	0.555, 0.746	0.490, 0.745
No. of measured, independent and observed [$I > 2\sigma(I)$] reflections	52032, 8322, 6968	43319, 14238, 11484	54500, 7070, 6203
R_{int}	0.040	0.031	0.061
$(\sin \theta/\lambda)_{max}$ (Å^{-1})	0.606	0.756	0.632
Refinement			
$R[F^2 > 2\sigma(F^2)]$, $wR(F^2)$, S	0.026, 0.061, 1.03	0.025, 0.052, 1.02	0.024, 0.059, 1.04
No. of reflections	8322	14238	7070
No. of parameters	590	500	361
No. of restraints	7	0	0
$\Delta\rho_{max}$, $\Delta\rho_{min}$ (e Å^{-3})	0.30, -0.22	0.92, -1.09	2.02, -1.81

Experiments were carried out at 200 K with Mo $K\alpha$ radiation using a Bruker APEX-II CCD. H-atom parameters were constrained. Crystals of **B1** and **B2**$\cdot CH_2Cl_2$ obtained from dichloromethane/methanol.

	**B1**	**B2**$\cdot CH_2Cl_2$
Crystal data		
Chemical formula	$C_{54}H_{30}AuF_{12}P_3Pt$	$C_{55}H_{32}AuCl_2F_{12}P_3Pt$
M_r	1391.74	1476.67
Crystal system, space group	Monoclinic, $P2_1/n$	Triclinic, $P\bar{1}$
a, b, c (Å)	11.5255 (17), 17.970 (3), 22.558 (3)	11.4606 (10), 14.8641 (14), 15.5004 (14)
α, β, γ (°)	90, 99.052 (5), 90	79.873 (4), 73.992 (5), 80.148 (4)
V (Å^3)	4613.9 (12)	2477.5 (4)
Z	4	2
μ (mm^{-1})	6.40	6.07
Crystal size (mm)	0.23 × 0.17 × 0.07	0.21 × 0.16 × 0.08
Data collection		
Absorption correction	Part of the refinement model (ΔF) Parkin S, Moezzi B & Hope H, (1995) J. Appl. Cryst. 28, 53-56 Cubic fit to sin(theta)/lambda - 24 parameters	Multi-scan *SADABS*
T_{min}, T_{max}	0.642, 0.782	0.560, 0.748
No. of measured, independent and observed [$I > 2\sigma(I)$] reflections	11123, 11123, 8895	55465, 13582, 11845
R_{int}	0	0.085
$(\sin \theta/\lambda)_{max}$ (Å^{-1})	0.661	0.693
Refinement		
$R[F^2 > 2\sigma(F^2)]$, $wR(F^2)$, S	0.041, 0.107, 1.08	0.043, 0.110, 1.05
No. of reflections	11123	13582
No. of parameters	641	666
No. of restraints	0	6
$\Delta\rho_{max}$, $\Delta\rho_{min}$ (e Å^{-3})	1.81, -1.11	1.94, -1.60

3. Discussion of molecular structures of syn-2M[a], 3M[b] and 4M (M = Ni, Pt):

In their crystal structure, all six complexes exhibit approximately square-planar geometry about the nickel and platinum atoms. For *syn*-**2M[a]**, the M-C- and M-P-bond lengths are similar to the corresponding *trans*-**1M** complexes. The C-M-C angle shows approximate linearity (174.74(6)°, 174.83(14)°, respectively), whereas the P-M-P angle is significantly bent out of linearity by more than 10° (166.66(2)°, 169.95(4)°, respectively). This distortion is presumably caused by steric effects of the *ortho*-PPh_2 groups. Similar behaviour was detected for the homologue *syn*-**2Pd[a]**.[S1] Complexes of the type **3M[b]** exhibit a *cis*-configuration about the transition metals. Complex [$(Ph_3P)Pd(\kappa^2$-2-$C_6F_4PPh_2)(\kappa C$-2-$C_6F_4PPh_2)$] is the only crystallographically characterized complex with a similar arrangement about the transition metal core as observed in compounds of type **3M[b]**.[S2] The deviation of the P1-M1 and P3-M1 bond lengths within a complex is only approximately 0.03 Å for **3Ni[b]** and **3Pt[b]**. The P2-M1 distance of the dangling 2-$C_6F_4PPh_2$ group shows a significant difference between **3Ni[b]** (2.7770(4) Å) and **3Pt[b]** (3.205(1) Å) by approximately 0.4 Å. The P2-Ni1 distance is significant longer as the sum of the covalent radii (2.31(5) Å)[S3] and shorter as the sum of the van der Waals radii (3.95 Å).[S4] Therefore the Ni coordination sphere can be described as slightly over capped. In contrast to **3Ni[b]**, the electron pair at P2 in **3Pt[b]** is pointing away from the transition metal centre. The characteristics of the chelating ligand in the complexes of the type **3M[b]** and **4M** are in good agreement to the corresponding *trans*-**1M** complexes. The bridging 2-$C_6F_4PPh_2$-ligands exhibit a *cis*-C-M-C arrangement about the transition metal. The Pt···Pt distance in **4Pt** is significantly shorter as in the corresponding complex [$Pt_2(\kappa^2$-2-$C_6H_4PPh_2)_2(\mu$-2-$C_6H_4PPh_2)_2$] by approximately 0.06 Å.

Table S1. Selected interatomic separations (Å) and angles (deg) in *syn*-**2Ni[a]**, *syn*-**2Pt[a]**, **3Ni[b]**, **3Pt[b]**, **4Ni** and **4Pt**.

	syn-**2Ni[a]**	*syn*-**2Pt[a]**	**3Ni[b]**	**3Pt[b§]**	**4Ni**	**4Pt**
M1···M1*					3.2958(7)	3.3245(8)
M1–P1	3.3420(6)	3.443(1)	2.2174(4)	2.3086(7)	2.2407(6)	2.3117(10)
M1–P2(*)	3.3094(5)	3.443(1)	2.7770(4)	3.205(1)	2.2150(6)	2.2939(11)
M1–P3	2.2033(5)	2.3034(10)	2.1849(4)	2.2781(9)		
M1–P4	2.1829(5)	2.2950(10)				
M1–C1	1.9449(13)	2.079(4)	1.9427(13)	2.064(2)	1.956(2)	2.078(4)
M1–C7	1.9406(14)	2.089(3)	1.9105(13)	2.062(2)	1.915(2)	2.061(4)
C1–M1–P4	90.11(4)	90.28(10)				
C7–M1–P3	89.76(4)	89.21(10)	90.94(4)	93.26(6)		
C1–M1–C7	174.74(6)	174.83(14)	96.12(5)	96.45(8)	90.77(9)	92.07(16)
P3–M1–P4	166.66(2)	169.95(4)				
C1–M1–P3(P2*)	88.50(4)	89.32(10)	163.98(4)	166.89(6)	166.83(7)	165.70(11)
C7–M1–P4(P2*)	89.76(4)	90.30(10)			91.39(7)	92.15(11)
P1–M1–C7			168.58(4)	164.40(6)	157.06(7)	156.84(11)
P1–M1–P3			100.18(2)	102.16(3)		
P1–M1–C1			72.47(4)	68.71(6)	72.44(7)	69.41(12)

* symmetry operation: -x+1, -y+1/2, z+0; [§] **3Ni[b]** and **3Pt[b]** crystallize isomorphos (monoclinic, space group *$P2_1/c$*). **3Pt[b]** was found to also crystalize in space group *P*n with conformational identical molecule in the asymmetric unit. Details are given in the supporting information.

4. Discussion of molecular structures of *trans*-5MCu (M = Ni, Pd, Pt):

In the complex series of *trans*-**5MCu**, the M-C1 bond length is increasing in the order Ni < Pd < Pt by approximately 0.13 Å and the M-P2/3 bond length are increasing in the order Ni < Pt < Pd by approximately 0.17 Å. This change in trends was investigated by comparing similar complexes bearing a 4-coordinated d^8-metal (M = Ni, Pd, Pt) binding to either an aromatic six-membered ring or a PMe_3 group (Chart S1). The respectiv bonds (marked in red) where satistically analysed from this Cambridge structre database (CSD) search.[S5] The following M-C bonding trend was found 1.909(31) Å (Ni) < 2.004(33) Å (Pd) < 2.017(39) Å (Pt), whereas the M-P bonding trend is 2.187(26) Å (Ni) < 2.287(31) Å (Pt) < 2.304(30) Å (Pd). Our findings are in aligment with the data reported in the literature.

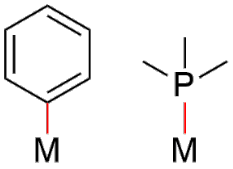

Chart S1. CSD search input.

Table S2. Selected interatomic separations (Å) and angles (deg) in *trans*-**5NiCu**, *trans*-**5PtCu**, *trans*-**5PtAu**, *cis*-**5PtCu** and *cis*-**5PtAg**.

	trans-**5NiCu**	*trans*-**5PtCu**	*trans*-**5PtAg**	*trans*-**5PtAu**	*cis*-**5PtCu**	*cis*-**5PtAg**
M1···M′1	2.8197(6)	2.8221(5)	2.9172(9)	2.8922(3)	2.8221(3)	2.9454(7)
M1–P1/P2/P4	2.2163(10)	2.3066(10)	2.3173(18)	2.3111(8)	2.3015(6)	2.3036(9)
M1–P3	2.2301(10)	2.3199(10)	2.3161(18)	2.3091(8)	2.3072(6)	2.3100(9)
M1–C1	1.936(2)	2.069(3)	2.068(4)	2.068(2)	2.0984(17)	2.113(2)
M1–C7					2.0797(18)	2.099(2)
M′1–P1	2.2839(7)	2.3151(8)	2.4613(15)	2.3356(6)	2.2410(5)	2.4674(8)
M′1–P2					2.2491(6)	2.4394(8)
M′1–Cl1	2.2791(9)	2.2960(11)	2.5387(18)	2.6777(9)	2.254(8)	2.4614(10)
C1–M1–P3	92.23(7)	92.21(7)	92.35(13)	92.48(6)	89.47(5)	88.60(6)
C7–M1–P1/P4					88.65(5)	88.60(7)
C1–M1–C7/C1*	169.94(15)	172.72(15)	171.9(3)	173.25(12)	89.22(7)	90.19(8)
P1/P2/P4–M1–P3	166.72(4)	167.26(4)	167.79(6)	167.66(3)	92.77(2)	92.88(3)
C1–M1–P1/P2/P4	86.80(7)	87.21(7)	87.01(13)	87.09(6)	177.74(5)	178.15(5)
C7–M1–P3					167.78(5)	167.92(6)
P1–M′1–Cl1	107.77(2)	107.18(2)	107.38(3)	103.40(2)	116.0(2)	113.24(2)
P2–M′1–Cl1					122.8(2)	128.12(2)
P1–M′1–P2/P1*	143.32(4)	144.05(4)	145.10(6)	153.14(3)	112.69(2)	109.66(3)

* symmetry operation: x, -y+1/2, z

5. NMR Calculations:

To address the issue of smaller magnitude in ΔδP in the ^{31}P NMR spectra of the series *syn*-**2M^a** vs. *trans*-**5MCu** (M = Ni, Pd, Pt), we performed ^{31}P NMR calculations (isotropic total nmr shielding tensor (ppm)) for the series *trans*-**5MCu** (M = Ni (317.6), Pd (320.4), Pt (324.2)) on the structures obtained from single crystal X-ray diffraction after H atom position optimisation. A second series was calculated were CuCl was removed from the *trans*-**5MCu** structures, without any relaxation of the complex structures (this corresponds to *syn*-**2M**^{a} (M = Ni (312.9), Pd (314.8), Pt (315.5))). With this approach we exclude any structural differences between *syn*-**2M**^{a} and *trans*-**5MCu**, which could have an impact on the ^{31}P NMR shift. Our results have shown that for the heterobimetallic complexes the deviation of the ^{31}P NMR shift is 6.6 ppm, whereas for the series *syn*-**2M**^{a} it is 2.6 ppm. The calculations support that including a second metal (and therefore metal-metal interactions) has an additional impact on the ^{31}P NMR shift. Furthermore, it was calculated that the nmr shielding is increaseing in the order *trans*-**5NiCu** < *trans*-**5PdCu** < *trans*-**5PtCu**, as observed experimentally.
The isotropic total nmr shielding tensor (ppm) of the PPh_2 units were calculated using ADF 2019.303 using the revPBE functional, ZORA spin-orbit relativistic, TZ2P basis set for C, H, F and Cl and QZ4P for Ni, Pd, Pt, Cu and P and COSMO solvation model (DCM).

6. Discussion of molecular structures of *cis*-**6PtCu** and *cis*-**6PtAg-dimer**:

Compound *cis*-**6PtAg** does show dimerisation due to Ag(μ-Cl)$_2$Ag bridges (*cis*-**6PtAg-dimer**), whereas *cis*-**6PtCu** appears to stay monomeric, which is supported by quantum chemical calculations were *cis*-**6PtCu** is approximately 1.7 kcal/mol more stable over *cis*-**6PtCu-dimer**. The dimeric structure of *cis*-**6PtAg-dimer** was confirmed by mass spectroscopy (ESI-MS (m/z): 2125.99 $[M-Cl+H]^+$). The dimerization energy of *cis*-**6PtAg** versus *cis*-**6PtAg-dimer** is calculated to be -1.9 kcal mol^{-1}. For *cis*-**6PtCu** the molecular weigth was measured to be ESI-MS (m/z): 1001.04 $[M-Cl+H]^+$ and the ^{31}P NMR characteristics of the Cu bound P atom (16.5 ppm (m, $J_{Pt,P}$ = 340 Hz)) are well in accord with the monomeric complex [(dppe)Pt(*κ*C-2-$C_6F_4PPh_2$)(*μ*-2-$C_6F_4PPh_2$)CuCl] (13.6 ppm (br. m, J_{PtP} = 360 Hz)).[S6] However, a variety of R_3P-Cu(μ-Cl)$_2$Cu-PR_3 and R_3P-Ag(μ-Cl)$_2$Ag-PR_3 bonding modes are given in the literature.[S7] The angles around the Cu center always sum up to approximately > 358°, whereas for the Ag centre a higher distortion of the trigonal planar coordination sphere is present (∑(∡ around Ag) > 350°). The angle around the Ag1 and Ag2 atoms in *cis*-**6PtAg-dimer** sum up to 350.0° and 351.1°, respectively, which is in good agreement with the literature. Bond lenths and angles around the Pt atoms in *cis*-**6PtCu** and *cis*-**6PtAg-dimer** are in good comparison with the starting material **3Pt**[b] and support the finding via ^{31}P NMR spectroscopy. The P3-Cu1-Cl1 angle is bend out of linearity by approximately 21° caused by repulsive interaction between the chloride atom, the PMe_3 ligand and PPh_2 unit and attractive Pt···Cu interaction (*vide infra*). This observation is similar for the complex [(dppe)Pt(*κ*C-2-$C_6F_4PPh_2$)(*μ*-2-$C_6F_4PPh_2$)CuCl] where the P3-Cu1-Cl1 angle is bend by approximately 30°.[S6]

Table S3. Selected interatomic separations (Å) and angles (deg) in *cis*-**6PtCu**, *cis*-**6PtAg-dimer**, *cis*-**6PtAu** and *trans*-**6PtAu**.

	cis-**6PtCu**	*cis*-**6PtAg-dimer**	*cis*-**6PtAu**	*trans*-**6PtAu**
M1···M′1	2.8826(5)	3.3287(7)	3.1224(3)	2.9485(4)
M2···M′2		2.9792(7)		
M1–P1	2.3229(7)	2.3178(7)	2.3237(9)	2.3214(8)
M2–P4		2.3048(8)		
M1–P3	2.3229(7)	2.2894(8)	2.2953(9)	2.2898(8)
M2–P6		2.2901(9)		
M1–C1	2.058(3)	2.070(3)	2.056(3)	2.080(3)
M2–C40		2.063(3)		
M1–C7	2.059(3)	2.059(3)	2.063(4)	2.076(3)
M2–C46		2.064(3)		
M′1–P2	2.1679(9)	2.3761(8)	2.2312(10)	2.2300(7)
M′2–P5		2.4008(8)		
M′1–Cl1	2.1679(9)	2.4924(7)	2.3036(10)	2.3041(7)
M′2–Cl2		2.5999(8)		
C1–M1–P3	170.07(8)	164.55(8)	169.47(10)	98.80(9)
C40–M2–P6		171.04(8)		
C7–M1–P1	160.43(9)	159.55(8)	160.67(10)	167.43(12)
C46–M2–P4		162.83(8)		
C1–M1–C7	92.03(11)	93.04(11)	92.54(14)	100.15(8)
C40–M2–C46		95.44(11)		
P1–M1–P3	105.32(3)	102.29(3)	104.76(3)	165.85(3)
P4–M2–P6		103.73(3)		
C1–M1–P1	68.96(8)	69.11(8)	68.83(10)	68.16(9)
C40–M2–P4		69.11(8)		
C7–M1–P3	94.16(8)	92.65(8)	94.42(10)	92.31(8)
C46–M2–P6		90.85(8)		
P2–M′1–Cl1	159.06(4)	135.09(3)	169.52(4)	173.21(2)
P5–M′2–Cl2		126.08(3)		

7. Discussion of molecular structures of *cis*-**6PtAu**, *trans*-**6PtAu**, *trans*-**7PtAu** and *trans*-**8PtAu**:

All complexes geometries about the Pt and Au atoms are approximately square-planar and linear. Contingent by the *cis-trans*-isomerisation, the main difference between *cis*-**6PtAu** and *trans*-**6PtAu** appears in the torsion angle C2-P2-Au1-Cl1 (*cis*-**6PtAu**: 120.6(3)°; *trans*-**6PtAu**: 161.8(4)°) with an deviation of approximately 40°. In *trans*-**6PtAu,** the P2-Au1-Cl1 axis is almost co-planar to the C_6F_4P plane at P2 (deviating by approximately 5.85(6)°), whereas for *cis*-**6PtAu** the axis is significantly out of plane (deviation of about 31.17(3)°) presumably caused by repulsiv interactions between the Cl1 atom and the phenyl group at P2. This feature leads in a significant shorter Pt1···Au1 separation in *trans*-**6PtAu** by approximately 0.17 Å.

Trans-**7PtAu** and *trans*-**8PtAu** reveal a *trans* configuration of the µ-2-$C_6F_4PPh_2$ ligands with a head-to-tail arrangement in *trans*-**7PtAu** and a head-to-head arrangement in *trans*-**8PtAu**. The Pt1···Au1 separations in *trans*-**7PtAu** and *trans*-**8PtAu** are significantly shorter as observed for *trans*-**5PtAu** by approximately 0.04 and 0.09 Å, respectively. The Pt1-P3 distances in both complexes are significantly shorter as in complex *trans*-**5PtAu** (*trans*-**7PtAu**: 0.07 and *trans*-**8PtAu**: 0.10 Å), respectively. The Au1-P2 and Au1-C7 distances in *trans*-**7PtAu** are in good agreement with distances found in the gold dimer [Au_2(µ-2-$C_6F_4PPh_2$)$_2$] with a similar linear P-Au-C arrangement.[S7] The geometry parameters for the P-Au^+-P arrangement in *trans*-**8PtAu** are similar to the once found in the homologue complex *trans*-[(Me_3P)ClPd(µ-2-$C_6F_4PPh_2$)$_2$Au][S1] and [F_3Sb(µ-2-$C_6H_4PPh_2$)$_2$Au]SbF_6.[S9]

8. Discussion of molecular structures of *trans*-8NiCu·acetone and *cis-trans*-7NiAu:

The molecular structure of *cis-trans*-**7NiAu** shows the expected *cis* orientation of the µ-2-$C_6F_4PPh_2$ ligands in a head-to-tail arrangement. The PMe_3 ligand is located *trans* to the PPh_2 unit at the nickel centre and is in agreement with the NMR spectroscopic findings. A similar complex geometry was found in the homologue compound *cis*-[(Ph_3P)ClPd(µ-2-$C_6F_4PPh_2$)$_2$Au].[S1] Compound *trans*-**8NiCu**·acetone exhibits a distorted square-planar geometry at nickel and a strong distorted tetrahedral geometry at copper. The Ni1-Cl1 distance is 2.2609(6) Å and elongated by approximately 0.04 Å in comparison to similar Ni-Cl bonds *trans* located to a PR_3 unit.[S10] The sum of the covalent radii of Ni and Cl is reported to be 2.26(6) Å, whereas for Cu and Cl it is 2.34(6) Å.[S3] The Cu1-Cl1 distance is 2.46 Å and therefore obove the sum of the covalent radii, whereas the Ni1-Cl1 distance is 2.26 Å and lays on the upper edge of the sum of the covalent radii. The P1-Cu1-P2 angle with 143.26(2)° is midway between ideal linearity and an ideal tetrahedral angle.

9. **Structural overlay of *trans*-5NiCu, *trans*-5PdCu and *trans*-5PtCu and molecular structures of A and B:**

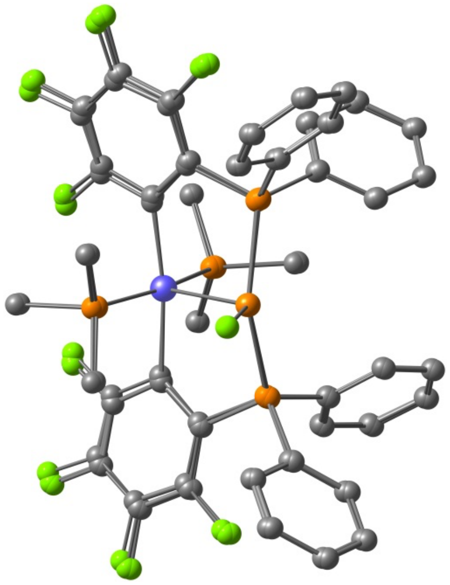

Figure S65. X-ray structure overlay of *trans*-**5NiCu**, *trans*-**5PdCu** and *trans*-**5PtCu**. Hydrogen atomes are omitted for clarity (RMSD were calculated by including M, M', Cl, P1, and P1*; $RMSD_{Ni\text{-}Pd}$ = 0.0217 Å, $RMSD_{Ni\text{-}Pt}$ = 0.0416 Å, $RMSD_{Pd\text{-}Pt}$ = 0.0246 Å).

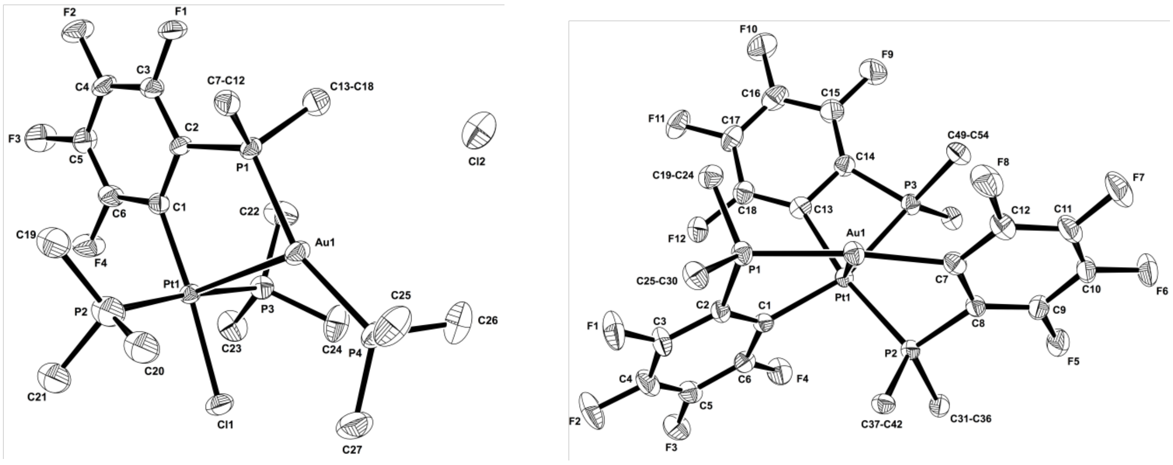

Figure S66. Molecular structures of two obtained products (**A** (left), **B** (right)) after decomposition of *trans*-**7PtAu** in toluene. **B** apear in two modifications. Ellipsoids are shown at 50% probability level. Hydrogen atoms and solvent molecules are omitted and only the *ipso*-carbons of the PPh_2-groups are depicted for clarity.

10. Additional ELF:

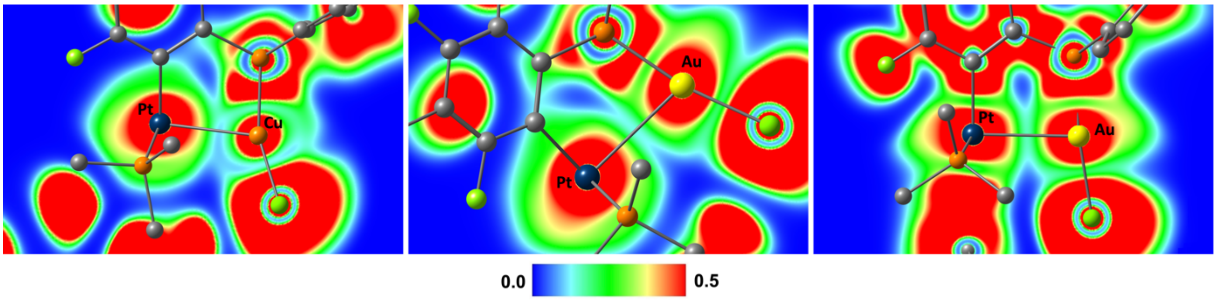

Figure S67. Electron localization function (ELF) of *cis*-**6PtCu** (left), *cis*-**6PtAu** (middle) and *trans*-**6PtAu** (right). Color ranges from ELF = 0.0 to 0.5 a.u.; Color code: blue – strong delocalization, red – electron-gas-like pair probability. Hydrogen atoms are omitted for clarity.

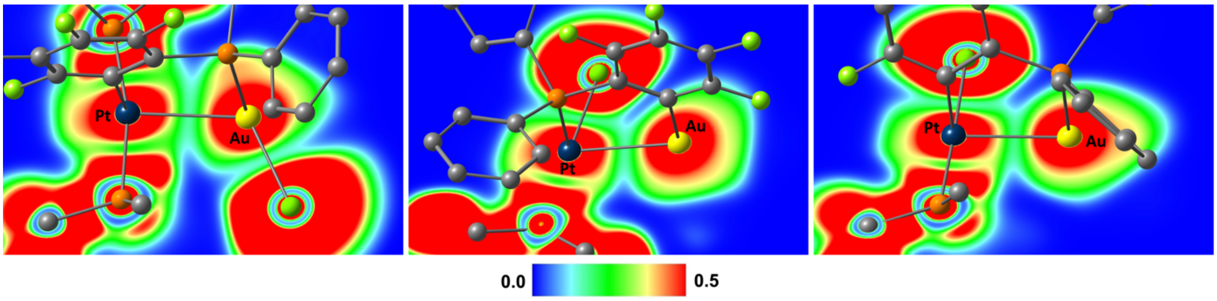

Figure S68. Electron localization function (ELF) of *trans*-**5PtAu** (left), *trans*-**7PtAu** (middle) and *trans*-**8PtAu** (right). Color ranges from ELF = 0.0 to 0.5 a.u.; Color code: blue – strong delocalization, red – electron-gas-like pair probability. Hydrogen atoms are omitted for clarity.

11. Additional NCI

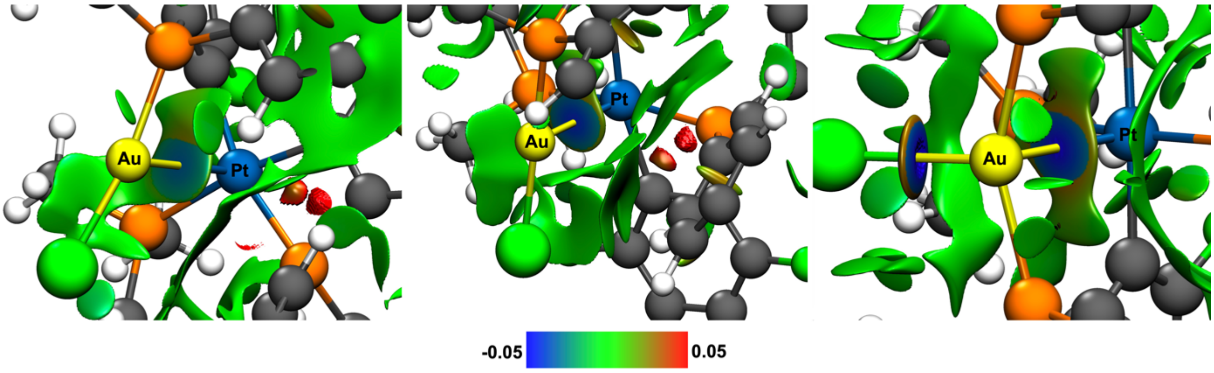

Figure S69. Non-covalent interaction (NCI) descriptor of *cis*-**6PtAu** (left), *trans*-**6PtAu** (middle) and *trans*-**5PtAu** (right). Isovalue is set to 0.45 and colour range from -0.05 to 0.05 a.u.; Colour code: blue – attractive interactions; green – van der Waals interactions; red – non-attractive interactions.

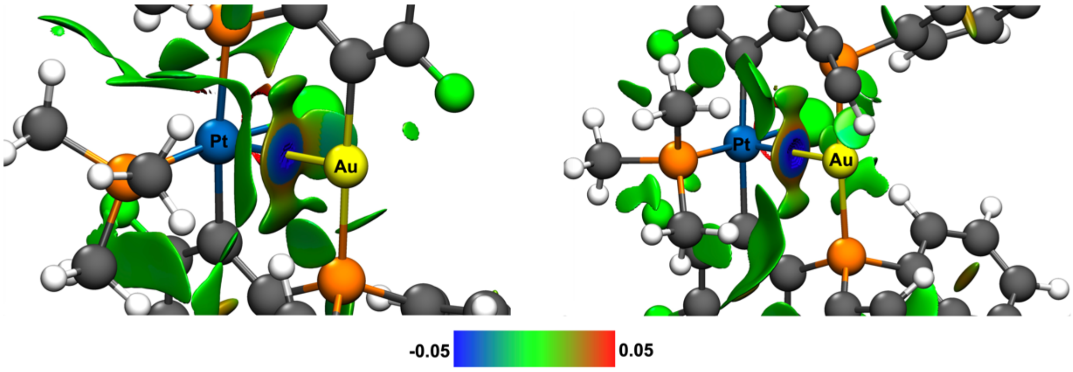

Figure S70. Non-covalent interaction (NCI) descriptor of *trans*-**7PtAu** (left), *trans*-**8PtAu** (right). Isovalue is set to 0.45 and colour range from -0.05 to 0.05 a.u.; Colour code: blue – attractive interactions; green – van der Waals interactions; red – non-attractive interactions.

12. Second Order Perturbation Theory Analysis of Fock Matrix in NBO Basis:

Threshold for printing: 0.50 kcal/mol
(Intermolecular threshold: 0.05 kcal/mol)

Donor (L) NBO	Acceptor (NL) NBO	E(2) kcal/mol	E(NL)-E(L) a.u.	F(L,NL) a.u.
trans-**5NiCu**				
95. LP (2)Ni 1	249. LV (1)Cu 2	1.12	0.35	0.018
96. LP (3)Ni 1	249. LV (1)Cu 2	0.11	0.35	0.006
98. LP (1)Cu 2	250. BD*(1)Ni 1- P 9	0.13	0.38	0.006
98. LP (1)Cu 2	251. BD*(1)Ni 1- C 68	0.09	0.38	0.005
102. LP (5)Cu 2	250. BD*(1)Ni 1- P 9	0.54	0.38	0.013
102. LP (5)Cu 2	251. BD*(1)Ni 1- C 68	0.30	0.37	10.009
trans-**5PdCu**				
90. LP (2)Pd 1	244. LV (1)Cu 2	7.67	0.38	0.048
91. LP (3)Pd 1	244. LV (1)Cu 2	0.52	0.41	0.013
93. LP (1)Cu 2	245. BD*(1)Pd 1- P 5	0.92	0.69	0.022
93. LP (1)Cu 2	246. BD*(1)Pd 1- C 11	0.70	0.58	0.018
94. LP (2)Cu 2	245. BD*(1)Pd 1- P 5	0.14	0.69	0.009
97. LP (5)Cu 2	245. BD*(1)Pd 1- P 5	1.41	0.68	0.028
97. LP (5)Cu 2	246. BD*(1)Pd 1- C 11	0.96	0.58	0.021
trans-**5PtCu**				
90. LP (2)Pt 1	244. LV (1)Cu 2	38.73	0.39	0.109
91. LP (3)Pt 1	244. LV (1)Cu 2	1.03	0.47	0.020
93. LP (1)Cu 2	245. BD*(1)Pt 1- P 10	2.75	0.91	0.045
93. LP (1)Cu 2	246. BD*(1)Pt 1- C 11	1.63	0.72	0.031
97. LP (5)Cu 2	245. BD*(1)Pt 1- P 10	3.43	0.91	0.050
97. LP (5)Cu 2	246. BD*(1)Pt 1- C 11	2.46	0.72	0.038

trans-**5PdAg**

85. LP (2)Pd 1	239. LV (1)Ag 2	7.34	0.63	0.061
86. LP (3)Pd 1	239. LV (1)Ag 2	0.78	0.66	0.020
88. LP (1)Ag 2	240. BD*(1)Pd 1- P 9	0.49	0.72	0.017
88. LP (1)Ag 2	241. BD*(1)Pd 1- C 11	0.21	0.63	0.010
89. LP (2)Ag 2	240. BD*(1)Pd 1- P 9	0.84	0.72	0.022
89. LP (2)Ag 2	241. BD*(1)Pd 1- C 11	0.76	0.62	0.019
92. LP (5)Ag 2	240. BD*(1)Pd 1- P 9	1.32	0.72	0.028
92. LP (5)Ag 2	241. BD*(1)Pd 1- C 11	1.08	0.62	0.023

trans-**5PtAg**

84. LP (1)Pt 1	239. LV (1)Ag 2	26.38	0.60	0.113
86. LP (3)Pt 1	239. LV (1)Ag 2	1.33	0.69	0.027
88. LP (1)Ag 2	240. BD*(1)Pt 1- P 10	0.76	0.96	0.024
88. LP (1)Ag 2	241. BD*(1)Pt 1- C 68	0.56	0.77	0.019
89. LP (2)Ag 2	240. BD*(1)Pt 1- P 10	1.78	0.96	0.037
89. LP (2)Ag 2	241. BD*(1)Pt 1- C 68	0.96	0.77	0.024
92. LP (5)Ag 2	240. BD*(1)Pt 1- P 10	2.31	0.96	0.042
92. LP (5)Ag 2	241. BD*(1)Pt 1- C 68	1.75	0.77	0.033

trans-**5PdAu**

90. LP (2)Pd 2	239. LV (1)Au 1	9.62	0.44	0.058
91. LP (3)Pd 2	239. LV (1)Au 1	1.00	0.47	0.019
84. LP (1)Au 1	240. BD*(1)Pd 2- P 5	1.63	0.72	0.031
84. LP (1)Au 1	241. BD*(1)Pd 2- C 11	0.95	0.63	0.022
85. LP (2)Au 1	240. BD*(1)Pd 2- P 5	0.73	0.73	0.021
85. LP (2)Au 1	241. BD*(1)Pd 2- C 11	0.75	0.63	0.019
88. LP (5)Au 1	240. BD*(1)Pd 2- P 5	3.32	0.72	0.044
88. LP (5)Au 1	241. BD*(1)Pd 2- C 11	2.79	0.63	0.037

trans-**5PtAu**

85. LP (2)Pt 1	239. LV (1)Au 2	30.41	0.41	0.100
86. LP (3)Pt 1	239. LV (1)Au 2	1.85	0.49	0.027
88. LP (1)Au 2	240. BD*(1)Pt 1- P 10	1.76	0.97	0.037
88. LP (1)Au 2	241. BD*(1)Pt 1- C 11	1.27	0.76	0.028
89. LP (2)Au 2	240. BD*(1)Pt 1- P 10	2.64	0.97	0.045
89. LP (2)Au 2	241. BD*(1)Pt 1- C 11	1.42	0.76	0.029
92. LP (5)Au 2	240. BD*(1)Pt 1- P 10	5.57	0.96	0.065
92. LP (5)Au 2	241. BD*(1)Pt 1- C 11	4.32	0.75	0.051

cis-**5PtCu**

89. LP (1)Pt 1	244. LV (1)Cu 2	39.86	0.41	0.114
90. LP (2)Pt 1	244. LV (1)Cu 2	2.06	0.48	0.028
91. LP (3)Pt 1	244. LV (1)Cu 2	1.87	0.47	0.027

92. LP (4)Pt 1	244. LV (1)Cu 2	0.07	0.50	0.005
93. LP (1)Cu 2	246. BD*(1)Pt 1- P 14	2.31	0.78	0.038
93. LP (1)Cu 2	247. BD*(1)Pt 1- P 15	1.95	0.77	0.035
94. LP (2)Cu 2	246. BD*(1)Pt 1- P 14	0.43	0.78	0.016
94. LP (2)Cu 2	247. BD*(1)Pt 1- P 15	0.49	0.77	0.017
95. LP (3)Cu 2	246. BD*(1)Pt 1- P 14	0.05	0.78	0.006
97. LP (5)Cu 2	246. BD*(1)Pt 1- P 14	0.18	0.78	0.011
97. LP (5)Cu 2	247. BD*(1)Pt 1- P 15	0.20	0.77	0.011
cis-**5PtAg**				
84. LP (1)Pt 1	239. LV (1)Ag 2	27.28	0.56	0.110
85. LP (2)Pt 1	239. LV (1)Ag 2	1.02	0.63	0.023
86. LP (3)Pt 1	239. LV (1)Ag 2	1.59	0.63	0.028
87. LP (4)Pt 1	239. LV (1)Ag 2	0.08	0.65	0.007
88. LP (1)Ag 2	240. BD*(1)Pt 1- P 14	0.60	0.82	0.020
88. LP (1)Ag 2	241. BD*(1)Pt 1- P 15	0.40	0.80	0.016
89. LP (2)Ag 2	240. BD*(1)Pt 1- P 14	2.45	0.82	0.040
89. LP (2)Ag 2	241. BD*(1)Pt 1- P 15	2.22	0.80	0.038
91. LP (4)Ag 2	261. BD*(1) P 15- C 48	0.06	0.52	0.005
92. LP (5)Ag 2	240. BD*(1)Pt 1- P 14	0.17	0.82	0.011
92. LP (5)Ag 2	241. BD*(1)Pt 1- P 15	0.16	0.80	0.010
cis-**6PtCu**				
81. LP (1)Pt 1	223. LV (1)Cu 2	26.44	0.28	0.077
82. LP (2)Pt 1	223. LV (1)Cu 2	0.57	0.35	0.013
83. LP (3)Pt 1	223. LV (1)Cu 2	1.44	0.35	0.020
86. LP (2)Cu 2	224. BD*(1)Pt 1- P 6	0.11	0.85	0.009
86. LP (2)Cu 2	225. BD*(1)Pt 1- C 21	0.10	0.70	0.007
87. LP (3)Cu 2	224. BD*(1)Pt 1- P 6	2.84	0.85	0.044
87. LP (3)Cu 2	225. BD*(1)Pt 1- C 21	2.62	0.70	0.038
88. LP (4)Cu 2	224. BD*(1)Pt 1- P 6	0.32	0.85	0.015
88. LP (4)Cu 2	225. BD*(1)Pt 1- C 21	0.21	0.70	0.011
cis-**6PtAu**				
76. LP (1)Pt 1	220. BD*(1)Au 2- P 4	6.62	0.61	0.057
78. LP (3)Pt 1	220. BD*(1)Au 2- P 4	0.42	0.68	0.015
81. LP (2)Au 2	218. BD*(1)Pt 1- P 5	0.18	0.71	0.010
81. LP (2)Au 2	219. BD*(1)Pt 1- P 6	0.19	0.82	0.011
82. LP (3)Au 2	218. BD*(1)Pt 1- P 5	2.94	0.71	0.041
82. LP (3)Au 2	219. BD*(1)Pt 1- P 6	3.02	0.83	0.045
83. LP (4)Au 2	218. BD*(1)Pt 1- P 5	0.74	0.72	0.021
83. LP (4)Au 2	219. BD*(1)Pt 1- P 6	0.81	0.83	20.023

trans-**6PtAu**

76. LP (1)Pt 1	221. BD*(1)Au 2- P 5	13.94	0.60	0.082
77. LP (2)Pt 1	221. BD*(1)Au 2- P 5	1.11	0.66	0.024
78. LP (3)Pt 1	221. BD*(1)Au 2- P 5	0.46	0.67	0.016
79. LP (4)Pt 1	221. BD*(1)Au 2- P 5	0.06	0.68	0.006
81. LP (2)Au 2	219. BD*(1)Pt 1- P 6	0.62	0.87	0.021
81. LP (2)Au 2	220. BD*(1)Pt 1- C 15	0.41	0.67	0.015
82. LP (3)Au 2	219. BD*(1)Pt 1- P 6	6.53	0.88	0.068
82. LP (3)Au 2	220. BD*(1)Pt 1- C 15	4.67	0.68	0.050
84. LP (5)Au 2	219. BD*(1)Pt 1- P 6	0.12	0.88	0.009
84. LP (5)Au 2	220. BD*(1)Pt 1- C 15	0.22	0.68	0.011

cis-trans-**7NiAu**

81. LP (1)Ni 1	225. BD*(1)Au 2- C 21	1.41	0.48	0.023
82. LP (2)Ni 1	225. BD*(1)Au 2- C 21	0.44	0.48	0.013
83. LP (3)Ni 1	225. BD*(1)Au 2- C 21	0.19	0.48	0.009
85. LP (1)Au 2	224. BD*(1)Ni 1- C 15	0.12	0.40	0.006
86. LP (2)Au 2	223. BD*(1)Ni 1- P 6	0.26	0.42	0.009
86. LP (2)Au 2	224. BD*(1)Ni 1- C 15	0.12	0.40	0.006
87. LP (3)Au 2	223. BD*(1)Ni 1- P 6	0.09	0.43	0.005
88. LP (4)Au 2	223. BD*(1)Ni 1- P 6	4.82	0.41	0.040
88. LP (4)Au 2	224. BD*(1)Ni 1- C 15	2.48	0.39	0.028
89. LP (5)Au 2	223. BD*(1)Ni 1- P 6	0.31	0.43	0.010
89. LP (5)Au 2	224. BD*(1)Ni 1- C 15	0.11	0.41	0.006

trans-**7PtAu**

76. LP (1)Pt 1	220. BD*(1)Au 2- C 21	14.10	0.46	0.072
81. LP (2)Au 2	219. BD*(1)Pt 1- P 6	0.56	0.97	0.021
82. LP (3)Au 2	218. BD*(1)Pt 1- P 5	6.83	0.80	0.066
82. LP (3)Au 2	219. BD*(1)Pt 1- P 6	7.89	0.95	0.077

trans-**8NiCu**

91. LP (2)Ni 1	244. LV (1)Cu 2	1.71	0.39	0.023
92. LP (3)Ni 1	244. LV (1)Cu 2	0.33	0.39	0.010
94. LP (1)Cu 2	246. BD*(1)Ni 1- P 6	0.14	0.42	0.007
95. LP (2)Cu 2	247. BD*(1)Ni 1- C 21	0.05	0.40	0.004
98. LP (5)Cu 2	246. BD*(1)Ni 1- P 6	0.57	0.42	0.014
98. LP (5)Cu 2	247. BD*(1)Ni 1- C 21	0.24	0.41	0.009

trans-**8PtAu**

77. LP (2)Pt 1	220. BD*(1)Au 2- P 5	19.20	0.43	0.081
78. LP (3)Pt 1	220. BD*(1)Au 2- P 5	9.42	0.45	0.058
81. LP (2)Au 2	218. BD*(1)Pt 1- P 6	0.79	1.08	0.026
82. LP (3)Au 2	218. BD*(1)Pt 1- P 6	5.42	1.08	0.068
82. LP (3)Au 2	219. BD*(1)Pt 1- C 21	4.27	0.80	0.052

==

13. Graphical representations of optimized molecular structures, total energies and atomic coordinates:

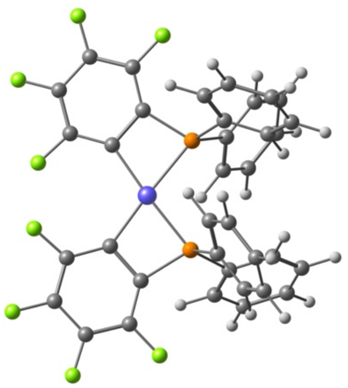

Figure S71. Optimized molecular structure of *cis*-**1Ni**.

PBE0:

final single point energy: -4395.750590013117 a.u.
final Gibbs free energy: -4395.35286665 a.u.

Table S4. Atomic coordinates for optimized structure of *cis*-**1Ni**.

Ni	6.232507000	6.356746000	5.956552000
P	5.692021000	5.009831000	7.576108000
F	6.446424000	9.749277000	7.250678000
F	5.496240000	10.382775000	9.637821000
F	4.456479000	8.548982000	11.288880000
F	4.389192000	5.929424000	10.544799000
C	5.325491000	6.521793000	8.443318000
C	5.837509000	7.458599000	7.518337000
C	5.905926000	8.762551000	7.972837000
C	5.434149000	9.118808000	9.228481000
C	4.896688000	8.172224000	10.092162000
C	4.857685000	6.849353000	9.699649000
C	4.257021000	3.928880000	7.434573000
C	2.972821000	4.452767000	7.556037000
C	1.870155000	3.648037000	7.312196000
C	2.043798000	2.321763000	6.941556000
C	3.323774000	1.797673000	6.817261000
C	4.428527000	2.597186000	7.059668000
C	6.959059000	4.077749000	8.461214000
C	8.286321000	4.246313000	8.075738000

C	9.295371000	3.570180000	8.744581000
C	8.980548000	2.724291000	9.798137000
C	7.656867000	2.555502000	10.187024000
C	6.645663000	3.228921000	9.521663000
H	2.834957000	5.490506000	7.836738000
H	0.872917000	4.058920000	7.413022000
H	1.181001000	1.695394000	6.749750000
H	3.462332000	0.763241000	6.527019000
H	5.425624000	2.185441000	6.956157000
H	8.524924000	4.902511000	7.247008000
H	10.325622000	3.699028000	8.435988000
H	9.767791000	2.191895000	10.318455000
H	7.412632000	1.896398000	11.011332000
H	5.613965000	3.093447000	9.823735000
P	6.773043000	5.009823000	4.337025000
F	6.018467000	9.749249000	4.662344000
F	6.968764000	10.382745000	2.275247000
F	8.008676000	8.548962000	0.624270000
F	8.076001000	5.929415000	1.368389000
C	7.139585000	6.521785000	3.469818000
C	6.627493000	7.458592000	4.394761000
C	6.559048000	8.762533000	3.940235000
C	7.030879000	9.118786000	2.684610000
C	7.568419000	8.172208000	1.820971000
C	7.607441000	6.849343000	2.213504000
C	8.208037000	3.928868000	4.478591000
C	9.492243000	4.452764000	4.357233000
C	10.594895000	3.648026000	4.601109000
C	10.421232000	2.321735000	4.971677000
C	9.141250000	1.797634000	5.095865000
C	8.036511000	2.597156000	4.853424000
C	5.506022000	4.077745000	3.451891000
C	4.178754000	4.246298000	3.837351000
C	3.169715000	3.570183000	3.168472000
C	3.484556000	2.724323000	2.114898000
C	4.808243000	2.555543000	1.726030000
C	5.819436000	3.228945000	2.391425000
H	9.630122000	5.490516000	4.076586000
H	11.592138000	4.058916000	4.500365000
H	11.284019000	1.695359000	5.163510000
H	9.002676000	0.763189000	5.386050000
H	7.039408000	2.185403000	4.956849000
H	3.940137000	4.902474000	4.666093000
H	2.139458000	3.699024000	3.477051000
H	2.697322000	2.191943000	1.594550000
H	5.052493000	1.896461000	0.901709000
H	6.851139000	3.093479000	2.089368000

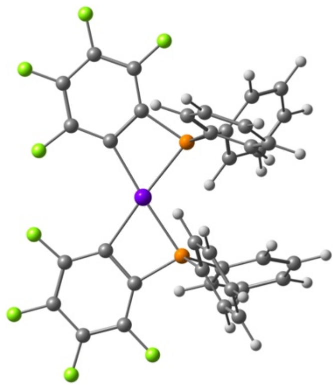

Figure S72. Optimized molecular structure of *cis*-**1Pd**.

PBE0:

final single point energy: -7972.010860689866 a.u.

final Gibbs free energy: -7971.61266264 a.u.

Table S5. Atomic coordinates for optimized structure of *cis*-**1Pd**.

Pd	6.232533000	6.369878000	5.956564000
P	5.661961000	5.015790000	7.694581000
F	5.917305000	9.854769000	7.273348000
F	5.026716000	10.408692000	9.712459000
F	4.317275000	8.482150000	11.435231000
F	4.508347000	5.864906000	10.704327000
C	5.312932000	6.532357000	8.574454000
C	5.685891000	7.513987000	7.633180000
C	5.581489000	8.822813000	8.055030000
C	5.121798000	9.139888000	9.326408000
C	4.753129000	8.146715000	10.224690000
C	4.852835000	6.823017000	9.842831000
C	4.198831000	3.971431000	7.575877000
C	2.927013000	4.524613000	7.698456000
C	1.806518000	3.741181000	7.467482000
C	1.949652000	2.407828000	7.108952000
C	3.217390000	1.855254000	6.983367000
C	4.340510000	2.633080000	7.212273000
C	6.927730000	4.047323000	8.537487000
C	8.234126000	4.107094000	8.060504000
C	9.237793000	3.390010000	8.694885000
C	8.937657000	2.613344000	9.804159000
C	7.633911000	2.552262000	10.283055000
C	6.628078000	3.265034000	9.652409000
H	2.812372000	5.567847000	7.968650000
H	0.818868000	4.174543000	7.568478000
H	1.072612000	1.798486000	6.927169000
H	3.332473000	0.815599000	6.701695000
H	5.328286000	2.200072000	7.104235000
H	8.459191000	4.710394000	7.188791000
H	10.251649000	3.434107000	8.316167000
H	9.720397000	2.050302000	10.298343000
H	7.401320000	1.945799000	11.150011000
H	5.611521000	3.212291000	10.023638000

P	6.803103000	5.015790000	4.218547000
F	6.547767000	9.854769000	4.639785000
F	7.438351000	10.408693000	2.200673000
F	8.147782000	8.482152000	0.477895000
F	7.956708000	5.864908000	1.208798000
C	7.152130000	6.532357000	3.338673000
C	6.779173000	7.513987000	4.279949000
C	6.883578000	8.822813000	3.858101000
C	7.343267000	9.139889000	2.586722000
C	7.711931000	8.146716000	1.688438000
C	7.612223000	6.823018000	2.070295000
C	8.266234000	3.971432000	4.337246000
C	9.538052000	4.524613000	4.214658000
C	10.658547000	3.741180000	4.445628000
C	10.515414000	2.407828000	4.804161000
C	9.247677000	1.855255000	4.929754000
C	8.124556000	2.633082000	4.700853000
C	5.537332000	4.047323000	3.375644000
C	4.230936000	4.107097000	3.852627000
C	3.227269000	3.390012000	3.218249000
C	3.527404000	2.613342000	2.108978000
C	4.831149000	2.552257000	1.630081000
C	5.836983000	3.265030000	2.260725000
H	9.652692000	5.567846000	3.944462000
H	11.646197000	4.174541000	4.344625000
H	11.392455000	1.798486000	4.985940000
H	9.132594000	0.815601000	5.211429000
H	7.136781000	2.200075000	4.808898000
H	4.005873000	4.710400000	4.724338000
H	2.213413000	3.434111000	3.596967000
H	2.744663000	2.050299000	1.614796000
H	5.063740000	1.945790000	0.763127000
H	6.853540000	3.212284000	1.889496000

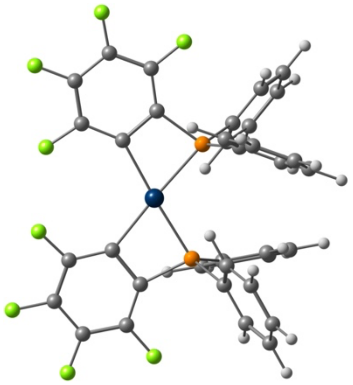

Figure S73. Optimized molecular structure of *cis*-**1Pt**.

PBE0:

final single point energy: -21907.047766384465 a.u.

final Gibbs free energy: -21906.64980847 a.u.

Table S6. Atomic coordinates for optimized structure of *cis*-**1Pt**.

Pt	6.231807000	6.357428000	5.956262000
P	5.660568000	5.023927000	7.698707000
F	5.928685000	9.841173000	7.248652000
F	5.052752000	10.424066000	9.678040000
F	4.342533000	8.523922000	11.430020000
F	4.519234000	5.896174000	10.730394000
C	5.307512000	6.535547000	8.588075000
C	5.683840000	7.502779000	7.627248000
C	5.589679000	8.818340000	8.037475000
C	5.137889000	9.150032000	9.308691000
C	4.769148000	8.171428000	10.221421000
C	4.860448000	6.842154000	9.854751000
C	4.204468000	3.969708000	7.573476000
C	2.929892000	4.520395000	7.676647000
C	1.814379000	3.731786000	7.440159000
C	1.965851000	2.395962000	7.093896000
C	3.236264000	1.846114000	6.987069000
C	4.354552000	2.628983000	7.223855000
C	6.925625000	4.049588000	8.535075000
C	8.229578000	4.099611000	8.050931000
C	9.231252000	3.375581000	8.680535000
C	8.932035000	2.604600000	9.794021000
C	7.630947000	2.555238000	10.281533000
C	6.626707000	3.272970000	9.654095000
H	2.809592000	5.565642000	7.936535000
H	0.824281000	4.162662000	7.526581000
H	1.092697000	1.782454000	6.907369000
H	3.357442000	0.804561000	6.715210000
H	5.344681000	2.197740000	7.132557000
H	8.452314000	4.702067000	7.178117000
H	10.243166000	3.411056000	8.295772000
H	9.713582000	2.037316000	10.285268000
H	7.399277000	1.953507000	11.151999000
H	5.611841000	3.228133000	10.031168000

P	6.804099000	5.023970000	4.214123000
F	6.535353000	9.841190000	4.664073000
F	7.412586000	10.424164000	2.235176000
F	8.123857000	8.524089000	0.483552000
F	7.946959000	5.896319000	1.183062000
C	7.157583000	6.535626000	3.324993000
C	6.780708000	7.502823000	4.285644000
C	6.874987000	8.818393000	3.875474000
C	7.327398000	9.150124000	2.604488000
C	7.696652000	8.171554000	1.691930000
C	7.605231000	6.842271000	2.058533000
C	8.260143000	3.969780000	4.340206000
C	9.534768000	4.520458000	4.237652000
C	10.650141000	3.732036000	4.475431000
C	10.498470000	2.396402000	4.822317000
C	9.228009000	1.846520000	4.928376000
C	8.109868000	2.629212000	4.690356000
C	5.539559000	4.049646000	3.376948000
C	4.235319000	4.099621000	3.860322000
C	3.233962000	3.375913000	3.229848000
C	3.533807000	2.605171000	2.116362000
C	4.835208000	2.555754000	1.629703000
C	5.839114000	3.273245000	2.257951000
H	9.655210000	5.565618000	3.977486000
H	11.640280000	4.162950000	4.389657000
H	11.371501000	1.783098000	5.010054000
H	9.106672000	0.805136000	5.200807000
H	7.119689000	2.198015000	4.781327000
H	4.012098000	4.701833000	4.733208000
H	2.221788000	3.411487000	3.613899000
H	2.752511000	2.038109000	1.624460000
H	5.067372000	1.954217000	0.759235000
H	6.854205000	3.228443000	1.881482000

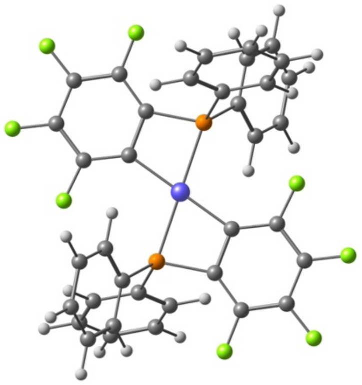

Figure S74. Optimized molecular structure of *trans*-**1Ni**.

PBE0:

final single point energy: -4395.755315358042 a.u.

final Gibbs free energy: -4395.35754721 a.u.

Table S7. Atomic coordinates for optimized structure of *trans*-**1Ni**.

Ni	3.358334000	6.102305000	6.164457000
P	3.693069000	7.040648000	8.115824000
F	0.006967000	6.380572000	5.107345000
F	-1.852562000	7.626261000	6.579014000
F	-1.265298000	8.726723000	8.946591000
F	1.262557000	8.596177000	9.934601000
C	1.946934000	7.408031000	7.991519000
C	1.676837000	6.820764000	6.741102000
C	0.378230000	6.907899000	6.285340000
C	-0.602843000	7.546209000	7.026631000
C	-0.302564000	8.119115000	8.258526000
C	0.987963000	8.049277000	8.749672000
C	4.004607000	6.096092000	9.623596000
C	2.980608000	5.342841000	10.193517000
C	3.233376000	4.558263000	11.307935000
C	4.508446000	4.515462000	11.855040000
C	5.532339000	5.260004000	11.285086000
C	5.284901000	6.047481000	10.171759000
C	4.665522000	8.553876000	8.253683000
C	5.368387000	8.989325000	7.134293000
C	6.097727000	10.168281000	7.188351000
C	6.124889000	10.911733000	8.359085000
C	5.423922000	10.478829000	9.478764000
C	4.695947000	9.301889000	9.429984000
H	1.984261000	5.372601000	9.768019000
H	2.431018000	3.980766000	11.750980000
H	4.704308000	3.902448000	12.726542000
H	6.528672000	5.229515000	11.709335000
H	6.087441000	6.626889000	9.732469000
H	5.343673000	8.398623000	6.225415000
H	6.646810000	10.503482000	6.316879000
H	6.695443000	11.831790000	8.402479000
H	5.446117000	11.060715000	10.392243000
H	4.152877000	8.963203000	10.304435000

P	3.023673000	5.164233000	4.212946000
F	6.709503000	5.823261000	7.221989000
F	8.569015000	4.577489000	5.750367000
F	7.981865000	3.477453000	3.382565000
F	5.454146000	3.608525000	2.394273000
C	4.769735000	4.796557000	4.337413000
C	5.039776000	5.383616000	5.587938000
C	6.338308000	5.296191000	6.043856000
C	7.319368000	4.657821000	5.302599000
C	7.019148000	4.085134000	4.070589000
C	5.728691000	4.155245000	3.579296000
C	2.712461000	6.108977000	2.705223000
C	3.736629000	6.862165000	2.135524000
C	3.484116000	7.646878000	1.021143000
C	2.209133000	7.689873000	0.473850000
C	1.185073000	6.945387000	1.043577000
C	1.432256000	6.157778000	2.156867000
C	2.050993000	3.651174000	4.074840000
C	1.347858000	3.215792000	5.194086000
C	0.618326000	2.036963000	5.139844000
C	0.591238000	1.293574000	3.969068000
C	1.292469000	1.726416000	2.849531000
C	2.020638000	2.903228000	2.898494000
H	4.732911000	6.832250000	2.561165000
H	4.286605000	8.224327000	0.578273000
H	2.013470000	8.302992000	-0.397623000
H	0.188809000	6.976025000	0.619180000
H	0.629588000	5.578412000	2.595976000
H	1.372513000	3.806448000	6.102997000
H	0.069033000	1.701814000	6.011204000
H	0.020532000	0.373618000	3.925530000
H	1.270328000	1.144581000	1.936017000
H	2.563913000	3.241866000	2.024152000

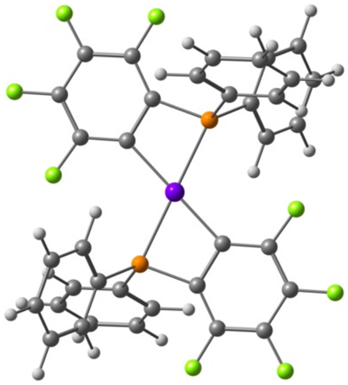

Figure S75. Optimized molecular structure of *trans*-**1Pd**.

PBE0:

final single point energy: -7972.012283235156 a.u.
final Gibbs free energy: -7971.61549209 a.u.

Table S8. Atomic coordinates for optimized structure of *trans*-**1Pd**.

Pd	3.358329000	6.102261000	6.164478000
P	3.651194000	7.100343000	8.222171000
F	-0.109823000	6.470086000	5.242826000
F	-1.932232000	7.728189000	6.758971000
F	-1.283922000	8.810196000	9.120184000
F	1.259366000	8.648685000	10.054627000
C	1.897695000	7.464148000	8.094401000
C	1.588212000	6.889227000	6.848226000
C	0.285094000	6.988206000	6.416562000
C	-0.675015000	7.632609000	7.181081000
C	-0.342838000	8.195805000	8.408329000
C	0.957222000	8.110402000	8.872621000
C	3.967381000	6.156610000	9.726650000
C	2.936381000	5.464956000	10.357104000
C	3.205670000	4.684682000	11.471091000
C	4.502447000	4.585591000	11.955672000
C	5.533287000	5.268446000	11.323488000
C	5.270265000	6.050799000	10.210787000
C	4.622209000	8.613553000	8.359230000
C	5.337419000	9.044870000	7.246213000
C	6.074174000	10.218885000	7.308246000
C	6.097136000	10.960038000	8.480381000
C	5.385114000	10.529790000	9.594231000
C	4.649514000	9.358120000	9.538022000
H	1.923313000	5.537189000	9.979354000
H	2.398780000	4.154655000	11.962518000
H	4.710612000	3.975950000	12.826657000
H	6.546838000	5.192474000	11.698281000
H	6.077997000	6.581074000	9.720472000
H	5.316751000	8.454173000	6.337081000
H	6.632958000	10.551207000	6.441889000
H	6.673952000	11.875856000	8.529964000
H	5.405242000	11.109366000	10.509189000
H	4.099001000	9.020623000	10.408224000

P	3.065544000	5.104492000	4.106621000
F	6.826313000	5.733745000	7.086495000
F	8.648707000	4.475598000	5.570372000
F	8.000502000	3.394015000	3.208937000
F	5.457339000	3.556016000	2.274239000
C	4.818971000	4.740396000	4.234546000
C	5.128399000	5.315098000	5.480836000
C	6.431453000	5.215863000	5.912634000
C	7.391553000	4.571426000	5.148133000
C	7.059430000	4.008449000	3.920770000
C	5.759434000	4.094106000	3.456345000
C	2.749679000	6.048457000	2.602219000
C	3.780861000	6.740023000	1.971967000
C	3.511824000	7.520476000	0.858044000
C	2.215119000	7.619834000	0.373326000
C	1.184097000	6.937067000	1.005308000
C	1.446867000	6.154535000	2.117944000
C	2.094305000	3.591453000	3.969264000
C	1.378827000	3.160162000	5.082120000
C	0.641883000	1.986278000	5.019855000
C	0.618998000	1.245232000	3.847651000
C	1.331285000	1.675456000	2.733962000
C	2.067075000	2.846995000	2.790401000
H	4.793874000	6.667582000	2.349823000
H	4.318856000	8.050433000	0.366773000
H	2.007152000	8.229614000	-0.497609000
H	0.170602000	7.013247000	0.630408000
H	0.638993000	5.624330000	2.608101000
H	1.399437000	3.750776000	5.991307000
H	0.082891000	1.653975000	5.886086000
H	0.042033000	0.329517000	3.797887000
H	1.311216000	1.095965000	1.818949000
H	2.617796000	3.184476000	1.920324000

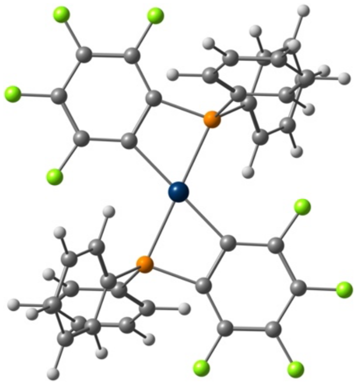

Figure S76. Optimized molecular structure of *trans*-**1Pt**.

PBE0:

final single point energy: -21907.048741495699 a.u.

final Gibbs free energy: -21906.65159268 a.u.

Table S9. Atomic coordinates for optimized structure of *trans*-**1Pt**.

Pt	3.358317000	6.102036000	6.164527000
P	3.632304000	7.112715000	8.204847000
F	-0.094324000	6.454266000	5.225181000
F	-1.936674000	7.709388000	6.715935000
F	-1.319823000	8.813068000	9.076441000
F	1.217782000	8.674833000	10.035410000
C	1.878393000	7.481520000	8.090182000
C	1.588237000	6.892750000	6.841572000
C	0.286145000	6.982411000	6.398134000
C	-0.683387000	7.626244000	7.151249000
C	-0.367748000	8.200197000	8.377813000
C	0.929075000	8.126420000	8.854950000
C	3.964232000	6.157437000	9.697652000
C	2.939622000	5.445973000	10.316452000
C	3.215699000	4.654154000	11.420364000
C	4.513161000	4.563010000	11.904990000
C	5.537407000	5.265781000	11.284303000
C	5.267253000	6.061216000	10.182472000
C	4.618534000	8.614754000	8.336758000
C	5.331431000	9.043914000	7.221689000
C	6.078740000	10.211024000	7.285474000
C	6.112013000	10.948253000	8.459912000
C	5.400309000	10.520834000	9.575043000
C	4.655828000	9.354922000	9.517965000
H	1.926705000	5.512548000	9.937176000
H	2.414359000	4.108386000	11.903492000
H	4.726888000	3.943893000	12.767929000
H	6.551030000	5.195827000	11.659980000
H	6.069158000	6.608225000	9.701435000
H	5.298748000	8.457368000	6.310325000
H	6.636645000	10.541987000	6.418048000
H	6.696237000	11.859332000	8.510070000
H	5.428158000	11.097639000	10.491505000
H	4.106166000	9.018744000	10.389338000

P	3.084358000	5.091745000	4.124031000
F	6.810810000	5.748717000	7.104276000
F	8.652780000	4.492572000	5.613959000
F	8.035848000	3.389146000	3.253336000
F	5.498503000	3.528723000	2.293839000
C	4.838133000	4.722389000	4.238921000
C	5.128306000	5.310972000	5.487619000
C	6.430286000	5.220726000	5.931269000
C	7.399628000	4.576339000	5.178379000
C	7.083946000	4.002508000	3.951768000
C	5.787262000	4.076966000	3.474364000
C	2.753171000	6.048010000	2.631684000
C	3.778309000	6.759121000	2.013352000
C	3.502849000	7.551759000	0.909873000
C	2.205484000	7.644062000	0.425207000
C	1.180719000	6.941634000	1.045423000
C	1.450251000	6.145390000	2.146823000
C	2.097639000	3.590135000	3.991116000
C	1.384893000	3.160289000	5.106020000
C	0.637256000	1.993434000	5.041490000
C	0.603487000	1.257155000	3.866468000
C	1.315025000	1.685273000	2.751499000
C	2.059848000	2.850930000	2.809325000
H	4.791150000	6.691632000	2.392666000
H	4.304593000	8.097258000	0.427112000
H	1.992238000	8.263816000	-0.437394000
H	0.167170000	7.012493000	0.669714000
H	0.647935000	5.598669000	2.627496000
H	1.417930000	3.746115000	6.017837000
H	0.079461000	1.661940000	5.908784000
H	0.018988000	0.346286000	3.815724000
H	1.286775000	1.109217000	1.834579000
H	2.609385000	3.187660000	1.938089000

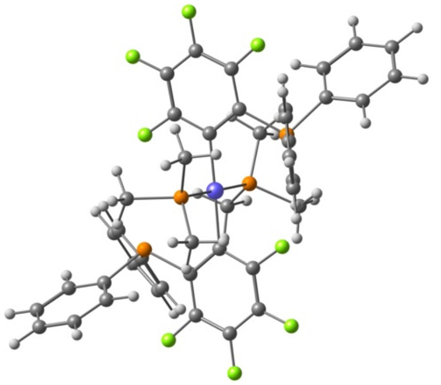

Figure S77. Optimized molecular structure of *anti*-**2Ni**[a].

PBE0:

final single point energy: -5320.381964444721 a.u.
final Gibbs free energy: -5319.76630310 a.u.

Table S10. Atomic coordinates for optimized structure of *anti*-**2Ni**[a].

Ni	8.343507000	2.293105000	3.009552000
P	6.086833000	4.253034000	1.488641000
P	10.403106000	0.417698000	4.658560000
P	9.665587000	2.685192000	1.282442000
P	7.068051000	1.885116000	4.743646000
F	6.283676000	7.220685000	2.420366000
F	8.027516000	8.284234000	4.127641000
F	9.876229000	6.714740000	5.333720000
F	9.980006000	4.110221000	4.841015000
F	10.670298000	-2.478120000	3.374103000
F	9.122172000	-3.531022000	1.485318000
F	7.168292000	-2.051308000	0.339884000
F	6.786421000	0.490783000	1.051885000
C	8.144411000	4.194690000	3.340477000
C	7.184772000	5.032061000	2.723284000
C	7.166342000	6.396491000	2.997808000
C	8.061190000	6.976620000	3.873903000
C	9.001669000	6.172196000	4.490192000
C	9.018204000	4.818760000	4.213245000
C	8.540204000	0.414688000	2.646930000
C	9.523706000	-0.386267000	3.270393000
C	9.717489000	-1.696519000	2.851144000
C	8.933928000	-2.266980000	1.865718000
C	7.938810000	-1.507531000	1.278884000
C	7.769118000	-0.193629000	1.675739000
C	5.390429000	2.567900000	4.660870000
H	4.843204000	2.300107000	5.566448000
H	4.867027000	2.173940000	3.790636000
H	5.438725000	3.651251000	4.575289000
C	7.669104000	2.555578000	6.322115000
H	7.715703000	3.642353000	6.266593000
H	8.665254000	2.165048000	6.524370000
H	6.985524000	2.264207000	7.121584000
C	6.722230000	0.155696000	5.175624000

H	6.022234000	0.122536000	6.012094000
H	7.643178000	-0.345816000	5.464332000
H	6.296371000	-0.362830000	4.317108000
C	10.649110000	4.213003000	1.338631000
H	11.319499000	4.174991000	2.197678000
H	9.994304000	5.075626000	1.449805000
H	11.237608000	4.312128000	0.425139000
C	8.794334000	2.799777000	-0.309001000
H	8.073317000	3.612680000	-0.303833000
H	8.261111000	1.867367000	-0.490581000
H	9.523430000	2.964865000	-1.104635000
C	10.931533000	1.452008000	0.851855000
H	11.461138000	1.792065000	-0.039769000
H	10.456460000	0.493316000	0.647161000
H	11.639692000	1.324504000	1.666663000
C	4.369078000	4.416565000	2.094966000
C	3.917696000	5.334911000	3.039922000
H	4.598206000	6.053107000	3.475138000
C	2.591787000	5.331367000	3.445848000
H	2.257650000	6.047127000	4.187831000
C	1.694397000	4.419308000	2.906286000
H	0.658904000	4.421432000	3.225139000
C	2.132778000	3.499531000	1.965137000
H	1.443068000	2.777015000	1.545198000
C	3.463858000	3.491295000	1.573951000
H	3.810716000	2.749265000	0.862332000
C	6.101977000	5.451696000	0.099805000
C	7.286424000	6.091457000	-0.268441000
H	8.176181000	5.975032000	0.338738000
C	7.339351000	6.897343000	-1.394951000
H	8.267755000	7.392694000	-1.654557000
C	6.210359000	7.073749000	-2.182737000
H	6.250306000	7.704570000	-3.062533000
C	5.028749000	6.437900000	-1.829152000
H	4.138172000	6.571398000	-2.432309000
C	4.974603000	5.633326000	-0.700451000
H	4.040434000	5.151842000	-0.438065000
C	10.693201000	-0.902943000	5.887634000
C	11.786980000	-0.790181000	6.747974000
H	12.516235000	-0.003337000	6.592418000
C	11.959085000	-1.678603000	7.798455000
H	12.820002000	-1.577314000	8.449018000
C	11.037115000	-2.693182000	8.015598000
H	11.172131000	-3.388833000	8.835022000
C	9.942937000	-2.811266000	7.169990000
H	9.219942000	-3.604017000	7.323121000
C	9.771116000	-1.923797000	6.118716000
H	8.919545000	-2.049075000	5.460780000
C	12.098123000	0.742549000	4.051471000
C	12.534562000	2.064850000	4.085302000
H	11.878734000	2.829816000	4.481315000

C	13.789593000	2.409357000	3.599380000
H	14.113412000	3.443083000	3.626934000
C	14.620486000	1.431222000	3.076432000
H	15.597618000	1.696506000	2.690476000
C	14.199477000	0.106016000	3.052565000
H	14.849664000	-0.662021000	2.650305000
C	12.949709000	-0.237989000	3.540339000
H	12.633174000	-1.272069000	3.519160000

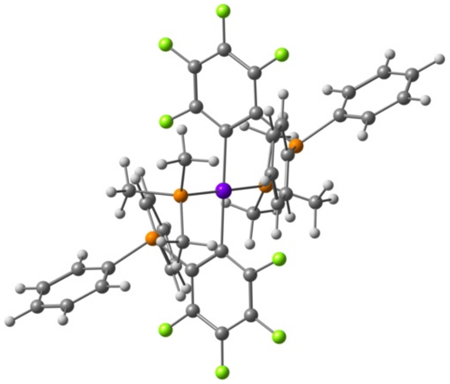

Figure S78. Optimized molecular structure of *anti*-**2Pd**[a].

PBE0:

final single point energy: -8896.643975610943 a.u.

final Gibbs free energy: -8896.03101531 a.u.

Table S11. Atomic coordinates for optimized structure of *anti*-**2Pd**[a].

Pd	8.346859000	2.317980000	3.003449000
P	6.190252000	4.356495000	1.414079000
P	10.416839000	0.373977000	4.691954000
P	9.746003000	2.726148000	1.193400000
P	6.949018000	1.900295000	4.800087000
F	6.248454000	7.340589000	2.414288000
F	7.893519000	8.406846000	4.211781000
F	9.720979000	6.862591000	5.475754000
F	9.899602000	4.257774000	4.952175000
F	10.628831000	-2.591660000	3.578370000
F	9.132284000	-3.693097000	1.674671000
F	7.271067000	-2.225320000	0.375531000
F	6.907942000	0.354764000	0.971765000
C	8.140624000	4.349719000	3.361693000
C	7.196770000	5.168615000	2.705920000
C	7.129301000	6.524586000	3.006794000
C	7.970322000	7.105126000	3.936213000
C	8.900024000	6.312843000	4.583986000
C	8.959899000	4.963664000	4.287742000
C	8.580064000	0.298362000	2.651803000
C	9.531373000	-0.483727000	3.338766000
C	9.711773000	-1.815775000	2.986889000
C	8.955315000	-2.410884000	1.994682000
C	8.006358000	-1.657063000	1.328837000
C	7.844507000	-0.325602000	1.666431000

C	5.452797000	2.915393000	4.931853000
H	4.928147000	2.667232000	5.856410000
H	4.799445000	2.725873000	4.082308000
H	5.720580000	3.970923000	4.934620000
C	7.714662000	2.159794000	6.424915000
H	8.046720000	3.194770000	6.503430000
H	8.580654000	1.508657000	6.530218000
H	6.985578000	1.949759000	7.209775000
C	6.268832000	0.221725000	4.904831000
H	5.650663000	0.120075000	5.798554000
H	7.077457000	-0.505778000	4.930442000
H	5.664168000	0.031174000	4.017526000
C	10.727536000	4.249414000	1.294489000
H	11.379636000	4.195037000	2.166638000
H	10.064026000	5.105161000	1.411479000
H	11.332823000	4.370433000	0.394386000
C	8.888998000	2.867459000	-0.401098000
H	8.185224000	3.696119000	-0.387821000
H	8.334203000	1.948200000	-0.587974000
H	9.624151000	3.020416000	-1.193704000
C	10.998789000	1.475505000	0.791006000
H	11.529225000	1.780067000	-0.113037000
H	10.513939000	0.514340000	0.623036000
H	11.706542000	1.370141000	1.609468000
C	4.466102000	4.332921000	2.016317000
C	3.885377000	5.280976000	2.856642000
H	4.455191000	6.136366000	3.191598000
C	2.574555000	5.131894000	3.281354000
H	2.137569000	5.871445000	3.942099000
C	1.821351000	4.041457000	2.864037000
H	0.797197000	3.928772000	3.199371000
C	2.386531000	3.096006000	2.021497000
H	1.807754000	2.240299000	1.694709000
C	3.704326000	3.237959000	1.608942000
H	4.155855000	2.483934000	0.973026000
C	6.119671000	5.608567000	0.082067000
C	7.291487000	6.252020000	-0.321015000
H	8.210171000	6.099788000	0.234304000
C	7.293205000	7.102978000	-1.414395000
H	8.210856000	7.602064000	-1.703459000
C	6.125665000	7.317701000	-2.135244000
H	6.126300000	7.982786000	-2.990397000
C	4.958064000	6.676011000	-1.748189000
H	4.039802000	6.839427000	-2.300374000
C	4.953928000	5.827969000	-0.649658000
H	4.031817000	5.340596000	-0.356182000
C	10.730224000	-0.917101000	5.943919000
C	11.950155000	-0.999189000	6.613265000
H	12.779616000	-0.374832000	6.303544000
C	12.118700000	-1.881713000	7.671563000
H	13.076999000	-1.935469000	8.175090000

C	11.072032000	-2.695085000	8.079708000
H	11.205915000	-3.387198000	8.902339000
C	9.849630000	-2.614803000	7.424298000
H	9.024421000	-3.245997000	7.733043000
C	9.678465000	-1.728795000	6.373689000
H	8.718256000	-1.681632000	5.872825000
C	12.081921000	0.705256000	4.019785000
C	12.527394000	2.023576000	4.084429000
H	11.895990000	2.775044000	4.544002000
C	13.756543000	2.382504000	3.545781000
H	14.088030000	3.412858000	3.596552000
C	14.550229000	1.423070000	2.936714000
H	15.505936000	1.700370000	2.507960000
C	14.118929000	0.102267000	2.877609000
H	14.739589000	-0.649903000	2.404987000
C	12.894992000	-0.256111000	3.417951000
H	12.567830000	-1.286246000	3.365254000

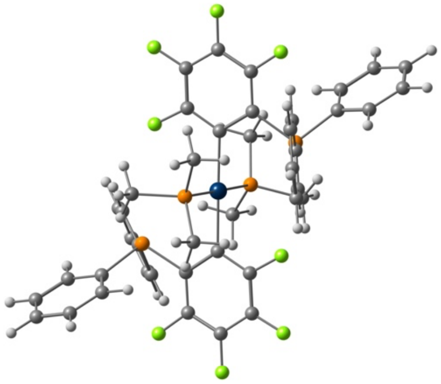

Figure S79. Optimized molecular structure of *anti*-**2Pt**[a].

PBE0:

final single point energy: -22831.682143520688 a.u.

final Gibbs free energy: -22831.06692962 a.u.

Table S12. Atomic coordinates for optimized structure of *anti*-**2Pt**[a].

Pt	8.345426000	2.303953000	2.998521000
P	6.192724000	4.367661000	1.393794000
P	10.419571000	0.376394000	4.711009000
P	9.727913000	2.718842000	1.196786000
P	6.943978000	1.875155000	4.776753000
F	6.262180000	7.339606000	2.396127000
F	7.895670000	8.402740000	4.205415000
F	9.704630000	6.848273000	5.486374000
F	9.877443000	4.250897000	4.971026000
F	10.627047000	-2.600931000	3.649794000
F	9.144339000	-3.734345000	1.754335000
F	7.298172000	-2.282103000	0.413695000
F	6.933380000	0.300386000	0.960714000
C	8.136959000	4.336912000	3.355703000
C	7.202246000	5.165631000	2.692453000
C	7.137312000	6.521553000	2.993986000

C	7.970921000	7.100886000	3.930029000
C	8.889753000	6.303817000	4.586151000
C	8.947971000	4.954265000	4.292442000
C	8.590978000	0.281587000	2.658924000
C	9.535546000	-0.496531000	3.366024000
C	9.714857000	-1.834597000	3.038822000
C	8.966500000	-2.446677000	2.051213000
C	8.026638000	-1.701274000	1.364524000
C	7.863692000	-0.363870000	1.676575000
C	5.461258000	2.906711000	4.900850000
H	4.922323000	2.654520000	5.815726000
H	4.816931000	2.734862000	4.040930000
H	5.742417000	3.958590000	4.919760000
C	7.695213000	2.102149000	6.411063000
H	8.048563000	3.128971000	6.502916000
H	8.546232000	1.432238000	6.519769000
H	6.952274000	1.900361000	7.184719000
C	6.248482000	0.202595000	4.837254000
H	5.625558000	0.088265000	5.725758000
H	7.048082000	-0.535391000	4.848285000
H	5.644440000	0.041154000	3.943769000
C	10.698085000	4.246260000	1.305465000
H	11.348702000	4.192064000	2.178679000
H	10.030256000	5.098510000	1.422662000
H	11.304002000	4.371748000	0.406751000
C	8.872006000	2.850586000	-0.397090000
H	8.162565000	3.674252000	-0.386220000
H	8.323571000	1.926782000	-0.580223000
H	9.607305000	3.005166000	-1.188947000
C	10.985027000	1.472639000	0.804760000
H	11.512342000	1.775241000	-0.101447000
H	10.505759000	0.507822000	0.642316000
H	11.693779000	1.377203000	1.623252000
C	4.471063000	4.348339000	2.003971000
C	3.902387000	5.287286000	2.862535000
H	4.480553000	6.132809000	3.208015000
C	2.593060000	5.141542000	3.292952000
H	2.165819000	5.874056000	3.967774000
C	1.829190000	4.063289000	2.863650000
H	0.806357000	3.952856000	3.203721000
C	2.382247000	3.126940000	2.003061000
H	1.795179000	2.280607000	1.666791000
C	3.698455000	3.265644000	1.584430000
H	4.140484000	2.518115000	0.934336000
C	6.123584000	5.632997000	0.074169000
C	7.301469000	6.259366000	-0.338470000
H	8.224259000	6.086482000	0.203960000
C	7.304711000	7.119386000	-1.424651000
H	8.227302000	7.605032000	-1.720859000
C	6.132429000	7.360100000	-2.129554000
H	6.134210000	8.032169000	-2.979228000

C	4.958811000	6.735098000	-1.733516000
H	4.036880000	6.918748000	-2.273125000
C	4.953229000	5.878239000	-0.641789000
H	4.026390000	5.404487000	-0.341011000
C	10.750053000	-0.908435000	5.965685000
C	11.993182000	-1.037038000	6.582174000
H	12.834155000	-0.451565000	6.230864000
C	12.169954000	-1.917508000	7.641181000
H	13.146114000	-2.007971000	8.103532000
C	11.108952000	-2.682510000	8.102005000
H	11.249406000	-3.373211000	8.924738000
C	9.862918000	-2.554272000	7.500456000
H	9.025586000	-3.145675000	7.852369000
C	9.683753000	-1.669004000	6.450834000
H	8.703799000	-1.578430000	5.995960000
C	12.075988000	0.713673000	4.021370000
C	12.524382000	2.030584000	4.093152000
H	11.899766000	2.778433000	4.567768000
C	13.746547000	2.393067000	3.541244000
H	14.080138000	3.422474000	3.597209000
C	14.530475000	1.438646000	2.911741000
H	15.480488000	1.719020000	2.472469000
C	14.096230000	0.119261000	2.844931000
H	14.708791000	-0.628986000	2.355804000
C	12.878871000	-0.242426000	3.397938000
H	12.548713000	-1.271297000	3.337630000

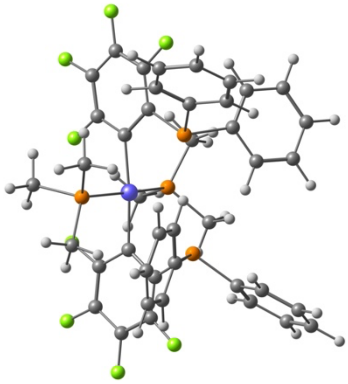

Figure S80. Optimized molecular structure of *syn*-**2Ni**[a].

PBE0:

final single point energy: -5320.388916660871 a.u.

final Gibbs free energy: -5319.77301300 a.u.

Table S13. Atomic coordinates for optimized structure of *syn*-**2Ni**[a].

Ni	8.556276000	2.504137000	3.089486000
P	10.366184000	4.322658000	5.182683000
P	11.069259000	0.565758000	3.948439000
P	9.593078000	3.132552000	1.260965000
P	7.205677000	1.886390000	4.704324000
F	9.343410000	7.315452000	5.346586000
F	7.220679000	8.352358000	4.122747000

F	5.720373000	6.823524000	2.466759000
F	6.346936000	4.273159000	2.027107000
F	10.750048000	-2.385088000	2.828196000
F	8.814817000	-3.290610000	1.243321000
F	6.788028000	-1.658829000	0.496997000
F	6.681326000	0.861687000	1.355350000
C	8.186661000	4.351296000	3.523458000
C	8.955353000	5.164270000	4.386331000
C	8.622749000	6.501583000	4.564449000
C	7.532946000	7.070323000	3.933129000
C	6.769871000	6.288688000	3.086261000
C	7.118317000	4.964564000	2.895378000
C	8.737922000	0.665179000	2.522864000
C	9.790618000	-0.199934000	2.895672000
C	9.790970000	-1.522166000	2.469625000
C	8.792694000	-2.023210000	1.655957000
C	7.756096000	-1.189475000	1.280475000
C	7.745352000	0.117582000	1.731411000
C	5.468457000	2.173110000	4.247981000
H	4.818735000	1.820164000	5.050974000
H	5.237862000	1.636143000	3.328914000
H	5.298160000	3.235902000	4.085583000
C	7.332003000	2.746995000	6.296391000
H	7.143631000	3.808405000	6.143960000
H	8.332097000	2.619284000	6.709002000
H	6.596172000	2.342913000	6.993629000
C	7.167422000	0.144123000	5.219813000
H	6.387757000	0.008009000	5.971143000
H	8.130076000	-0.141416000	5.640353000
H	6.960986000	-0.493250000	4.360813000
C	10.147378000	4.855237000	1.095400000
H	10.904885000	5.075384000	1.845482000
H	9.306867000	5.532676000	1.241336000
H	10.567670000	5.004299000	0.099370000
C	8.521871000	2.949215000	-0.197794000
H	7.611903000	3.531483000	-0.060171000
H	8.252053000	1.903193000	-0.331513000
H	9.052084000	3.303002000	-1.083935000
C	11.075540000	2.212554000	0.763306000
H	10.811927000	1.167537000	0.609171000
H	11.832485000	2.276897000	1.544190000
C	11.825466000	4.925071000	4.256980000
C	12.078131000	6.268507000	3.974546000
H	11.406801000	7.035658000	4.336727000
C	13.185540000	6.629777000	3.224921000
H	13.367716000	7.675156000	3.004831000
C	14.060925000	5.657065000	2.753869000
H	14.923506000	5.944168000	2.164175000
C	13.827152000	4.321739000	3.042016000
H	14.504712000	3.557074000	2.680353000
C	12.713633000	3.959574000	3.789474000

H	12.511660000	2.914552000	3.995053000
C	10.586576000	5.115100000	6.811417000
C	9.490808000	5.386858000	7.632166000
H	8.484635000	5.270816000	7.248352000
C	9.671511000	5.826547000	8.933525000
H	8.806700000	6.045240000	9.549207000
C	10.951632000	5.990876000	9.446873000
H	11.092170000	6.334882000	10.464497000
C	12.048057000	5.714039000	8.643233000
H	13.052334000	5.841119000	9.030605000
C	11.868079000	5.278933000	7.338004000
H	12.735052000	5.070775000	6.721921000
C	10.720515000	-0.083481000	5.622926000
C	10.639539000	0.850896000	6.652315000
H	10.787716000	1.900183000	6.423273000
C	10.341995000	0.453402000	7.949719000
H	10.277195000	1.193998000	8.738347000
C	10.120065000	-0.886098000	8.227599000
H	9.879703000	-1.200559000	9.236344000
C	10.207872000	-1.828063000	7.208041000
H	10.038570000	-2.876653000	7.423123000
C	10.510127000	-1.431964000	5.915539000
H	10.578076000	-2.175269000	5.132285000
C	12.643169000	-0.260402000	3.535485000
C	12.997378000	-0.519741000	2.210642000
H	12.274518000	-0.372920000	1.417611000
C	14.262640000	-0.986995000	1.893483000
H	14.512157000	-1.195295000	0.859494000
C	15.205256000	-1.192498000	2.892562000
H	16.194151000	-1.558367000	2.643809000
C	14.867439000	-0.928207000	4.212024000
H	15.592781000	-1.087032000	5.001586000
C	13.599002000	-0.465310000	4.531069000
H	13.349370000	-0.267389000	5.566991000
H	11.473212000	2.628585000	-0.163864000

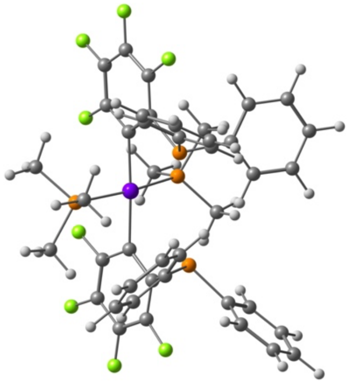

Figure S81. Optimized molecular structure of *syn*-**2Pd**[a].

PBE0:

final single point energy: -8896.650588968378 a.u.
final Gibbs free energy: -8896.03646339 a.u.

Table S14. Atomic coordinates for optimized structure of *syn*-**2Pd**[a].

Pd	8.521672000	2.458023000	2.999627000
P	10.274357000	4.322217000	5.093815000
P	11.214381000	0.590571000	3.826815000
P	9.598856000	3.144340000	1.080152000
P	7.122924000	1.791763000	4.717678000
F	9.233545000	7.325854000	5.392898000
F	7.133734000	8.403669000	4.164950000
F	5.649871000	6.934838000	2.448393000
F	6.253155000	4.378576000	1.967317000
F	10.945989000	-2.457440000	3.035222000
F	9.062035000	-3.551204000	1.506831000
F	6.983008000	-2.059781000	0.625648000
F	6.777815000	0.514492000	1.286683000
C	8.090620000	4.428825000	3.466246000
C	8.858419000	5.207418000	4.354561000
C	8.525603000	6.539124000	4.572163000
C	7.445776000	7.127091000	3.942252000
C	6.688551000	6.373927000	3.063419000
C	7.028117000	5.052377000	2.843148000
C	8.837556000	0.481627000	2.464197000
C	9.928001000	-0.302847000	2.894353000
C	9.974384000	-1.656425000	2.583327000
C	8.997093000	-2.254530000	1.809296000
C	7.934191000	-1.491803000	1.364304000
C	7.872052000	-0.154470000	1.711746000
C	5.504914000	2.615667000	4.688283000
H	4.871927000	2.219581000	5.484531000
H	5.027085000	2.447157000	3.723447000
H	5.640363000	3.687330000	4.829617000
C	7.712837000	2.114919000	6.398802000
H	7.875537000	3.184052000	6.521649000
H	8.656017000	1.598057000	6.567333000
H	6.970740000	1.775771000	7.124126000

C	6.654122000	0.039143000	4.776629000
H	6.017155000	-0.140480000	5.644580000
H	7.545511000	-0.582070000	4.841834000
H	6.110879000	-0.222091000	3.868829000
C	10.109812000	4.880837000	0.951464000
H	10.852110000	5.103582000	1.716865000
H	9.247411000	5.528480000	1.105668000
H	10.535706000	5.066020000	-0.036328000
C	8.500299000	2.940852000	-0.351432000
H	7.594199000	3.525827000	-0.196097000
H	8.225089000	1.891633000	-0.451571000
H	9.002839000	3.276937000	-1.260575000
C	11.090575000	2.247384000	0.577952000
H	10.852003000	1.188697000	0.480727000
H	11.862013000	2.367041000	1.337775000
C	11.731205000	5.018852000	4.228601000
C	11.993900000	6.382749000	4.090166000
H	11.332964000	7.112110000	4.538304000
C	13.101019000	6.813698000	3.377482000
H	13.290780000	7.875358000	3.270535000
C	13.967575000	5.890982000	2.801538000
H	14.831161000	6.233391000	2.243645000
C	13.723360000	4.534391000	2.945320000
H	14.393932000	3.807824000	2.501343000
C	12.609064000	4.102770000	3.653781000
H	12.398330000	3.042683000	3.746225000
C	10.465182000	4.986231000	6.780982000
C	9.359359000	5.282188000	7.578686000
H	8.364523000	5.286174000	7.147732000
C	9.515811000	5.594515000	8.920053000
H	8.644346000	5.831656000	9.519269000
C	10.780353000	5.609517000	9.493364000
H	10.901821000	5.854186000	10.541689000
C	11.887400000	5.313534000	8.710159000
H	12.880197000	5.326475000	9.144834000
C	11.731629000	5.002806000	7.367531000
H	12.605613000	4.773105000	6.768403000
C	10.845134000	0.223367000	5.573158000
C	11.350588000	1.114072000	6.521452000
H	11.938605000	1.964200000	6.195042000
C	11.090604000	0.934379000	7.871329000
H	11.484754000	1.639309000	8.593879000
C	10.306818000	-0.132986000	8.289217000
H	10.088399000	-0.267408000	9.341864000
C	9.798128000	-1.023974000	7.353717000
H	9.186240000	-1.858633000	7.675079000
C	10.070366000	-0.850139000	6.004255000
H	9.671528000	-1.553907000	5.284012000
C	12.762424000	-0.340981000	3.542067000
C	13.241483000	-0.433212000	2.233262000
H	12.649428000	-0.044621000	1.412707000

C	14.462021000	-1.030229000	1.965489000
H	14.810448000	-1.103938000	0.941818000
C	15.240070000	-1.526154000	3.004534000
H	16.198114000	-1.987374000	2.796725000
C	14.780524000	-1.424700000	4.308963000
H	15.378486000	-1.809477000	5.127036000
C	13.550199000	-0.839144000	4.576786000
H	13.200880000	-0.778502000	5.600149000
H	11.453289000	2.632351000	-0.376812000

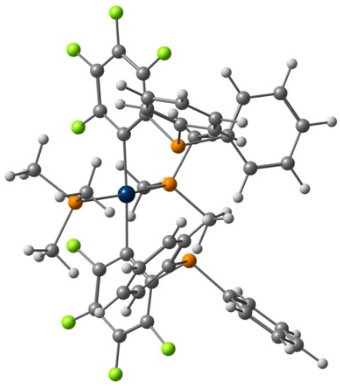

Figure S82. Optimized molecular structure of *syn*-**2Pt**[a].

PBE0:

final single point energy: -22831.688559988750 a.u.

final Gibbs free energy: -22831.07383836 a.u.

Table S15. Atomic coordinates for optimized structure of *syn*-**2Pt**[a].

Pt	8.505008000	2.459550000	2.981562000
P	10.274074000	4.305649000	5.104065000
P	11.200233000	0.603923000	3.834144000
P	9.581637000	3.142164000	1.073457000
P	7.121418000	1.795296000	4.695599000
F	9.242058000	7.298409000	5.443176000
F	7.151761000	8.407789000	4.228760000
F	5.673263000	6.974156000	2.475630000
F	6.263709000	4.433164000	1.952250000
F	10.957605000	-2.441006000	3.061494000
F	9.097106000	-3.564183000	1.525556000
F	7.013603000	-2.092952000	0.616718000
F	6.779730000	0.474780000	1.251997000
C	8.092925000	4.432482000	3.466851000
C	8.858385000	5.198394000	4.373192000
C	8.531013000	6.527884000	4.609889000
C	7.458027000	7.133529000	3.986050000
C	6.704292000	6.398858000	3.089880000
C	7.037337000	5.079598000	2.847767000
C	8.828462000	0.481099000	2.455351000
C	9.922906000	-0.297364000	2.897140000
C	9.983193000	-1.652133000	2.594870000
C	9.019258000	-2.265693000	1.817068000
C	7.955475000	-1.513602000	1.358065000

C	7.877206000	-0.174337000	1.694427000
C	5.505201000	2.617959000	4.652168000
H	4.868375000	2.223992000	5.446092000
H	5.034266000	2.445123000	3.684824000
H	5.638842000	3.690134000	4.791284000
C	7.703309000	2.122290000	6.376966000
H	7.859077000	3.192564000	6.497795000
H	8.648508000	1.610728000	6.549435000
H	6.959534000	1.780558000	7.099021000
C	6.660672000	0.042635000	4.746569000
H	6.022850000	-0.139322000	5.613039000
H	7.553363000	-0.576313000	4.813125000
H	6.119053000	-0.217854000	3.837693000
C	10.089058000	4.878149000	0.958771000
H	10.824167000	5.098209000	1.731803000
H	9.224701000	5.524577000	1.106431000
H	10.523595000	5.066629000	-0.024304000
C	8.494640000	2.934928000	-0.364028000
H	7.587278000	3.519615000	-0.215627000
H	8.221362000	1.885079000	-0.462918000
H	9.003994000	3.269186000	-1.269807000
C	11.077814000	2.245585000	0.590994000
H	10.841479000	1.186344000	0.494655000
H	11.840476000	2.369787000	1.358809000
C	11.722025000	5.001265000	4.222018000
C	11.966852000	6.365285000	4.054946000
H	11.297406000	7.095475000	4.488833000
C	13.066874000	6.796198000	3.331302000
H	13.242323000	7.857885000	3.202464000
C	13.944203000	5.873440000	2.772086000
H	14.802011000	6.215587000	2.205198000
C	13.717575000	4.517037000	2.943709000
H	14.396092000	3.790189000	2.512422000
C	12.610357000	4.085533000	3.663256000
H	12.412442000	3.025042000	3.775679000
C	10.484525000	4.971790000	6.788351000
C	9.387687000	5.250919000	7.604765000
H	8.386286000	5.246949000	7.189339000
C	9.560579000	5.557527000	8.945276000
H	8.695480000	5.781566000	9.558630000
C	10.833294000	5.583365000	9.500101000
H	10.967622000	5.823202000	10.547971000
C	11.931522000	5.304560000	8.698573000
H	12.930598000	5.326412000	9.118300000
C	11.759169000	4.999765000	7.356488000
H	12.626764000	4.783348000	6.743330000
C	10.828455000	0.232929000	5.579251000
C	11.333729000	1.120459000	6.530649000
H	11.924631000	1.969679000	6.207475000
C	11.069908000	0.939042000	7.879518000
H	11.463864000	1.641899000	8.604215000

C	10.282760000	-0.127333000	8.293704000
H	10.061382000	-0.263102000	9.345570000
C	9.775366000	-1.016043000	7.355395000
H	9.161660000	-1.850547000	7.673718000
C	10.051369000	-0.840254000	6.006900000
H	9.653801000	-1.542719000	5.284723000
C	12.753447000	-0.321536000	3.554356000
C	13.233525000	-0.418963000	2.246205000
H	12.637842000	-0.042303000	1.422733000
C	14.459535000	-1.006402000	1.982342000
H	14.808205000	-1.084376000	0.959053000
C	15.242709000	-1.487352000	3.024599000
H	16.205018000	-1.941005000	2.819878000
C	14.782277000	-1.380733000	4.328297000
H	15.383849000	-1.754013000	5.149072000
C	13.546519000	-0.804897000	4.592179000
H	13.197065000	-0.740469000	5.615260000
H	11.448998000	2.627715000	-0.361361000

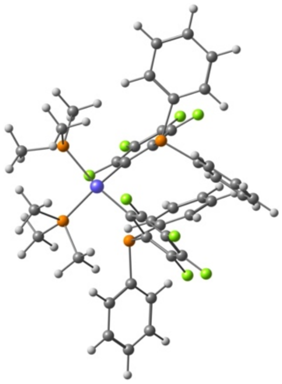

Figure S83. Optimized molecular structure of *anti*-**2Ni^b^**.

PBE0:

final single point energy: -5320.379298358783 a.u.
final Gibbs free energy: -5319.76236875 a.u.

Table S16. Atomic coordinates for optimized structure of *anti*-**2Ni^b^**.

Ni	0.292855000	-0.805794000	-0.193676000
C	1.384117000	4.192802000	-4.120848000
C	0.493935000	5.082269000	-3.532911000
C	1.976433000	3.197909000	-3.359973000
C	-0.962186000	1.429866000	-3.453942000
C	-2.016065000	2.213034000	-3.025990000
C	0.214817000	4.978693000	-2.178022000
C	-0.352840000	0.571214000	-2.559559000
C	1.708045000	3.091244000	-1.994346000
C	6.003030000	3.831650000	-1.044069000
C	4.672951000	3.495683000	-1.253669000
C	-2.452966000	2.081134000	-1.722411000
C	0.824313000	3.994443000	-1.413519000
C	-0.746109000	0.427998000	-1.240579000
C	6.850816000	2.958499000	-0.377344000

C	4.172310000	2.275382000	-0.807895000
C	-6.276094000	-0.309354000	-0.324280000
C	-1.857428000	1.193545000	-0.832623000
C	-7.119031000	0.688408000	0.147355000
C	-4.909394000	-0.219832000	-0.111933000
C	-6.582048000	1.774349000	0.824353000
C	6.362641000	1.737137000	0.068322000
C	5.039001000	1.394841000	-0.158836000
C	-4.357096000	0.878625000	0.548389000
C	-5.211557000	1.872029000	1.019224000
C	1.707031000	1.729588000	0.559221000
C	-1.702903000	3.538214000	1.233308000
C	2.000601000	2.717074000	1.493246000
C	0.892911000	0.637937000	0.922304000
C	-1.426817000	4.633981000	2.038312000
C	-2.263876000	2.385520000	1.772349000
C	1.550749000	2.645996000	2.797119000
C	0.481672000	0.589070000	2.242625000
C	-1.722511000	4.596371000	3.393023000
C	-2.549493000	2.353501000	3.138396000
C	0.796524000	1.553985000	3.180008000
C	-2.292347000	3.453825000	3.940097000
H	1.602535000	4.262277000	-5.179902000
H	0.015532000	5.848598000	-4.130932000
H	2.646450000	2.486984000	-3.832378000
H	6.375420000	4.786822000	-1.396026000
H	-0.481852000	5.665568000	-1.711232000
H	4.018335000	4.195366000	-1.758147000
H	-6.684752000	-1.165495000	-0.848362000
H	-8.188931000	0.616979000	-0.008007000
H	0.602152000	3.924545000	-0.357750000
H	7.887296000	3.225697000	-0.209298000
H	-4.262192000	-1.016343000	-0.462065000
H	-7.232631000	2.557325000	1.196627000
H	-1.471028000	3.579298000	0.178071000
H	-4.804989000	2.734171000	1.533097000
H	7.017081000	1.044242000	0.584312000
H	4.674249000	0.428851000	0.172154000
H	-0.977805000	5.519410000	1.603084000
H	-1.504582000	5.450778000	4.022521000
H	-2.966668000	1.453792000	3.578839000
H	-2.519951000	3.413248000	4.998738000
F	-0.560796000	1.503071000	-4.720586000
F	-2.598038000	3.071162000	-3.864216000
F	0.633011000	-0.198505000	-3.070792000
F	-3.478584000	2.858543000	-1.355822000
F	2.730751000	3.791336000	1.171999000
F	1.840447000	3.604139000	3.677988000
F	-0.206699000	-0.476020000	2.709148000
F	0.399396000	1.440880000	4.445197000
P	2.460466000	1.688732000	-1.104878000

P	-0.809675000	-2.455564000	-1.143858000
P	-2.544726000	0.852553000	0.825834000
P	1.872873000	-2.065452000	0.684505000
C	1.520256000	-3.700963000	1.408556000
H	0.878429000	-3.561413000	2.279697000
H	1.015419000	-4.362412000	0.708628000
H	2.454652000	-4.167142000	1.725961000
C	2.910047000	-1.382795000	2.014724000
H	3.278433000	-0.395214000	1.745212000
H	2.322990000	-1.296978000	2.928627000
H	3.750480000	-2.056446000	2.190790000
C	3.104470000	-2.398063000	-0.612333000
H	3.475516000	-1.442217000	-0.984564000
H	3.934244000	-2.981948000	-0.209922000
H	2.649390000	-2.934026000	-1.443468000
C	-1.836596000	-3.247024000	0.132509000
H	-1.213985000	-3.654955000	0.926714000
H	-2.488772000	-2.487316000	0.565468000
H	-2.442068000	-4.043395000	-0.304061000
C	0.044258000	-3.838307000	-1.969434000
H	-0.690298000	-4.570561000	-2.309163000
H	0.581123000	-3.444609000	-2.833781000
H	0.758497000	-4.331412000	-1.314382000
C	-2.045756000	-2.073993000	-2.423244000
H	-2.712129000	-1.282747000	-2.085926000
H	-1.544222000	-1.741628000	-3.331549000
H	-2.621538000	-2.975185000	-2.641156000

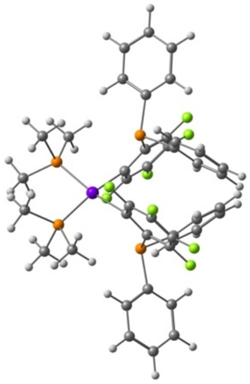

Figure S84. Optimized molecular structure of *anti*-**2Pd**^{b}.

PBE0:

final single point energy: -8896.642000039119 a.u.

final Gibbs free energy: -8896.02645577 a.u.

Table S17. Atomic coordinates for optimized structure of *anti*-**2Pd**^{b}.

Pd	0.298330000	-0.807045000	-0.184494000
C	1.394847000	4.191524000	-4.154004000
C	0.488824000	5.072070000	-3.577186000
C	1.997432000	3.210397000	-3.383294000
C	-1.103879000	1.573244000	-3.478620000
C	-2.160906000	2.326681000	-3.006845000

C	0.203299000	4.972918000	-2.223258000
C	-0.460590000	0.700180000	-2.621951000
C	1.725379000	3.110515000	-2.017712000
C	6.087008000	3.754475000	-1.190838000
C	4.747644000	3.446247000	-1.385276000
C	-2.569717000	2.154293000	-1.699004000
C	0.823505000	4.003829000	-1.448395000
C	-0.833290000	0.514333000	-1.304257000
C	6.903244000	2.905984000	-0.456640000
C	4.207313000	2.278266000	-0.855283000
C	-6.287607000	-0.351820000	-0.315103000
C	-1.943516000	1.248815000	-0.849351000
C	-7.151878000	0.593564000	0.221875000
C	-4.920580000	-0.229387000	-0.123324000
C	-6.636002000	1.661247000	0.942680000
C	6.374037000	1.736492000	0.074125000
C	5.041261000	1.420786000	-0.135913000
C	-4.390741000	0.851621000	0.582988000
C	-5.265523000	1.792951000	1.117989000
C	1.771042000	1.827509000	0.565307000
C	-1.723689000	3.542807000	1.248786000
C	2.083933000	2.855840000	1.447466000
C	0.954448000	0.763949000	0.989677000
C	-1.443722000	4.631488000	2.061863000
C	-2.294745000	2.390245000	1.778556000
C	1.649939000	2.845985000	2.758276000
C	0.550938000	0.777330000	2.311261000
C	-1.745580000	4.587846000	3.415088000
C	-2.583483000	2.350988000	3.143902000
C	0.889368000	1.780688000	3.199615000
C	-2.323624000	3.445182000	3.952974000
H	1.617677000	4.256494000	-5.212407000
H	0.002417000	5.827143000	-4.183009000
H	2.678908000	2.505514000	-3.848174000
H	6.491241000	4.669647000	-1.607924000
H	-0.506828000	5.651720000	-1.765037000
H	4.117899000	4.128123000	-1.943129000
H	-6.679848000	-1.192619000	-0.875339000
H	-8.221844000	0.495845000	0.082056000
H	0.593993000	3.937329000	-0.393730000
H	7.946808000	3.152145000	-0.300846000
H	-4.254272000	-0.983947000	-0.527527000
H	-7.303295000	2.403829000	1.364834000
H	-1.485206000	3.589065000	0.194980000
H	-4.875968000	2.641443000	1.666632000
H	7.003239000	1.063971000	0.645517000
H	4.641049000	0.496376000	0.266809000
H	-0.986887000	5.516438000	1.633943000
H	-1.525190000	5.437167000	4.050576000
H	-3.006186000	1.450821000	3.577982000
H	-2.555084000	3.399214000	5.010551000

F	-0.730236000	1.691525000	-4.750237000
F	-2.771521000	3.198774000	-3.808761000
F	0.540867000	-0.019209000	-3.169192000
F	-3.595251000	2.909101000	-1.288419000
F	2.814956000	3.909934000	1.067323000
F	1.958042000	3.840444000	3.590535000
F	-0.165959000	-0.241385000	2.829041000
F	0.505316000	1.732077000	4.472669000
P	2.480543000	1.719221000	-1.113060000
P	-0.790741000	-2.532171000	-1.274887000
P	-2.572775000	0.863567000	0.821278000
P	1.891114000	-2.137321000	0.844695000
C	1.432847000	-3.750727000	1.550042000
H	0.706325000	-3.592222000	2.348028000
H	0.988436000	-4.400804000	0.798795000
H	2.318268000	-4.238354000	1.962070000
C	2.849218000	-1.436828000	2.220843000
H	3.269080000	-0.474121000	1.934279000
H	2.202160000	-1.292321000	3.085148000
H	3.653031000	-2.126907000	2.483889000
C	3.197781000	-2.530678000	-0.356122000
H	3.622683000	-1.595617000	-0.723172000
H	3.980979000	-3.128709000	0.113761000
H	2.782363000	-3.073250000	-1.203850000
C	-1.880275000	-3.384226000	-0.095485000
H	-1.298946000	-3.796890000	0.727518000
H	-2.582589000	-2.656441000	0.312871000
H	-2.431281000	-4.184752000	-0.592798000
C	0.161855000	-3.870865000	-2.056109000
H	-0.518652000	-4.605557000	-2.490438000
H	0.782840000	-3.444827000	-2.845232000
H	0.808564000	-4.368710000	-1.336134000
C	-1.943454000	-2.121580000	-2.618025000
H	-2.646806000	-1.358212000	-2.289944000
H	-1.389541000	-1.740231000	-3.475001000
H	-2.485655000	-3.022414000	-2.911362000

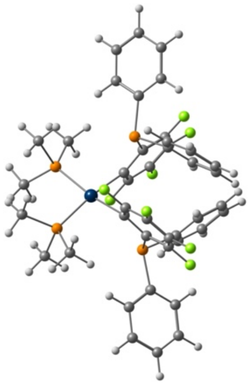

Figure S85. Optimized molecular structure of *anti*-**2Pt**$^{\mathbf{b}}$.

PBE0:

final single point energy: -22831.681436409541 a.u.

final Gibbs free energy: -22831.06501018 a.u.

Table S18. Atomic coordinates for optimized structure of *anti*-**2Pt**$^{\mathbf{b}}$.

Pt	0.300650000	-0.799401000	-0.185177000
C	1.413393000	4.191686000	-4.175205000
C	0.502189000	5.070720000	-3.604273000
C	2.012321000	3.211276000	-3.400786000
C	-1.131531000	1.603403000	-3.469483000
C	-2.188667000	2.347148000	-2.984068000
C	0.207519000	4.970488000	-2.252369000
C	-0.473665000	0.727989000	-2.626683000
C	1.731696000	3.110752000	-2.036880000
C	6.107632000	3.709624000	-1.220659000
C	4.764965000	3.415469000	-1.414362000
C	-2.582543000	2.162270000	-1.673849000
C	0.823984000	4.002221000	-1.473607000
C	-0.830524000	0.527067000	-1.303221000
C	6.911783000	2.860811000	-0.473643000
C	4.209164000	2.261222000	-0.870608000
C	-6.265901000	-0.379265000	-0.321164000
C	-1.943509000	1.254766000	-0.836271000
C	-7.141260000	0.542222000	0.238990000
C	-4.900069000	-0.242218000	-0.131460000
C	-6.637203000	1.601145000	0.980625000
C	6.366879000	1.705014000	0.071119000
C	5.030849000	1.403033000	-0.138018000
C	-4.382279000	0.830352000	0.596457000
C	-5.267966000	1.747853000	1.154290000
C	1.771049000	1.839412000	0.549709000
C	-1.725618000	3.540651000	1.264841000
C	2.090525000	2.876593000	1.418694000
C	0.953193000	0.778620000	0.986574000
C	-1.451468000	4.630259000	2.078505000
C	-2.296994000	2.387147000	1.792610000
C	1.664640000	2.883774000	2.731672000
C	0.557872000	0.811574000	2.313784000
C	-1.759255000	4.586820000	3.430441000

C	-2.590721000	2.347839000	3.156962000
C	0.904332000	1.824885000	3.187373000
C	-2.336862000	3.443112000	3.966476000
H	1.643229000	4.257223000	-5.232077000
H	0.018647000	5.825162000	-4.213157000
H	2.697538000	2.507321000	-3.861519000
H	6.523965000	4.614347000	-1.648501000
H	-0.507053000	5.647708000	-1.798774000
H	4.144991000	4.097963000	-1.982297000
H	-6.648439000	-1.212975000	-0.898459000
H	-8.210267000	0.432889000	0.100538000
H	0.586477000	3.934589000	-0.420731000
H	7.957951000	3.096101000	-0.318585000
H	-4.225431000	-0.978328000	-0.555117000
H	-7.312867000	2.325609000	1.420575000
H	-1.481795000	3.586837000	0.212210000
H	-4.888384000	2.590005000	1.719395000
H	6.986307000	1.032340000	0.652924000
H	4.618276000	0.489116000	0.275968000
H	-0.994414000	5.515786000	1.652068000
H	-1.543491000	5.436979000	4.066383000
H	-3.012593000	1.446782000	3.589961000
H	-2.572624000	3.397089000	5.023101000
F	-0.768415000	1.732427000	-4.742638000
F	-2.811764000	3.221108000	-3.773742000
F	0.524865000	0.022941000	-3.193009000
F	-3.608838000	2.906752000	-1.247770000
F	2.822756000	3.923304000	1.022067000
F	1.978951000	3.886778000	3.550786000
F	-0.158587000	-0.194765000	2.851073000
F	0.525026000	1.790169000	4.461760000
P	2.477845000	1.717001000	-1.128816000
P	-0.768033000	-2.516368000	-1.274576000
P	-2.564460000	0.858651000	0.835366000
P	1.863468000	-2.129041000	0.850665000
C	1.372753000	-3.725257000	1.569656000
H	0.629876000	-3.547271000	2.348104000
H	0.941190000	-4.384490000	0.819024000
H	2.244649000	-4.212708000	2.009206000
C	2.808000000	-1.422952000	2.232243000
H	3.240297000	-0.466349000	1.943766000
H	2.153986000	-1.268520000	3.089306000
H	3.603003000	-2.117990000	2.507631000
C	3.187605000	-2.553168000	-0.318376000
H	3.637183000	-1.627420000	-0.679405000
H	3.948389000	-3.161764000	0.173758000
H	2.783552000	-3.092845000	-1.172939000
C	-1.875687000	-3.380440000	-0.122893000
H	-1.311149000	-3.794570000	0.710542000
H	-2.590187000	-2.658207000	0.273605000
H	-2.411071000	-4.180593000	-0.637135000

C	0.200488000	-3.844526000	-2.051007000
H	-0.472456000	-4.573932000	-2.504993000
H	0.835358000	-3.409857000	-2.824105000
H	0.833048000	-4.351142000	-1.324859000
C	-1.899915000	-2.099590000	-2.632276000
H	-2.609445000	-1.338145000	-2.313359000
H	-1.334919000	-1.718364000	-3.481828000
H	-2.437056000	-3.000661000	-2.933080000

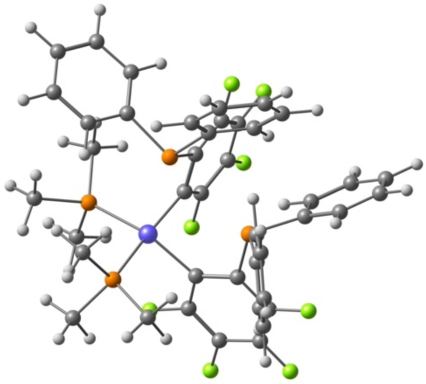

Figure S86. Optimized molecular structure of *syn*-**2Ni**[b].

PBE0:

final single point energy: -5320.380134800746 a.u.
final Gibbs free energy: -5319.76366454 a.u.

Table S19. Atomic coordinates for optimized structure of *syn*-**2Ni**[b].

Ni	-0.322595000	-0.936691000	-0.571065000
P	-1.270633000	1.338782000	0.906914000
P	2.017443000	1.448696000	0.425603000
P	-2.365762000	-1.665289000	-1.018127000
P	0.205638000	-2.457159000	0.923032000
F	-1.881007000	3.997665000	-0.874166000
F	-1.522577000	4.207297000	-3.502436000
F	-0.651991000	2.091792000	-4.940991000
F	-0.127875000	-0.245461000	-3.757154000
F	4.717129000	0.989149000	-1.010225000
F	5.328339000	-0.904972000	-2.789634000
F	3.475571000	-2.757165000	-3.481873000
F	1.047381000	-2.722594000	-2.415240000
C	-0.670026000	0.619295000	-1.616405000
C	-1.122795000	1.732526000	-0.873592000
C	-1.417808000	2.920928000	-1.523323000
C	-1.254265000	3.052957000	-2.891481000
C	-0.807635000	1.970250000	-3.624721000
C	-0.532756000	0.776124000	-2.978245000
C	1.524530000	-0.803084000	-1.096145000
C	2.504912000	0.144174000	-0.741670000
C	3.768743000	0.097528000	-1.322321000
C	4.111990000	-0.869751000	-2.244160000
C	3.164779000	-1.815170000	-2.593213000
C	1.910063000	-1.762581000	-2.018137000

C	-3.081659000	1.339226000	1.239323000
C	-4.012424000	2.198363000	0.654530000
H	-3.692948000	2.945012000	-0.058719000
C	-5.357586000	2.103968000	0.974982000
H	-6.068518000	2.773741000	0.505353000
C	-5.796073000	1.159426000	1.895298000
H	-6.848498000	1.089021000	2.143113000
C	-4.879851000	0.308928000	2.493702000
H	-5.210464000	-0.431197000	3.213026000
C	-3.534471000	0.396425000	2.160256000
H	-2.823321000	-0.281458000	2.617962000
C	-0.800745000	2.831450000	1.859052000
C	-1.129217000	2.840269000	3.216802000
H	-1.723731000	2.034694000	3.633859000
C	-0.699601000	3.864011000	4.045922000
H	-0.965380000	3.850302000	5.096495000
C	0.072182000	4.899788000	3.534111000
H	0.411878000	5.699163000	4.181673000
C	0.405920000	4.899186000	2.188117000
H	1.013225000	5.695904000	1.775085000
C	-0.023391000	3.872520000	1.358584000
H	0.262728000	3.887937000	0.317374000
C	2.680550000	2.952365000	-0.368408000
C	3.450125000	3.903626000	0.294165000
H	3.794233000	3.713945000	1.303648000
C	3.783414000	5.097693000	-0.331108000
H	4.386750000	5.827272000	0.196839000
C	3.352030000	5.358263000	-1.623802000
H	3.612465000	6.291468000	-2.108667000
C	2.583030000	4.413106000	-2.292125000
H	2.238257000	4.604990000	-3.301741000
C	2.246294000	3.223746000	-1.667731000
H	1.637321000	2.495707000	-2.192607000
C	3.066933000	1.233315000	1.902743000
C	4.155417000	0.369272000	2.001393000
H	4.469334000	-0.211932000	1.144726000
C	4.841235000	0.232244000	3.199691000
H	5.683327000	-0.447387000	3.259609000
C	4.455260000	0.961749000	4.315638000
H	4.994682000	0.854688000	5.249235000
C	3.368482000	1.821326000	4.231269000
H	3.051918000	2.388502000	5.098808000
C	2.672338000	1.945326000	3.038887000
H	1.809131000	2.599646000	2.987941000
C	0.097608000	-4.208445000	0.439736000
H	-0.919384000	-4.473779000	0.154653000
H	0.750668000	-4.376619000	-0.416579000
H	0.414549000	-4.843717000	1.268598000
C	-0.754726000	-2.362505000	2.464140000
H	-0.401097000	-3.114384000	3.171815000
H	-0.615936000	-1.368507000	2.891042000

H	-1.814662000	-2.514296000	2.273116000
C	1.896212000	-2.370833000	1.578406000
H	2.617993000	-2.521470000	0.776912000
H	2.064787000	-1.385924000	2.012378000
H	2.032972000	-3.133788000	2.346768000
C	-2.133108000	-2.828859000	-2.400453000
H	-1.649546000	-2.308557000	-3.226196000
H	-1.492522000	-3.654221000	-2.093580000
H	-3.100708000	-3.216619000	-2.725349000
C	-3.416039000	-2.635366000	0.114205000
H	-3.730796000	-2.010934000	0.949705000
H	-4.300553000	-2.980401000	-0.424461000
H	-2.881198000	-3.501451000	0.500535000
C	-3.606852000	-0.531442000	-1.713070000
H	-3.171518000	0.045373000	-2.527444000
H	-4.451188000	-1.113163000	-2.087015000
H	-3.954670000	0.157283000	-0.945312000

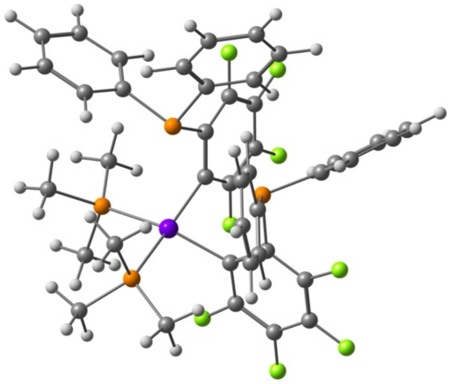

Figure S87. Optimized molecular structure of *syn*-**2Pd**[b].

PBE0:

final single point energy: -8896.638082095875 a.u.

final Gibbs free energy: -8896.02336603 a.u.

Table S20. Atomic coordinates for optimized structure of *syn*-**2Pd**[b].

Pd	-0.231738000	-1.045590000	-0.780098000
P	-1.349184000	1.415611000	0.900367000
P	1.978667000	1.488575000	0.382437000
P	-2.423513000	-1.783555000	-0.990957000
P	0.480429000	-2.737013000	0.640105000
F	-2.172895000	3.974264000	-0.807924000
F	-1.828347000	4.322023000	-3.422307000
F	-0.825409000	2.327000000	-4.948691000
F	-0.172123000	-0.023828000	-3.860105000
F	4.833732000	1.172312000	-0.764985000
F	5.669047000	-0.551356000	-2.625139000
F	3.962981000	-2.399571000	-3.622766000
F	1.456561000	-2.551036000	-2.755557000
C	-0.744596000	0.723251000	-1.686075000
C	-1.255978000	1.772970000	-0.893226000
C	-1.632122000	2.963074000	-1.500037000

C	-1.480444000	3.164126000	-2.860803000
C	-0.969602000	2.143255000	-3.638338000
C	-0.627189000	0.941987000	-3.039690000
C	1.740754000	-0.705331000	-1.282962000
C	2.632871000	0.259650000	-0.787166000
C	3.948069000	0.291141000	-1.242291000
C	4.407527000	-0.592592000	-2.196937000
C	3.533247000	-1.536952000	-2.704126000
C	2.233764000	-1.576556000	-2.237915000
C	-3.147299000	1.355455000	1.281384000
C	-4.169449000	1.911672000	0.514891000
H	-3.939117000	2.456543000	-0.390440000
C	-5.496049000	1.772537000	0.896489000
H	-6.277322000	2.205376000	0.282623000
C	-5.824325000	1.089676000	2.059852000
H	-6.861133000	0.986531000	2.356880000
C	-4.817064000	0.532798000	2.834322000
H	-5.061602000	-0.012400000	3.738377000
C	-3.493198000	0.652521000	2.437440000
H	-2.713448000	0.178789000	3.025061000
C	-0.891975000	2.968923000	1.764468000
C	-1.208049000	3.082511000	3.120287000
H	-1.813088000	2.323835000	3.602259000
C	-0.751032000	4.154088000	3.870888000
H	-1.010290000	4.220463000	4.921137000
C	0.039819000	5.133619000	3.283614000
H	0.402449000	5.968738000	3.870816000
C	0.360823000	5.030386000	1.938779000
H	0.980446000	5.782185000	1.464666000
C	-0.098927000	3.957743000	1.187912000
H	0.174717000	3.896579000	0.144787000
C	2.703403000	3.034636000	-0.265897000
C	3.512653000	3.897684000	0.464382000
H	3.837977000	3.621868000	1.460106000
C	3.911627000	5.112411000	-0.076924000
H	4.544881000	5.774263000	0.502627000
C	3.508539000	5.478909000	-1.352948000
H	3.821359000	6.427485000	-1.772701000
C	2.698169000	4.622243000	-2.088543000
H	2.373670000	4.899643000	-3.084822000
C	2.293021000	3.414116000	-1.545974000
H	1.645610000	2.757208000	-2.118574000
C	2.859491000	1.186512000	1.947559000
C	3.840986000	0.214785000	2.127387000
H	4.192590000	-0.364416000	1.283147000
C	4.379113000	-0.022299000	3.384559000
H	5.140399000	-0.784097000	3.506168000
C	3.951442000	0.715532000	4.479063000
H	4.375036000	0.531512000	5.459192000
C	2.973715000	1.687736000	4.311583000
H	2.628473000	2.266201000	5.160566000

C	2.423043000	1.912149000	3.060029000
H	1.641611000	2.655090000	2.944191000
C	-0.223429000	-4.414622000	0.608118000
H	-1.305567000	-4.392111000	0.723000000
H	0.019529000	-4.886325000	-0.344750000
H	0.207591000	-5.005181000	1.418780000
C	0.161859000	-2.168661000	2.336066000
H	0.514503000	-2.904388000	3.061337000
H	0.680731000	-1.221750000	2.491511000
H	-0.906844000	-2.000133000	2.469823000
C	2.254121000	-3.123621000	0.674293000
H	2.549292000	-3.579582000	-0.270677000
H	2.827967000	-2.209813000	0.814458000
H	2.460043000	-3.817280000	1.491487000
C	-2.382262000	-3.254914000	-2.061217000
H	-1.883143000	-2.992869000	-2.994701000
H	-1.825602000	-4.060538000	-1.586926000
H	-3.398645000	-3.592184000	-2.274705000
C	-3.450471000	-2.301011000	0.416901000
H	-3.735334000	-1.417736000	0.987340000
H	-4.350371000	-2.802185000	0.055188000
H	-2.902584000	-2.972055000	1.076441000
C	-3.568356000	-0.718268000	-1.912702000
H	-3.134780000	-0.482001000	-2.883989000
H	-4.519223000	-1.236234000	-2.051743000
H	-3.735568000	0.210097000	-1.370733000

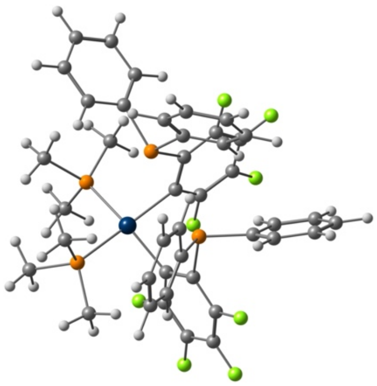

Figure S88. Optimized molecular structure of *syn*-**2Pt^b^**.

PBE0:

final single point energy: -22831.676375746130 a.u.

final Gibbs free energy: -22831.06090963 a.u.

Table S21. Atomic coordinates for optimized structure of *syn*-**2Pt^b^**.

Pt	-0.211778000	-1.076801000	-0.805735000
P	-1.358200000	1.419309000	0.908296000
P	1.955615000	1.481218000	0.373385000
P	-2.388949000	-1.803852000	-0.959168000
P	0.508795000	-2.764263000	0.583816000
F	-2.191226000	3.955115000	-0.806417000
F	-1.848738000	4.308778000	-3.418346000

F	-0.830510000	2.318768000	-4.945013000
F	-0.163316000	-0.022456000	-3.860845000
F	4.821853000	1.202206000	-0.721950000
F	5.714208000	-0.488621000	-2.584825000
F	4.051930000	-2.356718000	-3.620257000
F	1.546249000	-2.559755000	-2.797355000
C	-0.745327000	0.707980000	-1.682621000
C	-1.264153000	1.759412000	-0.890717000
C	-1.645197000	2.946963000	-1.498446000
C	-1.494327000	3.152233000	-2.858694000
C	-0.975687000	2.135678000	-3.635082000
C	-0.626395000	0.935805000	-3.037542000
C	1.760904000	-0.716009000	-1.304407000
C	2.634680000	0.261556000	-0.792099000
C	3.956924000	0.314881000	-1.224263000
C	4.447007000	-0.552458000	-2.177793000
C	3.595044000	-1.505374000	-2.704788000
C	2.289181000	-1.571684000	-2.260512000
C	-3.157579000	1.374100000	1.291587000
C	-4.180051000	1.923132000	0.520248000
H	-3.951242000	2.452399000	-0.394536000
C	-5.505988000	1.797263000	0.908823000
H	-6.286986000	2.224894000	0.290955000
C	-5.833946000	1.134499000	2.083769000
H	-6.870179000	1.041745000	2.386159000
C	-4.826761000	0.583965000	2.862786000
H	-5.070606000	0.054080000	3.776094000
C	-3.503789000	0.690890000	2.459490000
H	-2.724579000	0.222318000	3.052035000
C	-0.899448000	2.983195000	1.754400000
C	-1.209872000	3.106443000	3.110950000
H	-1.809864000	2.349515000	3.601736000
C	-0.753381000	4.184963000	3.851552000
H	-1.008712000	4.258296000	4.902314000
C	0.032338000	5.162464000	3.253907000
H	0.394804000	6.003089000	3.833311000
C	0.348450000	5.049516000	1.908839000
H	0.964311000	5.799085000	1.426442000
C	-0.111383000	3.969900000	1.167879000
H	0.158874000	3.901969000	0.124418000
C	2.677568000	3.032883000	-0.265391000
C	3.476347000	3.899224000	0.472320000
H	3.796006000	3.623524000	1.469868000
C	3.871807000	5.117399000	-0.063561000
H	4.496626000	5.781953000	0.522024000
C	3.475606000	5.484296000	-1.341650000
H	3.785272000	6.435861000	-1.756957000
C	2.676177000	4.624090000	-2.084982000
H	2.357202000	4.901556000	-3.083022000
C	2.274748000	3.412287000	-1.547825000
H	1.635788000	2.752527000	-2.126589000

C	2.820920000	1.178123000	1.946351000
C	3.782910000	0.189250000	2.137677000
H	4.129900000	-0.399632000	1.298167000
C	4.307193000	-0.052348000	3.399800000
H	5.053625000	-0.827268000	3.530235000
C	3.884887000	0.697803000	4.487976000
H	4.297639000	0.510325000	5.472069000
C	2.926445000	1.687055000	4.308946000
H	2.585535000	2.275450000	5.152847000
C	2.389523000	1.916520000	3.052405000
H	1.622806000	2.673158000	2.927932000
C	-0.207790000	-4.435010000	0.551045000
H	-1.286538000	-4.409486000	0.691672000
H	0.012563000	-4.900022000	-0.410436000
H	0.238975000	-5.033120000	1.347222000
C	0.240394000	-2.209842000	2.291876000
H	0.599687000	-2.958812000	2.999816000
H	0.778666000	-1.273225000	2.443399000
H	-0.821575000	-2.025800000	2.454271000
C	2.277916000	-3.169175000	0.569682000
H	2.544207000	-3.626752000	-0.382955000
H	2.867546000	-2.264218000	0.701079000
H	2.492974000	-3.868620000	1.379195000
C	-2.413339000	-3.280336000	-2.019875000
H	-1.951929000	-3.028181000	-2.975076000
H	-1.849820000	-4.093304000	-1.567810000
H	-3.443523000	-3.600918000	-2.186828000
C	-3.372592000	-2.289787000	0.488193000
H	-3.604218000	-1.398704000	1.070222000
H	-4.302624000	-2.758814000	0.161921000
H	-2.823131000	-2.981628000	1.124192000
C	-3.544720000	-0.726792000	-1.850311000
H	-3.136004000	-0.498526000	-2.834259000
H	-4.503285000	-1.236728000	-1.961072000
H	-3.689709000	0.204365000	-1.307501000

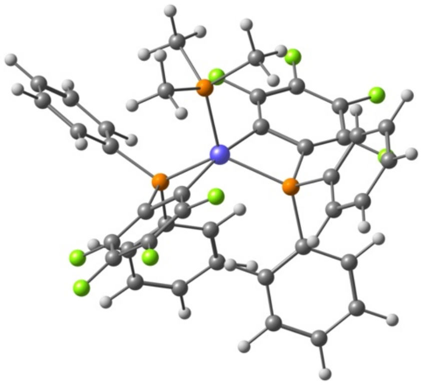

Figure S89. Optimized molecular structure of **3Ni**[a].

PBE0:

final single point energy: -4858.074430736005 a.u.

final Gibbs free energy: -4857.56855110 a.u.

Table S22. Atomic coordinates for optimized structure of **3Ni**[a].

Ni	2.928511000	5.186030000	6.690481000
P	2.245315000	3.279618000	7.591224000
P	3.563818000	6.831391000	8.058392000
F	-0.517565000	5.367709000	5.950743000
F	-2.128258000	7.193806000	7.046247000
F	-1.215250000	9.018660000	8.792668000
F	1.414636000	9.062828000	9.463036000
C	1.803325000	7.179379000	8.066504000
C	1.349768000	6.186902000	7.177822000
C	0.015451000	6.226458000	6.838357000
C	-0.840281000	7.174491000	7.380797000
C	-0.371917000	8.123821000	8.278585000
C	0.970288000	8.131119000	8.615929000
C	4.126326000	6.650502000	9.770529000
C	3.224373000	6.422070000	10.806162000
C	3.683156000	6.209320000	12.097769000
C	5.044837000	6.218357000	12.364524000
C	5.949073000	6.442391000	11.333931000
C	5.494140000	6.655792000	10.043007000
C	4.463724000	8.288130000	7.460113000
C	5.123506000	8.195740000	6.238751000
C	5.807835000	9.290580000	5.728914000
C	5.832866000	10.481320000	6.438427000
C	5.180909000	10.577050000	7.663339000
C	4.503208000	9.484172000	8.177014000
H	2.159945000	6.411876000	10.604491000
H	2.972807000	6.039660000	12.898191000
H	5.401976000	6.053366000	13.373952000
H	7.013415000	6.451466000	11.536524000
H	6.205351000	6.829513000	9.243523000
H	5.096039000	7.263220000	5.688511000
H	6.317551000	9.211383000	4.776245000
H	6.363358000	11.338407000	6.040817000
H	5.203222000	11.507029000	8.218856000

H	4.003169000	9.559011000	9.135127000
P	2.883431000	5.230295000	4.453467000
F	5.984056000	4.015336000	7.934217000
F	8.034740000	3.351654000	6.341197000
F	7.841825000	3.490277000	3.667083000
F	5.535441000	4.327122000	2.507245000
C	4.557131000	4.610520000	4.655153000
C	4.623365000	4.534971000	6.056640000
C	5.812262000	4.107838000	6.603270000
C	6.889087000	3.761018000	5.800946000
C	6.794917000	3.834060000	4.417217000
C	5.613467000	4.264424000	3.838661000
C	2.874264000	6.731531000	3.439912000
C	3.233434000	6.729785000	2.092017000
C	3.234867000	7.914524000	1.375143000
C	2.868926000	9.105086000	1.994112000
C	2.500495000	9.109911000	3.330727000
C	2.503700000	7.924670000	4.053063000
C	1.970153000	4.010016000	3.474993000
C	0.770574000	4.368176000	2.861803000
C	0.006308000	3.411502000	2.212456000
C	0.428811000	2.089168000	2.171725000
C	1.621615000	1.727341000	2.782737000
C	2.388194000	2.681421000	3.434283000
H	3.512884000	5.802513000	1.606567000
H	3.521051000	7.911776000	0.330121000
H	2.870153000	10.029622000	1.429008000
H	2.212954000	10.035516000	3.814655000
H	2.223642000	7.919670000	5.099863000
H	0.432506000	5.397602000	2.892211000
H	-0.923233000	3.700147000	1.736384000
H	-0.169673000	1.343038000	1.663081000
H	1.958808000	0.698045000	2.751444000
H	3.316046000	2.392069000	3.913942000
C	1.891995000	3.356554000	9.375209000
H	2.800972000	3.630947000	9.911145000
H	1.141276000	4.126628000	9.556757000
H	1.525496000	2.396405000	9.744719000
C	3.371546000	1.854065000	7.482474000
H	4.304943000	2.085409000	7.994943000
H	2.917676000	0.970475000	7.935551000
H	3.594079000	1.650687000	6.434360000
C	0.702288000	2.573881000	6.930387000
H	0.806482000	2.414610000	5.856670000
H	0.479186000	1.623849000	7.420651000
H	-0.118378000	3.269740000	7.095469000

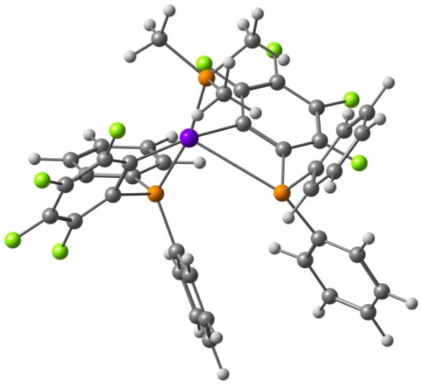

Figure S90. Optimized molecular structure of **3Pd**[a].

PBE0:

final single point energy: -8434.332064327524 a.u.
final Gibbs free energy: -8433.82673145 a.u.

Table S23. Atomic coordinates for optimized structure of **3Pd**[a].

Pd	2.463449000	4.961191000	7.584232000
P	1.511424000	2.891607000	7.327014000
P	3.203360000	7.044469000	8.281562000
F	-1.103055000	4.761883000	8.612237000
F	-2.349818000	6.694242000	9.947302000
F	-1.098273000	8.962851000	10.651574000
F	1.510773000	9.333861000	9.997712000
C	1.566785000	7.180885000	8.997829000
C	0.944868000	5.975845000	8.607061000
C	-0.384679000	5.850962000	8.944269000
C	-1.066451000	6.844298000	9.632198000
C	-0.425136000	8.019560000	9.998491000
C	0.905906000	8.191196000	9.668500000
C	4.500731000	7.091645000	9.530915000
C	4.270437000	7.514548000	10.836287000
C	5.303221000	7.492168000	11.762435000
C	6.566006000	7.053951000	11.389907000
C	6.798629000	6.630011000	10.087878000
C	5.768591000	6.640568000	9.162315000
C	3.458361000	8.478952000	7.209686000
C	2.463986000	8.778255000	6.277793000
C	2.611355000	9.862170000	5.430604000
C	3.755785000	10.647151000	5.495309000
C	4.749846000	10.346601000	6.413544000
C	4.603759000	9.265866000	7.272696000
H	3.288311000	7.857463000	11.135537000
H	5.118776000	7.818711000	12.778727000
H	7.369498000	7.037311000	12.116383000
H	7.780873000	6.279306000	9.795194000
H	5.945844000	6.291075000	8.151300000
H	1.577410000	8.158409000	6.216407000
H	1.835489000	10.089551000	4.709703000
H	3.873413000	11.489342000	4.824213000
H	5.644056000	10.955808000	6.467539000

H	5.379890000	9.043804000	7.994109000
P	3.088020000	5.613299000	4.544204000
F	4.993103000	3.273072000	8.639850000
F	7.305794000	2.630124000	7.450894000
F	7.746375000	3.259003000	4.860953000
F	5.879134000	4.537459000	3.445811000
C	4.433726000	4.673628000	5.337917000
C	4.190379000	4.340807000	6.681994000
C	5.168368000	3.640564000	7.354296000
C	6.369130000	3.284034000	6.767146000
C	6.600128000	3.612313000	5.442869000
C	5.625035000	4.291216000	4.735008000
C	3.938536000	6.662556000	3.312701000
C	4.838417000	7.611742000	3.799317000
C	5.471795000	8.490076000	2.936430000
C	5.196579000	8.451831000	1.575189000
C	4.286664000	7.525512000	1.086512000
C	3.662930000	6.633589000	1.948486000
C	2.234963000	4.374127000	3.513220000
C	0.955864000	4.708923000	3.064963000
C	0.211471000	3.813022000	2.313449000
C	0.727026000	2.557254000	2.020578000
C	1.993216000	2.210847000	2.470789000
C	2.745758000	3.115992000	3.205988000
H	5.043029000	7.665501000	4.863122000
H	6.173614000	9.215846000	3.330309000
H	5.686152000	9.143733000	0.900246000
H	4.064633000	7.489190000	0.026192000
H	2.966005000	5.905740000	1.551170000
H	0.537446000	5.677608000	3.317881000
H	-0.778505000	4.089129000	1.970305000
H	0.141098000	1.849143000	1.446960000
H	2.400229000	1.231751000	2.246931000
H	3.733711000	2.833164000	3.547643000
C	0.925954000	2.186667000	8.892480000
H	1.772454000	2.091503000	9.573141000
H	0.184524000	2.844947000	9.340167000
H	0.487908000	1.201714000	8.718190000
C	2.571559000	1.570953000	6.678415000
H	3.410651000	1.410756000	7.354675000
H	1.993948000	0.648748000	6.590010000
H	2.957198000	1.855635000	5.700806000
C	0.077256000	2.831838000	6.220027000
H	0.397892000	3.093012000	5.211546000
H	-0.347008000	1.825763000	6.216708000
H	-0.673193000	3.547617000	6.549227000

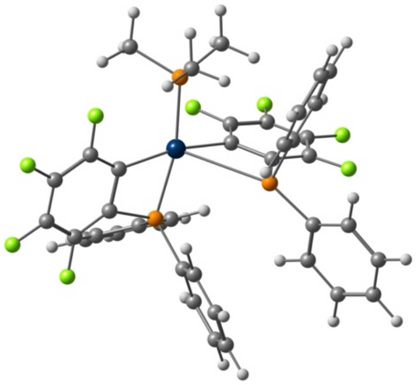

Figure S91. Optimized molecular structure of **3Pt**[a].

PBE0:

final single point energy: -22369.369143242449 a.u.
final Gibbs free energy: -22368.86329909 a.u.

Table S24. Atomic coordinates for optimized structure of **3Pt**[a].

Pt	2.450833000	4.972769000	7.592997000
P	1.434103000	2.950850000	7.334177000
P	3.229274000	7.031756000	8.297775000
F	-1.098805000	4.843764000	8.596653000
F	-2.321025000	6.776667000	9.942875000
F	-1.044226000	9.021075000	10.681849000
F	1.576079000	9.361157000	10.047126000
C	1.603516000	7.220296000	9.025092000
C	0.970019000	6.024431000	8.614056000
C	-0.365154000	5.916155000	8.943588000
C	-1.034114000	6.913722000	9.638540000
C	-0.380094000	8.075854000	10.022534000
C	0.955717000	8.232153000	9.701593000
C	4.546347000	7.037462000	9.525492000
C	4.356421000	7.499271000	10.823897000
C	5.402261000	7.448777000	11.734138000
C	6.636741000	6.943387000	11.351897000
C	6.828628000	6.481708000	10.056100000
C	5.785669000	6.521646000	9.145982000
C	3.529655000	8.447878000	7.214290000
C	2.547649000	8.769035000	6.276762000
C	2.727396000	9.845683000	5.426816000
C	3.891306000	10.601415000	5.495887000
C	4.872511000	10.279556000	6.420509000
C	4.693954000	9.205767000	7.282387000
H	3.396142000	7.894311000	11.129967000
H	5.250248000	7.805417000	12.745633000
H	7.450313000	6.904540000	12.066203000
H	7.788794000	6.079766000	9.756215000
H	5.930665000	6.146020000	8.139305000
H	1.646717000	8.170765000	6.213038000
H	1.961923000	10.090498000	4.700585000
H	4.033904000	11.437999000	4.822597000
H	5.781636000	10.865933000	6.477043000

H	5.460019000	8.965786000	8.008732000
P	3.108417000	5.649792000	4.553219000
F	4.936017000	3.179381000	8.608410000
F	7.212071000	2.469549000	7.408116000
F	7.674653000	3.113574000	4.823992000
F	5.852531000	4.480745000	3.431426000
C	4.420032000	4.650197000	5.329770000
C	4.159864000	4.310103000	6.672555000
C	5.117291000	3.561387000	7.328733000
C	6.301738000	3.167929000	6.733029000
C	6.544063000	3.503771000	5.413291000
C	5.593476000	4.229208000	4.718621000
C	3.982553000	6.653755000	3.301078000
C	4.917393000	7.578103000	3.769590000
C	5.568026000	8.429549000	2.892797000
C	5.275891000	8.389746000	1.535024000
C	4.331793000	7.488714000	1.064150000
C	3.690219000	6.623622000	1.940285000
C	2.181926000	4.446946000	3.543202000
C	0.911116000	4.837417000	3.117166000
C	0.113360000	3.973395000	2.383352000
C	0.566389000	2.694588000	2.086600000
C	1.824121000	2.293197000	2.514499000
C	2.630228000	3.166351000	3.231446000
H	5.135165000	7.633770000	4.830780000
H	6.296664000	9.136105000	3.272822000
H	5.778765000	9.061030000	0.849132000
H	4.096293000	7.451502000	0.006745000
H	2.965442000	5.915695000	1.556955000
H	0.541731000	5.824756000	3.374402000
H	-0.869649000	4.292262000	2.057662000
H	-0.061560000	2.011352000	1.527458000
H	2.182358000	1.295948000	2.287372000
H	3.611124000	2.841427000	3.556030000
C	0.835789000	2.256522000	8.897780000
H	1.684911000	2.128525000	9.569630000
H	0.119160000	2.933304000	9.357410000
H	0.365507000	1.287810000	8.718134000
C	2.462326000	1.606904000	6.684571000
H	3.292828000	1.419343000	7.364177000
H	1.858438000	0.702553000	6.590609000
H	2.859170000	1.883555000	5.709236000
C	0.004660000	2.927749000	6.221866000
H	0.340834000	3.164535000	5.212528000
H	-0.451656000	1.936086000	6.228822000
H	-0.724107000	3.671442000	6.536371000

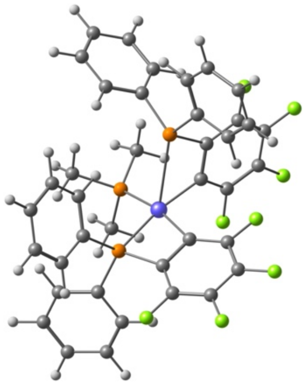

Figure S92. Optimized molecular structure of **3Ni[b]**.

PBE0:

final single point energy: -4858.074639166308 a.u.

final Gibbs free energy: -4857.56891465 a.u.

Table S25. Atomic coordinates for optimized structure of **3Ni[b]**.

Ni	-0.477648000	-0.041455000	0.835669000
P	-2.389875000	-0.346642000	-0.235719000
P	1.959650000	-0.515273000	-0.726537000
P	-0.418571000	-1.881791000	2.003838000
F	-4.051304000	2.044781000	-1.929452000
F	-2.981820000	4.536663000	-2.172812000
F	-0.620025000	5.099916000	-1.038062000
F	0.737782000	3.297882000	0.358639000
F	4.629474000	0.730252000	0.516217000
F	4.795713000	2.069444000	2.836243000
F	2.599420000	2.421291000	4.363205000
F	0.211763000	1.472491000	3.581991000
C	-0.928476000	1.632252000	-0.026819000
C	-2.167588000	1.377620000	-0.643744000
C	-2.875609000	2.320259000	-1.361751000
C	-2.343799000	3.588583000	-1.491183000
C	-1.119930000	3.875471000	-0.898066000
C	-0.431993000	2.912258000	-0.175298000
C	1.137343000	0.590769000	1.588494000
C	2.291203000	0.390537000	0.813361000
C	3.512750000	0.896071000	1.234233000
C	3.621826000	1.586066000	2.427003000
C	2.492724000	1.762754000	3.210965000
C	1.277599000	1.260157000	2.783271000
C	1.058647000	-2.268349000	2.982890000
H	1.267808000	-1.447495000	3.668211000
H	1.914653000	-2.395112000	2.321331000
H	0.897638000	-3.186971000	3.549606000
C	-0.706290000	-3.433182000	1.103003000
H	-0.718644000	-4.276425000	1.795869000
H	0.092264000	-3.574785000	0.373644000
H	-1.657643000	-3.384770000	0.573304000
C	-1.728136000	-1.861355000	3.264444000

H	-1.704286000	-2.782188000	3.850344000
H	-2.702305000	-1.757195000	2.787674000
H	-1.569609000	-1.007288000	3.923583000
C	-3.955723000	-0.567544000	0.639857000
C	-4.570035000	-1.817848000	0.686754000
H	-4.162892000	-2.645795000	0.118300000
C	-5.706140000	-2.005754000	1.458591000
H	-6.180434000	-2.979322000	1.487307000
C	-6.234568000	-0.950705000	2.189905000
H	-7.123496000	-1.098841000	2.791060000
C	-5.623259000	0.295103000	2.148125000
H	-6.033347000	1.121856000	2.715447000
C	-4.485853000	0.487305000	1.379555000
H	-4.007374000	1.459464000	1.355234000
C	-2.522907000	-1.327165000	-1.748331000
C	-1.391852000	-2.009324000	-2.186098000
H	-0.479576000	-1.949866000	-1.603959000
C	-1.431433000	-2.740668000	-3.363906000
H	-0.547572000	-3.269922000	-3.699776000
C	-2.601914000	-2.792598000	-4.106375000
H	-2.635671000	-3.364799000	-5.025821000
C	-3.733326000	-2.110513000	-3.673502000
H	-4.646090000	-2.148727000	-4.255741000
C	-3.697615000	-1.378877000	-2.498019000
H	-4.581287000	-0.848878000	-2.163382000
C	2.968885000	0.350647000	-1.979534000
C	3.845556000	-0.302670000	-2.841196000
H	4.024464000	-1.365278000	-2.730996000
C	4.506868000	0.402156000	-3.838058000
H	5.191034000	-0.119304000	-4.497442000
C	4.303297000	1.766255000	-3.985089000
H	4.823718000	2.315248000	-4.760668000
C	3.427903000	2.424469000	-3.130066000
H	3.262783000	3.490418000	-3.234994000
C	2.759455000	1.721790000	-2.141170000
H	2.078130000	2.245492000	-1.480566000
C	2.804873000	-2.121620000	-0.541506000
C	2.416407000	-3.134370000	-1.420958000
H	1.674709000	-2.925752000	-2.184848000
C	2.966389000	-4.403709000	-1.330405000
H	2.656677000	-5.175852000	-2.024649000
C	3.899771000	-4.685827000	-0.342259000
H	4.322659000	-5.679941000	-0.260595000
C	4.284816000	-3.689769000	0.544073000
H	5.011262000	-3.903477000	1.319265000
C	3.745624000	-2.415262000	0.442638000
H	4.058130000	-1.651107000	1.142254000

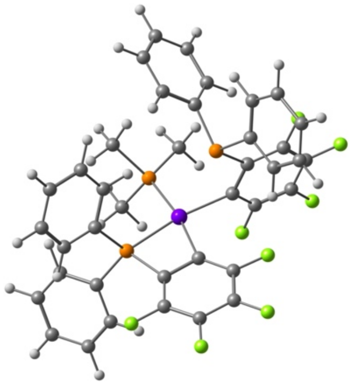

Figure S93. Optimized molecular structure of **3Pd**[b].

PBE0:

final single point energy: -8434.332553084003 a.u.

final Gibbs free energy: -8433.82723624 a.u.

Table S26. Atomic coordinates for optimized structure of **3Pd**[b].

Pd	0.480154000	-0.282478000	-0.794398000
P	2.512847000	-0.204538000	0.327317000
P	-2.118787000	0.005360000	0.995665000
P	0.311562000	-2.509271000	-1.326461000
F	4.083700000	2.542834000	1.427232000
F	2.949577000	4.998557000	1.154479000
F	0.572418000	5.258136000	-0.055187000
F	-0.743180000	3.155363000	-1.033109000
F	-4.809773000	0.564939000	-0.579330000
F	-5.063255000	0.961586000	-3.208047000
F	-2.908760000	0.782943000	-4.828874000
F	-0.490356000	0.203090000	-3.830944000
C	0.975850000	1.670027000	-0.323667000
C	2.223760000	1.567710000	0.316426000
C	2.901834000	2.660936000	0.819717000
C	2.335170000	3.914626000	0.687258000
C	1.103108000	4.044141000	0.057698000
C	0.439847000	2.933169000	-0.441649000
C	-1.336104000	0.130820000	-1.619363000
C	-2.458581000	0.233209000	-0.780840000
C	-3.705022000	0.503559000	-1.330018000
C	-3.863331000	0.698124000	-2.689819000
C	-2.759951000	0.599264000	-3.518849000
C	-1.523604000	0.307281000	-2.971345000
C	-1.103087000	-3.058794000	-2.317518000
H	-1.099834000	-2.533830000	-3.272606000
H	-2.029280000	-2.826210000	-1.794611000
H	-1.040796000	-4.134405000	-2.492158000
C	0.302681000	-3.645603000	0.087900000
H	0.282622000	-4.680364000	-0.259340000
H	-0.574854000	-3.451277000	0.705276000
H	1.196303000	-3.478632000	0.689562000
C	1.731847000	-3.061912000	-2.312899000
H	1.642574000	-4.124419000	-2.547617000

H	2.653443000	-2.886550000	-1.758269000
H	1.769479000	-2.486580000	-3.238319000
C	4.113082000	-0.572755000	-0.421509000
C	4.700742000	-1.821050000	-0.218001000
H	4.236991000	-2.538345000	0.449639000
C	5.883591000	-2.145591000	-0.862353000
H	6.337480000	-3.115012000	-0.695563000
C	6.484978000	-1.231126000	-1.717170000
H	7.410789000	-1.485630000	-2.218782000
C	5.899227000	0.009389000	-1.926678000
H	6.365995000	0.726406000	-2.591154000
C	4.715368000	0.339072000	-1.284136000
H	4.260174000	1.307758000	-1.453498000
C	2.612234000	-0.764725000	2.043633000
C	1.445464000	-1.233962000	2.641496000
H	0.530478000	-1.278615000	2.061227000
C	1.454318000	-1.627397000	3.971129000
H	0.543762000	-1.992205000	4.431210000
C	2.629075000	-1.557334000	4.705916000
H	2.638338000	-1.868277000	5.743783000
C	3.795162000	-1.088603000	4.112776000
H	4.711500000	-1.030639000	4.687733000
C	3.790105000	-0.691596000	2.785443000
H	4.700589000	-0.325082000	2.327048000
C	-3.324430000	1.098202000	1.828465000
C	-4.311538000	0.650538000	2.701192000
H	-4.450635000	-0.411356000	2.863589000
C	-5.132582000	1.557738000	3.357204000
H	-5.899788000	1.195097000	4.031546000
C	-4.982027000	2.920315000	3.144320000
H	-5.627573000	3.626305000	3.652941000
C	-3.996443000	3.374835000	2.276792000
H	-3.869294000	4.437464000	2.106064000
C	-3.165905000	2.471704000	1.633818000
H	-2.390970000	2.835736000	0.967502000
C	-2.746683000	-1.669307000	1.353538000
C	-2.350702000	-2.242453000	2.563946000
H	-1.743035000	-1.666780000	3.254338000
C	-2.719651000	-3.537837000	2.889955000
H	-2.406204000	-3.966015000	3.834727000
C	-3.474119000	-4.289646000	1.998445000
H	-3.751134000	-5.307875000	2.243833000
C	-3.869235000	-3.731387000	0.791178000
H	-4.459655000	-4.311477000	0.091674000
C	-3.514127000	-2.427658000	0.473816000
H	-3.832935000	-2.003668000	-0.470269000

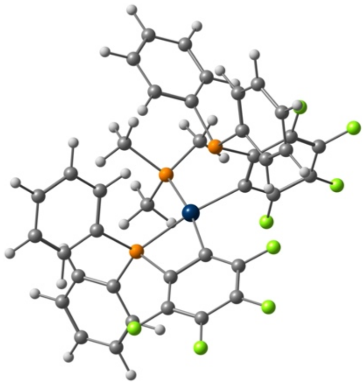

Figure S94. Optimized molecular structure of **3Pt^{b}**.

PBE0:

final single point energy: -22369.371677585885 a.u.

final Gibbs free energy: -22368.86586363 a.u.

Table S27. Atomic coordinates for optimized structure of **3Pt^{b}**.

Pt	0.485199000	-0.281834000	-0.781444000
P	2.496060000	-0.204391000	0.340583000
P	-2.120692000	0.089792000	1.001107000
P	0.334309000	-2.492164000	-1.328188000
F	4.098508000	2.556924000	1.447403000
F	2.951947000	5.007971000	1.168429000
F	0.570125000	5.250476000	-0.036044000
F	-0.740055000	3.141220000	-1.000415000
F	-4.810242000	0.619421000	-0.593377000
F	-5.055209000	0.973194000	-3.227430000
F	-2.895406000	0.750745000	-4.838318000
F	-0.491096000	0.168451000	-3.829642000
C	0.984995000	1.663727000	-0.288270000
C	2.237592000	1.572524000	0.350226000
C	2.913906000	2.667928000	0.844931000
C	2.340947000	3.919256000	0.708486000
C	1.106651000	4.040040000	0.082147000
C	0.443604000	2.925852000	-0.411148000
C	-1.331029000	0.145889000	-1.612590000
C	-2.459722000	0.276942000	-0.780560000
C	-3.702846000	0.541920000	-1.338286000
C	-3.857982000	0.712744000	-2.701659000
C	-2.753277000	0.591444000	-3.524577000
C	-1.520060000	0.299634000	-2.970226000
C	-1.077716000	-3.022996000	-2.331827000
H	-1.060052000	-2.497174000	-3.286236000
H	-2.006284000	-2.782825000	-1.816826000
H	-1.024093000	-4.098520000	-2.508119000
C	0.312328000	-3.643851000	0.072425000
H	0.286105000	-4.674049000	-0.287015000
H	-0.565423000	-3.450601000	0.689767000
H	1.206067000	-3.490693000	0.677542000
C	1.754608000	-3.042418000	-2.313266000
H	1.660505000	-4.102443000	-2.556133000

H	2.674938000	-2.876271000	-1.753966000
H	1.797523000	-2.459596000	-3.233605000
C	4.082794000	-0.601346000	-0.421748000
C	4.678803000	-1.840113000	-0.190253000
H	4.232317000	-2.536806000	0.509850000
C	5.848188000	-2.182137000	-0.850650000
H	6.308785000	-3.144623000	-0.663472000
C	6.427717000	-1.293411000	-1.746212000
H	7.343516000	-1.561016000	-2.259281000
C	5.833979000	-0.061080000	-1.981934000
H	6.284573000	0.635662000	-2.678319000
C	4.662875000	0.284969000	-1.325508000
H	4.199807000	1.246160000	-1.515497000
C	2.593931000	-0.795306000	2.045138000
C	1.436423000	-1.298681000	2.631975000
H	0.524421000	-1.350283000	2.048191000
C	1.452799000	-1.718381000	3.953593000
H	0.550619000	-2.113121000	4.405299000
C	2.624242000	-1.635062000	4.692045000
H	2.638601000	-1.964970000	5.723985000
C	3.780415000	-1.128035000	4.110870000
H	4.693823000	-1.059069000	4.689184000
C	3.768793000	-0.708563000	2.790781000
H	4.671903000	-0.314056000	2.340949000
C	-3.373728000	1.140749000	1.817926000
C	-4.358103000	0.660611000	2.676407000
H	-4.466670000	-0.405548000	2.834221000
C	-5.214989000	1.540312000	3.323995000
H	-5.979309000	1.152453000	3.987524000
C	-5.103623000	2.907496000	3.116505000
H	-5.777057000	3.591837000	3.618520000
C	-4.120823000	3.394641000	2.263534000
H	-4.023687000	4.461235000	2.097811000
C	-3.254146000	2.519220000	1.630145000
H	-2.479894000	2.908483000	0.977175000
C	-2.684173000	-1.603839000	1.374680000
C	-2.270174000	-2.150217000	2.591146000
H	-1.687480000	-1.544911000	3.277542000
C	-2.591946000	-3.455189000	2.928753000
H	-2.264471000	-3.862965000	3.877807000
C	-3.317651000	-4.242023000	2.043554000
H	-3.557451000	-5.267469000	2.297908000
C	-3.732825000	-3.709239000	0.831443000
H	-4.302101000	-4.316567000	0.137551000
C	-3.424352000	-2.396631000	0.502156000
H	-3.758407000	-1.991473000	-0.445068000

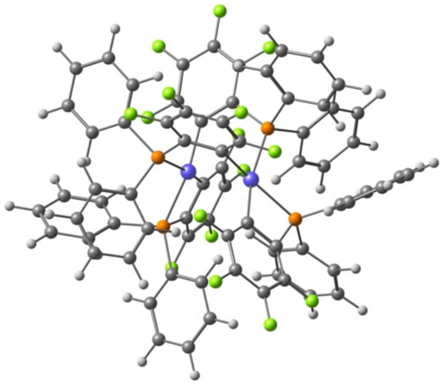

Figure S95. Optimized molecular structure of **4Ni**.

PBE0:

final single point energy: -8791.586316925803 a.u.

final Gibbs free energy: -8790.75110544 a.u.

Table S28. Atomic coordinates for optimized structure of **4Ni**.

Ni	1.325965000	0.856778000	-0.174347000
P	2.554617000	0.476852000	1.665068000
P	-1.897778000	0.853533000	-1.443791000
F	3.116181000	2.745055000	4.061198000
F	2.034036000	5.233650000	3.893429000
F	0.528205000	5.901280000	1.778691000
F	0.045419000	4.206081000	-0.192372000
F	-2.107692000	2.711603000	-3.855704000
F	-0.198226000	4.197213000	-4.968641000
F	2.296410000	4.208068000	-3.915032000
F	2.867889000	2.744903000	-1.772059000
C	1.267614000	2.464161000	0.896026000
C	2.067116000	2.162021000	2.012141000
C	2.348092000	3.062956000	3.019193000
C	1.811363000	4.333507000	2.940992000
C	1.030926000	4.672731000	1.843483000
C	0.781078000	3.757128000	0.831003000
C	0.704566000	1.780727000	-1.715766000
C	-0.579352000	1.775423000	-2.282161000
C	-0.873578000	2.619158000	-3.351588000
C	0.089085000	3.422208000	-3.924941000
C	1.360815000	3.429780000	-3.381146000
C	1.631712000	2.635851000	-2.286852000
C	2.346688000	-0.524821000	3.159589000
C	1.150891000	-1.208203000	3.355676000
H	0.384714000	-1.181739000	2.591665000
C	0.937444000	-1.926735000	4.520771000
H	0.001779000	-2.452076000	4.666008000
C	1.923505000	-1.971882000	5.495618000
H	1.761102000	-2.538215000	6.404784000
C	3.119483000	-1.291530000	5.305694000
H	3.889727000	-1.322659000	6.066784000
C	3.332439000	-0.566913000	4.143856000
H	4.264757000	-0.034229000	4.004586000

C	4.330633000	0.500386000	1.309122000
C	5.083251000	-0.672133000	1.355216000
H	4.636244000	-1.595010000	1.701987000
C	6.407801000	-0.663803000	0.947380000
H	6.983034000	-1.581013000	0.983726000
C	6.990949000	0.509760000	0.489018000
H	8.025291000	0.512158000	0.166901000
C	6.245232000	1.679407000	0.443097000
H	6.693819000	2.598218000	0.085445000
C	4.919432000	1.676183000	0.846822000
H	4.338201000	2.588174000	0.790264000
C	-3.015826000	0.205444000	-2.717125000
C	-2.534587000	-0.163984000	-3.970086000
H	-1.499262000	0.021919000	-4.230628000
C	-3.372025000	-0.778438000	-4.886847000
H	-2.986502000	-1.061490000	-5.858934000
C	-4.696079000	-1.036421000	-4.558559000
H	-5.349298000	-1.517906000	-5.276339000
C	-5.177955000	-0.680852000	-3.306905000
H	-6.206934000	-0.887296000	-3.037977000
C	-4.340955000	-0.069561000	-2.387154000
H	-4.723945000	0.183916000	-1.406416000
C	-2.825249000	2.211183000	-0.653532000
C	-3.924393000	2.843946000	-1.228803000
H	-4.299666000	2.522088000	-2.190588000
C	-4.549209000	3.888787000	-0.564855000
H	-5.410563000	4.367735000	-1.014900000
C	-4.077595000	4.321645000	0.667702000
H	-4.573207000	5.135667000	1.182948000
C	-2.967571000	3.711262000	1.233324000
H	-2.582452000	4.044521000	2.189848000
C	-2.350269000	2.662519000	0.572753000
H	-1.477939000	2.193504000	1.004610000
Ni	-1.325917000	-0.856921000	-0.174344000
P	-2.554633000	-0.476914000	1.665015000
P	1.897875000	-0.853689000	-1.443752000
F	-3.116196000	-2.744990000	4.061270000
F	-2.033979000	-5.233561000	3.893686000
F	-0.528074000	-5.901281000	1.779026000
F	-0.045265000	-4.206192000	-0.192116000
F	2.107908000	-2.712146000	-3.855347000
F	0.198496000	-4.197843000	-4.968227000
F	-2.296189000	-4.208565000	-3.914749000
F	-2.867746000	-2.745226000	-1.771928000
C	-1.267587000	-2.464251000	0.896109000
C	-2.067089000	-2.162048000	2.012207000
C	-2.348070000	-3.062931000	3.019305000
C	-1.811313000	-4.333475000	2.941193000
C	-1.030849000	-4.672750000	1.843720000
C	-0.781011000	-3.757207000	0.831183000
C	-0.704466000	-1.780952000	-1.715695000

C	0.579472000	-1.775676000	-2.282059000
C	0.873753000	-2.619560000	-3.351359000
C	-0.088877000	-3.422684000	-3.924658000
C	-1.360629000	-3.430195000	-3.380923000
C	-1.631575000	-2.636157000	-2.286724000
C	-2.346801000	0.524949000	3.159416000
C	-1.151186000	1.208694000	3.355319000
H	-0.385076000	1.182380000	2.591236000
C	-0.937853000	1.927427000	4.520314000
H	-0.002325000	2.453048000	4.665416000
C	-1.923858000	1.972426000	5.495224000
H	-1.761546000	2.538917000	6.404308000
C	-3.119677000	1.291748000	5.305459000
H	-3.889886000	1.322784000	6.066589000
C	-3.332519000	0.566936000	4.143723000
H	-4.264719000	0.034017000	4.004560000
C	-4.330651000	-0.500535000	1.309088000
C	-5.083439000	0.671841000	1.355919000
H	-4.636544000	1.594577000	1.703200000
C	-6.408026000	0.663551000	0.948221000
H	-6.983385000	1.580661000	0.985160000
C	-6.991048000	-0.509840000	0.489252000
H	-8.025436000	-0.512216000	0.167284000
C	-6.245154000	-1.679347000	0.442571000
H	-6.693644000	-2.598023000	0.084449000
C	-4.919313000	-1.676160000	0.846174000
H	-4.337950000	-2.588031000	0.789042000
C	3.015745000	-0.205419000	-2.717151000
C	2.534197000	0.164948000	-3.969717000
H	1.498714000	-0.020423000	-4.230007000
C	3.371530000	0.779671000	-4.886395000
H	2.985771000	1.063434000	-5.858181000
C	4.695781000	1.037019000	-4.558402000
H	5.348916000	1.518722000	-5.276113000
C	5.177949000	0.680583000	-3.307107000
H	6.207076000	0.886561000	-3.038384000
C	4.341054000	0.069021000	-2.387442000
H	4.724274000	-0.185114000	-1.406969000
C	2.825546000	-2.211290000	-0.653631000
C	3.924907000	-2.843702000	-1.228880000
H	4.300316000	-2.521490000	-2.190491000
C	4.549754000	-3.888656000	-0.565135000
H	5.411270000	-4.367331000	-1.015160000
C	4.077949000	-4.321991000	0.667184000
H	4.573536000	-5.136148000	1.182245000
C	2.967708000	-3.711969000	1.232763000
H	2.582420000	-4.045613000	2.189085000
C	2.350368000	-2.663125000	0.572388000
H	1.477813000	-2.194462000	1.004154000

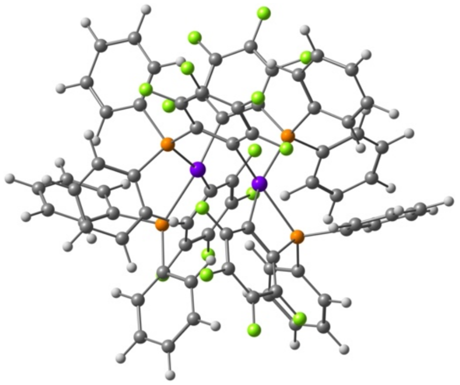

Figure S96. Optimized molecular structure of **4Pd**.

PBE0:

final single point energy: -15944.110250025693 a.u.
final Gibbs free energy: -15943.27759590 a.u.

Table S29. Atomic coordinates for optimized structure of **4Pd**.

Pd	1.331825000	0.874843000	-0.220743000
P	2.575281000	0.532380000	1.724569000
P	-1.942597000	0.924045000	-1.534106000
F	3.166700000	2.795529000	4.109938000
F	2.139047000	5.302756000	3.951868000
F	0.641003000	6.014347000	1.844467000
F	0.114982000	4.318667000	-0.137240000
F	-2.223535000	2.776476000	-3.944568000
F	-0.335144000	4.239141000	-5.130035000
F	2.189583000	4.241142000	-4.152543000
F	2.812970000	2.807797000	-2.005255000
C	1.312064000	2.571393000	0.951514000
C	2.105465000	2.234256000	2.061842000
C	2.404264000	3.129571000	3.069040000
C	1.893437000	4.412510000	2.995186000
C	1.116487000	4.774905000	1.903022000
C	0.845075000	3.866698000	0.888518000
C	0.644400000	1.858879000	-1.879154000
C	-0.654286000	1.853630000	-2.406650000
C	-0.975387000	2.685023000	-3.476557000
C	-0.023066000	3.474090000	-4.086799000
C	1.264120000	3.478090000	-3.580787000
C	1.562925000	2.698323000	-2.481384000
C	2.319496000	-0.475167000	3.203285000
C	1.137533000	-1.197298000	3.329445000
H	0.412246000	-1.183905000	2.525881000
C	0.887167000	-1.934148000	4.475627000
H	-0.037557000	-2.489809000	4.567252000
C	1.821921000	-1.956947000	5.500263000
H	1.630195000	-2.536258000	6.395454000
C	3.005289000	-1.239039000	5.378749000
H	3.735464000	-1.254907000	6.178790000
C	3.255986000	-0.497635000	4.235553000
H	4.178157000	0.063124000	4.147015000

C	4.351888000	0.524612000	1.386256000
C	5.079407000	-0.662207000	1.453661000
H	4.611461000	-1.570039000	1.812681000
C	6.404870000	-0.687198000	1.048855000
H	6.961437000	-1.615086000	1.101070000
C	7.012505000	0.466839000	0.573378000
H	8.047457000	0.443352000	0.254157000
C	6.290523000	1.650619000	0.505780000
H	6.758706000	2.554028000	0.134234000
C	4.964066000	1.680962000	0.905476000
H	4.400261000	2.602997000	0.831688000
C	-3.088032000	0.249417000	-2.764011000
C	-2.640633000	-0.129906000	-4.026217000
H	-1.621068000	0.079661000	-4.327530000
C	-3.492913000	-0.786118000	-4.899840000
H	-3.134957000	-1.077140000	-5.880089000
C	-4.796098000	-1.074691000	-4.518692000
H	-5.460378000	-1.588646000	-5.202972000
C	-5.242740000	-0.709623000	-3.256317000
H	-6.254713000	-0.941024000	-2.946200000
C	-4.391693000	-0.057906000	-2.378923000
H	-4.743967000	0.200905000	-1.387708000
C	-2.854883000	2.254953000	-0.690839000
C	-3.973354000	2.887639000	-1.226893000
H	-4.369655000	2.579234000	-2.185019000
C	-4.589994000	3.913825000	-0.526871000
H	-5.467167000	4.393286000	-0.944608000
C	-4.089393000	4.327048000	0.700852000
H	-4.577947000	5.127042000	1.244081000
C	-2.961110000	3.714557000	1.227648000
H	-2.556691000	4.031811000	2.181633000
C	-2.352807000	2.682347000	0.533799000
H	-1.470520000	2.205208000	0.936998000
Pd	-1.331789000	-0.874823000	-0.220718000
P	-2.575290000	-0.532357000	1.724568000
P	1.942623000	-0.924040000	-1.534109000
F	-3.166606000	-2.795446000	4.110015000
F	-2.138824000	-5.302628000	3.952040000
F	-0.640701000	-6.014207000	1.844692000
F	-0.114704000	-4.318552000	-0.137046000
F	2.223563000	-2.776582000	-3.944492000
F	0.335180000	-4.239336000	-5.129860000
F	-2.189527000	-4.241341000	-4.152322000
F	-2.812896000	-2.807916000	-2.005087000
C	-1.311978000	-2.571336000	0.951587000
C	-2.105381000	-2.234192000	2.061909000
C	-2.404149000	-3.129489000	3.069133000
C	-1.893259000	-4.412406000	2.995325000
C	-1.116276000	-4.774797000	1.903184000
C	-0.844904000	-3.866612000	0.888649000
C	-0.644367000	-1.858889000	-1.879113000

C	0.654314000	-1.853653000	-2.406622000
C	0.975415000	-2.685101000	-3.476487000
C	0.023100000	-3.474218000	-4.086674000
C	-1.264075000	-3.478222000	-3.580637000
C	-1.562878000	-2.698399000	-2.481274000
C	-2.319609000	0.475252000	3.203257000
C	-1.137646000	1.197369000	3.329485000
H	-0.412292000	1.183926000	2.525983000
C	-0.887360000	1.934254000	4.475663000
H	0.037367000	2.489896000	4.567348000
C	-1.822197000	1.957105000	5.500222000
H	-1.630531000	2.536438000	6.395412000
C	-3.005571000	1.239220000	5.378634000
H	-3.735813000	1.255132000	6.178613000
C	-3.256188000	0.497782000	4.235443000
H	-4.178364000	-0.062960000	4.146845000
C	-4.351898000	-0.524758000	1.386246000
C	-5.079581000	0.661946000	1.453916000
H	-4.611758000	1.569762000	1.813133000
C	-6.405053000	0.686839000	1.049136000
H	-6.961748000	1.614639000	1.101560000
C	-7.012536000	-0.467179000	0.573420000
H	-8.047496000	-0.443766000	0.254219000
C	-6.290393000	-1.650846000	0.505566000
H	-6.758458000	-2.554242000	0.133836000
C	-4.963928000	-1.681094000	0.905243000
H	-4.400001000	-2.603037000	0.831261000
C	3.088016000	-0.249390000	-2.764043000
C	2.640565000	0.129980000	-4.026216000
H	1.620990000	-0.079584000	-4.327497000
C	3.492803000	0.786238000	-4.899845000
H	3.134808000	1.077292000	-5.880071000
C	4.795997000	1.074820000	-4.518732000
H	5.460243000	1.588813000	-5.203016000
C	5.242686000	0.709720000	-3.256384000
H	6.254663000	0.941135000	-2.946288000
C	4.391681000	0.057954000	-2.378984000
H	4.743991000	-0.200876000	-1.387787000
C	2.854952000	-2.254920000	-0.690844000
C	3.973538000	-2.887462000	-1.226830000
H	4.369907000	-2.578949000	-2.184893000
C	4.590206000	-3.913637000	-0.526817000
H	5.467468000	-4.392983000	-0.944499000
C	4.089522000	-4.326993000	0.700827000
H	4.578099000	-5.126978000	1.244050000
C	2.961122000	-3.714655000	1.227549000
H	2.556635000	-4.032018000	2.181469000
C	2.352788000	-2.682456000	0.533709000
H	1.470401000	-2.205445000	0.936842000

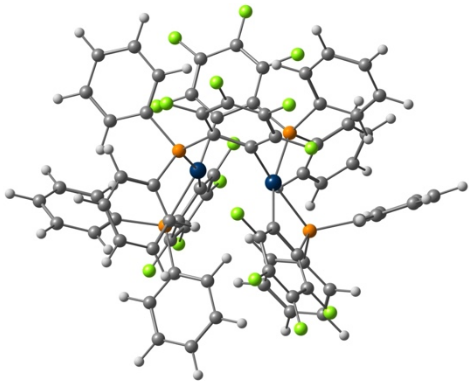

Figure S97. Optimized molecular structure of **4Pt**.

PBE0:

final single point energy: -43814.185929896616 a.u.
final Gibbs free energy: -43813.35307498 a.u.

Table S30. Atomic coordinates for optimized structure of **4Pt**.

Pt	0.586306000	-1.503440000	-0.234190000
Pt	-0.586294000	1.503614000	-0.234222000
P	0.012881000	-2.640287000	1.695654000
P	1.290622000	1.731179000	-1.523714000
F	2.138536000	-3.781794000	4.052186000
F	4.804122000	-3.303756000	3.851405000
F	5.770829000	-1.922995000	1.766042000
F	4.192141000	-0.999423000	-0.158448000
F	3.208648000	1.674994000	-3.864988000
F	4.330031000	-0.432507000	-5.050389000
F	3.804545000	-2.928633000	-4.146721000
F	2.211510000	-3.312195000	-2.068478000
C	2.248498000	-1.833141000	0.940286000
C	1.775080000	-2.565182000	2.046947000
C	2.604193000	-3.074955000	3.022803000
C	3.965631000	-2.845417000	2.927131000
C	4.461029000	-2.129809000	1.846130000
C	3.613316000	-1.644412000	0.859332000
C	1.698261000	-1.000579000	-1.885203000
C	1.959989000	0.284371000	-2.387544000
C	2.867167000	0.458633000	-3.429691000
C	3.480081000	-0.612587000	-4.042678000
C	3.214633000	-1.885386000	-3.573468000
C	2.357106000	-2.051940000	-2.504755000
C	-0.921696000	-2.146455000	3.160020000
C	-1.383801000	-0.838932000	3.259362000
H	-1.220675000	-0.150702000	2.439763000
C	-2.050650000	-0.418200000	4.398334000
H	-2.404800000	0.602446000	4.469006000
C	-2.260023000	-1.305701000	5.443621000
H	-2.784407000	-0.979653000	6.333805000
C	-1.798821000	-2.613069000	5.350437000
H	-1.960047000	-3.306097000	6.167192000
C	-1.129720000	-3.035611000	4.213347000

H	-0.769769000	-4.054808000	4.145797000
C	-0.406961000	-4.368447000	1.368706000
C	-1.719591000	-4.820158000	1.481551000
H	-2.491394000	-4.166066000	1.866404000
C	-2.045818000	-6.108281000	1.086052000
H	-3.070579000	-6.449012000	1.170737000
C	-1.067940000	-6.952427000	0.578960000
H	-1.326633000	-7.957592000	0.268595000
C	0.242065000	-6.505634000	0.467585000
H	1.009014000	-7.159271000	0.070295000
C	0.572427000	-5.217146000	0.854542000
H	1.591044000	-4.865453000	0.743558000
C	0.867507000	2.970619000	-2.774794000
C	0.410342000	2.587213000	-4.031964000
H	0.411318000	1.541232000	-4.315451000
C	-0.056017000	3.539847000	-4.924023000
H	-0.410219000	3.231750000	-5.900400000
C	-0.073876000	4.880876000	-4.566425000
H	-0.438716000	5.623919000	-5.265344000
C	0.370339000	5.266150000	-3.309080000
H	0.350151000	6.309586000	-3.018048000
C	0.831203000	4.315593000	-2.413118000
H	1.152581000	4.624463000	-1.425886000
C	2.775553000	2.356361000	-0.678314000
C	3.615288000	3.336821000	-1.198992000
H	3.386455000	3.807484000	-2.145475000
C	4.749906000	3.718995000	-0.498942000
H	5.392522000	4.491203000	-0.904557000
C	5.064446000	3.117890000	0.712695000
H	5.951283000	3.422726000	1.255259000
C	4.242322000	2.123418000	1.223535000
H	4.480509000	1.640494000	2.163931000
C	3.104360000	1.749959000	0.529349000
H	2.465054000	0.969460000	0.916822000
P	-0.013431000	2.640342000	1.695905000
P	-1.290142000	-1.731053000	-1.524212000
F	-2.139749000	3.781455000	4.052056000
F	-4.805254000	3.303251000	3.850559000
F	-5.771376000	1.922851000	1.764680000
F	-4.192148000	0.999765000	-0.159616000
F	-3.206965000	-1.674873000	-3.866498000
F	-4.328079000	0.432620000	-5.052178000
F	-3.803505000	2.928690000	-4.147849000
F	-2.211497000	3.312226000	-2.068814000
C	-2.248833000	1.833312000	0.939806000
C	-1.775731000	2.565224000	2.046688000
C	-2.605112000	3.074765000	3.022434000
C	-3.966512000	2.845146000	2.926396000
C	-4.461605000	2.129719000	1.845135000
C	-3.613621000	1.644555000	0.858457000
C	-1.697864000	1.000685000	-1.885677000

C	-1.959273000	-0.284254000	-2.388233000
C	-2.865911000	-0.458517000	-3.430845000
C	-3.478677000	0.612691000	-4.044001000
C	-3.213692000	1.885461000	-3.574461000
C	-2.356667000	2.052010000	-2.505342000
C	0.920679000	2.146365000	3.160512000
C	1.382349000	0.838705000	3.260049000
H	1.219156000	0.150467000	2.440470000
C	2.048833000	0.417847000	4.399188000
H	2.402646000	-0.602905000	4.470007000
C	2.258270000	1.305364000	5.444450000
H	2.782368000	0.979222000	6.334767000
C	1.797487000	2.612866000	5.351077000
H	1.958757000	3.305903000	6.167816000
C	1.128748000	3.035532000	4.213820000
H	0.769124000	4.054835000	4.146120000
C	0.406531000	4.368515000	1.369205000
C	1.719108000	4.820232000	1.482605000
H	2.490760000	4.166130000	1.867746000
C	2.045488000	6.108373000	1.087285000
H	3.070213000	6.449102000	1.172406000
C	1.067814000	6.952524000	0.579812000
H	1.326626000	7.957700000	0.269580000
C	-0.242142000	6.505724000	0.467881000
H	-1.008931000	7.159367000	0.070293000
C	-0.572656000	5.217225000	0.854669000
H	-1.591225000	4.865525000	0.743261000
C	-0.866574000	-2.970443000	-2.775184000
C	-0.409138000	-2.586953000	-4.032232000
H	-0.410177000	-1.540965000	-4.315695000
C	0.057557000	-3.539510000	-4.924193000
H	0.411969000	-3.231353000	-5.900474000
C	0.075487000	-4.880547000	-4.566618000
H	0.440592000	-5.623530000	-5.265462000
C	-0.369020000	-5.265903000	-3.309402000
H	-0.348801000	-6.309343000	-3.018387000
C	-0.830242000	-4.315422000	-2.413540000
H	-1.151876000	-4.624358000	-1.426412000
C	-2.775265000	-2.356350000	-0.679250000
C	-3.614598000	-3.337085000	-1.200066000
H	-3.385298000	-3.807884000	-2.146369000
C	-4.749383000	-3.719382000	-0.500357000
H	-5.391684000	-4.491804000	-0.906065000
C	-5.064489000	-3.118137000	0.711066000
H	-5.951454000	-3.423076000	1.253362000
C	-4.242758000	-2.123413000	1.222041000
H	-4.481377000	-1.640383000	2.162274000
C	-3.104620000	-1.749838000	0.528205000
H	-2.465605000	-0.969151000	0.915781000

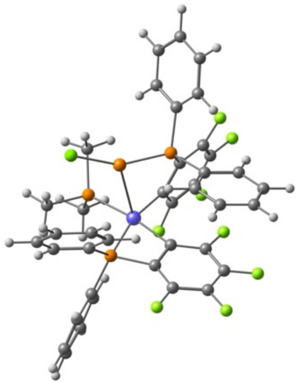

Figure S98. Optimized molecular structure of *cis*-**6NiCu**.

PBE0:

final single point energy: -6983.224029159665 a.u.

final Gibbs free energy: -6982.71750512 a.u.

Table S31. Atomic coordinates for optimized structure of *cis*-**6NiCu**.

Ni	-0.587572000	0.205481000	1.045031000
Cu	0.738703000	-1.904690000	-0.221706000
Cl	0.074189000	-3.927160000	-0.241388000
P	-2.482901000	-0.356015000	0.010522000
P	2.158520000	-0.287568000	-0.397129000
P	-0.466007000	-1.247758000	2.699455000
F	-3.702475000	1.229148000	-2.701400000
F	-2.545660000	3.536345000	-3.563101000
F	-0.456770000	4.565804000	-2.242900000
F	0.587689000	3.376235000	-0.116234000
F	4.529476000	1.391693000	0.785608000
F	4.398289000	3.114660000	2.822306000
F	2.038175000	3.593622000	4.047344000
F	-0.193815000	2.358173000	3.248172000
C	-0.948818000	1.555189000	-0.286378000
C	-2.067035000	1.067558000	-0.984648000
C	-2.630281000	1.709368000	-2.069234000
C	-2.061531000	2.889201000	-2.506862000
C	-0.973970000	3.415649000	-1.821028000
C	-0.439966000	2.765048000	-0.719768000
C	0.986894000	1.072334000	1.649739000
C	2.218734000	0.846390000	1.011953000
C	3.353591000	1.546544000	1.401289000
C	3.305231000	2.458888000	2.437608000
C	2.094746000	2.702583000	3.062880000
C	0.964152000	2.024287000	2.646403000
C	1.124652000	-2.062434000	3.023536000
H	1.061156000	-2.634268000	3.951046000
H	1.902245000	-1.303764000	3.113957000
H	1.375497000	-2.735749000	2.203210000
C	-1.657793000	-2.614838000	2.679858000
H	-1.525908000	-3.242126000	3.563149000
H	-1.509021000	-3.210941000	1.779642000
H	-2.667901000	-2.202291000	2.679592000

C	-0.808990000	-0.471531000	4.309527000
H	-1.771955000	0.036650000	4.288281000
H	-0.033773000	0.254463000	4.546264000
H	-0.825736000	-1.246296000	5.078348000
C	-4.042174000	0.023743000	0.851080000
C	-3.973382000	0.686340000	2.074057000
H	-3.004297000	0.917508000	2.502298000
C	-5.134525000	1.061342000	2.731966000
H	-5.073211000	1.576453000	3.682937000
C	-6.370775000	0.772182000	2.171611000
H	-7.279541000	1.060402000	2.686266000
C	-6.444229000	0.114629000	0.950245000
H	-7.408810000	-0.106988000	0.509634000
C	-5.285117000	-0.258562000	0.288368000
H	-5.347716000	-0.767743000	-0.665331000
C	-2.822386000	-1.774631000	-1.051291000
C	-3.496829000	-2.886746000	-0.552269000
H	-3.860997000	-2.891486000	0.467966000
C	-3.712243000	-3.988921000	-1.362583000
H	-4.240141000	-4.849119000	-0.969114000
C	-3.250233000	-3.993220000	-2.671861000
H	-3.418188000	-4.857507000	-3.302988000
C	-2.572280000	-2.890121000	-3.170232000
H	-2.210250000	-2.888108000	-4.191210000
C	-2.357697000	-1.783767000	-2.363418000
H	-1.831836000	-0.923157000	-2.758194000
C	1.947180000	0.733521000	-1.875973000
C	2.618281000	1.940457000	-2.053407000
H	3.303280000	2.303607000	-1.297762000
C	2.398674000	2.691630000	-3.196686000
H	2.914540000	3.635155000	-3.326624000
C	1.512955000	2.242677000	-4.168091000
H	1.336249000	2.838928000	-5.055230000
C	0.849338000	1.036002000	-3.999632000
H	0.154829000	0.684231000	-4.752915000
C	1.065130000	0.284575000	-2.855113000
H	0.538508000	-0.652067000	-2.710120000
C	3.801637000	-1.046734000	-0.559651000
C	4.307138000	-1.763209000	0.524877000
H	3.750011000	-1.806691000	1.453995000
C	5.523660000	-2.415310000	0.423066000
H	5.913335000	-2.962085000	1.273302000
C	6.238728000	-2.373896000	-0.767987000
H	7.188635000	-2.888463000	-0.848571000
C	5.731503000	-1.675597000	-1.853153000
H	6.283543000	-1.642081000	-2.784799000
C	4.516180000	-1.012031000	-1.751868000
H	4.129533000	-0.464650000	-2.602403000

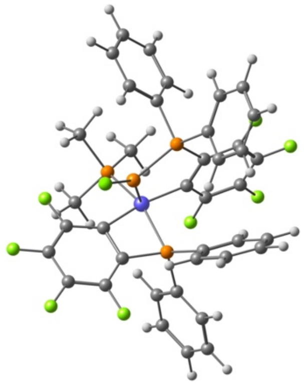

Figure S99. Optimized molecular structure of *trans*-**6NiCu**.

PBE0:

final single point energy: -6983.223759100865 a.u.

final Gibbs free energy: -6982.71770783 a.u.

Table S32. Atomic coordinates for optimized structure of *trans*-**6NiCu**.

Ni	0.431743000	-0.271271000	-1.065900000
Cu	-0.118035000	-1.323548000	1.170673000
Cl	0.493546000	-2.561208000	2.851731000
P	1.741137000	1.059999000	0.075951000
P	-1.975742000	-0.257490000	0.657739000
P	-0.231064000	-1.575289000	-2.687982000
F	4.514548000	0.148931000	1.748679000
F	5.304940000	-2.449802000	1.758422000
F	3.870049000	-4.299883000	0.457195000
F	1.653536000	-3.680808000	-0.858758000
F	-3.941169000	2.175082000	0.197741000
F	-3.674133000	4.100768000	-1.638361000
F	-1.603307000	4.049516000	-3.371574000
F	0.224783000	2.096952000	-3.268908000
C	1.827995000	-1.385727000	-0.248193000
C	2.611071000	-0.447336000	0.465312000
C	3.781708000	-0.767638000	1.117944000
C	4.200307000	-2.084987000	1.121908000
C	3.454002000	-3.039682000	0.442608000
C	2.298201000	-2.686616000	-0.231813000
C	-0.849180000	1.078341000	-1.422410000
C	-1.934607000	1.117495000	-0.528208000
C	-2.880890000	2.129676000	-0.611185000
C	-2.768408000	3.127655000	-1.561293000
C	-1.708968000	3.095480000	-2.452448000
C	-0.779221000	2.073720000	-2.372400000
C	-1.351923000	-0.843388000	-3.913865000
H	-0.884327000	0.031497000	-4.363929000
H	-2.276873000	-0.536831000	-3.426702000
H	-1.576097000	-1.575842000	-4.691425000
C	-1.071173000	-3.138883000	-2.307833000
H	-1.126443000	-3.748664000	-3.211738000
H	-2.080226000	-2.920046000	-1.961863000
H	-0.541766000	-3.680440000	-1.529067000

C	1.195694000	-2.053644000	-3.706387000
H	0.863665000	-2.642642000	-4.563662000
H	1.898766000	-2.635849000	-3.114028000
H	1.695752000	-1.150759000	-4.058787000
C	1.253374000	1.985727000	1.537735000
C	0.405318000	3.080320000	1.362581000
H	0.063636000	3.350086000	0.369574000
C	0.002579000	3.824868000	2.458217000
H	-0.656454000	4.672976000	2.318805000
C	0.437647000	3.479900000	3.731455000
H	0.119546000	4.062300000	4.587583000
C	1.267018000	2.382215000	3.909689000
H	1.597849000	2.104230000	4.902818000
C	1.674229000	1.631166000	2.816298000
H	2.312652000	0.769914000	2.964326000
C	2.870357000	2.136976000	-0.838917000
C	3.198966000	1.766970000	-2.142146000
H	2.744943000	0.884495000	-2.579500000
C	4.094351000	2.527495000	-2.874868000
H	4.346493000	2.236583000	-3.887430000
C	4.658338000	3.666671000	-2.314462000
H	5.353592000	4.265602000	-2.890373000
C	4.328988000	4.040521000	-1.019288000
H	4.768762000	4.928246000	-0.580993000
C	3.437931000	3.277789000	-0.278660000
H	3.187416000	3.570280000	0.733426000
C	-2.761373000	0.322609000	2.192886000
C	-4.122049000	0.168152000	2.445538000
H	-4.766005000	-0.283210000	1.701171000
C	-4.656530000	0.594865000	3.650713000
H	-5.715604000	0.469893000	3.842071000
C	-3.839292000	1.179631000	4.609634000
H	-4.260099000	1.509827000	5.551892000
C	-2.482172000	1.329980000	4.363406000
H	-1.836119000	1.776124000	5.109688000
C	-1.943421000	0.893377000	3.163371000
H	-0.879452000	0.985235000	2.983686000
C	-3.181973000	-1.430159000	-0.023445000
C	-3.249550000	-2.692734000	0.566781000
H	-2.599235000	-2.931984000	1.401816000
C	-4.131098000	-3.642781000	0.080533000
H	-4.175669000	-4.621373000	0.542876000
C	-4.943424000	-3.346113000	-1.007551000
H	-5.624106000	-4.094253000	-1.395498000
C	-4.878202000	-2.093061000	-1.598339000
H	-5.509227000	-1.857494000	-2.446832000
C	-4.003228000	-1.134282000	-1.105529000
H	-3.956460000	-0.159777000	-1.575241000

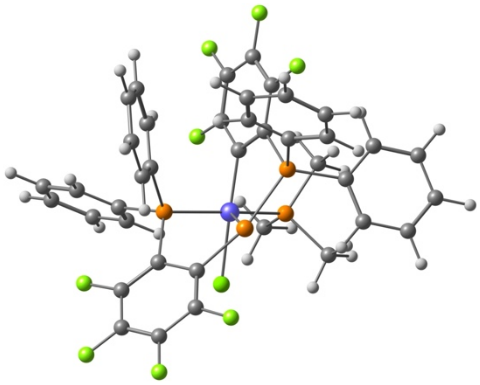

Figure S100. Optimized molecular structure of *cis*-*trans*-**7NiCu**.

PBE0:

final single point energy: -6983.224877324999 a.u.

final Gibbs free energy: -6982.71759667 a.u.

Table S33. Atomic coordinates for optimized structure of *cis*-*trans*-**7NiCu**.

Ni	-0.343417000	0.907879000	-1.291836000
Cu	0.124211000	-1.625898000	-0.312495000
P	2.080972000	-0.776349000	0.133296000
Cl	-1.714267000	0.124286000	-2.837308000
C	1.933185000	0.990315000	0.516316000
F	-1.360216000	-4.474985000	-0.828299000
C	3.353356000	-0.955651000	-1.140843000
C	-4.004117000	-1.453575000	-0.034730000
C	0.751821000	3.031248000	0.224094000
C	2.666030000	2.995333000	1.651007000
C	0.859118000	1.685283000	-0.071453000
C	2.823119000	1.656477000	1.352003000
C	-2.650442000	-1.177633000	-0.053843000
C	-2.195967000	-3.448637000	-0.567574000
C	-4.459387000	-2.736293000	-0.301659000
C	-1.708311000	-2.187787000	-0.331348000
F	3.517051000	3.617339000	2.462277000
F	-4.930749000	-0.528970000	0.233399000
F	-0.230242000	3.769869000	-0.323864000
F	3.880326000	1.041893000	1.888168000
F	-5.764271000	-3.002448000	-0.299028000
F	1.459501000	4.980650000	1.350113000
F	-3.994680000	-4.970954000	-0.827401000
C	1.616433000	3.690789000	1.077296000
P	-1.857726000	0.419069000	0.290545000
C	-1.377096000	0.256755000	2.044730000
C	-3.090704000	1.759043000	0.352890000
P	0.863816000	1.709103000	-2.950239000
C	2.774870000	-1.565097000	1.613823000
C	-3.551068000	-3.742537000	-0.568748000
C	-3.262725000	2.565622000	-0.769040000
H	-2.678912000	2.373847000	-1.660559000
C	-4.178968000	3.606637000	-0.748461000
H	-4.304366000	4.228691000	-1.626567000

C	-4.924823000	3.853656000	0.394420000
H	-5.636725000	4.670329000	0.412810000
C	-4.754464000	3.054929000	1.518131000
H	-5.334460000	3.244375000	2.413428000
C	-3.841683000	2.013600000	1.499280000
H	-3.715930000	1.395963000	2.379573000
C	-1.542562000	-0.931455000	2.752074000
H	-1.958417000	-1.800602000	2.261203000
C	-1.180315000	-1.009633000	4.090247000
H	-1.318618000	-1.940760000	4.626318000
C	-0.639998000	0.092377000	4.733954000
H	-0.351499000	0.028227000	5.776226000
C	-0.471711000	1.281588000	4.035515000
H	-0.056575000	2.151376000	4.530299000
C	-0.843322000	1.364721000	2.704265000
H	-0.736473000	2.306474000	2.183854000
C	3.171838000	-1.955600000	-2.093721000
H	2.275642000	-2.566325000	-2.065273000
C	4.125017000	-2.161862000	-3.080201000
H	3.976706000	-2.940740000	-3.818096000
C	5.256420000	-1.360572000	-3.127073000
H	5.996603000	-1.513325000	-3.903247000
C	5.440102000	-0.359624000	-2.181383000
C	4.496079000	-0.159889000	-1.187095000
C	3.878382000	-2.408767000	1.558323000
H	4.398983000	-2.566641000	0.621805000
C	4.320616000	-3.046013000	2.709845000
H	5.182970000	-3.700177000	2.662048000
C	3.666012000	-2.843962000	3.915890000
H	4.015765000	-3.340427000	4.813037000
C	2.559653000	-2.004745000	3.970521000
H	2.041295000	-1.843308000	4.908206000
C	2.110681000	-1.373676000	2.823764000
H	1.245225000	-0.723491000	2.869798000
H	6.322857000	0.267164000	-2.217611000
H	4.652821000	0.617715000	-0.450133000
C	2.418000000	2.581802000	-2.608988000
H	2.873533000	2.883943000	-3.553494000
H	2.224957000	3.466894000	-2.004285000
H	3.103242000	1.929393000	-2.070441000
C	1.357467000	0.503499000	-4.208585000
H	0.480656000	-0.046563000	-4.543784000
H	1.821378000	1.023039000	-5.048916000
H	2.074325000	-0.192900000	-3.774324000
C	-0.093339000	2.960904000	-3.848809000
H	0.499349000	3.357440000	-4.675240000
H	-1.008197000	2.508604000	-4.226820000
H	-0.350680000	3.770039000	-3.164838000

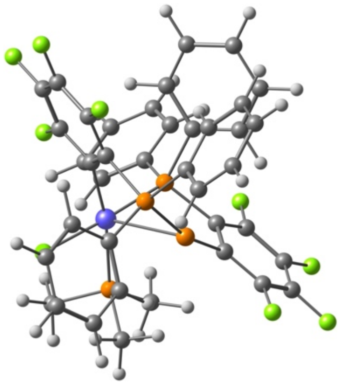

Figure S101. Optimized molecular structure of *cis-cis*-**7NiCu**.

PBE0:

final single point energy: -6983.219460393570 a.u.
final Gibbs free energy: -6982.71143922 a.u.

Table S34. Atomic coordinates for optimized structure of *cis-cis*-**7NiCu**.

Ni	0.380018000	0.413081000	-1.519232000
Cu	-0.331243000	-1.492068000	0.218896000
P	-2.294329000	-0.538781000	0.150744000
P	1.715376000	0.657582000	0.182698000
F	-4.188802000	1.836071000	0.794897000
F	-3.763997000	4.424338000	0.310163000
F	-1.497401000	5.220655000	-0.932541000
F	0.347130000	3.440569000	-1.678985000
F	4.646075000	-0.239090000	1.164966000
F	5.383534000	-2.723493000	1.746173000
F	3.618495000	-4.759097000	1.599741000
F	1.070942000	-4.305714000	0.897256000
C	-0.895542000	1.637624000	-0.783264000
C	-2.078348000	1.237454000	-0.138498000
C	-3.034897000	2.182354000	0.214925000
C	-2.845231000	3.526421000	-0.038476000
C	-1.688964000	3.929953000	-0.681802000
C	-0.751551000	2.982977000	-1.049489000
C	1.477369000	-1.994957000	0.599593000
C	2.420592000	-0.950021000	0.665302000
C	3.719971000	-1.195879000	1.072939000
C	4.126189000	-2.483369000	1.384847000
C	3.219471000	-3.524879000	1.309175000
C	1.913682000	-3.255284000	0.932300000
C	-3.442004000	-1.184900000	-1.090149000
C	-3.158918000	-2.434533000	-1.634865000
H	-2.265952000	-2.965351000	-1.322508000
C	-4.004320000	-2.992089000	-2.582300000
H	-3.776841000	-3.964304000	-3.002426000
C	-5.130665000	-2.297175000	-2.997513000
H	-5.787943000	-2.726632000	-3.744097000
C	-5.413688000	-1.047433000	-2.460702000
H	-6.290795000	-0.501921000	-2.787297000
C	-4.575667000	-0.492149000	-1.507436000

H	-4.807166000	0.481369000	-1.095860000
C	-3.157757000	-0.700878000	1.739551000
C	-4.180497000	-1.626048000	1.921081000
H	-4.538477000	-2.213362000	1.084209000
C	-4.750820000	-1.792188000	3.175705000
H	-5.550248000	-2.511196000	3.309384000
C	-4.304156000	-1.039473000	4.252024000
H	-4.753466000	-1.169253000	5.229263000
C	-3.280617000	-0.117070000	4.072505000
H	-2.926791000	0.476000000	4.907590000
C	-2.706598000	0.048495000	2.823968000
H	-1.909077000	0.768971000	2.689844000
C	3.025991000	1.863610000	-0.188551000
C	3.257504000	2.208234000	-1.515947000
H	2.646835000	1.771384000	-2.296301000
C	4.238429000	3.132155000	-1.844053000
H	4.406158000	3.393336000	-2.881783000
C	4.986426000	3.729210000	-0.841248000
H	5.749169000	4.456414000	-1.092767000
C	4.748393000	3.404225000	0.489060000
H	5.324994000	3.876238000	1.275356000
C	3.772270000	2.479168000	0.815702000
H	3.592529000	2.233373000	1.854476000
C	1.053382000	1.279681000	1.764925000
C	0.675496000	2.619149000	1.863651000
H	0.799685000	3.283107000	1.018818000
C	0.163991000	3.118365000	3.048816000
H	-0.128593000	4.159692000	3.108552000
C	0.038635000	2.290945000	4.158518000
H	-0.357890000	2.683796000	5.087007000
C	0.427131000	0.963995000	4.072689000
H	0.336134000	0.311777000	4.932722000
C	0.926138000	0.456892000	2.880033000
H	1.218694000	-0.582802000	2.825208000
Cl	-0.984362000	0.277360000	-3.244767000
P	1.727049000	-0.935330000	-2.723116000
C	3.403682000	-1.464191000	-2.245307000
H	4.004839000	-0.613629000	-1.924063000
H	3.357837000	-2.194290000	-1.440868000
H	3.874968000	-1.924282000	-3.115543000
C	0.924991000	-2.528262000	-3.058122000
H	1.541099000	-3.120156000	-3.737216000
H	0.806068000	-3.061079000	-2.113910000
H	-0.055453000	-2.352695000	-3.496411000
C	2.067821000	-0.238472000	-4.369150000
H	2.574542000	-0.988104000	-4.979303000
H	1.136935000	0.065151000	-4.841005000
H	2.718632000	0.631040000	-4.267830000

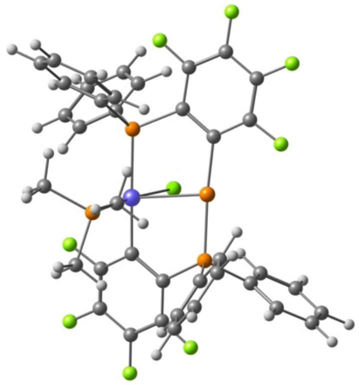

Figure S102. Optimized molecular structure of *trans*-**7NiCu**.

PBE0:

final single point energy: -6983.216677682291 a.u.
final Gibbs free energy: -6982.70997514 a.u.

Table S35. Atomic coordinates for optimized structure of *trans*-**7NiCu**.

Ni	-0.194315000	-1.210218000	-0.079789000
Cu	0.338363000	1.313567000	-0.457453000
Cl	-0.150713000	-0.701683000	-2.255102000
P	2.423970000	0.783077000	-0.101158000
P	-2.363325000	-0.555685000	-0.122306000
P	-0.028379000	-1.698920000	2.021583000
F	5.062794000	-0.757131000	-0.583814000
F	5.356398000	-3.376138000	-1.046796000
F	3.202358000	-5.010167000	-1.007434000
F	0.758458000	-4.038352000	-0.491907000
F	-4.954702000	0.990466000	-0.609932000
F	-5.250325000	3.615576000	-0.987782000
F	-3.095989000	5.239304000	-1.050334000
F	-0.627871000	4.241871000	-0.754244000
C	1.586911000	-1.833414000	-0.298558000
C	2.711099000	-0.993265000	-0.324486000
C	3.970600000	-1.526017000	-0.572513000
C	4.146796000	-2.875494000	-0.807952000
C	3.042271000	-3.710396000	-0.786254000
C	1.793586000	-3.177249000	-0.526421000
C	-1.441839000	2.029576000	-0.553531000
C	-2.578741000	1.200942000	-0.531102000
C	-3.851296000	1.744821000	-0.666114000
C	-4.030418000	3.102124000	-0.848875000
C	-2.922864000	3.931682000	-0.879149000
C	-1.665047000	3.378093000	-0.727188000
C	1.495111000	-2.492062000	2.609681000
H	1.428353000	-2.593616000	3.694388000
H	2.365279000	-1.891753000	2.353181000
H	1.599645000	-3.479662000	2.162524000
C	-1.272376000	-2.842600000	2.678855000
H	-1.031863000	-3.066324000	3.719584000
H	-1.247030000	-3.763719000	2.095644000
H	-2.268965000	-2.414292000	2.621113000

C	-0.103604000	-0.230071000	3.079494000
H	-1.008990000	0.338428000	2.886556000
H	0.759301000	0.399283000	2.862158000
H	-0.077662000	-0.534472000	4.127402000
C	3.517273000	1.613203000	-1.290017000
C	4.524961000	2.490770000	-0.907457000
H	4.722500000	2.669017000	0.142441000
C	5.284549000	3.135886000	-1.873392000
H	6.069419000	3.818604000	-1.570369000
C	5.044523000	2.904401000	-3.219966000
H	5.640810000	3.407525000	-3.971642000
C	4.035787000	2.029264000	-3.603904000
H	3.842020000	1.848995000	-4.654457000
C	3.267685000	1.391330000	-2.644261000
H	2.470535000	0.717973000	-2.942351000
C	3.047628000	1.233612000	1.540207000
C	3.955932000	0.463883000	2.260581000
H	4.354523000	-0.450671000	1.840762000
C	4.351222000	0.860258000	3.529921000
H	5.052045000	0.251101000	4.087880000
C	3.852433000	2.031413000	4.082612000
H	4.162197000	2.337359000	5.074664000
C	2.950992000	2.807344000	3.365983000
H	2.554476000	3.719465000	3.795243000
C	2.542244000	2.405181000	2.104752000
H	1.815237000	2.993111000	1.553944000
C	-3.115736000	-1.604294000	-1.397905000
C	-2.907018000	-2.978525000	-1.278864000
H	-2.335385000	-3.369216000	-0.443414000
C	-3.408271000	-3.848577000	-2.233077000
H	-3.242062000	-4.914056000	-2.129857000
C	-4.106477000	-3.351223000	-3.325141000
H	-4.492218000	-4.028874000	-4.077234000
C	-4.296102000	-1.983342000	-3.458666000
H	-4.826136000	-1.589463000	-4.317502000
C	-3.803110000	-1.110472000	-2.499587000
H	-3.940877000	-0.045483000	-2.628215000
C	-3.515905000	-0.708429000	1.292509000
C	-4.387404000	-1.784511000	1.437770000
H	-4.436961000	-2.552451000	0.677313000
C	-5.203054000	-1.881465000	2.555665000
H	-5.877455000	-2.723828000	2.652708000
C	-5.160529000	-0.905618000	3.540617000
H	-5.797624000	-0.983745000	4.413261000
C	-4.308403000	0.180174000	3.393783000
H	-4.280455000	0.957745000	4.147667000
C	-3.496081000	0.280231000	2.275802000
H	-2.856661000	1.147394000	2.158885000

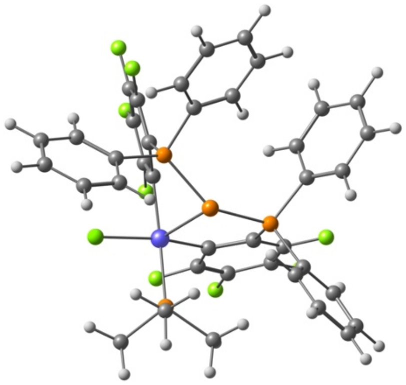

Figure S103. Optimized molecular structure of *cis*-**8NiCu**.

PBE0:

final single point energy: -6983.207361617735 a.u.

final Gibbs free energy: -6982.70178596 a.u.

Table S36. Atomic coordinates for optimized structure of *cis*-**8NiCu**.

Ni	-0.176910000	-1.883598000	-0.273187000
Cu	0.148925000	0.557963000	-0.950075000
P	-1.788263000	1.191610000	-0.159190000
Cl	1.546832000	-2.838576000	-1.292631000
C	-2.433706000	-0.208501000	0.780088000
F	-0.072990000	-2.862449000	2.720433000
C	-2.981772000	1.681613000	-1.435833000
C	3.071409000	-0.369222000	2.076446000
C	-2.419307000	-2.535179000	1.220401000
C	-4.134880000	-1.185305000	2.194231000
C	-1.784706000	-1.449744000	0.632866000
C	-3.570527000	-0.092136000	1.576791000
C	2.141504000	-0.530386000	1.058631000
C	0.924483000	-2.003227000	2.444142000
C	2.926852000	-1.004615000	3.293233000
C	1.016885000	-1.381345000	1.213287000
F	-5.221692000	-1.064980000	2.953017000
F	4.155729000	0.400641000	1.923276000
F	-1.941429000	-3.778343000	1.038817000
F	-4.163787000	1.091831000	1.774656000
F	3.820868000	-0.838988000	4.265654000
F	-4.107242000	-3.498427000	2.556628000
F	1.676985000	-2.452343000	4.639324000
C	-3.556905000	-2.428589000	1.994242000
P	2.275468000	0.443019000	-0.467781000
C	3.585176000	-0.197919000	-1.532644000
C	2.865011000	2.092205000	0.034599000
P	-1.351017000	-2.373925000	-2.079442000
C	-1.684758000	2.577671000	1.003986000
C	1.834299000	-1.826802000	3.475962000
C	2.068232000	2.820847000	0.916666000
H	1.156151000	2.384926000	1.306986000
C	2.434153000	4.099502000	1.300034000
H	1.807710000	4.650844000	1.991106000

C	3.594026000	4.671811000	0.791622000
H	3.879376000	5.674728000	1.085800000
C	4.384165000	3.955160000	-0.094830000
H	5.290308000	4.395731000	-0.493477000
C	4.024928000	2.667841000	-0.471860000
H	4.654812000	2.112646000	-1.155508000
C	4.657757000	-0.940762000	-1.051846000
H	4.724435000	-1.188617000	-0.000551000
C	5.638896000	-1.383002000	-1.925375000
H	6.469094000	-1.967684000	-1.547843000
C	5.557184000	-1.082648000	-3.278621000
H	6.324888000	-1.432402000	-3.958369000
C	4.487579000	-0.341437000	-3.761309000
H	4.415987000	-0.111367000	-4.817510000
C	3.500925000	0.095650000	-2.891149000
H	2.655056000	0.660304000	-3.268436000
C	-2.441189000	2.047466000	-2.668498000
H	-1.365090000	2.030318000	-2.806738000
C	-3.266392000	2.424847000	-3.717108000
H	-2.833210000	2.708438000	-4.668521000
C	-4.642518000	2.424266000	-3.546327000
H	-5.291478000	2.707645000	-4.366201000
C	-5.188706000	2.055587000	-2.323662000
C	-4.366007000	1.691201000	-1.269885000
C	-1.920448000	3.884935000	0.590794000
H	-2.261932000	4.083964000	-0.418128000
C	-1.723085000	4.936993000	1.473431000
H	-1.912552000	5.952640000	1.147423000
C	-1.288667000	4.690868000	2.768047000
H	-1.136689000	5.514094000	3.455553000
C	-1.050340000	3.386490000	3.181542000
H	-0.711258000	3.188043000	4.191119000
C	-1.243792000	2.332619000	2.303580000
H	-1.051124000	1.316056000	2.628720000
H	-6.263492000	2.051733000	-2.188267000
H	-4.809626000	1.412603000	-0.325305000
C	-3.117410000	-1.959355000	-2.166540000
H	-3.520362000	-2.283639000	-3.127510000
H	-3.656222000	-2.458424000	-1.361364000
H	-3.257416000	-0.884253000	-2.063979000
C	-0.690178000	-1.571986000	-3.569585000
H	0.360721000	-1.835320000	-3.677089000
H	-1.251240000	-1.887854000	-4.450995000
H	-0.776204000	-0.488944000	-3.459776000
C	-1.359508000	-4.144041000	-2.479039000
H	-1.897517000	-4.313452000	-3.413523000
H	-0.333570000	-4.496265000	-2.566038000
H	-1.854066000	-4.685623000	-1.672110000

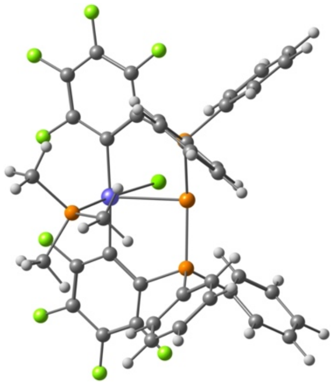

Figure S104. Optimized molecular structure of *trans*-**8NiCu**.

PBE0:

final single point energy: -6983.228153946684 a.u.

final Gibbs free energy: -6982.72096042 a.u.

Table S37. Atomic coordinates for optimized structure of *trans*-**8NiCu**.

Ni	-0.077864000	1.698045000	-0.209441000
Cu	0.011599000	-0.787903000	-0.757534000
Cl	-0.019382000	1.009362000	-2.367204000
F	-5.071266000	-0.283210000	-0.288736000
F	-6.129801000	2.163334000	-0.555241000
F	-4.517235000	4.331591000	-0.635491000
F	-1.866428000	4.072736000	-0.415381000
P	-2.107446000	-1.046806000	-0.207894000
P	-0.127631000	2.024239000	1.911583000
C	-2.005396000	1.716163000	-0.273449000
C	-2.862428000	0.599811000	-0.257419000
C	-4.240875000	0.763413000	-0.341516000
C	-4.809462000	2.014054000	-0.470311000
C	-3.980490000	3.122353000	-0.507583000
C	-2.613434000	2.949671000	-0.401313000
C	-2.466874000	-1.745221000	1.425660000
C	-1.796142000	-2.913310000	1.787986000
H	-1.124785000	-3.387084000	1.080824000
C	-1.973198000	-3.461685000	3.046881000
H	-1.446821000	-4.368895000	3.318017000
C	-2.810083000	-2.838053000	3.963805000
H	-2.939545000	-3.258945000	4.953600000
C	-3.473904000	-1.671895000	3.612188000
H	-4.124118000	-1.179685000	4.325357000
C	-3.306830000	-1.127399000	2.346740000
H	-3.820700000	-0.210691000	2.087201000
C	-3.039721000	-2.055953000	-1.397460000
C	-3.809660000	-3.154534000	-1.033596000
H	-3.908968000	-3.428517000	0.009397000
C	-4.463292000	-3.896169000	-2.007645000
H	-5.063427000	-4.750548000	-1.717980000
C	-4.357616000	-3.541283000	-3.344645000
H	-4.872799000	-4.119738000	-4.102083000
C	-3.588695000	-2.443570000	-3.710860000

H	-3.501428000	-2.162942000	-4.753667000
C	-2.924156000	-1.708148000	-2.743452000
H	-2.314732000	-0.856906000	-3.029345000
C	1.127499000	3.115307000	2.639950000
H	0.964133000	3.174654000	3.717267000
H	2.122163000	2.717693000	2.444316000
C	0.121319000	0.475147000	2.818328000
H	0.037081000	0.666941000	3.889784000
H	1.110517000	0.077470000	2.600480000
H	-0.625349000	-0.259109000	2.522009000
H	1.051201000	4.111330000	2.205489000
C	-1.655121000	2.675654000	2.646856000
H	-1.524244000	2.752085000	3.727576000
H	-1.871676000	3.662493000	2.238437000
H	-2.488624000	2.010781000	2.425997000
F	5.041409000	0.026675000	-0.224327000
F	5.946384000	2.538080000	-0.425607000
F	4.198411000	4.601661000	-0.502637000
F	1.571434000	4.172955000	-0.344648000
P	2.145103000	-0.931401000	-0.229028000
C	1.852670000	1.827362000	-0.197100000
C	2.780626000	0.767214000	-0.183017000
C	4.146703000	1.020348000	-0.253203000
C	4.637324000	2.305652000	-0.355898000
C	3.740006000	3.359044000	-0.391930000
C	2.385304000	3.097918000	-0.308913000
C	2.577051000	-1.734726000	1.337036000
C	1.946152000	-2.944751000	1.623559000
H	1.256090000	-3.371231000	0.904020000
C	2.187196000	-3.595340000	2.822007000
H	1.691756000	-4.535164000	3.034019000
C	3.048741000	-3.032620000	3.754881000
H	3.228830000	-3.533513000	4.698438000
C	3.672116000	-1.824071000	3.479753000
H	4.340301000	-1.378834000	4.207028000
C	3.441679000	-1.177317000	2.273877000
H	3.925271000	-0.229554000	2.075855000
C	3.136011000	-1.776077000	-1.499238000
C	3.934942000	-2.881926000	-1.232234000
H	4.025708000	-3.257278000	-0.220531000
C	4.628332000	-3.502023000	-2.262317000
H	5.250807000	-4.362370000	-2.046958000
C	4.533447000	-3.018576000	-3.559082000
H	5.079459000	-3.501901000	-4.360336000
C	3.735791000	-1.913332000	-3.828288000
H	3.656682000	-1.531903000	-4.839308000
C	3.032729000	-1.298134000	-2.805757000
H	2.400703000	-0.442086000	-3.017706000

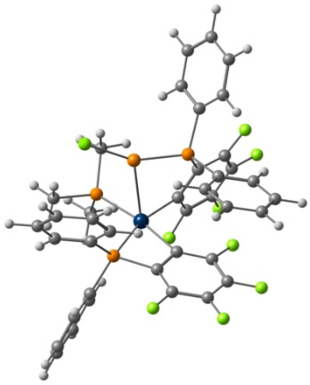

Figure S105. Optimized molecular structure of *cis*-**6PtCu**.

PBE0:

final single point energy: -24494.524791291533 a.u.

final Gibbs free energy: -24494.01893571 a.u.

Table S38. Atomic coordinates for optimized structure of *cis*-**6PtCu**.

Pt	3.407211000	8.689803000	4.105885000
Cu	0.847259000	9.116618000	2.995678000
Cl	0.485682000	10.577268000	1.480996000
P	4.408817000	8.439991000	2.033361000
P	0.310060000	7.703729000	4.543353000
P	3.222397000	10.962621000	4.399359000
F	5.185440000	5.517354000	0.517312000
F	4.933292000	3.172673000	1.867071000
F	4.075478000	3.162665000	4.403759000
F	3.394137000	5.410035000	5.665505000
F	-0.471918000	7.042056000	7.492464000
F	1.044370000	7.334233000	9.666011000
F	3.597856000	8.194918000	9.435906000
F	4.637315000	8.761034000	7.048773000
C	3.848163000	6.709309000	3.723181000
C	4.344595000	6.684754000	2.406272000
C	4.718473000	5.521968000	1.766449000
C	4.603682000	4.323452000	2.446847000
C	4.151774000	4.322853000	3.760148000
C	3.790953000	5.503832000	4.391437000
C	2.586657000	8.322266000	5.946001000
C	1.260057000	7.871515000	6.081166000
C	0.762507000	7.526635000	7.330920000
C	1.535910000	7.648214000	8.469635000
C	2.841891000	8.085362000	8.348898000
C	3.344342000	8.393481000	7.097427000
C	1.654918000	11.592880000	5.057327000
H	1.742280000	12.659777000	5.269936000
H	1.411091000	11.053611000	5.973028000
H	0.859571000	11.433520000	4.328367000
C	3.538205000	11.970626000	2.930919000
H	3.460819000	13.031396000	3.175258000
H	2.811359000	11.714823000	2.159987000
H	4.543792000	11.754322000	2.567464000

C	4.432903000	11.596988000	5.594596000
H	5.440650000	11.331984000	5.276046000
H	4.249616000	11.160740000	6.574753000
H	4.345054000	12.683325000	5.655517000
C	6.144693000	8.914601000	1.860352000
C	6.802181000	9.424397000	2.976229000
H	6.252960000	9.554599000	3.901963000
C	8.148155000	9.750104000	2.903560000
H	8.654678000	10.147649000	3.774654000
C	8.840363000	9.567671000	1.715076000
H	9.891379000	9.824071000	1.655807000
C	8.188159000	9.056152000	0.599743000
H	8.729823000	8.910962000	-0.327095000
C	6.843682000	8.729458000	0.668597000
H	6.338427000	8.331848000	-0.203163000
C	3.648880000	8.751112000	0.429344000
C	3.887488000	9.950522000	-0.237876000
H	4.565915000	10.683070000	0.182646000
C	3.262269000	10.206539000	-1.446803000
H	3.452650000	11.139724000	-1.962808000
C	2.393651000	9.271588000	-1.994031000
H	1.905414000	9.474383000	-2.939538000
C	2.151455000	8.077911000	-1.329708000
H	1.475663000	7.345380000	-1.754029000
C	2.775589000	7.817030000	-0.120013000
H	2.585711000	6.883197000	0.395019000
C	0.462720000	5.975834000	4.027409000
C	0.407745000	4.912105000	4.924164000
H	0.259483000	5.093555000	5.981190000
C	0.558624000	3.613046000	4.466642000
H	0.525163000	2.789060000	5.168921000
C	0.763201000	3.367799000	3.114698000
H	0.892019000	2.350997000	2.763521000
C	0.811953000	4.423777000	2.216187000
H	0.977214000	4.237270000	1.161942000
C	0.664217000	5.724140000	2.672533000
H	0.717678000	6.555472000	1.978046000
C	-1.438254000	7.946719000	4.970396000
C	-1.809487000	9.182644000	5.499786000
H	-1.054429000	9.936715000	5.695150000
C	-3.138534000	9.450332000	5.777915000
H	-3.418225000	10.409785000	6.196242000
C	-4.111827000	8.494532000	5.510939000
H	-5.152949000	8.707036000	5.721931000
C	-3.747754000	7.271430000	4.968630000
H	-4.503209000	6.524804000	4.754721000
C	-2.414087000	6.994739000	4.699084000
H	-2.136892000	6.035388000	4.280234000

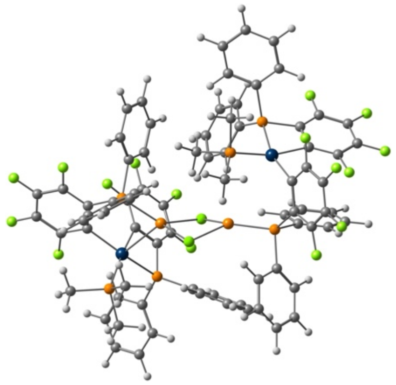

Figure S106. Optimized molecular structure of *cis*-**6PtCu-dimer**.

PBE0:

final single point energy: -48989.075280179866 a.u.
final Gibbs free energy: -48988.03520863 a.u.

Table S39. Atomic coordinates for optimized structure of *cis*-**6PtCu-dimer** .

Pt	0.879589000	13.244383000	22.083851000
Pt	-2.448825000	20.015231000	26.434462000
Cu	-0.492497000	16.726068000	22.353985000
Cu	-2.272238000	17.681939000	24.770570000
Cl	-0.011708000	17.533167000	24.450527000
Cl	-2.696867000	17.453699000	22.419851000
P	1.865984000	12.579759000	24.062888000
P	0.722397000	16.306888000	20.587279000
P	-1.338734000	13.130984000	22.589121000
P	-0.504339000	21.033081000	25.733848000
P	-3.819683000	16.873577000	26.087574000
P	-4.072989000	21.032550000	25.176373000
F	5.116990000	11.788451000	24.539018000
F	6.868292000	11.886595000	22.463293000
F	6.014666000	12.566248000	20.012646000
F	3.468683000	13.166618000	19.530466000
F	0.425525000	16.087113000	17.439865000
F	0.006414000	13.917480000	16.016090000
F	-0.074365000	11.480817000	17.198304000
F	0.285021000	11.241417000	19.825565000
F	2.624642000	20.477969000	26.974573000
F	2.983635000	18.747342000	29.019655000
F	0.887713000	17.466578000	30.097871000
F	-1.592241000	17.840086000	29.201380000
F	-5.813343000	16.148879000	28.286105000
F	-6.552680000	17.612269000	30.397146000
F	-5.544408000	20.101673000	30.734868000
F	-3.810014000	21.120013000	28.991218000
C	2.903398000	12.910278000	21.828777000
C	3.377612000	12.516888000	23.096117000
C	4.689850000	12.166402000	23.333491000
C	5.587974000	12.201632000	22.282079000
C	5.141451000	12.555188000	21.015650000
C	3.813549000	12.888124000	20.793001000

C	0.483795000	13.587797000	20.114218000
C	0.488411000	14.855170000	19.497304000
C	0.349751000	14.936344000	18.114027000
C	0.148780000	13.810184000	17.336496000
C	0.113867000	12.569355000	17.938512000
C	0.292748000	12.484283000	19.305499000
C	-2.538389000	13.818708000	21.420831000
H	-3.553883000	13.617834000	21.767192000
H	-2.387623000	13.363984000	20.441970000
H	-2.392339000	14.896055000	21.339457000
C	-1.834947000	13.794853000	24.189830000
H	-1.742211000	14.881260000	24.184157000
H	-1.182784000	13.399944000	24.967215000
H	-2.863717000	13.518085000	24.418797000
C	-1.816786000	11.382450000	22.689508000
H	-1.217426000	10.885035000	23.452284000
H	-1.630448000	10.902337000	21.729172000
H	-2.875107000	11.297807000	22.943898000
C	1.441018000	10.916639000	24.628154000
C	0.552441000	10.756351000	25.690348000
H	0.199368000	11.621031000	26.240232000
C	0.116293000	9.489845000	26.045770000
H	-0.574161000	9.372588000	26.872460000
C	0.563267000	8.377353000	25.345380000
H	0.223472000	7.387602000	25.625520000
C	1.446794000	8.534533000	24.286134000
H	1.798630000	7.668727000	23.738297000
C	1.881744000	9.800011000	23.923100000
H	2.562288000	9.920414000	23.088356000
C	2.110341000	13.544190000	25.565690000
C	1.656156000	14.855647000	25.592203000
H	1.125559000	15.262772000	24.741445000
C	1.872517000	15.644623000	26.712245000
H	1.507465000	16.663743000	26.717082000
C	2.537322000	15.120347000	27.809805000
H	2.703882000	15.732425000	28.688129000
C	2.988225000	13.804564000	27.788780000
H	3.506526000	13.394340000	28.647076000
C	2.778626000	13.015397000	26.670814000
H	3.132275000	11.991573000	26.657039000
C	2.509718000	16.332115000	20.899231000
C	2.945525000	16.555881000	22.202328000
H	2.215676000	16.716156000	22.987564000
C	4.300524000	16.557780000	22.497001000
H	4.629236000	16.720043000	23.516553000
C	5.227427000	16.337311000	21.488661000
H	6.286511000	16.327021000	21.717579000
C	4.798434000	16.123291000	20.184475000
H	5.521108000	15.944670000	19.397326000
C	3.444902000	16.117967000	19.889926000
H	3.116508000	15.925813000	18.875660000

C	0.391246000	17.769163000	19.547553000
C	1.379688000	18.623212000	19.074449000
H	2.420107000	18.431507000	19.302720000
C	1.037962000	19.727797000	18.306295000
H	1.816334000	20.388100000	17.942221000
C	-0.290933000	19.989306000	18.008160000
H	-0.554248000	20.852912000	17.409298000
C	-1.284385000	19.144831000	18.489487000
H	-2.325869000	19.347968000	18.270629000
C	-0.945813000	18.047355000	19.261951000
H	-1.724693000	17.403908000	19.654954000
C	-0.827104000	19.346836000	27.523608000
C	0.292813000	20.008600000	26.982625000
C	1.568607000	19.834483000	27.473186000
C	1.765504000	18.961091000	28.529130000
C	0.678677000	18.300797000	29.083511000
C	-0.602469000	18.503323000	28.590819000
C	-3.846740000	19.158842000	27.655539000
C	-4.375780000	17.870034000	27.497045000
C	-5.280770000	17.365781000	28.424791000
C	-5.681715000	18.103964000	29.518519000
C	-5.166322000	19.377553000	29.686606000
C	-4.269635000	19.875746000	28.761865000
C	-4.679188000	20.134961000	23.726365000
H	-5.104227000	19.181642000	24.040026000
H	-3.856394000	19.927925000	23.044038000
H	-5.443779000	20.726937000	23.219675000
C	-3.570322000	22.638840000	24.503888000
H	-2.722808000	22.500204000	23.831876000
H	-3.263186000	23.293275000	25.319790000
H	-4.396270000	23.096764000	23.957067000
C	-5.599004000	21.420310000	26.075972000
H	-5.367701000	22.036187000	26.944853000
H	-6.062173000	20.494279000	26.416357000
H	-6.289841000	21.954441000	25.421741000
C	-0.366593000	22.751035000	26.299249000
C	-0.174317000	23.800885000	25.406177000
H	-0.029902000	23.598816000	24.352281000
C	-0.167591000	25.111063000	25.863498000
H	-0.015951000	25.922549000	25.161897000
C	-0.350826000	25.380253000	27.211695000
H	-0.342284000	26.403630000	27.567146000
C	-0.545689000	24.334874000	28.105980000
H	-0.689710000	24.539714000	29.159977000
C	-0.559528000	23.026307000	27.653158000
H	-0.723161000	22.214189000	28.351872000
C	0.320081000	20.999702000	24.136054000
C	-0.395761000	20.607606000	23.010641000
H	-1.423962000	20.282421000	23.113299000
C	0.218009000	20.601340000	21.767714000
H	-0.336063000	20.287983000	20.892148000

C	1.548239000	20.977282000	21.652079000
H	2.028061000	20.963662000	20.682126000
C	2.268209000	21.362730000	22.775289000
H	3.308210000	21.651601000	22.683274000
C	1.655537000	21.380902000	24.016520000
H	2.213918000	21.693244000	24.889789000
C	-3.241357000	15.305060000	26.816694000
C	-4.035268000	14.171239000	26.962088000
H	-5.070505000	14.189663000	26.646361000
C	-3.499446000	13.012312000	27.503469000
H	-4.121698000	12.131186000	27.606124000
C	-2.173085000	12.979621000	27.914068000
H	-1.757996000	12.071531000	28.335491000
C	-1.377053000	14.107982000	27.773830000
H	-0.336421000	14.086482000	28.074911000
C	-1.906453000	15.260940000	27.216753000
H	-1.273769000	16.128091000	27.066558000
C	-5.336094000	16.475108000	25.169128000
C	-5.253205000	15.561525000	24.118158000
H	-4.316907000	15.060077000	23.912969000
C	-6.354586000	15.305710000	23.319915000
H	-6.275162000	14.590191000	22.510024000
C	-7.550491000	15.975283000	23.546918000
H	-8.410926000	15.780284000	22.918215000
C	-7.635441000	16.899546000	24.577287000
H	-8.562406000	17.430800000	24.757383000
C	-6.534868000	17.148855000	25.385347000
H	-6.613735000	17.877056000	26.183174000

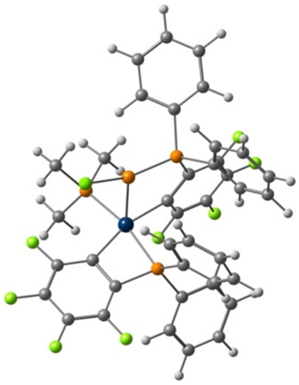

Figure S107. Optimized molecular structure of *trans*-**6PtCu**.

PBE0:

final single point energy: -24494.520853239501 a.u.

final Gibbs free energy: -24494.01523852 a.u.

Table S40. Atomic coordinates for optimized structure of *trans*-**6PtCu**.

Pt	0.031511000	-0.064349000	0.109379000
Cu	0.026520000	-0.011098000	2.863387000
Cl	-1.933529000	0.316272000	3.668945000
P	-0.385906000	-2.327253000	-0.132487000
P	2.155236000	-0.409898000	2.829219000
P	0.068709000	2.221535000	0.028737000
F	-3.326266000	-3.813430000	-0.695604000
F	-5.632598000	-2.367115000	-0.709646000
F	-5.547725000	0.287609000	-0.319937000
F	-3.267000000	1.577153000	0.087829000
F	5.091843000	-0.762741000	2.064527000
F	6.189097000	-0.668645000	-0.372809000
F	4.624616000	-0.252907000	-2.541762000
F	1.998464000	0.079642000	-2.286465000
C	-2.003571000	-0.431771000	-0.098531000
C	-2.091875000	-1.824933000	-0.316384000
C	-3.281888000	-2.493701000	-0.512929000
C	-4.460004000	-1.771039000	-0.516156000
C	-4.410115000	-0.398234000	-0.313625000
C	-3.201848000	0.250456000	-0.107090000
C	2.085305000	-0.168091000	0.070760000
C	2.917715000	-0.375296000	1.184804000
C	4.288911000	-0.541029000	1.020903000
C	4.876944000	-0.501552000	-0.226101000
C	4.073602000	-0.290124000	-1.333289000
C	2.712523000	-0.127233000	-1.164382000
C	-0.775521000	3.107338000	1.361930000
H	-0.720605000	4.181259000	1.173937000
H	-0.289761000	2.876136000	2.309444000
H	-1.812847000	2.789951000	1.425216000
C	1.714928000	2.978904000	0.026103000
H	2.273633000	2.643625000	-0.846896000
H	2.258256000	2.683061000	0.922856000

H	1.615595000	4.065319000	-0.000905000
C	-0.673436000	2.852536000	-1.498646000
H	-1.715770000	2.545021000	-1.555380000
H	-0.133576000	2.437915000	-2.350108000
H	-0.609361000	3.942045000	-1.520979000
C	0.282916000	-3.069201000	-1.628517000
C	1.658168000	-3.300146000	-1.684871000
H	2.282293000	-3.079390000	-0.826134000
C	2.228750000	-3.804257000	-2.840925000
H	3.296862000	-3.980202000	-2.880958000
C	1.433450000	-4.073902000	-3.947787000
H	1.881608000	-4.463710000	-4.853735000
C	0.067013000	-3.839924000	-3.895458000
H	-0.554059000	-4.047437000	-4.758323000
C	-0.510763000	-3.336734000	-2.738618000
H	-1.576626000	-3.148309000	-2.705837000
C	-0.288950000	-3.581408000	1.164301000
C	-0.761121000	-3.246150000	2.432074000
H	-1.158432000	-2.253927000	2.621454000
C	-0.735083000	-4.181023000	3.451678000
H	-1.098748000	-3.910538000	4.435430000
C	-0.239672000	-5.456245000	3.212654000
H	-0.211705000	-6.185079000	4.013627000
C	0.221941000	-5.795299000	1.949953000
H	0.605319000	-6.790460000	1.759545000
C	0.198725000	-4.862102000	0.923978000
H	0.556696000	-5.135654000	-0.060142000
C	2.561289000	-2.028567000	3.538298000
C	2.348427000	-2.227606000	4.902359000
H	1.995612000	-1.410111000	5.521069000
C	2.592038000	-3.465765000	5.472062000
H	2.432598000	-3.608919000	6.534019000
C	3.033729000	-4.520450000	4.683380000
H	3.220518000	-5.490103000	5.128920000
C	3.223298000	-4.332393000	3.323049000
H	3.552955000	-5.155094000	2.700415000
C	2.987645000	-3.091572000	2.749839000
H	3.135279000	-2.955342000	1.685547000
C	3.068697000	0.795846000	3.843508000
C	2.578613000	2.100085000	3.871511000
H	1.673889000	2.342513000	3.326323000
C	3.229654000	3.081899000	4.601801000
H	2.839553000	4.092433000	4.613777000
C	4.367249000	2.761527000	5.328881000
H	4.873247000	3.523857000	5.909082000
C	4.849808000	1.459424000	5.321240000
H	5.733044000	1.204256000	5.894493000
C	4.207286000	0.479813000	4.580447000
H	4.592150000	-0.531294000	4.579104000

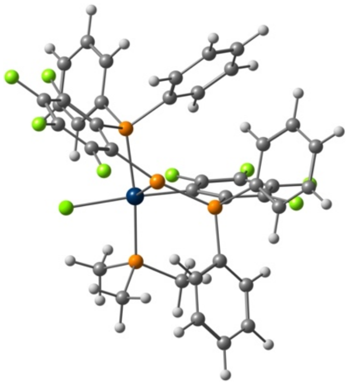

Figure S108. Optimized molecular structure of *cis-trans*-**7PtCu**.

PBE0:

final single point energy: -24494.515064198888 a.u.

final Gibbs free energy: -24494.00836186 a.u.

Table S41. Atomic coordinates for optimized structure of *cis-trans*-**7PtCu**.

Pt	3.799107000	6.717342000	4.790928000
Cu	1.407395000	5.612498000	3.849784000
Cl	4.181296000	4.978878000	6.377468000
P	4.222374000	5.181830000	3.080724000
P	0.812547000	7.679405000	3.497053000
C	2.256872000	8.705452000	3.097315000
F	0.001854000	2.760423000	4.432742000
C	-0.061092000	8.472147000	4.870164000
C	3.938231000	2.351748000	3.390410000
C	4.607686000	9.064448000	3.218822000
C	3.260954000	10.610515000	1.992345000
C	3.518179000	8.281279000	3.565834000
C	2.151224000	9.866516000	2.338462000
C	3.382660000	3.616615000	3.448329000
C	1.303519000	2.672340000	4.089189000
C	3.172917000	1.238070000	3.701476000
C	2.033193000	3.800913000	3.810238000
F	3.143280000	11.713865000	1.259098000
F	5.209251000	2.130884000	3.044478000
F	5.843617000	8.740916000	3.633291000
F	0.969270000	10.331431000	1.927153000
F	3.708544000	0.019491000	3.663118000
F	5.579549000	10.906944000	2.115174000
F	1.118433000	0.324570000	4.355044000
C	4.503721000	10.199690000	2.437371000
C	3.682814000	5.576232000	1.387630000
C	6.010432000	4.929848000	2.873916000
P	3.670607000	8.121138000	6.603735000
C	-0.304784000	7.817372000	2.072996000
C	1.845926000	1.397201000	4.052813000
C	6.859251000	5.186595000	3.947237000
H	6.444352000	5.524316000	4.888859000
C	8.227924000	5.006723000	3.811993000
H	8.881130000	5.208422000	4.652369000

C	8.757020000	4.577350000	2.603930000
H	9.826760000	4.442289000	2.496947000
C	7.914277000	4.325202000	1.528787000
H	8.323541000	3.991180000	0.582923000
C	6.547117000	4.501196000	1.661165000
H	5.896818000	4.302010000	0.818635000
C	2.795609000	4.769314000	0.681302000
H	2.381801000	3.884037000	1.145131000
C	2.441096000	5.093590000	-0.621468000
H	1.751956000	4.455890000	-1.161651000
C	2.959995000	6.228602000	-1.224795000
H	2.677152000	6.483703000	-2.238981000
C	3.844835000	7.039109000	-0.523930000
H	4.258916000	7.925458000	-0.989184000
C	4.209077000	6.711683000	0.771058000
H	4.922567000	7.332323000	1.296654000
C	-0.746382000	7.637400000	5.751460000
H	-0.714877000	6.562792000	5.605087000
C	-1.452540000	8.174290000	6.817245000
H	-1.981591000	7.518509000	7.497828000
C	-1.463884000	9.547259000	7.017922000
H	-2.004247000	9.967660000	7.857497000
C	-0.779182000	10.383021000	6.145308000
C	-0.085612000	9.850002000	5.070061000
C	-1.638006000	8.189990000	2.197145000
H	-2.037508000	8.476519000	3.162254000
C	-2.459463000	8.202442000	1.077876000
H	-3.497623000	8.495605000	1.178947000
C	-1.954743000	7.846673000	-0.164252000
H	-2.598262000	7.860699000	-1.035668000
C	-0.622707000	7.469880000	-0.287891000
H	-0.221388000	7.187416000	-1.253947000
C	0.197000000	7.447186000	0.826806000
H	1.233180000	7.145475000	0.728693000
H	-0.786463000	11.454991000	6.301144000
H	0.437637000	10.512051000	4.391813000
C	3.410306000	9.885254000	6.299919000
H	3.359721000	10.409392000	7.255581000
H	4.237534000	10.282578000	5.712631000
H	2.482264000	10.037645000	5.752342000
C	2.380823000	7.676219000	7.788233000
H	2.498453000	6.627317000	8.057217000
H	2.462310000	8.303512000	8.677517000
H	1.404243000	7.820306000	7.327287000
C	5.213516000	8.083621000	7.548249000
H	5.128933000	8.732605000	8.421731000
H	5.413476000	7.059528000	7.858946000
H	6.028493000	8.429594000	6.912095000

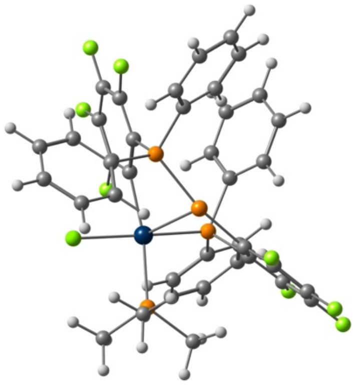

Figure S109. Optimized molecular structure of *cis*-*cis*-**7PtCu**.

PBE0:
final single point energy: -24494.513582859345 a.u.
final Gibbs free energy: -24494.00651387 a.u.

Table S42. Atomic coordinates for optimized structure of *cis*-*cis*-**7PtCu**.

Pt	5.392765000	12.951117000	8.171154000
Cu	3.002915000	13.176222000	6.842590000
Cl	6.104211000	15.107297000	8.838502000
P	6.698643000	13.320750000	6.279130000
P	2.303099000	14.273063000	8.597157000
P	4.773526000	10.860426000	7.643825000
F	1.304804000	14.237158000	11.536402000
F	2.484522000	13.115649000	13.641723000
F	4.900558000	11.926088000	13.375695000
F	6.122378000	11.839738000	11.013079000
F	5.227832000	8.667529000	5.353923000
F	4.330867000	8.812053000	2.864379000
F	2.859374000	10.920176000	2.043710000
F	2.238293000	12.893991000	3.751252000
C	4.328385000	12.945917000	9.932921000
C	3.070173000	13.559930000	10.083388000
C	2.473353000	13.620260000	11.337688000
C	3.074514000	13.063904000	12.450238000
C	4.313006000	12.466464000	12.314118000
C	4.918275000	12.435867000	11.071531000
C	3.443564000	11.868666000	5.507474000
C	4.224005000	10.764983000	5.909897000
C	4.498725000	9.736245000	5.024828000
C	4.040196000	9.789388000	3.718739000
C	3.285836000	10.870263000	3.301661000
C	2.994818000	11.876468000	4.208471000
C	2.653652000	16.047351000	8.619787000
C	2.877267000	16.673515000	7.396687000
H	2.850312000	16.088925000	6.483263000
C	3.150394000	18.032127000	7.347461000
H	3.327123000	18.512632000	6.392813000
C	3.210860000	18.767847000	8.522083000
H	3.434858000	19.827297000	8.487058000
C	2.991838000	18.145677000	9.744689000

H	3.044007000	18.718213000	10.662848000
C	2.711254000	16.789956000	9.795701000
H	2.547851000	16.313913000	10.754052000
C	0.506158000	14.133680000	8.821073000
C	-0.346809000	15.220436000	8.666555000
H	0.056951000	16.206886000	8.474651000
C	-1.720531000	15.043673000	8.766291000
H	-2.379583000	15.895426000	8.647404000
C	-2.247293000	13.786669000	9.022715000
H	-3.319318000	13.653187000	9.104204000
C	-1.395759000	12.698651000	9.174692000
H	-1.799699000	11.713314000	9.375564000
C	-0.026753000	12.869413000	9.066638000
H	0.632975000	12.017539000	9.181233000
C	6.093701000	9.662329000	7.996893000
C	7.388032000	10.122406000	8.215355000
H	7.587748000	11.186455000	8.178654000
C	8.409740000	9.232177000	8.512277000
H	9.413038000	9.602900000	8.683112000
C	8.139414000	7.875781000	8.604478000
H	8.934059000	7.178630000	8.841954000
C	6.844565000	7.411098000	8.405525000
H	6.627962000	6.352881000	8.487465000
C	5.825666000	8.298198000	8.106973000
H	4.819282000	7.928080000	7.957877000
C	3.364605000	10.159559000	8.559656000
C	3.544228000	9.808513000	9.897765000
H	4.517359000	9.911591000	10.360204000
C	2.488466000	9.305311000	10.637105000
H	2.639674000	9.043401000	11.677303000
C	1.243664000	9.129341000	10.044417000
H	0.418234000	8.732478000	10.623036000
C	1.064879000	9.455236000	8.709714000
H	0.100163000	9.313352000	8.237682000
C	2.118858000	9.975052000	7.969217000
H	1.965132000	10.234658000	6.930610000
C	6.970788000	12.071862000	4.991314000
H	7.291228000	11.126801000	5.429668000
H	6.057050000	11.912475000	4.423551000
H	7.749790000	12.437593000	4.320294000
C	6.054082000	14.728719000	5.340604000
H	6.717685000	14.963912000	4.506869000
H	5.064636000	14.468103000	4.962443000
H	5.966534000	15.587127000	6.005193000
C	8.398470000	13.783487000	6.709759000
H	8.932401000	14.095312000	5.810658000
H	8.379024000	14.593088000	7.436312000
H	8.906216000	12.923215000	7.147717000

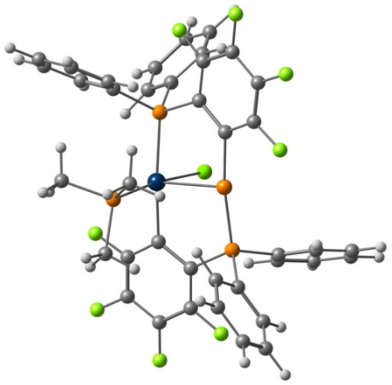

Figure S110. Optimized molecular structure of *trans*-**7PtCu**.

PBE0:

final single point energy: -24494.507532870259 a.u.

final Gibbs free energy: -24494.00014452 a.u.

Table S43. Atomic coordinates for optimized structure of *trans*-**7PtCu**.

Pt	22.168920000	4.094222000	5.939724000
Cu	24.340963000	5.579875000	6.380072000
Cl	21.379847000	6.108058000	4.958773000
P	24.929080000	5.268283000	4.292755000
P	21.483043000	4.874096000	8.032428000
P	23.065079000	2.165704000	6.648878000
F	25.131835000	4.320793000	1.397669000
F	23.522179000	2.651767000	0.076726000
F	21.351970000	1.603280000	1.307795000
F	20.811692000	2.193637000	3.850734000
F	21.466593000	6.034476000	10.863233000
F	23.424973000	7.326513000	12.123280000
F	25.765796000	7.820902000	10.870157000
F	26.150926000	7.031166000	8.345597000
C	22.704669000	3.604876000	4.024893000
C	23.829598000	4.143436000	3.376538000
C	24.085405000	3.812296000	2.051125000
C	23.262620000	2.957633000	1.344575000
C	22.151942000	2.426728000	1.974393000
C	21.895796000	2.759373000	3.291754000
C	23.996957000	6.060737000	8.210815000
C	22.787629000	5.803611000	8.888565000
C	22.607747000	6.242210000	10.197046000
C	23.605915000	6.918761000	10.870311000
C	24.802703000	7.172024000	10.223656000
C	24.963318000	6.744306000	8.918534000
C	23.990415000	1.225671000	5.406574000
H	24.446775000	0.365395000	5.899034000
H	24.766452000	1.845078000	4.960333000
H	23.321340000	0.878541000	4.621076000
C	21.895097000	0.929831000	7.261097000
H	22.440894000	0.021395000	7.522043000
H	21.175792000	0.709214000	6.472287000
H	21.362359000	1.299640000	8.133625000

C	24.295286000	2.357942000	7.960225000
H	23.845695000	2.796566000	8.846034000
H	25.082171000	3.025847000	7.610698000
H	24.716416000	1.382794000	8.209897000
C	24.924041000	6.833714000	3.377552000
C	25.871840000	7.796402000	3.726947000
H	26.619279000	7.573654000	4.480485000
C	25.863407000	9.037670000	3.113602000
H	26.606830000	9.776877000	3.386636000
C	24.900547000	9.334747000	2.156892000
H	24.892353000	10.307139000	1.679304000
C	23.946532000	8.386398000	1.820060000
H	23.188480000	8.615398000	1.080764000
C	23.954936000	7.139268000	2.428737000
H	23.197273000	6.410424000	2.170546000
C	26.613459000	4.605324000	4.096936000
C	27.464936000	4.951979000	3.050930000
H	27.138774000	5.654611000	2.295597000
C	28.734360000	4.400609000	2.975854000
H	29.391389000	4.677919000	2.160230000
C	29.163067000	3.495597000	3.937994000
H	30.155600000	3.065941000	3.873983000
C	28.323045000	3.152053000	4.987673000
H	28.656239000	2.457727000	5.749534000
C	27.058704000	3.714122000	5.071307000
H	26.412167000	3.470336000	5.906194000
C	20.044154000	5.979266000	7.886564000
C	18.961170000	5.550396000	7.118588000
H	19.010438000	4.605289000	6.591182000
C	17.837694000	6.346789000	6.982308000
H	17.003459000	5.999442000	6.384897000
C	17.794411000	7.597217000	7.584839000
H	16.920211000	8.226595000	7.469045000
C	18.883359000	8.045969000	8.315581000
H	18.867848000	9.029668000	8.769079000
C	20.003902000	7.240900000	8.467694000
H	20.849588000	7.617584000	9.026252000
C	20.931613000	3.611312000	9.235334000
C	19.682350000	3.010617000	9.086331000
H	19.002804000	3.341623000	8.313440000
C	19.292644000	1.976201000	9.921981000
H	18.317751000	1.522681000	9.790055000
C	20.146758000	1.520258000	10.916271000
H	19.843761000	0.707750000	11.565334000
C	21.387064000	2.118537000	11.078591000
H	22.057938000	1.781772000	11.859603000
C	21.774583000	3.158476000	10.247126000
H	22.738918000	3.624517000	10.405125000

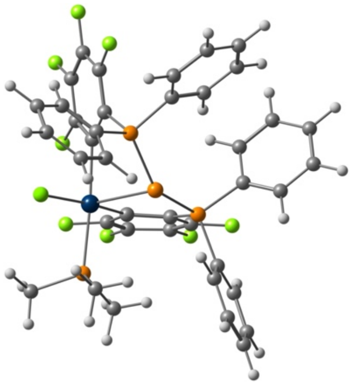

Figure S111. Optimized molecular structure of *cis*-**8PtCu**.

PBE0:

final single point energy: -24494.500661192014 a.u.

final Gibbs free energy: -24493.99530291 a.u.

Table S44. Atomic coordinates for optimized structure of *cis*-**8PtCu**.

Pt	3.905452000	6.341807000	4.670610000
Cu	1.625337000	5.110749000	4.426108000
Cl	4.859762000	4.830710000	6.239037000
P	0.650627000	6.788850000	3.419658000
C	1.962977000	7.900424000	2.848598000
F	5.991879000	6.524941000	2.290542000
C	-0.471034000	7.736693000	4.487490000
C	4.516163000	2.740283000	2.190214000
C	4.174179000	8.746766000	3.058850000
C	2.642680000	9.863163000	1.601512000
C	3.258091000	7.755114000	3.396354000
C	1.690535000	8.933049000	1.954508000
C	3.963195000	3.635520000	3.096920000
C	5.447322000	5.297162000	2.294912000
C	5.519115000	3.115676000	1.319126000
C	4.437273000	4.968809000	3.181081000
F	2.364039000	10.844679000	0.747676000
F	4.108991000	1.467776000	2.126226000
F	5.393827000	8.772227000	3.616000000
F	0.478940000	9.076992000	1.404573000
F	6.036651000	2.249026000	0.451755000
F	4.818803000	10.680967000	1.879555000
F	6.952012000	4.795584000	0.545546000
C	3.897407000	9.772700000	2.177358000
P	2.540425000	3.146596000	4.119106000
C	3.088234000	2.055908000	5.451535000
C	1.495817000	2.089400000	3.066920000
P	3.412400000	7.698856000	6.449867000
C	-0.313075000	6.327670000	1.954894000
C	5.984009000	4.413864000	1.372540000
C	0.894622000	2.683757000	1.958767000
H	1.086125000	3.727228000	1.736914000
C	0.048299000	1.952420000	1.143313000
H	-0.410020000	2.426867000	0.283752000

C	-0.220203000	0.621195000	1.438160000
H	-0.888915000	0.048876000	0.806449000
C	0.367024000	0.028421000	2.546411000
H	0.160998000	-1.009239000	2.780800000
C	1.225693000	0.756977000	3.358388000
H	1.687770000	0.283003000	4.215321000
C	4.284455000	1.348607000	5.413816000
H	4.952870000	1.448559000	4.568575000
C	4.636683000	0.527512000	6.473792000
H	5.574086000	-0.014694000	6.443492000
C	3.796239000	0.404517000	7.571948000
H	4.076998000	-0.235045000	8.400163000
C	2.600414000	1.108352000	7.612196000
H	1.945149000	1.021436000	8.470531000
C	2.249761000	1.936108000	6.557245000
H	1.324544000	2.501286000	6.595138000
C	-0.920189000	7.090003000	5.638039000
H	-0.601481000	6.071984000	5.836832000
C	-1.762407000	7.738115000	6.529332000
H	-2.103695000	7.224060000	7.419604000
C	-2.153307000	9.045257000	6.282109000
H	-2.802686000	9.559146000	6.980605000
C	-1.709240000	9.697038000	5.138457000
C	-0.876578000	9.047844000	4.241311000
C	-1.687333000	6.128180000	2.041018000
H	-2.212575000	6.349485000	2.962661000
C	-2.389780000	5.648500000	0.944874000
H	-3.460307000	5.498971000	1.018436000
C	-1.726542000	5.364508000	-0.240175000
H	-2.277241000	4.991145000	-1.095026000
C	-0.354469000	5.561808000	-0.328141000
H	0.169131000	5.343320000	-1.251067000
C	0.351732000	6.036709000	0.764798000
H	1.423721000	6.183844000	0.691448000
H	-2.012863000	10.718438000	4.943294000
H	-0.544780000	9.570011000	3.355368000
C	2.551948000	9.263324000	6.141440000
H	2.396188000	9.786515000	7.086232000
H	3.147411000	9.887809000	5.475856000
H	1.588146000	9.071792000	5.671479000
C	2.371639000	6.879359000	7.684918000
H	2.857774000	5.953833000	7.991027000
H	2.221614000	7.529355000	8.548574000
H	1.406983000	6.642453000	7.235545000
C	4.887640000	8.211111000	7.367371000
H	4.604902000	8.798415000	8.242742000
H	5.436691000	7.321929000	7.674774000
H	5.522272000	8.810712000	6.714229000

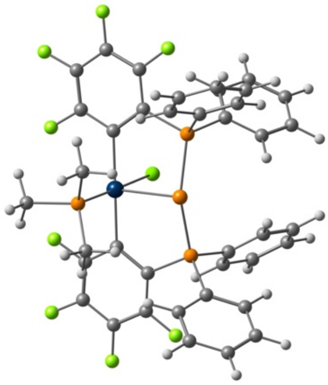

Figure S112. Optimized molecular structure of *trans*-**8PtCu**.

PBE0:

final single point energy: -24494.507077938222 a.u.

final Gibbs free energy: -24494.00126374 a.u.

Table S45. Atomic coordinates for optimized structure of *trans*-**8PtCu**.

Pt	3.830927000	6.056527000	6.706508000
Cu	5.758622000	6.051499000	8.451228000
Cl	5.488945000	6.055465000	5.001029000
F	5.602092000	0.948490000	7.670245000
F	3.618340000	-0.152716000	6.258841000
F	1.788647000	1.427413000	5.043285000
F	1.927357000	4.071933000	5.233720000
P	6.115589000	3.886772000	8.227549000
P	2.226302000	6.056496000	8.239031000
C	3.830951000	3.987961000	6.644622000
C	4.770471000	3.142895000	7.271886000
C	4.692698000	1.761508000	7.124459000
C	3.687727000	1.169771000	6.387561000
C	2.758848000	1.981186000	5.761894000
C	2.858324000	3.354361000	5.890299000
C	6.326601000	2.839183000	9.703586000
C	7.577562000	2.422240000	10.146366000
H	8.466537000	2.658816000	9.576432000
C	7.694151000	1.683480000	11.315457000
H	8.673883000	1.360398000	11.646650000
C	6.566088000	1.351849000	12.050608000
H	6.659773000	0.771741000	12.960645000
C	5.314554000	1.761091000	11.609451000
H	4.425751000	1.498456000	12.170672000
C	5.195710000	2.503433000	10.446303000
H	4.213496000	2.803658000	10.104152000
C	7.623354000	3.658778000	7.241636000
C	7.648448000	2.925547000	6.059890000
H	6.749895000	2.447531000	5.692555000
C	8.822771000	2.823830000	5.329200000
H	8.830249000	2.256917000	4.406154000
C	9.979210000	3.449352000	5.772235000
H	10.894022000	3.369338000	5.197481000
C	9.957569000	4.190566000	6.945491000

H	10.852189000	4.695112000	7.289277000
C	8.782769000	4.304045000	7.670127000
H	8.761554000	4.905199000	8.571726000
C	0.563827000	6.044472000	7.522492000
H	-0.178396000	6.047620000	8.322804000
H	0.434015000	6.924970000	6.894938000
C	2.205009000	7.466381000	9.369980000
H	1.348627000	7.384641000	10.041110000
H	2.149890000	8.399147000	8.810906000
H	3.124783000	7.454116000	9.954765000
H	0.440121000	5.154363000	6.907604000
C	2.221631000	4.657072000	9.383433000
H	1.367494000	4.737771000	10.057544000
H	2.172228000	3.718705000	8.833346000
H	3.143980000	4.682665000	9.963902000
F	5.659502000	11.148451000	7.643936000
F	3.693610000	12.264029000	6.217224000
F	1.838212000	10.698068000	5.022840000
F	1.938116000	8.053892000	5.241380000
P	6.121111000	8.217147000	8.250145000
C	3.847782000	8.124565000	6.645139000
C	4.797614000	8.962673000	7.265832000
C	4.739786000	10.343582000	7.103561000
C	3.743994000	10.942090000	6.360092000
C	2.801726000	10.137602000	5.745245000
C	2.881524000	8.764629000	5.887719000
C	6.290497000	9.271524000	9.728002000
C	7.528305000	9.671755000	10.220639000
H	8.437068000	9.416151000	9.692011000
C	7.607744000	10.420568000	11.386367000
H	8.578044000	10.730517000	11.755670000
C	6.455469000	10.779221000	12.069425000
H	6.520368000	11.367314000	12.976829000
C	5.217223000	10.386938000	11.578684000
H	4.309707000	10.670943000	12.097984000
C	5.135301000	9.635238000	10.418302000
H	4.163284000	9.351040000	10.036170000
C	7.653624000	8.440349000	7.302170000
C	7.697775000	9.130531000	6.095314000
H	6.801205000	9.579733000	5.688629000
C	8.889008000	9.225709000	5.391397000
H	8.911143000	9.758850000	4.448676000
C	10.043710000	8.637500000	5.886645000
H	10.972018000	8.713279000	5.333382000
C	10.003378000	7.938691000	7.085271000
H	10.897180000	7.463515000	7.470689000
C	8.811826000	7.829621000	7.782556000
H	8.777699000	7.258374000	8.703231000

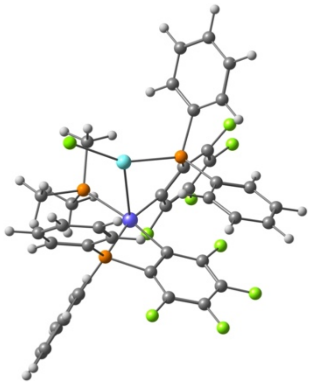

Figure S113. Optimized molecular structure of *cis*-**6NiAg**.

PBE0:

final single point energy: -10699.975089318632 a.u.

final Gibbs free energy: -10699.46924177 a.u.

Table S46. Atomic coordinates for optimized structure of *cis*-**6NiAg**.

Ni	-0.605096000	0.233899000	1.075724000
Ag	0.646461000	-2.099647000	-0.267533000
Cl	-0.064771000	-4.335302000	-0.084102000
P	-2.504713000	-0.314320000	0.045875000
P	2.159452000	-0.310210000	-0.441775000
P	-0.503853000	-1.207087000	2.745725000
F	-3.673122000	1.226358000	-2.715057000
F	-2.493170000	3.515147000	-3.593743000
F	-0.419167000	4.555245000	-2.258426000
F	0.598022000	3.383400000	-0.105927000
F	4.559145000	1.283536000	0.760880000
F	4.491977000	2.968536000	2.824023000
F	2.151446000	3.505214000	4.067016000
F	-0.120510000	2.358802000	3.253266000
C	-0.945258000	1.569099000	-0.273873000
C	-2.060772000	1.080640000	-0.975577000
C	-2.606156000	1.709632000	-2.076766000
C	-2.024865000	2.879775000	-2.523464000
C	-0.943517000	3.410806000	-1.830631000
C	-0.424276000	2.770381000	-0.716294000
C	1.011625000	1.052087000	1.637521000
C	2.233096000	0.798013000	0.988317000
C	3.390599000	1.459343000	1.384229000
C	3.376118000	2.355195000	2.435404000
C	2.176486000	2.629491000	3.068140000
C	1.024123000	1.994472000	2.643970000
C	1.075413000	-2.039675000	3.086147000
H	1.012148000	-2.565264000	4.040554000
H	1.873470000	-1.298360000	3.128025000
H	1.300145000	-2.760604000	2.299432000
C	-1.707517000	-2.564248000	2.728883000
H	-1.592184000	-3.180220000	3.622456000
H	-1.554835000	-3.178759000	1.841032000
H	-2.714101000	-2.143647000	2.709519000

C	-0.847088000	-0.414985000	4.347545000
H	-1.799894000	0.111426000	4.314740000
H	-0.060806000	0.297651000	4.588174000
H	-0.885491000	-1.185881000	5.119412000
C	-4.080529000	0.078642000	0.844319000
C	-4.040252000	0.723919000	2.077540000
H	-3.082021000	0.933630000	2.539435000
C	-5.216235000	1.108050000	2.703423000
H	-5.177636000	1.609614000	3.662752000
C	-6.437829000	0.845673000	2.099856000
H	-7.358202000	1.141057000	2.589193000
C	-6.482533000	0.205010000	0.867910000
H	-7.436030000	0.004022000	0.394593000
C	-5.308980000	-0.177546000	0.238326000
H	-5.348543000	-0.674027000	-0.723374000
C	-2.814313000	-1.757793000	-0.993694000
C	-3.538165000	-2.845019000	-0.508819000
H	-3.958012000	-2.819747000	0.489465000
C	-3.730962000	-3.960650000	-1.306298000
H	-4.297350000	-4.801189000	-0.924190000
C	-3.196807000	-4.004862000	-2.586983000
H	-3.347518000	-4.880201000	-3.206992000
C	-2.467164000	-2.928521000	-3.070175000
H	-2.047982000	-2.957617000	-4.068559000
C	-2.275655000	-1.807761000	-2.277414000
H	-1.716458000	-0.963714000	-2.662491000
C	1.953274000	0.739411000	-1.900454000
C	2.604414000	1.963239000	-2.032735000
H	3.257416000	2.325158000	-1.248901000
C	2.404844000	2.733310000	-3.166931000
H	2.905184000	3.689299000	-3.261153000
C	1.558419000	2.287791000	-4.174075000
H	1.396303000	2.899008000	-5.053720000
C	0.914466000	1.065193000	-4.050330000
H	0.250521000	0.715293000	-4.831405000
C	1.111178000	0.294342000	-2.915383000
H	0.601674000	-0.656776000	-2.809625000
C	3.796718000	-1.076753000	-0.622427000
C	4.270564000	-1.865097000	0.426201000
H	3.686595000	-1.968604000	1.334342000
C	5.487011000	-2.515058000	0.313559000
H	5.852297000	-3.118588000	1.135710000
C	6.232374000	-2.400633000	-0.854122000
H	7.181864000	-2.914399000	-0.943919000
C	5.755474000	-1.632011000	-1.904775000
H	6.330782000	-1.542508000	-2.818469000
C	4.540860000	-0.968897000	-1.791695000
H	4.178453000	-0.365266000	-2.614229000

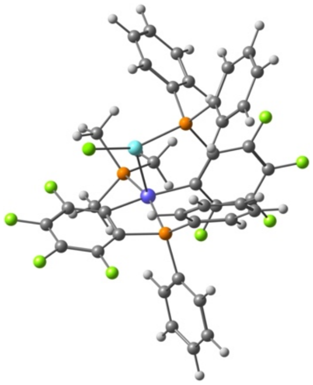

Figure S114. Optimized molecular structure of *trans*-**6NiAg**.

PBE0:

final single point energy: -10699.974915513481 a.u.

final Gibbs free energy: -10699.46983788 a.u.

Table S47. Atomic coordinates for optimized structure of *trans*-**6NiAg**.

Ni	0.553907000	-0.316949000	-1.082840000
Ag	-0.152147000	-1.410074000	1.410215000
Cl	1.136096000	-2.763931000	2.869455000
P	1.840498000	1.031721000	0.065368000
P	-2.098519000	-0.262633000	0.709997000
P	-0.209466000	-1.653017000	-2.632214000
F	4.621244000	0.155959000	1.742867000
F	5.522145000	-2.407221000	1.639255000
F	4.196214000	-4.238873000	0.200732000
F	1.993624000	-3.646282000	-1.139295000
F	-3.940615000	2.167142000	0.117642000
F	-3.596803000	4.039323000	-1.752186000
F	-1.469242000	3.920187000	-3.417972000
F	0.315404000	1.943850000	-3.215559000
C	2.066468000	-1.371725000	-0.427631000
C	2.793795000	-0.444571000	0.353576000
C	3.952597000	-0.753204000	1.033642000
C	4.425913000	-2.049608000	0.981507000
C	3.736687000	-2.993279000	0.231752000
C	2.587568000	-2.650427000	-0.460486000
C	-0.804784000	0.991324000	-1.362755000
C	-1.920407000	1.068124000	-0.507664000
C	-2.851764000	2.091074000	-0.650995000
C	-2.701666000	3.062928000	-1.620302000
C	-1.615688000	2.994797000	-2.476090000
C	-0.705737000	1.963390000	-2.337717000
C	-1.465775000	-0.975242000	-3.754128000
H	-1.070253000	-0.092368000	-4.255447000
H	-2.353418000	-0.692127000	-3.191334000
H	-1.732525000	-1.725885000	-4.500323000
C	-0.958492000	-3.227723000	-2.130474000
H	-1.132166000	-3.848911000	-3.011215000
H	-1.911117000	-3.022430000	-1.643471000
H	-0.311190000	-3.750891000	-1.431353000

C	1.112475000	-2.098189000	-3.795934000
H	0.709902000	-2.719886000	-4.598151000
H	1.905662000	-2.634024000	-3.280482000
H	1.523446000	-1.181945000	-4.221747000
C	1.343531000	1.876201000	1.574818000
C	0.557158000	3.023497000	1.453119000
H	0.265218000	3.381755000	0.472175000
C	0.159638000	3.713379000	2.585795000
H	-0.446149000	4.605689000	2.486408000
C	0.531472000	3.257455000	3.844399000
H	0.219890000	3.798893000	4.729351000
C	1.286596000	2.100470000	3.969214000
H	1.564003000	1.733399000	4.949633000
C	1.693517000	1.407002000	2.837727000
H	2.281811000	0.504283000	2.943152000
C	2.893618000	2.198576000	-0.832651000
C	3.228804000	1.880628000	-2.147957000
H	2.816041000	0.989147000	-2.606139000
C	4.080396000	2.702467000	-2.866384000
H	4.336983000	2.450371000	-3.888174000
C	4.595461000	3.851480000	-2.279751000
H	5.256729000	4.497964000	-2.844116000
C	4.261954000	4.172163000	-0.971718000
H	4.664551000	5.066155000	-0.510982000
C	3.414989000	3.347780000	-0.245339000
H	3.165144000	3.599984000	0.777397000
C	-2.932233000	0.405206000	2.181082000
C	-4.291557000	0.226966000	2.421752000
H	-4.906751000	-0.304083000	1.706322000
C	-4.863036000	0.736306000	3.577311000
H	-5.921352000	0.593213000	3.759799000
C	-4.085001000	1.429449000	4.495025000
H	-4.535175000	1.826706000	5.396882000
C	-2.728265000	1.604088000	4.259950000
H	-2.112947000	2.135948000	4.975396000
C	-2.151574000	1.083772000	3.112684000
H	-1.087885000	1.199766000	2.943337000
C	-3.309903000	-1.411632000	0.001299000
C	-3.423323000	-2.672108000	0.588519000
H	-2.795745000	-2.932026000	1.434619000
C	-4.323625000	-3.596957000	0.087938000
H	-4.403483000	-4.573595000	0.549454000
C	-5.108194000	-3.277308000	-1.013461000
H	-5.802797000	-4.005918000	-1.413636000
C	-4.997715000	-2.026530000	-1.602053000
H	-5.607235000	-1.772854000	-2.460899000
C	-4.105712000	-1.092576000	-1.093368000
H	-4.028578000	-0.118348000	-1.559493000

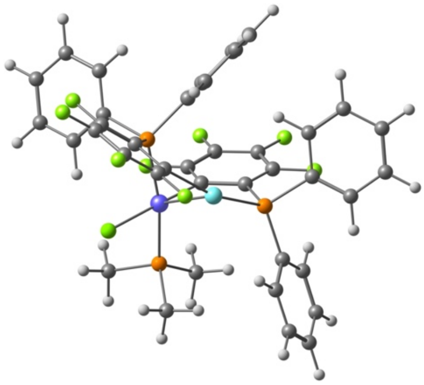

Figure S115. Optimized molecular structure of *cis*-*trans*-**7NiAg**.

PBE0:

final single point energy: -10699.973300103266 a.u.

final Gibbs free energy: -10699.46683097 a.u.

Table S48. Atomic coordinates for optimized structure of *cis*-*trans*-**7NiAg**.

Ni	-0.388486000	0.851213000	-1.311402000
Ag	0.117661000	-1.793950000	-0.370605000
P	2.193715000	-0.800541000	0.116367000
Cl	-1.787453000	0.089333000	-2.842452000
C	1.934488000	0.959278000	0.492709000
F	-1.763743000	-4.544385000	-0.875914000
C	3.508668000	-0.902744000	-1.122853000
C	-4.164217000	-1.344938000	-0.020035000
C	0.699317000	2.965568000	0.180780000
C	2.593976000	2.992897000	1.629845000
C	0.840652000	1.618883000	-0.103302000
C	2.791234000	1.658219000	1.338585000
C	-2.793056000	-1.165466000	-0.056593000
C	-2.520278000	-3.462729000	-0.595548000
C	-4.718003000	-2.587543000	-0.290400000
C	-1.940972000	-2.244683000	-0.356408000
F	3.417066000	3.641081000	2.449049000
F	-5.020040000	-0.361010000	0.270967000
F	-0.295979000	3.675268000	-0.381591000
F	3.858936000	1.079333000	1.893983000
F	-6.039249000	-2.754142000	-0.269055000
F	1.340363000	4.943430000	1.296813000
F	-4.432400000	-4.845461000	-0.842122000
C	1.535590000	3.656619000	1.036415000
P	-1.911015000	0.390083000	0.288752000
C	-1.404265000	0.185008000	2.032064000
C	-3.088147000	1.777748000	0.383000000
P	0.817837000	1.613496000	-2.984525000
C	2.883702000	-1.549932000	1.617732000
C	-3.893208000	-3.656756000	-0.579909000
C	-3.295435000	2.553652000	-0.754810000
H	-2.767974000	2.313788000	-1.669605000
C	-4.174532000	3.625440000	-0.719898000
H	-4.328577000	4.222336000	-1.610835000

C	-4.846597000	3.935974000	0.453126000
H	-5.528834000	4.777280000	0.482388000
C	-4.640225000	3.168471000	1.592124000
H	-5.162840000	3.406461000	2.510884000
C	-3.765926000	2.094669000	1.558684000
H	-3.615134000	1.500193000	2.450674000
C	-1.598626000	-1.006530000	2.726717000
H	-2.057864000	-1.852244000	2.233479000
C	-1.209673000	-1.118222000	4.055006000
H	-1.370778000	-2.051655000	4.580623000
C	-0.617553000	-0.045730000	4.703194000
H	-0.310678000	-0.135622000	5.738280000
C	-0.420270000	1.146369000	4.017911000
H	0.036582000	1.993404000	4.515383000
C	-0.814019000	1.261660000	2.695391000
H	-0.680668000	2.205518000	2.185439000
C	3.397960000	-1.888389000	-2.100783000
H	2.531130000	-2.540365000	-2.109811000
C	4.383887000	-2.030553000	-3.066143000
H	4.288566000	-2.798510000	-3.823939000
C	5.479861000	-1.180571000	-3.065485000
H	6.245765000	-1.283304000	-3.824724000
C	5.594741000	-0.194723000	-2.093520000
C	4.616988000	-0.057476000	-1.121665000
C	4.056778000	-2.295416000	1.607776000
H	4.632605000	-2.396509000	0.696125000
C	4.496669000	-2.907344000	2.773858000
H	5.412699000	-3.485770000	2.761705000
C	3.771953000	-2.775743000	3.949128000
H	4.120330000	-3.251759000	4.857787000
C	2.596285000	-2.034597000	3.957733000
H	2.022353000	-1.929550000	4.870692000
C	2.148541000	-1.431427000	2.795761000
H	1.226626000	-0.861726000	2.804438000
H	6.449719000	0.470407000	-2.092141000
H	4.720722000	0.710510000	-0.366103000
C	2.384378000	2.471507000	-2.665193000
H	2.828367000	2.770388000	-3.616244000
H	2.207617000	3.357546000	-2.056691000
H	3.072042000	1.813166000	-2.137434000
C	1.288587000	0.376562000	-4.221019000
H	0.400366000	-0.156886000	-4.552845000
H	1.770859000	0.870071000	-5.066692000
H	1.985411000	-0.329735000	-3.770178000
C	-0.123395000	2.864335000	-3.901314000
H	0.472073000	3.235705000	-4.737389000
H	-1.048131000	2.422015000	-4.266427000
H	-0.362468000	3.690065000	-3.230623000

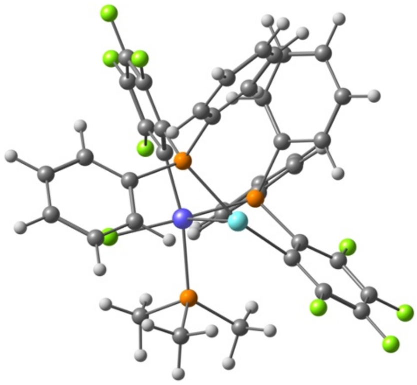

Figure S116. Optimized molecular structure of *cis*-*cis*-**7NiAg**.

PBE0:

final single point energy: -10699.967292936421 a.u.

final Gibbs free energy: -10699.45993004 a.u.

Table S49. Atomic coordinates for optimized structure of *cis*-*cis*-**7NiAg**.

Ni	0.423267000	0.304707000	-1.541143000
Ag	-0.343349000	-1.703419000	0.243126000
Cl	-0.956939000	0.035883000	-3.240220000
P	-2.414520000	-0.586783000	0.142349000
P	1.742266000	0.592437000	0.179450000
F	-4.187336000	1.845576000	0.712528000
F	-3.701124000	4.406734000	0.157788000
F	-1.421453000	5.108614000	-1.120758000
F	0.378771000	3.268061000	-1.804406000
F	4.736163000	-0.111723000	1.088295000
F	5.670364000	-2.533987000	1.621702000
F	4.063418000	-4.698116000	1.494862000
F	1.466032000	-4.427751000	0.864979000
C	-0.891962000	1.518453000	-0.842132000
C	-2.091159000	1.165977000	-0.196279000
C	-3.023144000	2.146939000	0.129310000
C	-2.802786000	3.478321000	-0.161977000
C	-1.640707000	3.832993000	-0.821918000
C	-0.724993000	2.852563000	-1.154957000
C	1.699458000	-2.094359000	0.578728000
C	2.555797000	-0.978580000	0.629098000
C	3.880117000	-1.132347000	1.004350000
C	4.389824000	-2.388382000	1.292459000
C	3.564395000	-3.495468000	1.227435000
C	2.232625000	-3.320068000	0.886491000
C	-3.616500000	-1.180948000	-1.071572000
C	-3.605896000	-2.543077000	-1.364004000
H	-2.879567000	-3.191055000	-0.884838000
C	-4.512518000	-3.070469000	-2.270481000
H	-4.498159000	-4.130755000	-2.491771000
C	-5.424122000	-2.236300000	-2.901689000
H	-6.126367000	-2.644459000	-3.618603000
C	-5.429603000	-0.876303000	-2.621860000
H	-6.134475000	-0.221701000	-3.119951000

C	-4.531769000	-0.348101000	-1.707709000
H	-4.541735000	0.714462000	-1.504686000
C	-3.268641000	-0.639253000	1.743658000
C	-4.444776000	-1.353842000	1.939728000
H	-4.924553000	-1.853763000	1.107324000
C	-5.011898000	-1.418884000	3.205433000
H	-5.930254000	-1.974774000	3.352285000
C	-4.411709000	-0.771666000	4.275497000
H	-4.859187000	-0.822012000	5.260852000
C	-3.234918000	-0.058657000	4.080139000
H	-2.759073000	0.449902000	4.910283000
C	-2.662208000	0.002140000	2.821836000
H	-1.742891000	0.556068000	2.674031000
C	2.982799000	1.884605000	-0.140068000
C	3.257829000	2.234215000	-1.457729000
H	2.716208000	1.755338000	-2.263487000
C	4.193865000	3.215828000	-1.746349000
H	4.395943000	3.480042000	-2.777168000
C	4.852661000	3.865929000	-0.714234000
H	5.579301000	4.638550000	-0.935396000
C	4.570921000	3.534464000	0.605701000
H	5.078074000	4.046036000	1.414797000
C	3.640424000	2.551070000	0.893073000
H	3.429121000	2.298247000	1.924184000
C	1.043797000	1.125737000	1.781963000
C	0.532100000	2.417902000	1.905508000
H	0.548208000	3.093020000	1.061079000
C	0.025709000	2.861091000	3.115239000
H	-0.368848000	3.866943000	3.192729000
C	0.034635000	2.024758000	4.224780000
H	-0.358416000	2.373900000	5.171976000
C	0.549632000	0.743435000	4.113154000
H	0.562922000	0.083438000	4.971957000
C	1.047859000	0.292831000	2.897731000
H	1.445397000	-0.710337000	2.827124000
P	1.851853000	-0.954836000	-2.752870000
C	3.565564000	-1.370400000	-2.294001000
H	4.109472000	-0.487274000	-1.959372000
H	3.575608000	-2.117936000	-1.504430000
H	4.061483000	-1.779899000	-3.175763000
C	1.172761000	-2.597972000	-3.116232000
H	1.819008000	-3.116330000	-3.826767000
H	1.122820000	-3.167574000	-2.187655000
H	0.171101000	-2.491906000	-3.527718000
C	2.127604000	-0.203709000	-4.387336000
H	2.701361000	-0.892892000	-5.009190000
H	1.172048000	0.019249000	-4.855099000
H	2.693194000	0.721941000	-4.272625000

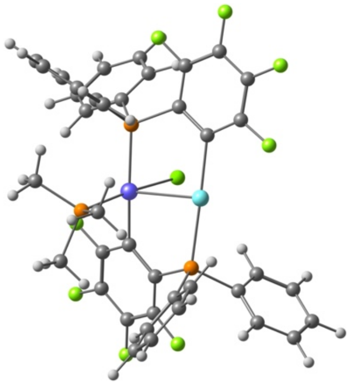

Figure S117. Optimized molecular structure of *trans*-**7NiAg**.

PBE0:

final single point energy: -10699.964625573088 a.u.

final Gibbs free energy: -10699.45799338 a.u.

Table S50. Atomic coordinates for optimized structure of *trans*-**7NiAg**.

Ni	-0.240463000	-1.120655000	-0.110737000
Ag	0.288332000	1.576798000	-0.291463000
Cl	-0.235659000	-0.703124000	-2.300698000
P	2.520845000	0.858869000	-0.011689000
P	-2.434477000	-0.531284000	-0.156107000
P	-0.051593000	-1.614208000	1.993601000
F	5.053943000	-0.744577000	-0.611728000
F	5.269008000	-3.347478000	-1.175530000
F	3.067176000	-4.919705000	-1.180317000
F	0.658865000	-3.896143000	-0.616124000
F	-5.108092000	0.766753000	-0.797174000
F	-5.633116000	3.337005000	-1.248565000
F	-3.645647000	5.166173000	-1.222687000
F	-1.116698000	4.419273000	-0.755732000
C	1.547738000	-1.723891000	-0.341051000
C	2.697042000	-0.917307000	-0.338633000
C	3.938884000	-1.479815000	-0.621653000
C	4.075497000	-2.822406000	-0.910125000
C	2.947129000	-3.625198000	-0.910319000
C	1.717957000	-3.064613000	-0.621919000
C	-1.724021000	2.140127000	-0.574900000
C	-2.767408000	1.199484000	-0.612505000
C	-4.077601000	1.618952000	-0.821102000
C	-4.377641000	2.949792000	-1.037472000
C	-3.357364000	3.883863000	-1.019944000
C	-2.063423000	3.457190000	-0.782418000
C	1.461164000	-2.452174000	2.547926000
H	1.405388000	-2.574098000	3.631105000
H	2.342507000	-1.868498000	2.292683000
H	1.536896000	-3.432662000	2.079419000
C	-1.304832000	-2.733593000	2.676888000
H	-1.040970000	-2.969085000	3.709272000
H	-1.315097000	-3.651979000	2.088758000
H	-2.293195000	-2.284393000	2.649081000

C	-0.076306000	-0.153129000	3.066351000
H	-0.974837000	0.433258000	2.893365000
H	0.794699000	0.462764000	2.841658000
H	-0.037998000	-0.467571000	4.110960000
C	3.610655000	1.682584000	-1.207667000
C	4.684426000	2.479438000	-0.829021000
H	4.932861000	2.597956000	0.218442000
C	5.447057000	3.117553000	-1.797202000
H	6.284310000	3.736584000	-1.497675000
C	5.144710000	2.958169000	-3.141601000
H	5.744346000	3.454603000	-3.895030000
C	4.069723000	2.163622000	-3.521037000
H	3.827565000	2.039355000	-4.569617000
C	3.298308000	1.533785000	-2.558810000
H	2.450908000	0.923150000	-2.854273000
C	3.235771000	1.185791000	1.621785000
C	4.091901000	0.307216000	2.279005000
H	4.385533000	-0.624785000	1.814402000
C	4.570755000	0.616208000	3.543741000
H	5.230404000	-0.077643000	4.050639000
C	4.208634000	1.807520000	4.155734000
H	4.584267000	2.045857000	5.143524000
C	3.358165000	2.690587000	3.503746000
H	3.067361000	3.619432000	3.979137000
C	2.865374000	2.376589000	2.247688000
H	2.180263000	3.054648000	1.749465000
C	-3.146407000	-1.659539000	-1.386583000
C	-2.948844000	-3.023636000	-1.172232000
H	-2.426813000	-3.363619000	-0.284188000
C	-3.395466000	-3.951143000	-2.099268000
H	-3.238293000	-5.008020000	-1.920849000
C	-4.026487000	-3.522863000	-3.259120000
H	-4.369048000	-4.245770000	-3.989674000
C	-4.203050000	-2.165744000	-3.487372000
H	-4.678993000	-1.825313000	-4.399102000
C	-3.764149000	-1.235671000	-2.556752000
H	-3.886419000	-0.180612000	-2.760949000
C	-3.561595000	-0.672496000	1.281140000
C	-4.450234000	-1.729558000	1.453855000
H	-4.533392000	-2.500804000	0.699939000
C	-5.240077000	-1.802630000	2.592209000
H	-5.928541000	-2.630609000	2.711706000
C	-5.154022000	-0.821673000	3.569097000
H	-5.769978000	-0.882304000	4.458141000
C	-4.285997000	0.247419000	3.393407000
H	-4.224798000	1.029279000	4.140878000
C	-3.500364000	0.324095000	2.255048000
H	-2.846924000	1.177377000	2.114483000

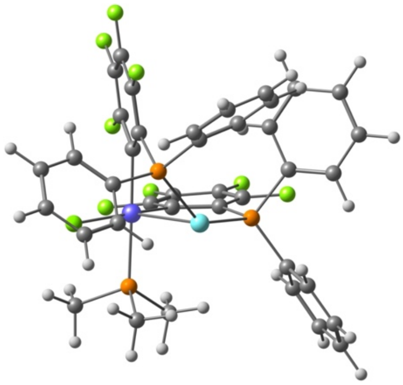

Figure S118. Optimized molecular structure of *cis*-**8NiAg**.

PBE0:

final single point energy: -10699.955875622691 a.u.

final Gibbs free energy: -10699.45111866 a.u.

Table S51. Atomic coordinates for optimized structure of *cis*-**8NiAg**.

Ni	-0.171869000	-1.848167000	-0.325146000
Ag	0.143559000	0.675851000	-1.290427000
P	-1.935651000	1.234428000	-0.284694000
Cl	1.595652000	-2.777958000	-1.293257000
C	-2.503593000	-0.203002000	0.643068000
F	-0.157440000	-2.644407000	2.706996000
C	-3.243387000	1.782771000	-1.414537000
C	3.018470000	-0.205280000	2.048960000
C	-2.422272000	-2.508778000	1.153882000
C	-4.184919000	-1.184563000	2.079185000
C	-1.812551000	-1.424901000	0.536081000
C	-3.645565000	-0.094072000	1.436081000
C	2.130744000	-0.416699000	1.001025000
C	0.850607000	-1.801271000	2.419054000
C	2.818398000	-0.764475000	3.294946000
C	0.992285000	-1.251559000	1.158308000
F	-5.280824000	-1.075298000	2.826183000
F	4.120716000	0.539461000	1.899579000
F	-1.902978000	-3.741913000	1.022287000
F	-4.264393000	1.081468000	1.595036000
F	3.672884000	-0.545782000	4.292028000
F	-4.091904000	-3.485767000	2.502197000
F	1.505993000	-2.132471000	4.665067000
C	-3.569371000	-2.415168000	1.914946000
P	2.395734000	0.470463000	-0.566540000
C	3.773427000	-0.257713000	-1.479273000
C	2.980280000	2.134856000	-0.106071000
P	-1.313898000	-2.464771000	-2.113945000
C	-1.727480000	2.533687000	0.968658000
C	1.715734000	-1.570870000	3.477849000
C	2.125819000	2.935291000	0.651116000
H	1.168225000	2.547908000	0.979826000
C	2.492148000	4.225210000	0.994451000
H	1.820622000	4.830977000	1.591299000

C	3.710610000	4.738567000	0.566759000
H	3.996522000	5.750313000	0.828233000
C	4.558304000	3.951025000	-0.197944000
H	5.510337000	4.344777000	-0.533677000
C	4.199079000	2.651816000	-0.531220000
H	4.875207000	2.041061000	-1.116023000
C	4.738694000	-1.060671000	-0.882077000
H	4.675122000	-1.301522000	0.171037000
C	5.776743000	-1.575421000	-1.642961000
H	6.522229000	-2.206941000	-1.174934000
C	5.858876000	-1.288811000	-2.998997000
H	6.670142000	-1.695719000	-3.590622000
C	4.895521000	-0.489057000	-3.598174000
H	4.950389000	-0.269766000	-4.657658000
C	3.851749000	0.020226000	-2.841534000
H	3.087723000	0.630215000	-3.311720000
C	-2.869895000	2.719910000	-2.380165000
H	-1.849309000	3.087642000	-2.408311000
C	-3.789757000	3.175528000	-3.310096000
H	-3.489697000	3.909372000	-4.048159000
C	-5.085245000	2.676338000	-3.305576000
H	-5.802922000	3.021778000	-4.039788000
C	-5.454170000	1.723438000	-2.367565000
C	-4.541182000	1.279836000	-1.422041000
C	-2.077203000	3.861052000	0.748096000
H	-2.571474000	4.151968000	-0.170415000
C	-1.802415000	4.819609000	1.713318000
H	-2.082591000	5.851198000	1.536956000
C	-1.178057000	4.461076000	2.899173000
H	-0.965608000	5.212164000	3.650253000
C	-0.828403000	3.135521000	3.121857000
H	-0.340853000	2.847135000	4.045289000
C	-1.097693000	2.174967000	2.161071000
H	-0.814845000	1.142434000	2.335904000
H	-6.459416000	1.319760000	-2.368356000
H	-4.847218000	0.530265000	-0.705567000
C	-3.081661000	-2.069938000	-2.251962000
H	-3.471846000	-2.461269000	-3.193015000
H	-3.632166000	-2.508963000	-1.420468000
H	-3.221941000	-0.989861000	-2.229028000
C	-0.657904000	-1.757378000	-3.654732000
H	0.407248000	-1.969953000	-3.724043000
H	-1.182986000	-2.177603000	-4.514626000
H	-0.809438000	-0.675150000	-3.646702000
C	-1.294613000	-4.256994000	-2.397297000
H	-1.820708000	-4.494962000	-3.323535000
H	-0.262709000	-4.598653000	-2.453011000
H	-1.788149000	-4.752182000	-1.560702000

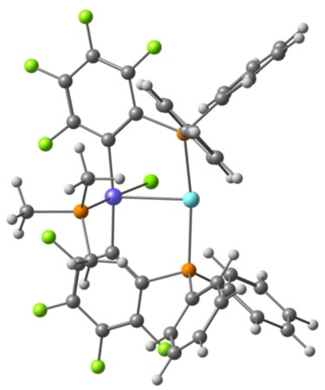

Figure S119. Optimized molecular structure of *trans*-**8NiAg**.

PBE0:

final single point energy: -10699.973700896395 a.u.

final Gibbs free energy: -10699.46739475 a.u.

Table S52. Atomic coordinates for optimized structure of *trans*-**8NiAg**.

Ni	0.000010000	1.587877000	-0.189053000
Ag	0.000090000	-1.073414000	-0.470744000
Cl	0.000357000	1.113519000	-2.387031000
F	-5.178476000	0.005608000	-0.353833000
F	-6.004030000	2.532391000	-0.619918000
F	-4.195219000	4.547581000	-0.651530000
F	-1.594132000	4.049107000	-0.419006000
P	-2.348526000	-1.043209000	-0.130731000
P	-0.000335000	1.902042000	1.933252000
C	-1.937288000	1.713964000	-0.222385000
C	-2.899103000	0.682080000	-0.212206000
C	-4.253270000	0.972671000	-0.352219000
C	-4.705484000	2.267747000	-0.493720000
C	-3.778362000	3.294340000	-0.506199000
C	-2.434895000	2.995726000	-0.373823000
C	-2.871983000	-1.768128000	1.446490000
C	-2.146332000	-2.869263000	1.901036000
H	-1.317276000	-3.245281000	1.310791000
C	-2.469108000	-3.475766000	3.104351000
H	-1.899724000	-4.331767000	3.445857000
C	-3.508684000	-2.973793000	3.875094000
H	-3.755275000	-3.438047000	4.822379000
C	-4.225369000	-1.869519000	3.435305000
H	-5.031893000	-1.469772000	4.038025000
C	-3.913210000	-1.269527000	2.224683000
H	-4.476911000	-0.405837000	1.900386000
C	-3.283065000	-1.913868000	-1.419847000
C	-4.090172000	-3.013462000	-1.152864000
H	-4.222291000	-3.358049000	-0.134538000
C	-4.737031000	-3.665708000	-2.193378000
H	-5.367704000	-4.520524000	-1.980059000
C	-4.584167000	-3.221535000	-3.498535000
H	-5.093862000	-3.729922000	-4.308111000
C	-3.774680000	-2.124252000	-3.766655000

H	-3.649958000	-1.775270000	-4.784694000
C	-3.118952000	-1.475509000	-2.733844000
H	-2.477193000	-0.625689000	-2.944201000
C	0.000026000	3.657338000	2.392446000
H	-0.000231000	3.746787000	3.480318000
H	0.884888000	4.143382000	1.984642000
C	1.400778000	1.231837000	2.869122000
H	1.279667000	1.475195000	3.926063000
H	2.336055000	1.648422000	2.499746000
H	1.430243000	0.148965000	2.751806000
H	-0.884359000	4.143863000	1.984180000
C	-1.402155000	1.232572000	2.868599000
H	-1.281302000	1.475860000	3.925585000
H	-2.337083000	1.649640000	2.498883000
H	-1.432140000	0.149712000	2.751262000
F	5.178627000	0.005711000	-0.352302000
F	6.004177000	2.532509000	-0.618198000
F	4.195302000	4.547639000	-0.650425000
F	1.594172000	4.049090000	-0.418610000
P	2.348654000	-1.043224000	-0.130356000
C	1.937339000	1.713965000	-0.221798000
C	2.899186000	0.682100000	-0.211415000
C	4.253385000	0.972740000	-0.351001000
C	4.705601000	2.267829000	-0.492378000
C	3.778448000	3.294388000	-0.505166000
C	2.434953000	2.995736000	-0.373136000
C	2.871956000	-1.768456000	1.446768000
C	2.145835000	-2.869251000	1.901381000
H	1.316499000	-3.244835000	1.311254000
C	2.468484000	-3.475939000	3.104638000
H	1.898722000	-4.331663000	3.446210000
C	3.508424000	-2.974502000	3.875237000
H	3.754924000	-3.438900000	4.822476000
C	4.225607000	-1.870585000	3.435360000
H	5.032427000	-1.471263000	4.037965000
C	3.913567000	-1.270409000	2.224800000
H	4.477666000	-0.407006000	1.900423000
C	3.283377000	-1.913586000	-1.419536000
C	4.090401000	-3.013272000	-1.152689000
H	4.222355000	-3.358101000	-0.134422000
C	4.737395000	-3.665297000	-2.193257000
H	5.368007000	-4.520185000	-1.980043000
C	4.584746000	-3.220814000	-3.498333000
H	5.094545000	-3.729028000	-4.307951000
C	3.775336000	-2.123440000	-3.766319000
H	3.650781000	-1.774215000	-4.784295000
C	3.119475000	-1.474915000	-2.733456000
H	2.477776000	-0.625026000	-2.943711000

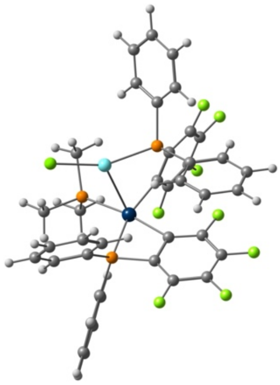

Figure S120. Optimized molecular structure of *cis*-**6PtAg**.

PBE0:

final single point energy: -28211.274926079856 a.u.

final Gibbs free energy: -28210.76973869 a.u.

Table S53. Atomic coordinates for optimized structure of *cis*-**6PtAg**.

Pt	3.424270000	8.701011000	4.104457000
Ag	0.745654000	9.198475000	2.806686000
Cl	0.352658000	10.860310000	1.187192000
P	4.440128000	8.454530000	2.041319000
P	0.283285000	7.668622000	4.538051000
P	3.234130000	10.974962000	4.392197000
F	5.179198000	5.534080000	0.504027000
F	4.901737000	3.183342000	1.838815000
F	4.045606000	3.166438000	4.375714000
F	3.383731000	5.412963000	5.650686000
F	-0.467385000	7.007733000	7.476323000
F	1.035912000	7.328006000	9.648891000
F	3.578201000	8.224486000	9.423795000
F	4.614624000	8.797864000	7.039157000
C	3.852744000	6.719435000	3.716871000
C	4.351854000	6.697697000	2.400900000
C	4.712090000	5.535182000	1.752920000
C	4.583837000	4.333706000	2.425626000
C	4.132285000	4.329709000	3.739131000
C	3.782418000	5.510354000	4.377436000
C	2.574731000	8.327728000	5.929435000
C	1.252755000	7.861542000	6.061869000
C	0.758523000	7.512442000	7.313502000
C	1.526569000	7.647329000	8.453886000
C	2.826391000	8.102242000	8.335742000
C	3.326665000	8.412645000	7.084426000
C	1.679584000	11.595550000	5.091481000
H	1.768923000	12.661418000	5.308053000
H	1.456500000	11.050534000	6.008917000
H	0.864197000	11.442750000	4.383382000
C	3.488334000	11.979773000	2.909271000
H	3.419598000	13.040777000	3.155471000
H	2.731746000	11.726495000	2.165371000
H	4.478205000	11.764291000	2.504646000

C	4.470655000	11.628926000	5.548953000
H	5.472018000	11.377749000	5.200652000
H	4.322863000	11.191178000	6.534477000
H	4.369386000	12.714019000	5.610794000
C	6.180296000	8.911159000	1.873003000
C	6.835684000	9.424992000	2.988173000
H	6.282276000	9.568023000	3.909347000
C	8.185147000	9.737300000	2.920616000
H	8.690338000	10.137928000	3.791060000
C	8.882534000	9.537029000	1.738127000
H	9.936323000	9.782698000	1.682858000
C	8.232249000	9.021154000	0.623577000
H	8.778163000	8.862041000	-0.298449000
C	6.884430000	8.707836000	0.687164000
H	6.380844000	8.306492000	-0.183881000
C	3.679546000	8.784181000	0.440558000
C	3.959696000	9.965150000	-0.243442000
H	4.668100000	10.676484000	0.163756000
C	3.338908000	10.228164000	-1.453246000
H	3.561694000	11.146759000	-1.982221000
C	2.433591000	9.319444000	-1.984523000
H	1.949333000	9.528184000	-2.930714000
C	2.147305000	8.145852000	-1.302238000
H	1.441083000	7.434811000	-1.713182000
C	2.766260000	7.877615000	-0.091551000
H	2.544694000	6.958046000	0.436637000
C	0.443539000	5.940225000	4.029361000
C	0.394967000	4.883805000	4.935899000
H	0.251832000	5.073669000	5.991979000
C	0.543617000	3.581222000	4.488642000
H	0.514404000	2.763587000	5.198410000
C	0.739716000	3.324051000	3.137622000
H	0.866227000	2.304293000	2.794451000
C	0.783022000	4.371985000	2.229869000
H	0.941634000	4.176986000	1.176236000
C	0.637612000	5.676113000	2.676051000
H	0.686619000	6.499581000	1.971990000
C	-1.458527000	7.905448000	4.992326000
C	-1.823359000	9.134068000	5.543074000
H	-1.066169000	9.885431000	5.741326000
C	-3.149142000	9.398267000	5.838326000
H	-3.423693000	10.351839000	6.273137000
C	-4.125696000	8.446658000	5.567900000
H	-5.164440000	8.656683000	5.792459000
C	-3.767713000	7.231236000	5.004863000
H	-4.525676000	6.487945000	4.788429000
C	-2.437040000	6.957343000	4.717843000
H	-2.164634000	6.003716000	4.283077000

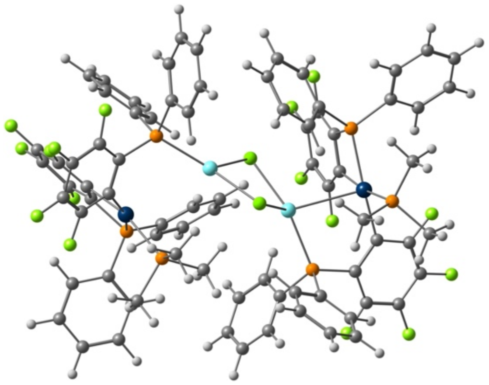

Figure S121. Optimized molecular structure of *cis*-**6PtAg-dimer** .

PBE0:

final single point energy: -56422.579491737335 a.u.

final Gibbs free energy: -56421.54251371 a.u.

Table S54. Atomic coordinates for optimized structure of *cis*-**6PtAg-dimer** .

Pt	4.166641000	-0.622988000	-0.315635000
Pt	-4.638041000	0.309075000	0.152566000
Ag	1.203989000	1.483872000	-0.678683000
Ag	-1.818371000	-0.280521000	-0.455327000
Cl	-0.481982000	1.030484000	1.195065000
Cl	-0.498408000	0.936831000	-2.467757000
P	3.889558000	-2.131575000	1.419388000
P	3.241606000	2.668897000	-0.474473000
P	2.909479000	-1.597690000	-1.961321000
P	-4.122884000	2.380257000	1.046404000
P	-2.782294000	-2.378398000	-1.104517000
P	-5.463063000	0.844690000	-1.923063000
F	5.587865000	-2.210183000	4.330620000
F	7.580448000	-0.422864000	4.806417000
F	8.244173000	1.379074000	2.936442000
F	6.997428000	1.494961000	0.592419000
F	5.364358000	4.412969000	-1.993551000
F	7.331656000	3.567829000	-3.544017000
F	7.840385000	0.919695000	-3.794477000
F	6.397775000	-0.869355000	-2.452297000
F	-2.982654000	2.984657000	4.193189000
F	-2.445867000	0.927774000	5.863690000
F	-2.883376000	-1.601651000	5.087747000
F	-3.850681000	-2.176556000	2.670225000
F	-4.239657000	-5.065644000	-0.871478000
F	-6.663860000	-5.546391000	0.131426000
F	-8.196889000	-3.496539000	1.012028000
F	-7.305637000	-0.990859000	0.918733000
C	5.494124000	-0.239776000	1.230246000
C	5.199825000	-1.201412000	2.216130000
C	5.882322000	-1.290033000	3.410923000
C	6.908054000	-0.396484000	3.657893000
C	7.247602000	0.535022000	2.686399000
C	6.563416000	0.595000000	1.481387000

C	4.986181000	0.802529000	-1.538763000
C	4.684011000	2.178055000	-1.475499000
C	5.508297000	3.091021000	-2.125239000
C	6.564791000	2.677844000	-2.915694000
C	6.827628000	1.328291000	-3.035798000
C	6.047671000	0.425737000	-2.337372000
C	2.596909000	-0.681954000	-3.491151000
H	2.147187000	-1.337673000	-4.238175000
H	3.541049000	-0.291784000	-3.869302000
H	1.918632000	0.146176000	-3.289051000
C	1.296641000	-2.264975000	-1.484947000
H	0.624647000	-1.451208000	-1.214381000
H	1.410172000	-2.923805000	-0.624771000
H	0.857160000	-2.829693000	-2.307209000
C	3.798711000	-3.072421000	-2.540715000
H	3.967678000	-3.750382000	-1.704917000
H	4.763419000	-2.774170000	-2.946995000
H	3.217618000	-3.581488000	-3.311225000
C	4.494770000	-3.795133000	1.053608000
C	3.592567000	-4.837243000	0.852433000
H	2.536249000	-4.682390000	1.032074000
C	4.042422000	-6.070969000	0.408584000
H	3.336345000	-6.877693000	0.255753000
C	5.392645000	-6.270679000	0.157806000
H	5.743542000	-7.234863000	-0.187615000
C	6.293644000	-5.232218000	0.352476000
H	7.346318000	-5.381353000	0.148750000
C	5.848868000	-3.998271000	0.798960000
H	6.552767000	-3.187499000	0.940634000
C	2.487432000	-2.351046000	2.528858000
C	1.372997000	-1.538490000	2.370422000
H	1.322539000	-0.826677000	1.557019000
C	0.314304000	-1.629228000	3.261743000
H	-0.535184000	-0.972731000	3.126255000
C	0.359918000	-2.545583000	4.300212000
H	-0.471653000	-2.625495000	4.988813000
C	1.470762000	-3.368920000	4.456236000
H	1.507365000	-4.084398000	5.268061000
C	2.535292000	-3.271583000	3.577532000
H	3.404529000	-3.903043000	3.710141000
C	3.855939000	2.799852000	1.223588000
C	3.173040000	2.117037000	2.225864000
H	2.271537000	1.566984000	1.982047000
C	3.650178000	2.129501000	3.527576000
H	3.122829000	1.581357000	4.298012000
C	4.805723000	2.831527000	3.834416000
H	5.186779000	2.831855000	4.848168000
C	5.478812000	3.534029000	2.841733000
H	6.380739000	4.082977000	3.081044000
C	5.009809000	3.514523000	1.539257000
H	5.555693000	4.036741000	0.763618000

C	2.824540000	4.365068000	-0.990542000
C	2.916141000	5.466893000	-0.148752000
H	3.258446000	5.345650000	0.870053000
C	2.570443000	6.729485000	-0.611992000
H	2.652712000	7.582962000	0.049466000
C	2.125320000	6.899069000	-1.914294000
H	1.863807000	7.885627000	-2.275795000
C	2.003571000	5.795199000	-2.750560000
H	1.635964000	5.916261000	-3.761829000
C	2.341786000	4.535932000	-2.288236000
H	2.234536000	3.676433000	-2.940950000
C	-3.923846000	0.129928000	2.084840000
C	-3.684199000	1.446587000	2.521437000
C	-3.203375000	1.736607000	3.779240000
C	-2.928571000	0.694288000	4.646469000
C	-3.158253000	-0.612644000	4.240574000
C	-3.658781000	-0.887138000	2.976521000
C	-5.222535000	-1.655297000	0.001810000
C	-4.441248000	-2.729385000	-0.457740000
C	-4.942019000	-4.026765000	-0.410850000
C	-6.199051000	-4.299859000	0.089410000
C	-6.982462000	-3.249675000	0.533268000
C	-6.487952000	-1.961086000	0.471763000
C	-4.288897000	0.746912000	-3.300892000
H	-4.012449000	-0.293478000	-3.462528000
H	-3.378525000	1.299249000	-3.072496000
H	-4.744181000	1.144446000	-4.208970000
C	-6.108556000	2.536677000	-2.026239000
H	-5.296625000	3.242703000	-1.851324000
H	-6.865613000	2.687628000	-1.257331000
H	-6.541295000	2.723698000	-3.009551000
C	-6.848773000	-0.173386000	-2.498368000
H	-7.655586000	-0.141201000	-1.767109000
H	-6.513774000	-1.205003000	-2.600832000
H	-7.213521000	0.187797000	-3.460343000
C	-5.555514000	3.417825000	1.427583000
C	-5.619756000	4.759507000	1.066811000
H	-4.767809000	5.237908000	0.601300000
C	-6.776669000	5.487947000	1.307017000
H	-6.823959000	6.529782000	1.016252000
C	-7.867631000	4.883852000	1.913188000
H	-8.768675000	5.454414000	2.100243000
C	-7.806423000	3.543322000	2.275310000
H	-8.654788000	3.070052000	2.753429000
C	-6.659932000	2.809628000	2.025587000
H	-6.621204000	1.757490000	2.282487000
C	-2.811620000	3.488786000	0.518470000
C	-2.332274000	3.370184000	-0.778957000
H	-2.733312000	2.608025000	-1.432585000
C	-1.295067000	4.177992000	-1.216077000
H	-0.911369000	4.061884000	-2.220689000

C	-0.729996000	5.100035000	-0.351544000
H	0.089991000	5.719894000	-0.686875000
C	-1.203400000	5.219656000	0.949497000
H	-0.743824000	5.925001000	1.630461000
C	-2.247492000	4.424464000	1.385349000
H	-2.614469000	4.522365000	2.397896000
C	-1.703281000	-3.738500000	-0.558386000
C	-1.263971000	-4.750907000	-1.405078000
H	-1.599214000	-4.780843000	-2.433807000
C	-0.401399000	-5.727284000	-0.931659000
H	-0.066242000	-6.514511000	-1.595216000
C	0.024272000	-5.701048000	0.389172000
H	0.687462000	-6.473183000	0.759325000
C	-0.402117000	-4.686239000	1.235362000
H	-0.055120000	-4.643592000	2.260311000
C	-1.258395000	-3.706316000	0.761300000
H	-1.578227000	-2.907668000	1.419323000
C	-2.919167000	-2.588511000	-2.903177000
C	-1.885474000	-2.076557000	-3.687603000
H	-1.064699000	-1.547629000	-3.219778000
C	-1.938610000	-2.176662000	-5.067607000
H	-1.126893000	-1.781176000	-5.665271000
C	-3.036930000	-2.763225000	-5.681936000
H	-3.085241000	-2.828445000	-6.761623000
C	-4.077687000	-3.256695000	-4.908679000
H	-4.944580000	-3.699643000	-5.382630000
C	-4.016842000	-3.177159000	-3.524964000
H	-4.834594000	-3.567550000	-2.934477000

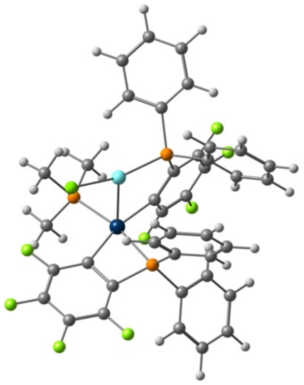

Figure S122. Optimized molecular structure of *trans*-**6PtAg**.

PBE0:

final single point energy: -28211.270811362836 a.u.

final Gibbs free energy: -28210.76654358 a.u.

Table S55. Atomic coordinates for optimized structure of *trans*-**6PtAg**.

Pt	0.023687000	-0.009314000	0.004212000
Ag	-0.112977000	-0.127798000	2.924842000
Cl	-2.302147000	0.347277000	3.663537000
P	-0.433050000	-2.273339000	-0.144475000
P	2.200631000	-0.520847000	2.744630000
P	0.051880000	2.280049000	-0.027218000
F	-3.398950000	-3.729705000	-0.703865000
F	-5.675357000	-2.247549000	-0.757314000
F	-5.548454000	0.413853000	-0.420984000
F	-3.249756000	1.672330000	-0.037190000
F	5.127587000	-0.487035000	1.977916000
F	6.221436000	-0.128916000	-0.431729000
F	4.634525000	0.249373000	-2.590767000
F	1.989438000	0.296137000	-2.347593000
C	-2.014148000	-0.356096000	-0.209632000
C	-2.124128000	-1.751848000	-0.398914000
C	-3.326984000	-2.404960000	-0.569508000
C	-4.493407000	-1.664415000	-0.585644000
C	-4.422002000	-0.289209000	-0.409967000
C	-3.202973000	0.343609000	-0.220264000
C	2.082334000	-0.064599000	-0.005577000
C	2.926260000	-0.268208000	1.101863000
C	4.309857000	-0.282622000	0.942482000
C	4.898602000	-0.107632000	-0.291940000
C	4.083757000	0.079935000	-1.394317000
C	2.712739000	0.094137000	-1.230216000
C	-0.777396000	3.089832000	1.363477000
H	-0.784229000	4.169758000	1.203968000
H	-0.233123000	2.864215000	2.280815000
H	-1.793584000	2.719285000	1.467790000
C	1.686858000	3.063039000	-0.015945000
H	2.236272000	2.787836000	-0.915277000
H	2.251923000	2.732679000	0.854878000
H	1.565013000	4.147081000	0.015535000

C	-0.707278000	2.973309000	-1.518350000
H	-1.745120000	2.656594000	-1.590692000
H	-0.162042000	2.608172000	-2.388940000
H	-0.655870000	4.063230000	-1.487025000
C	0.268604000	-3.189113000	-1.525078000
C	1.658852000	-3.239918000	-1.626927000
H	2.274923000	-2.740098000	-0.888258000
C	2.252386000	-3.922569000	-2.676000000
H	3.332364000	-3.960330000	-2.750657000
C	1.462621000	-4.544054000	-3.634143000
H	1.926683000	-5.070364000	-4.459501000
C	0.079094000	-4.486900000	-3.539248000
H	-0.537821000	-4.967974000	-4.288471000
C	-0.521194000	-3.813536000	-2.485816000
H	-1.600689000	-3.775131000	-2.417996000
C	-0.436365000	-3.391292000	1.278041000
C	-1.284719000	-3.093808000	2.345543000
H	-1.941819000	-2.232643000	2.300543000
C	-1.302688000	-3.909115000	3.464302000
H	-1.963375000	-3.669542000	4.288604000
C	-0.479928000	-5.026195000	3.524422000
H	-0.493135000	-5.662752000	4.400846000
C	0.359837000	-5.325225000	2.462888000
H	1.004575000	-6.193923000	2.507318000
C	0.386993000	-4.509761000	1.341194000
H	1.046183000	-4.751686000	0.517228000
C	2.700211000	-2.184957000	3.263625000
C	2.322337000	-2.603866000	4.539806000
H	1.742033000	-1.943884000	5.175902000
C	2.689981000	-3.856220000	5.000161000
H	2.397315000	-4.169722000	5.994881000
C	3.421753000	-4.710217000	4.185131000
H	3.706567000	-5.691558000	4.545020000
C	3.772009000	-4.310572000	2.905071000
H	4.327580000	-4.978896000	2.258286000
C	3.411405000	-3.053455000	2.442826000
H	3.685555000	-2.755629000	1.438795000
C	3.088231000	0.610876000	3.863120000
C	2.669778000	1.940328000	3.882893000
H	1.843017000	2.254280000	3.255419000
C	3.296204000	2.861418000	4.706583000
H	2.964005000	3.892517000	4.711555000
C	4.333265000	2.455774000	5.535699000
H	4.816592000	3.171333000	6.189953000
C	4.743915000	1.129973000	5.530830000
H	5.550863000	0.808687000	6.178454000
C	4.128704000	0.210117000	4.695082000
H	4.462838000	-0.819359000	4.689853000

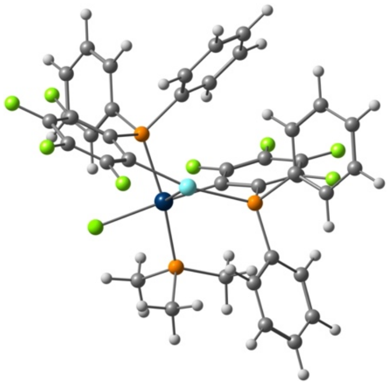

Figure S123. Optimized molecular structure of *cis-trans*-**7PtAg**.

PBE0:

final single point energy: -28211.262214243394 a.u.
final Gibbs free energy: -28210.75686993 a.u.

Table S56. Atomic coordinates for optimized structure of *cis-trans*-**7PtAg**.

Pt	3.769546000	6.653381000	4.806129000
Ag	1.243178000	5.468012000	3.861473000
Cl	4.159059000	4.914545000	6.388125000
P	4.222823000	5.117465000	3.092652000
P	0.718591000	7.738736000	3.517881000
C	2.223860000	8.679465000	3.115973000
F	0.169661000	2.342129000	4.378035000
C	-0.116950000	8.603837000	4.870326000
C	4.138075000	2.278631000	3.391545000
C	4.583959000	8.974941000	3.223688000
C	3.280593000	10.547389000	1.988026000
C	3.476474000	8.219564000	3.580382000
C	2.152872000	9.838540000	2.346924000
C	3.479244000	3.495141000	3.448963000
C	1.478774000	2.365738000	4.052657000
C	3.465240000	1.100909000	3.679492000
C	2.115468000	3.550717000	3.793592000
F	3.189877000	11.646361000	1.244883000
F	5.428226000	2.160688000	3.066662000
F	5.813182000	8.623366000	3.635176000
F	0.984557000	10.339298000	1.937717000
F	4.105150000	-0.065924000	3.636454000
F	5.606717000	10.778955000	2.103114000
F	1.487402000	0.006540000	4.290633000
C	4.511848000	10.106163000	2.433749000
C	3.646677000	5.497131000	1.406385000
C	6.019018000	4.951651000	2.868970000
P	3.637435000	8.052665000	6.616683000
C	-0.371783000	7.913181000	2.077397000
C	2.125384000	1.140528000	4.011733000
C	6.857766000	5.206700000	3.950666000
H	6.430384000	5.498367000	4.902153000
C	8.232077000	5.083355000	3.811236000
H	8.877349000	5.282259000	4.658386000

C	8.776943000	4.713397000	2.590446000
H	9.850999000	4.622191000	2.480350000
C	7.944130000	4.464155000	1.507076000
H	8.365524000	4.176156000	0.551442000
C	6.571111000	4.582450000	1.643924000
H	5.928774000	4.382846000	0.795512000
C	2.758412000	4.678158000	0.715176000
H	2.373663000	3.781088000	1.181079000
C	2.365055000	5.005050000	-0.575868000
H	1.674947000	4.357711000	-1.103247000
C	2.848832000	6.152537000	-1.184806000
H	2.536803000	6.408528000	-2.190170000
C	3.737060000	6.973290000	-0.500799000
H	4.125826000	7.868736000	-0.970464000
C	4.136915000	6.645318000	0.783511000
H	4.852290000	7.275611000	1.294383000
C	-0.868510000	7.832272000	5.754796000
H	-0.914406000	6.756381000	5.623849000
C	-1.544877000	8.431944000	6.806160000
H	-2.125450000	7.824154000	7.489209000
C	-1.461358000	9.804795000	6.989460000
H	-1.978492000	10.273402000	7.818044000
C	-0.711143000	10.578103000	6.113479000
C	-0.046060000	9.982825000	5.053130000
C	-1.670063000	8.400035000	2.169769000
H	-2.054564000	8.754178000	3.118369000
C	-2.474404000	8.440927000	1.038819000
H	-3.485426000	8.823033000	1.114612000
C	-1.986370000	8.002010000	-0.183188000
H	-2.615899000	8.039623000	-1.064039000
C	-0.689505000	7.510497000	-0.274438000
H	-0.302137000	7.161650000	-1.224343000
C	0.111753000	7.457035000	0.852463000
H	1.119072000	7.062847000	0.780514000
H	-0.643714000	11.649935000	6.255027000
H	0.529536000	10.597768000	4.373453000
C	3.418315000	9.822763000	6.315369000
H	3.368141000	10.345035000	7.272082000
H	4.261482000	10.202958000	5.739348000
H	2.500723000	9.997784000	5.757450000
C	2.313935000	7.624916000	7.770346000
H	2.412802000	6.574697000	8.042198000
H	2.380388000	8.250979000	8.661760000
H	1.350519000	7.780026000	7.285608000
C	5.158297000	7.987449000	7.594806000
H	5.065172000	8.637732000	8.466441000
H	5.335255000	6.960267000	7.908659000
H	5.992155000	8.320757000	6.976542000

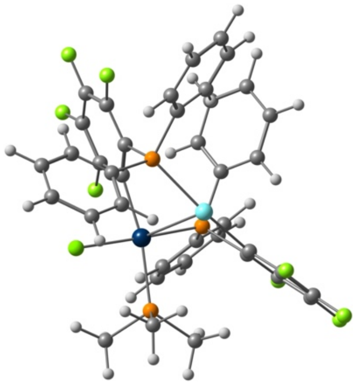

Figure S124. Optimized molecular structure of *cis-cis-***7PtAg**.

PBE0:

final single point energy: -28211.260139912327 a.u.
final Gibbs free energy: -28210.75393014 a.u.

Table S57. Atomic coordinates for optimized structure of *cis-cis-***7PtAg**.

Pt	5.389830000	12.953388000	8.074870000
Ag	2.857063000	13.209022000	6.681185000
Cl	6.050906000	15.137574000	8.697994000
P	6.741730000	13.291934000	6.211527000
P	2.232595000	14.349768000	8.650661000
P	4.772717000	10.854295000	7.563558000
F	1.422002000	14.307358000	11.607814000
F	2.683341000	13.173824000	13.652647000
F	5.072088000	11.956160000	13.271810000
F	6.171444000	11.848618000	10.857702000
F	5.443568000	8.628029000	5.371882000
F	4.704346000	8.657702000	2.833788000
F	3.201173000	10.670848000	1.848824000
F	2.394784000	12.671305000	3.446013000
C	4.343450000	12.973683000	9.853916000
C	3.101977000	13.606324000	10.066516000
C	2.569573000	13.672688000	11.350754000
C	3.214276000	13.110425000	12.434614000
C	4.437050000	12.498672000	12.239342000
C	4.978036000	12.458924000	10.968093000
C	3.522540000	11.748288000	5.305536000
C	4.318119000	10.696640000	5.801617000
C	4.691351000	9.654321000	4.967812000
C	4.316588000	9.647096000	3.633864000
C	3.548098000	10.679652000	3.131698000
C	3.159640000	11.700447000	3.984119000
C	2.558112000	16.128325000	8.693405000
C	2.640247000	16.796494000	7.474429000
H	2.526783000	16.241904000	6.548959000
C	2.879614000	18.161846000	7.440281000
H	2.944153000	18.674063000	6.488019000
C	3.050159000	18.863516000	8.624789000
H	3.248067000	19.928416000	8.600278000
C	2.975847000	18.199699000	9.842556000

H	3.115250000	18.744846000	10.768221000
C	2.728101000	16.837097000	9.879615000
H	2.678183000	16.330646000	10.834495000
C	0.458366000	14.163371000	8.985727000
C	-0.423590000	15.236923000	8.947702000
H	-0.053202000	16.241136000	8.782340000
C	-1.783295000	15.022691000	9.130548000
H	-2.466020000	15.863539000	9.101822000
C	-2.265978000	13.742037000	9.355292000
H	-3.327030000	13.579479000	9.501578000
C	-1.384836000	12.667552000	9.390632000
H	-1.754309000	11.663724000	9.564464000
C	-0.030281000	12.875440000	9.198525000
H	0.652072000	12.033565000	9.218741000
C	6.053908000	9.649811000	8.020599000
C	7.350658000	10.096241000	8.251553000
H	7.572295000	11.153156000	8.167189000
C	8.345469000	9.203059000	8.621448000
H	9.351121000	9.562807000	8.801763000
C	8.045079000	7.858447000	8.774617000
H	8.818516000	7.159636000	9.070148000
C	6.747291000	7.408469000	8.561603000
H	6.507426000	6.359893000	8.689676000
C	5.755335000	8.298226000	8.189105000
H	4.746872000	7.939478000	8.026037000
C	3.303438000	10.195917000	8.419983000
C	3.399538000	9.905048000	9.780939000
H	4.341920000	10.028921000	10.297281000
C	2.300934000	9.431408000	10.476466000
H	2.389501000	9.215877000	11.534364000
C	1.094927000	9.224266000	9.817788000
H	0.235649000	8.851776000	10.362350000
C	0.998790000	9.488329000	8.461027000
H	0.065598000	9.320814000	7.937045000
C	2.096193000	9.976876000	7.763646000
H	2.006276000	10.181597000	6.705672000
C	7.071867000	12.022404000	4.957704000
H	7.379271000	11.086593000	5.424197000
H	6.184307000	11.849252000	4.353755000
H	7.876696000	12.382487000	4.314651000
C	6.136092000	14.678888000	5.217082000
H	6.840827000	14.903213000	4.414642000
H	5.170077000	14.405254000	4.790847000
H	6.009704000	15.549033000	5.859591000
C	8.423396000	13.765193000	6.699699000
H	8.992643000	14.058826000	5.816261000
H	8.376117000	14.588946000	7.408747000
H	8.912335000	12.913624000	7.174541000

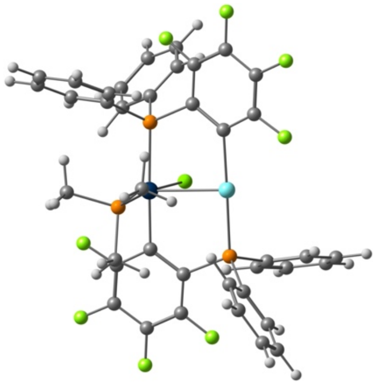

Figure S125. Optimized molecular structure of *trans*-**7PtAg**.

PBE0:

final single point energy: -28211.255003679002 a.u.

final Gibbs free energy: -28210.74865514 a.u.

Table S58. Atomic coordinates for optimized structure of *trans*-**7PtAg**.

Pt	22.229347000	4.145352000	6.004076000
Ag	24.520801000	5.730182000	6.482897000
Cl	21.468151000	6.171212000	5.020023000
P	25.031299000	5.287189000	4.208921000
P	21.491445000	4.904970000	8.093262000
P	23.107375000	2.207759000	6.710007000
F	25.102081000	4.267674000	1.366695000
F	23.428808000	2.603002000	0.128747000
F	21.281557000	1.611223000	1.449349000
F	20.829355000	2.260267000	3.988160000
F	21.258186000	5.974702000	10.932496000
F	23.076949000	7.267687000	12.374165000
F	25.493738000	7.860254000	11.321743000
F	26.088394000	7.164172000	8.809576000
C	22.749062000	3.644786000	4.085327000
C	23.862426000	4.152567000	3.390851000
C	24.069499000	3.789397000	2.063579000
C	23.213244000	2.935297000	1.398111000
C	22.115163000	2.435019000	2.072332000
C	21.906471000	2.798662000	3.389463000
C	23.975002000	6.153648000	8.482998000
C	22.725314000	5.840391000	9.051362000
C	22.438682000	6.229951000	10.358022000
C	23.364687000	6.907346000	11.126776000
C	24.599730000	7.210974000	10.583432000
C	24.868105000	6.831523000	9.281106000
C	24.040004000	1.269963000	5.470930000
H	24.495486000	0.409944000	5.964764000
H	24.816867000	1.891099000	5.028919000
H	23.375638000	0.922147000	4.681730000
C	21.929683000	0.968469000	7.300698000
H	22.472069000	0.055399000	7.552340000
H	21.215393000	0.760585000	6.503959000
H	21.392013000	1.326532000	8.174640000

C	24.333121000	2.384038000	8.029322000
H	23.909138000	2.895056000	8.888366000
H	25.167371000	2.977923000	7.656539000
H	24.688509000	1.396747000	8.329031000
C	25.001778000	6.820199000	3.242687000
C	25.982028000	7.777756000	3.507030000
H	26.768684000	7.566427000	4.223112000
C	25.954549000	9.000598000	2.858779000
H	26.723174000	9.735462000	3.065721000
C	24.940080000	9.285629000	1.953023000
H	24.916449000	10.244416000	1.449237000
C	23.954561000	8.343198000	1.701620000
H	23.156375000	8.563197000	1.003015000
C	23.982017000	7.113546000	2.344423000
H	23.199600000	6.390133000	2.153562000
C	26.691725000	4.583306000	3.958695000
C	27.511204000	4.901318000	2.878620000
H	27.169798000	5.597871000	2.124448000
C	28.768954000	4.329511000	2.767633000
H	29.401347000	4.586357000	1.926270000
C	29.216836000	3.430281000	3.726151000
H	30.200068000	2.984644000	3.634335000
C	28.407796000	3.112998000	4.807978000
H	28.755890000	2.422891000	5.566974000
C	27.156678000	3.697084000	4.928754000
H	26.538317000	3.473175000	5.790293000
C	20.045062000	5.994767000	7.904872000
C	18.994931000	5.560268000	7.095513000
H	19.072681000	4.618980000	6.564708000
C	17.868849000	6.346095000	6.923758000
H	17.060081000	5.993822000	6.295050000
C	17.791145000	7.592377000	7.531394000
H	16.915167000	8.213589000	7.388172000
C	18.848642000	8.047480000	8.303207000
H	18.807043000	9.028203000	8.761507000
C	19.970916000	7.252528000	8.491047000
H	20.792333000	7.634242000	9.081744000
C	20.910801000	3.612662000	9.251779000
C	19.660714000	3.022837000	9.073410000
H	18.990630000	3.378481000	8.303393000
C	19.256814000	1.968686000	9.877476000
H	18.280946000	1.524343000	9.723342000
C	20.097208000	1.482364000	10.868859000
H	19.782844000	0.654983000	11.493256000
C	21.338423000	2.070270000	11.060805000
H	21.998559000	1.710419000	11.840689000
C	21.740092000	3.129250000	10.261080000
H	22.703605000	3.587699000	10.443793000

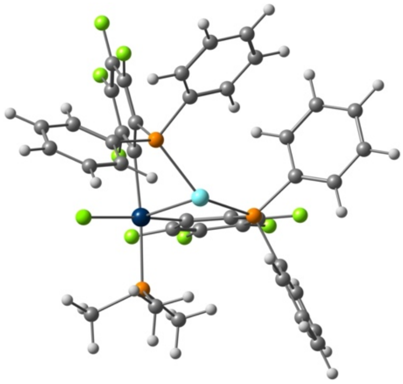

Figure S126. Optimized molecular structure of *cis*-**8PtAg**.

PBE0:

final single point energy: -28211.247378312386 a.u.
final Gibbs free energy: -28210.74281863 a.u.

Table S59. Atomic coordinates for optimized structure of *cis*-**8PtAg**.

Pt	3.850672000	6.340030000	4.703793000
Ag	1.396450000	4.996179000	4.610203000
Cl	4.732329000	4.801876000	6.288567000
P	0.540172000	6.902708000	3.478966000
C	1.916244000	7.919568000	2.883029000
F	5.824637000	6.543740000	2.247360000
C	-0.545341000	7.957748000	4.480785000
C	4.528977000	2.701021000	2.255120000
C	4.142250000	8.725621000	3.064347000
C	2.634346000	9.829940000	1.574003000
C	3.212601000	7.753943000	3.424406000
C	1.663500000	8.933060000	1.958985000
C	3.956032000	3.581643000	3.166174000
C	5.331081000	5.295573000	2.291892000
C	5.481526000	3.107927000	1.343692000
C	4.366679000	4.939834000	3.219720000
F	2.373459000	10.792279000	0.693060000
F	4.188641000	1.407953000	2.223904000
F	5.361475000	8.749301000	3.622544000
F	0.449961000	9.094807000	1.419001000
F	6.011565000	2.252000000	0.473588000
F	4.825342000	10.613221000	1.833508000
F	6.797206000	4.846325000	0.496343000
C	3.886078000	9.731650000	2.154672000
P	2.583938000	2.978854000	4.202604000
C	3.216129000	1.850342000	5.463607000
C	1.570401000	1.929665000	3.108609000
P	3.395279000	7.725391000	6.465395000
C	-0.412650000	6.426755000	2.010086000
C	5.879424000	4.427705000	1.361728000
C	0.908943000	2.556276000	2.053501000
H	1.035146000	3.621126000	1.891415000
C	0.085255000	1.830397000	1.210457000
H	-0.419442000	2.331095000	0.392636000

C	-0.101614000	0.470273000	1.424890000
H	-0.752912000	-0.098571000	0.772254000
C	0.545321000	-0.155721000	2.480348000
H	0.404053000	-1.216206000	2.652133000
C	1.382580000	0.568126000	3.318308000
H	1.893801000	0.067379000	4.130848000
C	4.498770000	1.315559000	5.432865000
H	5.182851000	1.582067000	4.637815000
C	4.914596000	0.456048000	6.438155000
H	5.917790000	0.047932000	6.412983000
C	4.053567000	0.124395000	7.475125000
H	4.383283000	-0.545175000	8.260444000
C	2.773042000	0.659756000	7.510823000
H	2.100400000	0.411500000	8.322931000
C	2.357561000	1.525543000	6.511748000
H	1.362827000	1.957271000	6.548177000
C	-1.285228000	7.315891000	5.474162000
H	-1.187872000	6.243274000	5.607564000
C	-2.136571000	8.037148000	6.296568000
H	-2.707803000	7.525213000	7.061339000
C	-2.239804000	9.412630000	6.147978000
H	-2.894398000	9.981334000	6.797286000
C	-1.495369000	10.060471000	5.171738000
C	-0.655372000	9.339064000	4.337489000
C	-1.803082000	6.402167000	2.017273000
H	-2.350607000	6.765055000	2.878787000
C	-2.493722000	5.917708000	0.915324000
H	-3.577062000	5.905371000	0.926064000
C	-1.803262000	5.456331000	-0.195955000
H	-2.345245000	5.079929000	-1.055012000
C	-0.414325000	5.480397000	-0.204807000
H	0.131501000	5.123388000	-1.069878000
C	0.279863000	5.958581000	0.894039000
H	1.364579000	5.971945000	0.883378000
H	-1.566961000	11.135290000	5.057442000
H	-0.083650000	9.864411000	3.585621000
C	2.721711000	9.373720000	6.128979000
H	2.548246000	9.896190000	7.071126000
H	3.426221000	9.944010000	5.523986000
H	1.782169000	9.287838000	5.585149000
C	2.187113000	7.010375000	7.612385000
H	2.530311000	6.021516000	7.915075000
H	2.067978000	7.648751000	8.489604000
H	1.226666000	6.915679000	7.103140000
C	4.843143000	8.081703000	7.493636000
H	4.554980000	8.688738000	8.353633000
H	5.280886000	7.141822000	7.826471000
H	5.577684000	8.621409000	6.895080000

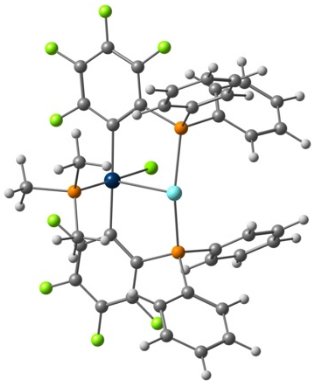

Figure S127. Optimized molecular structure of *trans*-**8PtAg**.

PBE0:

final single point energy: -28211.257161613808 a.u.

final Gibbs free energy: -28210.75259721 a.u.

Table S60. Atomic coordinates for optimized structure of *trans*-**8PtAg**.

Pt	3.874163000	6.056940000	6.770018000
Ag	5.873085000	6.054372000	8.710503000
Cl	5.551888000	6.055815000	5.081763000
F	5.538023000	0.854819000	7.599331000
F	3.562801000	-0.132482000	6.104896000
F	1.798103000	1.545351000	4.921484000
F	1.986814000	4.167472000	5.240422000
P	6.126897000	3.717800000	8.311503000
P	2.262621000	6.057563000	8.292345000
C	3.862338000	3.985584000	6.681773000
C	4.768834000	3.088856000	7.289840000
C	4.659201000	1.716365000	7.077387000
C	3.657767000	1.180979000	6.294964000
C	2.762320000	2.041499000	5.688280000
C	2.890543000	3.404097000	5.883274000
C	6.299182000	2.568913000	9.710500000
C	7.525850000	2.024273000	10.075037000
H	8.409860000	2.224685000	9.483358000
C	7.620055000	1.206505000	11.192383000
H	8.579641000	0.783612000	11.464908000
C	6.493788000	0.923320000	11.950356000
H	6.569653000	0.281132000	12.819441000
C	5.266461000	1.462665000	11.586895000
H	4.379847000	1.241068000	12.168872000
C	5.170087000	2.285649000	10.477415000
H	4.207334000	2.695304000	10.197541000
C	7.640079000	3.538547000	7.325862000
C	7.681007000	2.841281000	6.123126000
H	6.791842000	2.359676000	5.737993000
C	8.859127000	2.782701000	5.393601000
H	8.878997000	2.245514000	4.453169000
C	10.002926000	3.414378000	5.859710000
H	10.920057000	3.369448000	5.284898000
C	9.965970000	4.117549000	7.056168000

H	10.851199000	4.625756000	7.418482000
C	8.787566000	4.188727000	7.780018000
H	8.754414000	4.761733000	8.700565000
C	0.596975000	6.059846000	7.582226000
H	-0.142647000	6.060089000	8.384959000
H	0.468927000	6.945473000	6.961621000
C	2.248635000	7.464152000	9.430226000
H	1.395824000	7.382606000	10.105983000
H	2.191239000	8.397458000	8.872156000
H	3.171776000	7.452966000	10.010393000
H	0.466909000	5.175427000	6.960325000
C	2.245790000	4.649367000	9.428263000
H	1.393653000	4.732203000	10.104714000
H	2.185610000	3.716946000	8.869021000
H	3.169369000	4.657341000	10.007794000
F	5.554616000	11.254049000	7.597362000
F	3.583278000	12.247004000	6.101337000
F	1.812275000	10.574359000	4.920158000
F	1.991580000	7.951941000	5.241615000
P	6.131597000	8.390992000	8.315213000
C	3.867656000	8.128448000	6.683008000
C	4.776993000	9.022612000	7.290613000
C	4.672594000	10.395266000	7.076339000
C	3.673330000	10.933439000	6.293104000
C	2.774643000	10.075505000	5.687529000
C	2.897905000	8.712668000	5.883969000
C	6.302764000	9.540419000	9.713992000
C	7.528754000	10.085668000	10.079858000
H	8.413683000	9.885122000	9.489632000
C	7.621169000	10.904412000	11.196624000
H	8.580290000	11.327722000	11.470137000
C	6.493760000	11.188063000	11.952728000
H	6.568252000	11.830997000	12.821377000
C	5.267074000	10.648229000	11.587888000
H	4.379530000	10.870166000	12.168314000
C	5.172513000	9.824246000	10.478986000
H	4.210234000	9.414270000	10.198046000
C	7.647391000	8.566122000	7.332935000
C	7.689893000	9.253482000	6.124607000
H	6.800744000	9.730566000	5.733775000
C	8.869504000	9.307642000	5.397075000
H	8.890588000	9.837008000	4.452246000
C	10.013170000	8.681503000	5.870800000
H	10.931468000	8.722978000	5.297599000
C	9.974677000	7.988287000	7.073067000
H	10.859940000	7.484577000	7.441550000
C	8.794850000	7.921288000	7.794923000
H	8.760620000	7.355750000	8.720062000

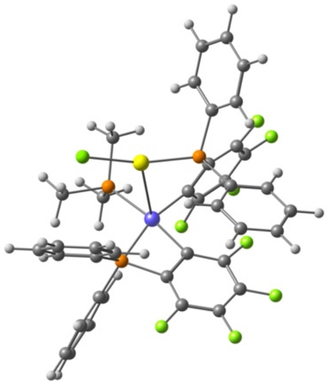

Figure S128. Optimized molecular structure of *cis*-**6NiAu**.

PBE0:

final single point energy: -24996.284554649272 a.u.

final Gibbs free energy: -24995.77822161 a.u.

Table S61. Atomic coordinates for optimized structure of *cis*-**6NiAu**.

Ni	-0.601439000	0.152033000	1.039661000
Au	0.860745000	-2.093514000	-0.345800000
Cl	-0.160192000	-4.157922000	-0.341707000
P	-2.515471000	-0.364998000	0.018717000
P	2.243720000	-0.334015000	-0.438422000
P	-0.511503000	-1.281143000	2.716736000
F	-3.700665000	1.226890000	-2.705388000
F	-2.512052000	3.519642000	-3.563959000
F	-0.411132000	4.519165000	-2.240308000
F	0.625709000	3.304556000	-0.124084000
F	4.539983000	1.354522000	0.790058000
F	4.378018000	3.061665000	2.824109000
F	2.003716000	3.518383000	4.033591000
F	-0.207176000	2.265320000	3.215103000
C	-0.933884000	1.502865000	-0.298873000
C	-2.062982000	1.035618000	-0.993602000
C	-2.618251000	1.688098000	-2.076113000
C	-2.034079000	2.861210000	-2.511455000
C	-0.939312000	3.371308000	-1.824836000
C	-0.410246000	2.707663000	-0.729106000
C	0.992735000	0.987914000	1.624624000
C	2.232124000	0.771064000	0.996605000
C	3.356869000	1.485661000	1.397581000
C	3.293469000	2.395163000	2.434660000
C	2.077224000	2.626471000	3.051967000
C	0.958291000	1.937543000	2.624484000
C	1.080387000	-2.073802000	3.092067000
H	1.004109000	-2.623057000	4.032092000
H	1.852285000	-1.308593000	3.175977000
H	1.354063000	-2.762617000	2.292881000
C	-1.679342000	-2.668173000	2.664624000
H	-1.553544000	-3.303132000	3.543356000
H	-1.510070000	-3.253425000	1.760783000
H	-2.695605000	-2.271221000	2.650678000

C	-0.911803000	-0.514198000	4.318663000
H	-1.883543000	-0.024532000	4.272553000
H	-0.158040000	0.226913000	4.576970000
H	-0.934475000	-1.289574000	5.086712000
C	-4.066789000	0.070404000	0.847189000
C	-3.983427000	0.729242000	2.071047000
H	-3.009461000	0.923776000	2.505875000
C	-5.135038000	1.147592000	2.719889000
H	-5.062092000	1.659656000	3.671709000
C	-6.376514000	0.906051000	2.148911000
H	-7.278045000	1.228169000	2.656155000
C	-6.464548000	0.252622000	0.926131000
H	-7.433165000	0.068401000	0.477204000
C	-5.314963000	-0.163842000	0.273786000
H	-5.388617000	-0.669445000	-0.681049000
C	-2.897669000	-1.782089000	-1.031595000
C	-3.623056000	-2.860947000	-0.531332000
H	-3.996889000	-2.842407000	0.485332000
C	-3.878266000	-3.958784000	-1.336076000
H	-4.446286000	-4.792654000	-0.941738000
C	-3.405225000	-3.992160000	-2.640840000
H	-3.605076000	-4.852458000	-3.268148000
C	-2.674083000	-2.923718000	-3.139435000
H	-2.301231000	-2.945584000	-4.156304000
C	-2.419086000	-1.822172000	-2.338184000
H	-1.850122000	-0.988936000	-2.732502000
C	1.997768000	0.705506000	-1.895272000
C	2.672711000	1.914622000	-2.046931000
H	3.363967000	2.257783000	-1.287730000
C	2.450646000	2.692483000	-3.171050000
H	2.970364000	3.636222000	-3.281769000
C	1.556824000	2.270075000	-4.146913000
H	1.377710000	2.887681000	-5.018791000
C	0.887753000	1.063623000	-4.001974000
H	0.186021000	0.733314000	-4.758101000
C	1.107477000	0.282866000	-2.878094000
H	0.577855000	-0.654160000	-2.750736000
C	3.913904000	-1.026753000	-0.580886000
C	4.418900000	-1.749550000	0.499529000
H	3.836431000	-1.844367000	1.409421000
C	5.665685000	-2.343377000	0.414488000
H	6.056686000	-2.896652000	1.259771000
C	6.410453000	-2.234454000	-0.754243000
H	7.385031000	-2.702746000	-0.821122000
C	5.902907000	-1.529437000	-1.834642000
H	6.478638000	-1.445114000	-2.748443000
C	4.656042000	-0.924289000	-1.751091000
H	4.267311000	-0.371368000	-2.596915000

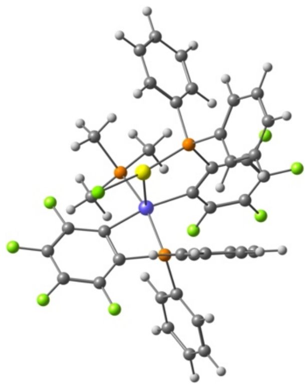

Figure S129. Optimized molecular structure of *trans*-**6NiAu**.

PBE0:

final single point energy: -24996.283190876231 a.u.

final Gibbs free energy: -24995.77749839 a.u.

Table S62. Atomic coordinates for optimized structure of *trans*-**6NiAu**.

Ni	0.602317000	-0.356258000	-1.059644000
Au	-0.371350000	-1.361545000	1.494549000
Cl	1.185951000	-2.592666000	2.676592000
P	1.881123000	1.018582000	0.072708000
P	-2.177930000	-0.276973000	0.738015000
P	-0.176392000	-1.706725000	-2.583389000
F	4.690084000	0.198204000	1.709549000
F	5.691390000	-2.327336000	1.526705000
F	4.425796000	-4.163383000	0.037939000
F	2.193183000	-3.618654000	-1.269167000
F	-3.934746000	2.159057000	0.095106000
F	-3.542853000	4.006008000	-1.785995000
F	-1.393694000	3.840725000	-3.422313000
F	0.353925000	1.843069000	-3.180219000
C	2.183972000	-1.361054000	-0.494329000
C	2.882484000	-0.433396000	0.308097000
C	4.053339000	-0.717271000	0.977526000
C	4.575472000	-1.992236000	0.886887000
C	3.917571000	-2.936849000	0.111081000
C	2.752223000	-2.616547000	-0.566143000
C	-0.785220000	0.930301000	-1.318415000
C	-1.913674000	1.031795000	-0.483146000
C	-2.832827000	2.063644000	-0.653550000
C	-2.659802000	3.022544000	-1.630347000
C	-1.563518000	2.930311000	-2.470243000
C	-0.669870000	1.889390000	-2.306624000
C	-1.516502000	-1.081530000	-3.638213000
H	-1.187010000	-0.180665000	-4.155129000
H	-2.386565000	-0.840305000	-3.030628000
H	-1.787297000	-1.841142000	-4.373909000
C	-0.845795000	-3.300197000	-2.030959000
H	-1.071126000	-3.928216000	-2.895053000
H	-1.763285000	-3.115057000	-1.472824000
H	-0.135213000	-3.804846000	-1.381360000

C	1.086898000	-2.110547000	-3.825274000
H	0.661202000	-2.758882000	-4.593806000
H	1.935990000	-2.603013000	-3.358665000
H	1.425634000	-1.181649000	-4.286084000
C	1.398296000	1.829361000	1.605524000
C	0.655065000	3.008021000	1.523307000
H	0.393323000	3.419689000	0.554790000
C	0.260908000	3.660838000	2.679525000
H	-0.309808000	4.578803000	2.610521000
C	0.589535000	3.133769000	3.922180000
H	0.280310000	3.645145000	4.825727000
C	1.298511000	1.943997000	4.006144000
H	1.540565000	1.520869000	4.973347000
C	1.704300000	1.289909000	2.851557000
H	2.251680000	0.358602000	2.922772000
C	2.902431000	2.217664000	-0.824531000
C	3.228859000	1.919396000	-2.146695000
H	2.821651000	1.028648000	-2.610712000
C	4.065793000	2.757552000	-2.863529000
H	4.314913000	2.519345000	-3.890540000
C	4.576609000	3.904236000	-2.268686000
H	5.226487000	4.563383000	-2.831658000
C	4.254117000	4.204622000	-0.953266000
H	4.654545000	5.095376000	-0.484344000
C	3.422355000	3.363131000	-0.228678000
H	3.184405000	3.599271000	0.800593000
C	-3.025733000	0.436123000	2.175020000
C	-4.376587000	0.223507000	2.428398000
H	-4.973786000	-0.368705000	1.746622000
C	-4.963240000	0.778971000	3.555515000
H	-6.015402000	0.609884000	3.750397000
C	-4.208003000	1.550246000	4.427926000
H	-4.670228000	1.983551000	5.306768000
C	-2.858030000	1.759055000	4.178036000
H	-2.261033000	2.353276000	4.859079000
C	-2.265534000	1.195094000	3.060762000
H	-1.207733000	1.339098000	2.877809000
C	-3.386403000	-1.408238000	0.001404000
C	-3.511767000	-2.683362000	0.552406000
H	-2.876359000	-2.976361000	1.380924000
C	-4.433210000	-3.578025000	0.034733000
H	-4.522122000	-4.567583000	0.465953000
C	-5.229258000	-3.210650000	-1.042752000
H	-5.942046000	-3.914783000	-1.454677000
C	-5.107882000	-1.943711000	-1.593690000
H	-5.725603000	-1.653361000	-2.434766000
C	-4.192285000	-1.041628000	-1.071187000
H	-4.105363000	-0.056077000	-1.510675000

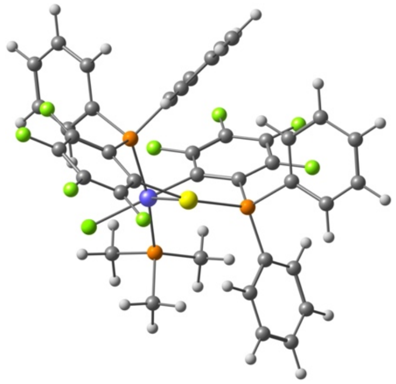

Figure S130. Optimized molecular structure of *cis-trans*-**7NiAu**.

PBE0:

final single point energy: -24996.298121780965 a.u.

final Gibbs free energy: -24995.79105683 a.u.

Table S63. Atomic coordinates for optimized structure of *cis-trans*-**7NiAu**.

Ni	-0.386041000	0.900810000	-1.291961000
Au	0.149071000	-1.705967000	-0.289245000
P	2.196557000	-0.801995000	0.136484000
Cl	-1.824139000	0.213432000	-2.827769000
C	1.967379000	0.967641000	0.484383000
F	-1.552600000	-4.482285000	-0.920587000
C	3.472168000	-0.967968000	-1.132460000
C	-4.098670000	-1.393147000	-0.087660000
C	0.734052000	2.980898000	0.221022000
C	2.678934000	2.994845000	1.601995000
C	0.862229000	1.637039000	-0.080994000
C	2.857097000	1.659694000	1.301389000
C	-2.736954000	-1.155276000	-0.079328000
C	-2.356679000	-3.440744000	-0.647435000
C	-4.594999000	-2.652995000	-0.390119000
C	-1.836929000	-2.199767000	-0.371973000
F	3.533479000	3.635681000	2.394125000
F	-5.000828000	-0.448549000	0.185576000
F	-0.278584000	3.698158000	-0.299659000
F	3.938216000	1.071926000	1.821557000
F	-5.907434000	-2.870844000	-0.408280000
F	1.419875000	4.950235000	1.325307000
F	-4.201091000	-4.890259000	-0.960352000
C	1.601515000	3.664280000	1.051457000
P	-1.923694000	0.437081000	0.289656000
C	-1.432400000	0.248160000	2.037662000
C	-3.160253000	1.771104000	0.384923000
P	0.837990000	1.646041000	-2.958888000
C	2.886037000	-1.537698000	1.641463000
C	-3.721471000	-3.684609000	-0.670397000
C	-3.330266000	2.608338000	-0.714179000
H	-2.747646000	2.438306000	-1.610668000
C	-4.242769000	3.651810000	-0.663617000
H	-4.367485000	4.297868000	-1.524309000

C	-4.985204000	3.871008000	0.487063000
H	-5.694001000	4.689542000	0.528752000
C	-4.814813000	3.042572000	1.589231000
H	-5.391444000	3.210799000	2.490916000
C	-3.906387000	1.998692000	1.540295000
H	-3.780522000	1.357478000	2.403576000
C	-1.583200000	-0.948461000	2.733602000
H	-2.011599000	-1.811485000	2.242458000
C	-1.189762000	-1.043325000	4.061814000
H	-1.315897000	-1.980925000	4.589543000
C	-0.635500000	0.051164000	4.706799000
H	-0.323059000	-0.026127000	5.741248000
C	-0.484443000	1.249460000	4.020218000
H	-0.058890000	2.113309000	4.516537000
C	-0.884988000	1.348423000	2.698410000
H	-0.788900000	2.295954000	2.186009000
C	3.287367000	-1.935572000	-2.116959000
H	2.385824000	-2.538226000	-2.112420000
C	4.245609000	-2.118984000	-3.103083000
H	4.094296000	-2.871743000	-3.866978000
C	5.386763000	-1.330898000	-3.114245000
H	6.131697000	-1.467390000	-3.888899000
C	5.575175000	-0.364336000	-2.134180000
C	4.624856000	-0.184024000	-1.142733000
C	4.025566000	-2.332697000	1.623852000
H	4.573821000	-2.482603000	0.701990000
C	4.465594000	-2.933427000	2.795604000
H	5.355234000	-3.551440000	2.778849000
C	3.773072000	-2.741559000	3.981920000
H	4.121206000	-3.209678000	4.894773000
C	2.630442000	-1.950437000	3.997577000
H	2.082583000	-1.798927000	4.919938000
C	2.183062000	-1.356136000	2.830979000
H	1.287021000	-0.746495000	2.844485000
H	6.465938000	0.251883000	-2.142263000
H	4.784500000	0.568294000	-0.381180000
C	2.444405000	2.427954000	-2.641258000
H	2.880130000	2.744676000	-3.590389000
H	2.318722000	3.295781000	-1.995042000
H	3.115586000	1.721781000	-2.155750000
C	1.243985000	0.418591000	-4.227503000
H	0.330919000	-0.067942000	-4.563784000
H	1.741493000	0.909492000	-5.065854000
H	1.911840000	-0.327927000	-3.797987000
C	-0.059116000	2.955759000	-3.837309000
H	0.538393000	3.315460000	-4.677023000
H	-1.009333000	2.563054000	-4.193947000
H	-0.248756000	3.778707000	-3.147625000

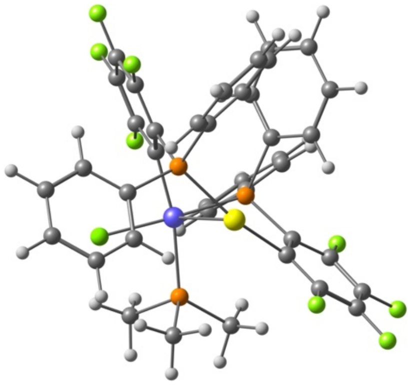

Figure S131. Optimized molecular structure of *cis*-*cis*-**7NiAu**.

PBE0:

final single point energy: -24996.291619039093 a.u.
final Gibbs free energy: -24995.78372146 a.u.

Table S64. Atomic coordinates for optimized structure of *cis*-*cis*-**7NiAu**.

Ni	0.399963000	0.370818000	-1.505648000
Au	-0.356328000	-1.587171000	0.343323000
Cl	-0.982849000	0.199980000	-3.217111000
P	1.771111000	-0.917498000	-2.744144000
P	-2.400652000	-0.586170000	0.181332000
P	1.760033000	0.635006000	0.186435000
F	-4.230222000	1.800670000	0.761537000
F	-3.774642000	4.381333000	0.286294000
F	-1.478701000	5.155658000	-0.920929000
F	0.357770000	3.361412000	-1.632155000
F	4.741008000	-0.190273000	1.036180000
F	5.587208000	-2.635740000	1.592734000
F	3.889086000	-4.734433000	1.543104000
F	1.296657000	-4.369836000	0.970368000
C	-0.909424000	1.564873000	-0.758961000
C	-2.109778000	1.177139000	-0.134142000
C	-3.061886000	2.134282000	0.203123000
C	-2.857243000	3.476522000	-0.046435000
C	-1.687435000	3.867924000	-0.670685000
C	-0.754296000	2.910415000	-1.020663000
C	1.614719000	-2.050084000	0.649787000
C	2.514837000	-0.964571000	0.657472000
C	3.840439000	-1.172509000	0.998271000
C	4.306403000	-2.443845000	1.294628000
C	3.436511000	-3.517092000	1.268206000
C	2.103837000	-3.297699000	0.958119000
C	-3.528339000	-1.223867000	-1.075928000
C	-3.338611000	-2.533650000	-1.508831000
H	-2.518671000	-3.118090000	-1.106019000
C	-4.185030000	-3.082173000	-2.460133000
H	-4.031360000	-4.100980000	-2.794367000
C	-5.216690000	-2.320673000	-2.989566000
H	-5.873219000	-2.744808000	-3.739794000

C	-5.404166000	-1.011732000	-2.564441000
H	-6.205464000	-0.414068000	-2.981635000
C	-4.565432000	-0.462775000	-1.608213000
H	-4.719443000	0.558864000	-1.287352000
C	-3.286345000	-0.686389000	1.759259000
C	-4.440193000	-1.445307000	1.913610000
H	-4.877668000	-1.952471000	1.062480000
C	-5.037475000	-1.547097000	3.162923000
H	-5.938322000	-2.137636000	3.279077000
C	-4.488031000	-0.893401000	4.255889000
H	-4.958837000	-0.972756000	5.228377000
C	-3.332432000	-0.136653000	4.101664000
H	-2.897413000	0.376416000	4.951192000
C	-2.730025000	-0.037548000	2.859948000
H	-1.826573000	0.549361000	2.743816000
C	3.052121000	1.863290000	-0.178026000
C	3.276394000	2.219735000	-1.503281000
H	2.674771000	1.774678000	-2.285702000
C	4.236886000	3.166742000	-1.826202000
H	4.399474000	3.436511000	-2.862548000
C	4.970058000	3.776159000	-0.819976000
H	5.716266000	4.521723000	-1.067323000
C	4.737599000	3.440283000	0.508681000
H	5.302168000	3.921981000	1.297826000
C	3.782713000	2.491700000	0.829853000
H	3.608037000	2.236617000	1.867300000
C	1.102782000	1.237370000	1.778906000
C	0.683654000	2.564410000	1.875205000
H	0.784081000	3.229437000	1.028227000
C	0.160411000	3.051462000	3.060479000
H	-0.165082000	4.083118000	3.117203000
C	0.063876000	2.224314000	4.172856000
H	-0.343870000	2.606748000	5.100808000
C	0.497889000	0.911017000	4.091117000
H	0.433719000	0.259877000	4.954422000
C	1.010609000	0.416480000	2.899057000
H	1.340644000	-0.612093000	2.850372000
C	3.466507000	-1.409238000	-2.292049000
H	4.052060000	-0.550186000	-1.965040000
H	3.446454000	-2.153026000	-1.498993000
H	3.939071000	-1.846096000	-3.173494000
C	1.016450000	-2.530440000	-3.093272000
H	1.637810000	-3.085793000	-3.798000000
H	0.937909000	-3.086935000	-2.158675000
H	0.020578000	-2.381625000	-3.505504000
C	2.073882000	-0.186118000	-4.382235000
H	2.600413000	-0.909284000	-5.007325000
H	1.128747000	0.091621000	-4.841570000
H	2.694756000	0.704220000	-4.274524000

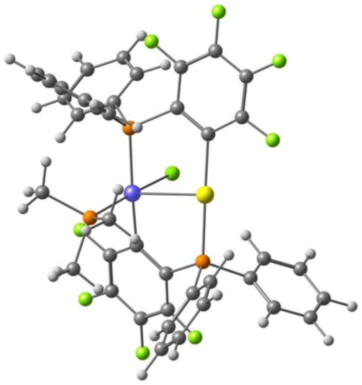

Figure S132. Optimized molecular structure of *trans*-**7NiAu**.

PBE0:
final single point energy: -24996.288271718389 a.u.
final Gibbs free energy: -24995.78058644 a.u.

Table S65. Atomic coordinates for optimized structure of *trans*-**7NiAu**.

Ni	-0.220763000	-1.175103000	-0.121834000
Au	0.318176000	1.524602000	-0.240201000
Cl	-0.249234000	-0.864694000	-2.332012000
P	2.488689000	0.861394000	0.020036000
P	-2.402743000	-0.572398000	-0.163818000
P	-0.039753000	-1.691216000	1.979326000
F	5.059002000	-0.662657000	-0.563264000
F	5.352087000	-3.250665000	-1.161620000
F	3.194521000	-4.882613000	-1.212404000
F	0.753475000	-3.934349000	-0.662192000
F	-5.027401000	0.782781000	-0.844842000
F	-5.489586000	3.349654000	-1.371750000
F	-3.458111000	5.133201000	-1.358803000
F	-0.964049000	4.346321000	-0.826563000
C	1.578034000	-1.742519000	-0.345858000
C	2.704652000	-0.905730000	-0.320191000
C	3.965361000	-1.429198000	-0.596347000
C	4.141792000	-2.762757000	-0.901859000
C	3.035398000	-3.595969000	-0.926151000
C	1.788220000	-3.074013000	-0.644201000
C	-1.620777000	2.083030000	-0.605172000
C	-2.684335000	1.162122000	-0.643608000
C	-3.979434000	1.610083000	-0.878849000
C	-4.247641000	2.941175000	-1.132039000
C	-3.207094000	3.851348000	-1.120286000
C	-1.925533000	3.407285000	-0.848916000
C	1.462772000	-2.555201000	2.522759000
H	1.401674000	-2.699668000	3.602863000
H	2.350093000	-1.973447000	2.283690000
H	1.532244000	-3.526127000	2.033851000
C	-1.307931000	-2.807660000	2.640425000
H	-1.061117000	-3.049767000	3.675533000
H	-1.315282000	-3.723138000	2.047795000
H	-2.293317000	-2.352051000	2.600398000

C	-0.052224000	-0.254113000	3.084005000
H	-0.954452000	0.333115000	2.935971000
H	0.812984000	0.370013000	2.861164000
H	-0.000320000	-0.592264000	4.120573000
C	3.540586000	1.721153000	-1.180668000
C	4.605797000	2.531184000	-0.807440000
H	4.864031000	2.646083000	0.238016000
C	5.346696000	3.187865000	-1.780035000
H	6.177585000	3.818081000	-1.486484000
C	5.029889000	3.033191000	-3.121643000
H	5.612430000	3.544506000	-3.878542000
C	3.963284000	2.224498000	-3.494905000
H	3.710913000	2.104132000	-4.541474000
C	3.214075000	1.574808000	-2.528579000
H	2.373580000	0.951807000	-2.817533000
C	3.188009000	1.200070000	1.654955000
C	4.036635000	0.320866000	2.320108000
H	4.329153000	-0.614441000	1.861555000
C	4.507930000	0.633972000	3.586616000
H	5.162300000	-0.059337000	4.100888000
C	4.143535000	1.828289000	4.191136000
H	4.512832000	2.069640000	5.180577000
C	3.299473000	2.711126000	3.530254000
H	3.007481000	3.642143000	4.000549000
C	2.815529000	2.394949000	2.271594000
H	2.135744000	3.071032000	1.764252000
C	-3.162216000	-1.687661000	-1.377796000
C	-3.023479000	-3.054580000	-1.136704000
H	-2.516873000	-3.399670000	-0.241950000
C	-3.507977000	-3.980154000	-2.046524000
H	-3.396815000	-5.039033000	-1.846695000
C	-4.117169000	-3.548405000	-3.216599000
H	-4.489134000	-4.270175000	-3.933778000
C	-4.233125000	-2.189756000	-3.472418000
H	-4.690123000	-1.846907000	-4.392884000
C	-3.756921000	-1.261312000	-2.558814000
H	-3.830699000	-0.206750000	-2.786967000
C	-3.528472000	-0.647480000	1.279586000
C	-4.454205000	-1.668406000	1.473931000
H	-4.566289000	-2.451064000	0.735758000
C	-5.245891000	-1.689199000	2.613067000
H	-5.963799000	-2.489130000	2.749121000
C	-5.125036000	-0.691380000	3.568915000
H	-5.743468000	-0.710602000	4.458087000
C	-4.218651000	0.341428000	3.371862000
H	-4.129116000	1.136025000	4.102836000
C	-3.430430000	0.365719000	2.232970000
H	-2.745624000	1.191179000	2.075849000

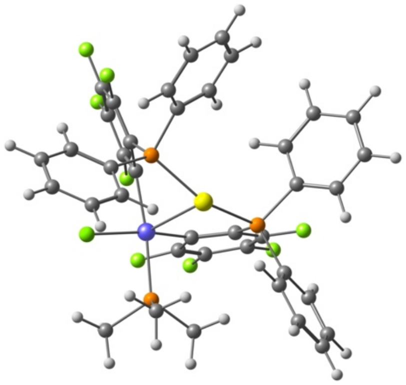

Figure S133. Optimized molecular structure of *cis*-**8NiAu**.

PBE0:

final single point energy: -24996.272468882249 a.u.
final Gibbs free energy: -24995.76706513 a.u.

Table S66. Atomic coordinates for optimized structure of *cis*-**8NiAu**.

Ni	-0.165604000	-1.791843000	-0.219198000
Au	0.259107000	0.890695000	-0.783499000
P	-1.903947000	1.339453000	-0.209812000
Cl	1.538572000	-2.762510000	-1.271281000
C	-2.457602000	-0.074307000	0.772807000
F	-0.079307000	-2.798441000	2.780088000
C	-3.069397000	1.640520000	-1.559669000
C	3.294093000	-0.657079000	2.056543000
C	-2.417464000	-2.383455000	1.281237000
C	-4.160642000	-1.033375000	2.201754000
C	-1.781088000	-1.310149000	0.671431000
C	-3.608882000	0.045832000	1.550828000
C	2.295052000	-0.653183000	1.092798000
C	0.996008000	-2.050859000	2.474086000
C	3.146752000	-1.339207000	3.247266000
C	1.093906000	-1.392399000	1.262947000
F	-5.258704000	-0.905467000	2.942335000
F	4.448670000	-0.002943000	1.875202000
F	-1.941693000	-3.630714000	1.123549000
F	-4.236244000	1.221592000	1.686989000
F	4.106533000	-1.329905000	4.169537000
F	-4.103603000	-3.334083000	2.628916000
F	1.804355000	-2.692163000	4.600637000
C	-3.561867000	-2.272147000	2.044207000
P	2.464069000	0.453390000	-0.334813000
C	3.627281000	-0.147242000	-1.570013000
C	3.181298000	1.993347000	0.312351000
P	-1.354909000	-2.336959000	-2.000017000
C	-1.939309000	2.792778000	0.866884000
C	1.973845000	-2.032565000	3.458406000
C	2.500242000	2.645254000	1.340445000
H	1.597371000	2.209100000	1.753459000
C	2.970651000	3.849146000	1.833878000
H	2.436301000	4.346811000	2.634379000

C	4.119113000	4.419558000	1.297120000
H	4.485355000	5.364419000	1.680206000
C	4.792899000	3.778094000	0.269026000
H	5.688442000	4.218848000	-0.152292000
C	4.328227000	2.565712000	-0.223880000
H	4.864351000	2.067104000	-1.021652000
C	4.721726000	-0.942096000	-1.245464000
H	4.887709000	-1.256459000	-0.223544000
C	5.596526000	-1.346106000	-2.241107000
H	6.444437000	-1.971096000	-1.988597000
C	5.385763000	-0.956439000	-3.557478000
H	6.071216000	-1.277237000	-4.332693000
C	4.294210000	-0.163405000	-3.881920000
H	4.123098000	0.135986000	-4.908794000
C	3.412443000	0.237562000	-2.890288000
H	2.547711000	0.842705000	-3.140156000
C	-2.523874000	1.971644000	-2.798994000
H	-1.446847000	2.037147000	-2.908667000
C	-3.348961000	2.205928000	-3.888259000
H	-2.914768000	2.461424000	-4.847037000
C	-4.724572000	2.099657000	-3.748545000
H	-5.371170000	2.272194000	-4.600449000
C	-5.273797000	1.767676000	-2.516771000
C	-4.453683000	1.542388000	-1.423371000
C	-2.486208000	4.001734000	0.454053000
H	-2.974246000	4.078990000	-0.509888000
C	-2.409885000	5.111951000	1.283752000
H	-2.840895000	6.052121000	0.961479000
C	-1.788686000	5.018254000	2.520290000
H	-1.732417000	5.886268000	3.165923000
C	-1.239946000	3.809501000	2.932022000
H	-0.754957000	3.731825000	3.897597000
C	-1.311060000	2.699629000	2.108245000
H	-0.880530000	1.756837000	2.428657000
H	-6.347816000	1.681870000	-2.405827000
H	-4.898584000	1.286600000	-0.472531000
C	-3.138366000	-1.993950000	-2.068401000
H	-3.536860000	-2.335373000	-3.025330000
H	-3.647122000	-2.517259000	-1.259002000
H	-3.325061000	-0.927061000	-1.963660000
C	-0.747321000	-1.527443000	-3.507925000
H	0.311119000	-1.752315000	-3.628167000
H	-1.308269000	-1.874990000	-4.377376000
H	-0.871561000	-0.448533000	-3.405405000
C	-1.320045000	-4.110203000	-2.387932000
H	-1.879553000	-4.300049000	-3.305761000
H	-0.288106000	-4.434880000	-2.501704000
H	-1.777395000	-4.659995000	-1.564691000

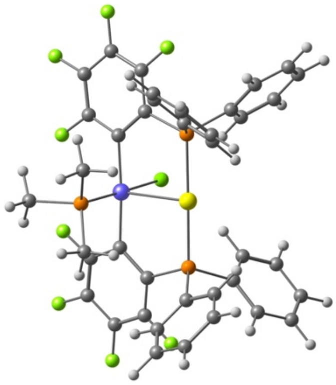

Figure S134. Optimized molecular structure of *trans*-**8NiAu**.

PBE0:
final single point energy: -24996.292894028084 a.u.
final Gibbs free energy: -24995.78694169 a.u.

Table S67. Atomic coordinates for optimized structure of *trans*-**8NiAu**.

Ni	-0.000024000	1.625205000	-0.119816000
Au	0.000083000	-1.071992000	0.106344000
Cl	0.000290000	1.334922000	-2.340825000
F	-5.104033000	-0.101689000	-0.333611000
F	-6.012322000	2.393818000	-0.719981000
F	-4.269387000	4.463924000	-0.765391000
F	-1.662037000	4.063392000	-0.439577000
P	-2.297179000	-1.031543000	0.058915000
P	-0.000353000	1.978769000	1.992542000
C	-1.934357000	1.727911000	-0.160829000
C	-2.865677000	0.671462000	-0.129670000
C	-4.223179000	0.905029000	-0.332253000
C	-4.711877000	2.176472000	-0.539569000
C	-3.816298000	3.232403000	-0.559857000
C	-2.468599000	2.985920000	-0.376665000
C	-3.045697000	-1.765544000	1.531740000
C	-2.626423000	-3.048752000	1.885843000
H	-1.899425000	-3.568218000	1.270651000
C	-3.130507000	-3.656911000	3.022504000
H	-2.804858000	-4.655335000	3.287731000
C	-4.041392000	-2.981663000	3.825488000
H	-4.429869000	-3.454281000	4.719504000
C	-4.445883000	-1.699270000	3.486559000
H	-5.149159000	-1.166024000	4.114504000
C	-3.952280000	-1.090791000	2.341410000
H	-4.267552000	-0.086059000	2.092886000
C	-2.938177000	-1.937031000	-1.373747000
C	-3.917179000	-2.918436000	-1.275704000
H	-4.344976000	-3.169614000	-0.313284000
C	-4.353627000	-3.572031000	-2.419321000
H	-5.117543000	-4.336202000	-2.341167000
C	-3.820116000	-3.243994000	-3.657281000
H	-4.165230000	-3.754904000	-4.548154000
C	-2.843887000	-2.260150000	-3.754989000

H	-2.425597000	-2.000817000	-4.720076000
C	-2.397050000	-1.609702000	-2.617122000
H	-1.633872000	-0.840242000	-2.689751000
C	-0.000856000	3.748319000	2.394830000
H	-0.000950000	3.877707000	3.478719000
H	0.883647000	4.219852000	1.969032000
C	1.402636000	1.339717000	2.948220000
H	1.279963000	1.605829000	3.999402000
H	2.335281000	1.754387000	2.569993000
H	1.438669000	0.254055000	2.855948000
H	-0.885563000	4.219379000	1.968938000
C	-1.403232000	1.338971000	2.947882000
H	-1.280927000	1.605105000	3.999101000
H	-2.336005000	1.753178000	2.569466000
H	-1.438691000	0.253289000	2.855557000
F	5.104191000	-0.101163000	-0.332819000
F	6.012322000	2.394488000	-0.718604000
F	4.269198000	4.464439000	-0.764042000
F	1.661818000	4.063605000	-0.438820000
P	2.297342000	-1.031343000	0.059004000
C	1.934310000	1.728110000	-0.160366000
C	2.865722000	0.671745000	-0.129182000
C	4.223240000	0.905473000	-0.331464000
C	4.711861000	2.176995000	-0.538478000
C	3.816186000	3.232843000	-0.558789000
C	2.468473000	2.986198000	-0.375927000
C	3.045887000	-1.765610000	1.531687000
C	2.626588000	-3.048868000	1.885577000
H	1.899546000	-3.568200000	1.270323000
C	3.130699000	-3.657248000	3.022108000
H	2.805025000	-4.655708000	3.287170000
C	4.041635000	-2.982172000	3.825180000
H	4.430123000	-3.454958000	4.719102000
C	4.446159000	-1.699735000	3.486460000
H	5.149474000	-1.166621000	4.114474000
C	3.952534000	-1.091036000	2.341436000
H	4.267834000	-0.086271000	2.093083000
C	2.938398000	-1.936494000	-1.373838000
C	3.917451000	-2.917867000	-1.275994000
H	4.345250000	-3.169230000	-0.313623000
C	4.353936000	-3.571200000	-2.419747000
H	5.117888000	-4.335351000	-2.341750000
C	3.820414000	-3.242928000	-3.657641000
H	4.165559000	-3.753632000	-4.548619000
C	2.844132000	-2.259117000	-3.755147000
H	2.425829000	-1.999606000	-4.720181000
C	2.397255000	-1.608934000	-2.617144000
H	1.634027000	-0.839509000	-2.689610000

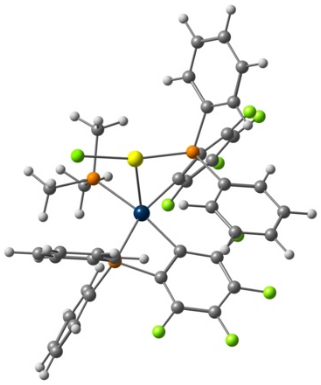

Figure S135. Optimized molecular structure of *cis*-**6PtAu**.

PBE0:

final single point energy: -42507.582661047556 a.u.

final Gibbs free energy: -42507.07717449 a.u.

Table S68. Atomic coordinates for optimized structure of *cis*-**6PtAu**.

Pt	3.415910000	8.710575000	4.101413000
Au	0.577830000	9.228266000	2.904854000
Cl	0.521264000	10.829987000	1.248120000
P	4.438028000	8.447570000	2.044115000
P	0.218013000	7.722391000	4.526573000
P	3.295223000	10.987378000	4.406670000
F	5.222521000	5.518571000	0.546461000
F	4.941669000	3.177528000	1.900128000
F	4.042120000	3.180058000	4.422189000
F	3.340894000	5.433141000	5.662875000
F	-0.524631000	7.065115000	7.452969000
F	0.974398000	7.353816000	9.623850000
F	3.531721000	8.213368000	9.409715000
F	4.582089000	8.776574000	7.033277000
C	3.827957000	6.724824000	3.723077000
C	4.350221000	6.693351000	2.416267000
C	4.732601000	5.527577000	1.786959000
C	4.603887000	4.331722000	2.469275000
C	4.129333000	4.337628000	3.774532000
C	3.757744000	5.521447000	4.394102000
C	2.538007000	8.351459000	5.912691000
C	1.205533000	7.911367000	6.040513000
C	0.705689000	7.559004000	7.290308000
C	1.473269000	7.675027000	8.432891000
C	2.779478000	8.110679000	8.320343000
C	3.286541000	8.417997000	7.071456000
C	1.769765000	11.656807000	5.124368000
H	1.890817000	12.721694000	5.329946000
H	1.549317000	11.125784000	6.050779000
H	0.940337000	11.512348000	4.431842000
C	3.577684000	11.994751000	2.930414000
H	3.527672000	13.056030000	3.179890000
H	2.823253000	11.756730000	2.180484000
H	4.566591000	11.761947000	2.532792000

C	4.559361000	11.600899000	5.557540000
H	5.550757000	11.320400000	5.203311000
H	4.404689000	11.166344000	6.543321000
H	4.492279000	12.688508000	5.622277000
C	6.178290000	8.900013000	1.858263000
C	6.850603000	9.392043000	2.973294000
H	6.309613000	9.519462000	3.904085000
C	8.199631000	9.702880000	2.892856000
H	8.717658000	10.086730000	3.763341000
C	8.880181000	9.523568000	1.697133000
H	9.933434000	9.769015000	1.631450000
C	8.213341000	9.029192000	0.582757000
H	8.745860000	8.886143000	-0.349713000
C	6.865829000	8.716891000	0.659626000
H	6.349258000	8.332480000	-0.211373000
C	3.671523000	8.760133000	0.442444000
C	3.920160000	9.950818000	-0.236301000
H	4.607148000	10.680238000	0.175699000
C	3.296045000	10.201515000	-1.446910000
H	3.495235000	11.127810000	-1.971926000
C	2.416832000	9.270852000	-1.983951000
H	1.929514000	9.469589000	-2.930802000
C	2.161660000	8.087107000	-1.306788000
H	1.476689000	7.357959000	-1.722200000
C	2.785795000	7.831152000	-0.096097000
H	2.588399000	6.903896000	0.427798000
C	0.433055000	6.013582000	3.981481000
C	0.395122000	4.950857000	4.881063000
H	0.237514000	5.130030000	5.937137000
C	0.573360000	3.654735000	4.426594000
H	0.552035000	2.831651000	5.130205000
C	0.791688000	3.412263000	3.076363000
H	0.943560000	2.397846000	2.727591000
C	0.824989000	4.467662000	2.176737000
H	1.000884000	4.283206000	1.123945000
C	0.645961000	5.765579000	2.628407000
H	0.683137000	6.597121000	1.934116000
C	-1.525452000	7.897182000	4.996483000
C	-1.929872000	9.107683000	5.558474000
H	-1.200903000	9.888932000	5.746003000
C	-3.260136000	9.313818000	5.877929000
H	-3.567708000	10.253134000	6.321316000
C	-4.199386000	8.321324000	5.622101000
H	-5.241721000	8.485663000	5.867211000
C	-3.801246000	7.123962000	5.047995000
H	-4.530611000	6.349742000	4.842411000
C	-2.465665000	6.908357000	4.735403000
H	-2.160920000	5.969306000	4.290983000

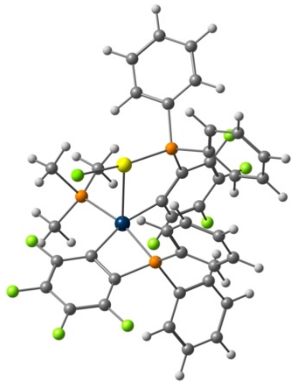

Figure S136. Optimized molecular structure of *trans*-**6PtAu**.

PBE0:

final single point energy: -42507.578767482679 a.u.

final Gibbs free energy: -42507.07367558 a.u.

Table S69. Atomic coordinates for optimized structure of *trans*-**6PtAu**.

Pt	0.011258000	-0.063278000	0.109560000
Au	0.001027000	0.000349000	3.099655000
Cl	-2.216929000	0.342199000	3.646028000
P	-0.405100000	-2.322825000	-0.154230000
P	2.191642000	-0.410548000	2.912026000
P	0.045122000	2.220145000	-0.001303000
F	-3.350647000	-3.807552000	-0.713670000
F	-5.653917000	-2.357085000	-0.725873000
F	-5.563727000	0.297885000	-0.335579000
F	-3.279636000	1.582982000	0.069734000
F	5.062401000	-0.756372000	2.133795000
F	6.179594000	-0.644710000	-0.290343000
F	4.630326000	-0.216584000	-2.469652000
F	2.008471000	0.105414000	-2.235049000
C	-2.019674000	-0.427010000	-0.128378000
C	-2.110614000	-1.820235000	-0.344054000
C	-3.302539000	-2.487066000	-0.535325000
C	-4.479513000	-1.762366000	-0.536682000
C	-4.426851000	-0.389681000	-0.334922000
C	-3.216841000	0.256948000	-0.132418000
C	2.065143000	-0.160717000	0.122225000
C	2.892181000	-0.368781000	1.241187000
C	4.267140000	-0.529532000	1.085176000
C	4.866330000	-0.481332000	-0.154984000
C	4.070721000	-0.264644000	-1.266162000
C	2.708212000	-0.108695000	-1.105880000
C	-0.774308000	3.115150000	1.342433000
H	-0.763890000	4.185243000	1.126556000
H	-0.238868000	2.925705000	2.272823000
H	-1.797082000	2.765801000	1.457918000
C	1.689340000	2.983207000	-0.037003000
H	2.231422000	2.648734000	-0.920809000
H	2.252157000	2.691489000	0.848995000
H	1.586116000	4.069303000	-0.064835000

C	-0.718153000	2.846028000	-1.520676000
H	-1.761260000	2.539827000	-1.563504000
H	-0.189410000	2.426792000	-2.376860000
H	-0.652885000	3.935350000	-1.548962000
C	0.267145000	-3.058914000	-1.652511000
C	1.646388000	-3.260165000	-1.721013000
H	2.274086000	-3.014940000	-0.871566000
C	2.216473000	-3.765950000	-2.876734000
H	3.287792000	-3.918707000	-2.925680000
C	1.416333000	-4.066159000	-3.972055000
H	1.863747000	-4.456570000	-4.878117000
C	0.045612000	-3.861700000	-3.908024000
H	-0.579489000	-4.092736000	-4.761981000
C	-0.531447000	-3.358150000	-2.751012000
H	-1.600947000	-3.193838000	-2.709493000
C	-0.318508000	-3.590072000	1.132956000
C	-0.876387000	-3.294478000	2.376072000
H	-1.343960000	-2.331378000	2.547208000
C	-0.843132000	-4.232487000	3.392993000
H	-1.274510000	-3.992053000	4.357085000
C	-0.257380000	-5.472994000	3.175906000
H	-0.225531000	-6.204199000	3.974563000
C	0.287010000	-5.774205000	1.936948000
H	0.740067000	-6.742612000	1.762324000
C	0.259026000	-4.836858000	0.914753000
H	0.683235000	-5.082160000	-0.050242000
C	2.579221000	-2.040031000	3.596064000
C	2.357295000	-2.256742000	4.956091000
H	1.999685000	-1.447780000	5.583121000
C	2.592344000	-3.504412000	5.507037000
H	2.425254000	-3.664386000	6.565262000
C	3.033412000	-4.548230000	4.703099000
H	3.211710000	-5.525946000	5.134194000
C	3.231672000	-4.340998000	3.347151000
H	3.558809000	-5.156138000	2.713667000
C	3.005313000	-3.090325000	2.791589000
H	3.155611000	-2.938626000	1.729899000
C	3.138374000	0.795968000	3.887229000
C	2.725911000	2.126351000	3.831934000
H	1.852806000	2.393533000	3.248067000
C	3.417057000	3.103075000	4.529837000
H	3.089877000	4.134433000	4.478616000
C	4.514442000	2.754080000	5.305212000
H	5.050138000	3.514735000	5.860318000
C	4.919133000	1.428371000	5.375940000
H	5.771472000	1.151854000	5.984724000
C	4.238137000	0.450781000	4.666656000
H	4.565307000	-0.579104000	4.721335000

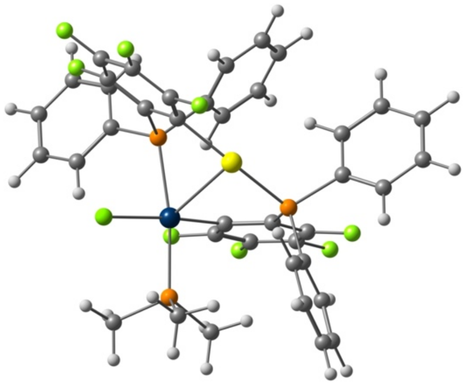

Figure S137. Optimized molecular structure of *cis-trans*-**7PtAu**.

PBE0:

final single point energy: -42507.585445467012 a.u.

final Gibbs free energy: -42507.07904240 a.u.

Table S70. Atomic coordinates for optimized structure of *cis-trans*-**7PtAu**.

Pt	3.810649000	6.694697000	4.786912000
Au	1.274789000	5.535122000	3.772667000
Cl	4.228663000	4.963782000	6.372641000
P	4.250067000	5.144316000	3.082401000
P	0.717782000	7.726921000	3.491080000
C	2.211509000	8.697577000	3.117088000
F	0.101211000	2.526050000	4.421783000
C	-0.130532000	8.518986000	4.875836000
C	4.068133000	2.314300000	3.457412000
C	4.568320000	9.025455000	3.191328000
C	3.222308000	10.604648000	2.011534000
C	3.477915000	8.251566000	3.560154000
C	2.111473000	9.871428000	2.373936000
C	3.448032000	3.551256000	3.462755000
C	1.405840000	2.498288000	4.102310000
C	3.361204000	1.167206000	3.786423000
C	2.082176000	3.653970000	3.797466000
F	3.102818000	11.718298000	1.295094000
F	5.355188000	2.149128000	3.148574000
F	5.809781000	8.684991000	3.574283000
F	0.928902000	10.362427000	1.994417000
F	3.967701000	-0.016780000	3.790771000
F	5.546865000	10.863128000	2.086234000
F	1.349259000	0.156882000	4.430759000
C	4.467253000	10.169478000	2.423229000
C	3.696081000	5.521781000	1.389855000
C	6.038368000	4.907663000	2.862751000
P	3.669935000	8.097988000	6.589998000
C	-0.369479000	7.909480000	2.052805000
C	2.022356000	1.257029000	4.110918000
C	6.892493000	5.184180000	3.926605000
H	6.480456000	5.525400000	4.868229000
C	8.262030000	5.019435000	3.781266000
H	8.919848000	5.235533000	4.614421000

C	8.786185000	4.586876000	2.572164000
H	9.856525000	4.463439000	2.457362000
C	7.937825000	4.317011000	1.505719000
H	8.343347000	3.981073000	0.558916000
C	6.569838000	4.477014000	1.648428000
H	5.914736000	4.263051000	0.813245000
C	2.804201000	4.713442000	0.691182000
H	2.405415000	3.819925000	1.152208000
C	2.423760000	5.047318000	-0.601735000
H	1.729889000	4.409253000	-1.135419000
C	2.923948000	6.191399000	-1.204061000
H	2.620660000	6.453746000	-2.210466000
C	3.817781000	7.000022000	-0.512889000
H	4.219335000	7.892161000	-0.978056000
C	4.205849000	6.664354000	0.773127000
H	4.924307000	7.285242000	1.291643000
C	-0.854589000	7.698096000	5.738668000
H	-0.870722000	6.626781000	5.570581000
C	-1.535976000	8.245933000	6.814510000
H	-2.094660000	7.601614000	7.482112000
C	-1.485491000	9.613849000	7.042094000
H	-2.006911000	10.041475000	7.889931000
C	-0.763164000	10.435142000	6.186546000
C	-0.092001000	9.892573000	5.102190000
C	-1.677019000	8.367279000	2.155243000
H	-2.070182000	8.689034000	3.111793000
C	-2.479825000	8.418357000	1.023662000
H	-3.498913000	8.776770000	1.105752000
C	-1.979942000	8.017998000	-0.206732000
H	-2.608647000	8.062765000	-1.087850000
C	-0.672903000	7.555994000	-0.307365000
H	-0.277390000	7.237543000	-1.264521000
C	0.127904000	7.493386000	0.819285000
H	1.143295000	7.121831000	0.741166000
H	-0.721811000	11.502993000	6.363343000
H	0.462703000	10.543759000	4.439046000
C	3.433360000	9.865177000	6.282948000
H	3.373818000	10.389140000	7.238189000
H	4.274742000	10.253121000	5.709497000
H	2.516220000	10.029441000	5.720928000
C	2.346960000	7.662889000	7.741712000
H	2.451028000	6.613264000	8.013993000
H	2.407594000	8.289494000	8.633137000
H	1.383785000	7.811736000	7.254505000
C	5.188374000	8.051235000	7.572841000
H	5.085271000	8.701235000	8.443580000
H	5.375993000	7.026454000	7.888380000
H	6.020289000	8.393124000	6.956692000

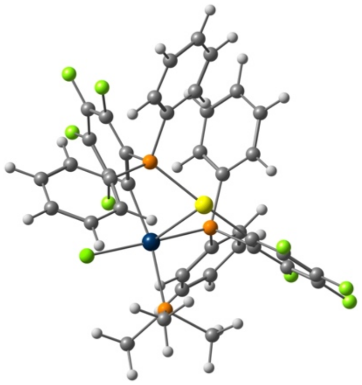

Figure S138. Optimized molecular structure of *cis*-*cis*-**7PtAu**.

PBE0:

final single point energy: -42507.582536493195 a.u.
final Gibbs free energy: -42507.07541562 a.u.

Table S71. Atomic coordinates for optimized structure of *cis*-*cis*-**7PtAu**.

Pt	5.385623000	12.945153000	8.122643000
Au	2.791452000	13.136182000	6.767789000
Cl	6.053828000	15.116892000	8.781088000
P	6.743444000	13.307895000	6.273382000
P	2.191886000	14.316893000	8.625475000
P	4.783095000	10.848088000	7.587955000
F	1.321961000	14.274702000	11.559841000
F	2.538653000	13.149163000	13.633644000
F	4.941886000	11.941866000	13.311190000
F	6.101863000	11.843382000	10.926950000
F	5.436754000	8.704174000	5.312910000
F	4.621634000	8.772778000	2.802243000
F	3.028905000	10.763272000	1.914543000
F	2.212410000	12.692619000	3.581980000
C	4.293767000	12.957351000	9.876727000
C	3.040736000	13.579618000	10.058188000
C	2.478446000	13.645842000	11.329620000
C	3.100293000	13.088247000	12.429432000
C	4.329813000	12.482237000	12.264055000
C	4.902177000	12.446451000	11.006446000
C	3.425110000	11.761578000	5.383963000
C	4.268006000	10.722430000	5.834778000
C	4.643180000	9.715737000	4.959625000
C	4.229367000	9.729508000	3.637129000
C	3.416330000	10.749400000	3.183924000
C	3.021365000	11.738771000	4.069523000
C	2.578720000	16.079908000	8.625536000
C	2.740525000	16.712448000	7.396135000
H	2.645117000	16.137867000	6.481338000
C	3.040506000	18.065051000	7.343882000
H	3.170616000	18.551410000	6.384808000
C	3.187209000	18.787246000	8.519346000
H	3.432230000	19.841874000	8.480137000
C	3.028408000	18.158088000	9.747479000

H	3.148378000	18.719720000	10.665905000
C	2.723465000	16.808071000	9.803241000
H	2.610567000	16.325550000	10.765522000
C	0.410771000	14.182914000	8.931728000
C	-0.427570000	15.290894000	8.930019000
H	-0.017382000	16.284906000	8.801976000
C	-1.795367000	15.122500000	9.099011000
H	-2.445667000	15.989022000	9.097174000
C	-2.326878000	13.853681000	9.274348000
H	-3.394213000	13.726534000	9.409571000
C	-1.488452000	12.744924000	9.273328000
H	-1.898038000	11.750799000	9.407692000
C	-0.125935000	12.906899000	9.094148000
H	0.524703000	12.039812000	9.083121000
C	6.095637000	9.650433000	7.970469000
C	7.382433000	10.112240000	8.224646000
H	7.581647000	11.176371000	8.189050000
C	8.394628000	9.223994000	8.558405000
H	9.392275000	9.596016000	8.757298000
C	8.121249000	7.868475000	8.652572000
H	8.908051000	7.173157000	8.919727000
C	6.832695000	7.402269000	8.418105000
H	6.613484000	6.344730000	8.501685000
C	5.823733000	8.287144000	8.082428000
H	4.821969000	7.916368000	7.905394000
C	3.362715000	10.145112000	8.486473000
C	3.526881000	9.829761000	9.835225000
H	4.494036000	9.948106000	10.306034000
C	2.463545000	9.344495000	10.575926000
H	2.603379000	9.112200000	11.624701000
C	1.225933000	9.151125000	9.974324000
H	0.393544000	8.771378000	10.554436000
C	1.063919000	9.435191000	8.627801000
H	0.106406000	9.274590000	8.147204000
C	2.126183000	9.934287000	7.885136000
H	1.984344000	10.154805000	6.836227000
C	7.059718000	12.053825000	5.000717000
H	7.372304000	11.112134000	5.451748000
H	6.164170000	11.888831000	4.406049000
H	7.855890000	12.420295000	4.350587000
C	6.121542000	14.704893000	5.303597000
H	6.810948000	14.936835000	4.490116000
H	5.146193000	14.436947000	4.895432000
H	6.007672000	15.568103000	5.957725000
C	8.427824000	13.779237000	6.752130000
H	8.986186000	14.092037000	5.868310000
H	8.383860000	14.589726000	7.476607000
H	8.926077000	12.921604000	7.205819000

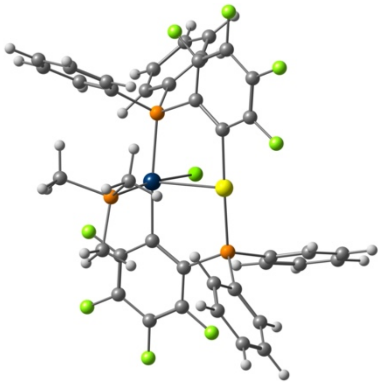

Figure S139. Optimized molecular structure of *trans*-**7PtAu**.

PBE0:

final single point energy: -42507.576772377470 a.u.

final Gibbs free energy: -42507.06950295 a.u.

Table S72. Atomic coordinates for optimized structure of *trans*-**7PtAu**.

Pt	22.186776000	4.087022000	5.967883000
Au	24.519499000	5.652338000	6.443177000
Cl	21.329755000	6.037300000	4.906620000
P	24.997935000	5.272358000	4.236482000
P	21.488738000	4.891461000	8.044823000
P	23.078817000	2.167911000	6.713229000
F	25.171548000	4.240681000	1.420008000
F	23.586232000	2.519929000	0.145049000
F	21.425064000	1.480618000	1.404391000
F	20.875679000	2.134913000	3.922921000
F	21.338718000	5.972399000	10.869623000
F	23.209072000	7.200668000	12.296948000
F	25.633348000	7.722057000	11.215709000
F	26.176052000	7.021954000	8.706063000
C	22.753705000	3.569651000	4.069463000
C	23.876267000	4.100331000	3.407438000
C	24.133499000	3.735532000	2.089294000
C	23.321615000	2.854327000	1.404249000
C	22.216083000	2.330247000	2.047316000
C	21.955794000	2.697653000	3.354285000
C	24.029382000	6.073267000	8.393615000
C	22.775994000	5.789117000	8.975975000
C	22.520339000	6.188080000	10.285550000
C	23.473653000	6.834972000	11.047508000
C	24.710298000	7.102136000	10.491556000
C	24.956861000	6.721993000	9.185013000
C	24.020801000	1.212280000	5.494431000
H	24.480436000	0.365323000	6.006645000
H	24.795020000	1.829731000	5.042667000
H	23.361509000	0.843572000	4.710605000
C	21.901126000	0.934189000	7.316660000
H	22.444586000	0.027832000	7.589565000
H	21.194219000	0.709079000	6.518065000
H	21.354601000	1.305781000	8.179387000

C	24.301009000	2.361010000	8.033360000
H	23.875798000	2.880360000	8.886765000
H	25.132869000	2.953612000	7.653015000
H	24.656880000	1.377389000	8.344200000
C	24.907695000	6.814070000	3.292322000
C	25.816443000	7.824518000	3.608603000
H	26.578436000	7.653169000	4.360932000
C	25.745932000	9.048408000	2.965962000
H	26.457894000	9.826627000	3.212751000
C	24.759353000	9.278814000	2.014836000
H	24.701596000	10.238627000	1.515750000
C	23.844343000	8.282169000	1.711861000
H	23.067435000	8.460451000	0.978365000
C	23.915271000	7.051289000	2.348820000
H	23.186264000	6.284770000	2.119557000
C	26.682953000	4.632478000	3.999161000
C	27.511348000	5.030172000	2.953891000
H	27.160942000	5.750518000	2.226444000
C	28.789089000	4.504250000	2.842021000
H	29.429430000	4.822006000	2.028017000
C	29.245869000	3.573250000	3.764862000
H	30.244560000	3.163515000	3.672701000
C	28.425364000	3.175838000	4.811454000
H	28.780048000	2.458591000	5.541653000
C	27.153656000	3.712139000	4.934079000
H	26.523952000	3.425157000	5.768068000
C	20.088666000	6.045122000	7.890412000
C	18.987406000	5.647106000	7.131709000
H	19.000438000	4.697016000	6.612175000
C	17.891751000	6.480752000	6.992709000
H	17.042542000	6.155576000	6.404018000
C	17.895979000	7.739252000	7.579468000
H	17.043372000	8.397188000	7.461448000
C	19.005408000	8.158866000	8.296371000
H	19.028324000	9.148360000	8.736688000
C	20.097049000	7.315989000	8.452899000
H	20.959070000	7.670502000	9.001311000
C	20.888568000	3.621580000	9.218650000
C	19.634204000	3.044040000	9.028447000
H	18.979518000	3.397729000	8.244729000
C	19.206851000	2.002033000	9.835903000
H	18.228011000	1.567933000	9.671865000
C	20.028647000	1.513174000	10.841555000
H	19.696568000	0.694337000	11.468022000
C	21.275065000	2.086205000	11.042948000
H	21.922272000	1.723017000	11.832035000
C	21.699780000	3.134143000	10.240349000
H	22.668618000	3.577627000	10.430553000

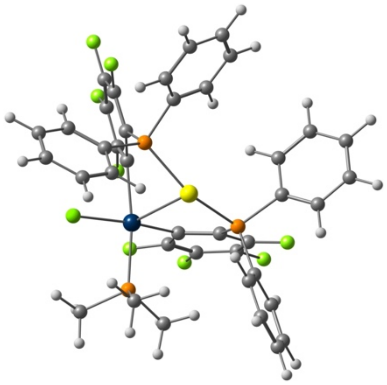

Figure S140. Optimized molecular structure of *cis*-**8PtAu**.

PBE0:

final single point energy: -42507.562182372618 a.u.
final Gibbs free energy: -42507.05762890 a.u.

Table S73. Atomic coordinates for optimized structure of *cis*-**8PtAu**.

Pt	3.871085000	6.318139000	4.566599000
Au	1.400221000	4.921430000	4.168702000
Cl	4.828278000	4.809908000	6.139591000
P	0.496244000	6.878834000	3.418388000
C	1.855207000	7.933564000	2.848611000
F	5.890628000	6.515003000	2.116571000
C	-0.519720000	7.807551000	4.591118000
C	4.722075000	2.631465000	2.275800000
C	4.090188000	8.726606000	2.950281000
C	2.505747000	9.914736000	1.610317000
C	3.177953000	7.741271000	3.320974000
C	1.555745000	8.997885000	1.998542000
C	4.073185000	3.543537000	3.098871000
C	5.442968000	5.253079000	2.210640000
C	5.715185000	3.020020000	1.400371000
C	4.447450000	4.913403000	3.109210000
F	2.198008000	10.927787000	0.805240000
F	4.416450000	1.328989000	2.296134000
F	5.337018000	8.730034000	3.442638000
F	0.313346000	9.191828000	1.539685000
F	6.319669000	2.135435000	0.611788000
F	4.711319000	10.676344000	1.785583000
F	7.016720000	4.753219000	0.522768000
C	3.788190000	9.779272000	2.109468000
P	2.620187000	2.985042000	4.036687000
C	3.075722000	2.030784000	5.494009000
C	1.733196000	1.830538000	2.949728000
P	3.429578000	7.680621000	6.346354000
C	-0.543853000	6.522083000	1.979815000
C	6.067773000	4.352727000	1.362266000
C	1.289514000	2.307871000	1.716818000
H	1.511474000	3.327022000	1.419590000
C	0.562604000	1.486465000	0.873373000
H	0.224428000	1.864060000	-0.084196000

C	0.260168000	0.185760000	1.259649000
H	-0.313823000	-0.455730000	0.601931000
C	0.691612000	-0.287600000	2.489403000
H	0.457916000	-1.300417000	2.794994000
C	1.429538000	0.530435000	3.334572000
H	1.770822000	0.151287000	4.289673000
C	4.252393000	1.293026000	5.568544000
H	4.955098000	1.295690000	4.745810000
C	4.536974000	0.566245000	6.713264000
H	5.458260000	-0.000292000	6.773399000
C	3.650307000	0.570306000	7.782158000
H	3.880041000	0.005081000	8.677436000
C	2.475078000	1.304614000	7.707969000
H	1.785269000	1.316114000	8.543082000
C	2.189059000	2.037832000	6.566927000
H	1.282134000	2.629995000	6.511229000
C	-1.116987000	7.079820000	5.619455000
H	-0.946286000	6.010725000	5.687490000
C	-1.915557000	7.717244000	6.556201000
H	-2.374670000	7.143151000	7.351610000
C	-2.111538000	9.088421000	6.479476000
H	-2.726727000	9.590415000	7.216439000
C	-1.513552000	9.819124000	5.461457000
C	-0.723251000	9.184051000	4.516795000
C	-1.924722000	6.672435000	2.013058000
H	-2.407403000	7.090436000	2.888006000
C	-2.687004000	6.287072000	0.918739000
H	-3.763348000	6.406632000	0.947274000
C	-2.075134000	5.754829000	-0.206309000
H	-2.673015000	5.455546000	-1.058616000
C	-0.693653000	5.605170000	-0.239493000
H	-0.211304000	5.190052000	-1.116216000
C	0.070896000	5.982376000	0.850758000
H	1.148278000	5.857901000	0.827217000
H	-1.661238000	10.890484000	5.401818000
H	-0.265423000	9.769297000	3.731728000
C	2.705029000	9.315534000	6.051174000
H	2.556392000	9.824690000	7.004787000
H	3.373553000	9.907288000	5.426220000
H	1.747570000	9.215743000	5.543216000
C	2.287428000	6.919958000	7.528874000
H	2.689611000	5.952898000	7.829153000
H	2.158412000	7.560351000	8.403108000
H	1.323748000	6.767854000	7.042062000
C	4.903554000	8.075464000	7.322941000
H	4.627937000	8.668115000	8.197050000
H	5.383328000	7.148548000	7.632824000
H	5.597389000	8.642559000	6.701539000

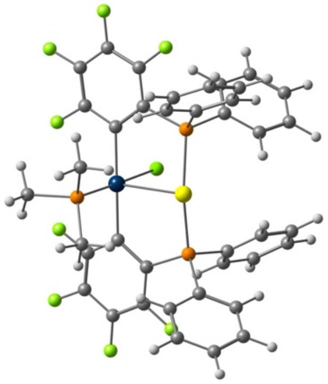

Figure S141. Optimized molecular structure of *trans*-**8PtAu**.

PBE0:

final single point energy: -42507.572657643221 a.u.
final Gibbs free energy: -42507.06734400 a.u.

Table S74. Atomic coordinates for optimized structure of *trans*-**8PtAu**.

Pt	3.860088000	6.055563000	6.695621000
Au	5.970151000	6.054787000	8.555785000
Cl	5.517531000	6.054683000	4.978763000
F	5.564629000	0.907653000	7.686160000
F	3.584274000	-0.153874000	6.247224000
F	1.802008000	1.462942000	5.007223000
F	1.982571000	4.097403000	5.207802000
P	6.152133000	3.777263000	8.265773000
P	2.243757000	6.056828000	8.213907000
C	3.865425000	3.986649000	6.644865000
C	4.779745000	3.120644000	7.285411000
C	4.676786000	1.739376000	7.132264000
C	3.673630000	1.166451000	6.379980000
C	2.770059000	1.996409000	5.742583000
C	2.893429000	3.366718000	5.876354000
C	6.304202000	2.726513000	9.738484000
C	7.539014000	2.269002000	10.185892000
H	8.438669000	2.481513000	9.623155000
C	7.621894000	1.521487000	11.351863000
H	8.587538000	1.165165000	11.689781000
C	6.477048000	1.224298000	12.075963000
H	6.544643000	0.637409000	12.983930000
C	5.242362000	1.675645000	11.628423000
H	4.341789000	1.439798000	12.182503000
C	5.155598000	2.426047000	10.467888000
H	4.187021000	2.761049000	10.119488000
C	7.659626000	3.536572000	7.287134000
C	7.674067000	2.799953000	6.107794000
H	6.771277000	2.328200000	5.743195000
C	8.845296000	2.690055000	5.372987000
H	8.845583000	2.122067000	4.450736000
C	10.005931000	3.311159000	5.809863000
H	10.917306000	3.226577000	5.230406000

C	9.994212000	4.054965000	6.982213000
H	10.893217000	4.555257000	7.320888000
C	8.824417000	4.175925000	7.712690000
H	8.809046000	4.781081000	8.612199000
C	0.579167000	6.062004000	7.500857000
H	-0.162895000	6.061863000	8.301403000
H	0.453480000	6.948583000	6.881106000
C	2.231101000	7.458938000	9.356371000
H	1.381450000	7.372026000	10.035385000
H	2.169648000	8.396037000	8.805274000
H	3.157217000	7.442716000	9.931237000
H	0.449912000	5.178452000	6.877482000
C	2.223865000	4.651249000	9.351937000
H	1.373620000	4.739484000	10.030033000
H	2.159531000	3.716060000	8.797921000
H	3.149168000	4.662136000	9.928216000
F	5.561026000	11.203546000	7.692458000
F	3.579032000	12.265494000	6.256497000
F	1.799606000	10.649098000	5.011772000
F	1.984179000	8.014381000	5.205885000
P	6.153962000	8.331849000	8.262549000
C	3.865561000	8.124544000	6.644800000
C	4.779077000	8.990287000	7.286941000
C	4.674245000	10.371715000	7.137002000
C	3.670223000	10.944982000	6.386045000
C	2.768174000	10.115303000	5.746207000
C	2.893455000	8.744834000	5.876809000
C	6.313663000	9.380681000	9.735681000
C	7.551109000	9.837413000	10.176559000
H	8.447373000	9.625770000	9.608054000
C	7.640852000	10.582669000	11.343485000
H	8.608442000	10.938377000	11.676455000
C	6.500259000	10.878369000	12.074863000
H	6.573176000	11.463491000	12.983561000
C	5.262941000	10.427756000	11.633817000
H	4.365703000	10.662436000	12.193775000
C	5.169323000	9.679513000	10.472431000
H	4.198778000	9.344707000	10.129282000
C	7.657939000	8.571942000	7.278350000
C	7.670982000	9.317951000	6.104878000
H	6.769004000	9.796829000	5.747698000
C	8.839679000	9.428382000	5.366192000
H	8.838872000	10.003770000	4.448537000
C	9.999194000	8.798360000	5.793269000
H	10.908532000	8.883306000	5.210672000
C	9.988839000	8.045310000	6.959669000
H	10.886823000	7.538035000	7.290561000
C	8.821509000	7.924086000	7.694080000
H	8.806963000	7.312166000	8.588982000

14. Literature:

(S1) Wächtler, E.; Privér, S. H.; Wagler, J.; Heine, T.; Zhechkov, L.; Bennett, M. A.; Bhargava, S. K., Metallophilic Contacts in 2-$C_6F_4PPh_2$ Bridged Heterobinuclear Complexes: A Crystallographic and Computational Study, *Inorg. Chem.* **2015**, *54*, 6947.

(S2) Bhargava, S. K.; Privér, S. H.; Willis, A. C.; Bennett, M. A., Preparation, Structure, and Reactivity of Dipalladium(I) Complexes Containing the Carbanion 2-$C_6F_4PPh_2$: Coexistence of Distinct, Noninterconverting Head-to-Head [Dipalladium(0/II)] and Head-to-Tail [Dipalladium(I)] Species, *Organometallics* **2012**, *31*, 5561.

(S3) Cordero, B.; Gómez, V.; Platero-Prats, A. E.; Revés, M.; Echeverría, J.; Cremades, E.; Barragán, F.; Alvarez, S., Covalent radii revisited, *J. Chem. Soc., Dalton Trans.* **2008**, 2832.

(S4) Batsanov, S. S., Van der Waals Radii of Elements, *Inorg. Mater.* **2001**, *37(9)*, 871.

(S5) Cambridge Crystallographic Data Center, ConQuest Version 2021.3.0 (Build 333817), **2022**, https://www.ccdc.cam.ac.uk/structures/

(S6) Gericke, R.; Bennett, M. A.; Privér, S. H.; Bhargava, S. K., Formation of Heterobimetallic Complexes by Addition of d^{10}-Metal Ions to *cis*-[(dppe)M(κC-2-$C_6F_4PPh_2$)$_2$] (M = Ni, Pd, and Pt), *Organometallics* **2017**, *36*, 3178.

(S7) a) Bullock, J. P.; Bond, A. M.; Boeré, R. T.; Gietz, T. M.; Roemmele, T. L.; Seagrave, S. D.; Masuda, J. D.; Parvez, M., Synthesis, Characterization, and Electrochemical Studies of PPh_{3-n}(dipp)$_n$ (dipp = 2,6-Diisopropylphenyl): Steric and Electronic Effects on the Chemical and Electrochemical Oxidation of a Homologous Series of Triarylphosphines and the Reactivities of the Corresponding Phosphoniumyl Radical Cations, *J. Am. Chem. Soc.* **2013**, *135*, 11205. b) Churchill, M. R.; Rotella, F. J., Molecules with an M_4X_4 Core. 10.[1] Failure of the 1:1:1 Tricyclohexylphosphine-Copper(I)-Chloride Complex to Form a Tetramer. Crystal Structure of Dimeric (Tricyclohexylphosphine)copper(I) Chloride, [(P(cHx)$_3$)CuC1]$_2$, *Inorg. Chem.* **1979**, *18(1)*, 166. c) Ramaprabhu, S.; Amstutz, N; Lucken, E. A. C.; Bernardinelli, G., Copper-63,65 Nuclear Quadrupole Resonance of Complexes of Copper(I) Halides with Phosphorus-containing Ligands, *J. Chem. Soc., Dalton Trans.* **1993**, *0(6)*, 871. d) Fujihara, T.; Semba, K.; Terao, J.; Tsuji, Y., Copper-Catalyzed Hydrosilylation with a Bowl-Shaped Phosphane Ligand: Preferential Reduction of a Bulky Ketone in the Presence of an Aldehyde, *Angew. Chem. Int. Ed.* **2010**, *49*, 1472. e) Ag Qi, L.; Li, Q.; Hong, X.; Liu, L.; Zhong, X.-X.; Chen, Q.; Li, F.-B.; Liu, Q.; Qin, H.-M.; Wong, W.-Y., Synthesis, characterization and luminescent properties of three-coordinate copper(I) halide complexes containing 2-(diphenylphosphino)biphenyl, *J.Coord. Chem* **2016**, *69(24)*, 3692. f) Bowmaker, G. A.; Effendy; Harvey, P. J.; Healy, P. C.; Skelton, B. W.; White, A. H., Spectroscopicand structural studies on 1:1 adducts of silver(I)salts with tricyclohexylphosphine, *J. Chem. Soc., Dalton Trans.* **1996**, *0(12)*, 2450. g) Grirrane, A.; Álvarez, E.; García, H.; Avelino Corma, A., Catalytic Activity of Cationic and Neutral Silver(I)–XPhos Complexes with Nitrogen Ligands or Tolylsulfonate for Mannich and Aza-Diels–Alder Coupling Reactions, *Chem. Eur. J.* **2016**, *22*, 340. h) Sabounchei, S. J.; Pourshahbaz, M.; Salehzadeh, S.; Bayat, M.; Karamian,R.; Asadbegy, M.; Khavasi, H. R., New chlorine bridged binuclear silver(I) complexes of bidentate

phosphorus ylides: Synthesis, spectroscopy, theoretical and anti-bacterial studies, *Polyhedron* **2015**, *85*, 652.

(S8) Bennett, M. A.; Bhargava, S. K.; Mirzadeh, N.; Privér, S. H.; Wagler, J.; Willis, A. C., Synthesis and interconversions of digold(I), tetragold(I), digold(II), gold(I)–gold(III) and digold(III) complexes of fluorine-substituted aryl carbanions, *Dalton Trans.* **2009**, *0(36)*, 7537.

(S9) Yang, H.; Gabbaï, F. P., Activation of a Hydroamination Gold Catalyst by Oxidation of a Redox-Noninnocent Chlorostibine Z-Ligand, *J. Am. Chem. Soc.* **2015**, *137*, 13425.

(S10) a) Ge, S.; Hartwig, J. F., Nickel-Catalyzed Asymmetric α-Arylation and Heteroarylation of Ketones with Chloroarenes: Effect of Halide on Selectivity, Oxidation State, and Room-Temperature Reactions, *J. Am. Chem. Soc.* **2011**, *133*, 16330. b) Smith, E. E.; Du, G.; Fanwick, P. E.; Abu-Omar, M. M., Dehydrocoupling of Organosilanes with a Dinuclear Nickel Hydride Catalyst and Isolation of a Nickel Silyl Complex, *Organometallics* **2010**, *29*, 6527.
